# Selective C–O bond formation *via* a photocatalytic radical coupling strategy: access to perfluoroalkoxylated (OR_F_) arenes and heteroarenes†
†Electronic supplementary information (ESI) available. See DOI: 10.1039/c7sc01684k



**DOI:** 10.1039/c7sc01684k

**Published:** 2017-06-05

**Authors:** Johnny W. Lee, Dominique N. Spiegowski, Ming-Yu Ngai

**Affiliations:** a Department of Chemistry , Stony Brook University , Stony Brook , New York 11794-3400 , USA . Email: ming-yu.ngai@stonybrook.edu; b Institute of Chemical Biology and Drug Discovery , Stony Brook University , Stony Brook , New York 11794-3400 , USA

## Abstract

Development of an efficient process that employs commercially available and cost effective reagents for the synthesis of perfluoroalkoxylated aromatic compounds (Ar–OR_F_) remains a daunting challenge in organic synthesis. Herein, we report the first catalytic protocol using readily available perfluoroalkyl iodides (R_F_I) and *N*-(hetero)aryl-*N*-hydroxylamides to access a wide range of perfluoroalkoxylated (hetero)arenes. Mild reaction conditions allow for selective O–R_F_ bond formation over a broad substrate scope and are tolerant of a wide variety of functional groups. Mechanistic studies suggest the formation and recombination of persistent *N*-hydroxyl radicals and transient *R*_F_ radicals under photocatalytic reaction conditions to generate N–OR_F_ compounds that rearrange to afford the desired products.

## Abstract 2

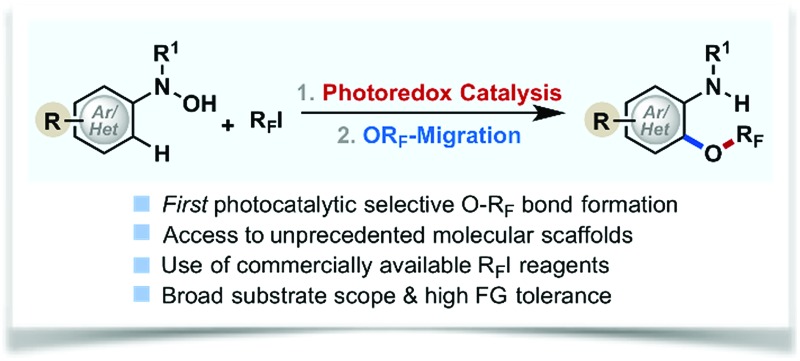
Synthesis of perfluoroalkoxylated (hetero)arenes (Ar–OR_F_) from readily available perfluoroalkyl iodides (R_F_–I) through photocatalytic selective O–R_F_ bond formation.

## Introduction

**Fig. 1:**
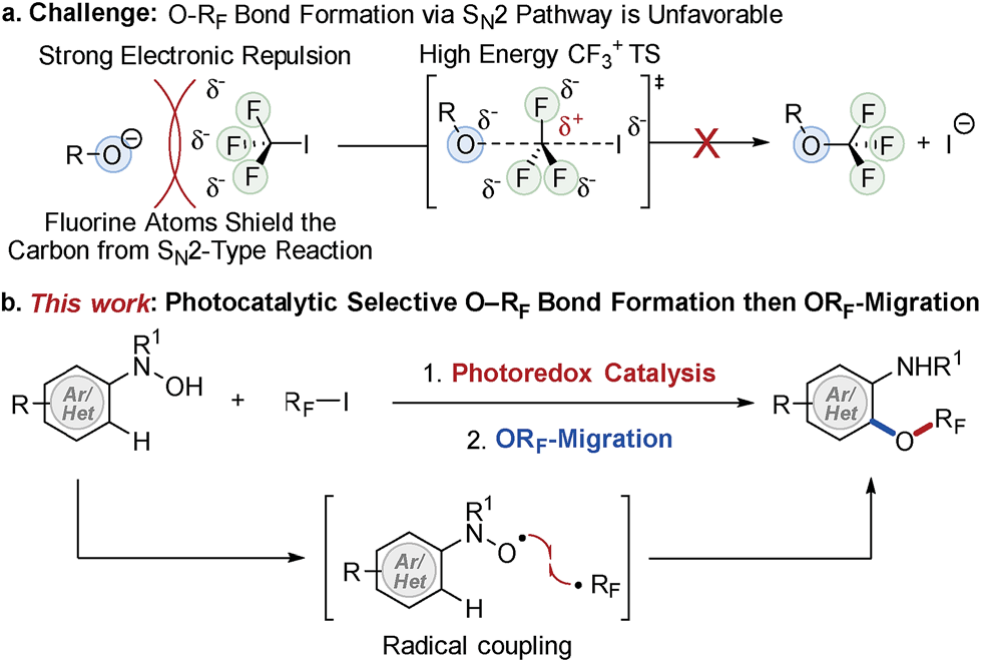
Photocatalytic radical coupling for the synthesis of perfluoroalkoxylated (hetero)arenes.

Molecules containing a perfluoroalkoxy group (OR_F_) have emerged as an important class of compounds in the fields of pharmaceutical, agrochemical, and materials science because incorporation of an OR_F_ group into organic compounds often improves thermal, chemical and metabolic stability, lipophilicity, and bioavailability of parent molecules.^1–10^ While much progress has been made for late stage fluorination,^11,12^ perfluoroalkylation,^13–15^ and perfluoroalkylthiolation^16–19^ of (hetero)arenes, the facile synthesis of perfluoroalkoxylated (hetero)aromatic compounds remains an unmet challenge in synthetic organic chemistry.^9,20–25^ Unlike their analogous alkoxy groups, formation of an O–R_F_ bond (*e.g.* R_F_ = CF_3_) *via* direct S_N_2 type displacement is unfavorable due to (i) strong electron repulsion between fluorine atoms and incoming nucleophiles and (ii) the formation of an energetically adverse CF_3_ carbocation transition state (TS) structure (Fig. 1a).^2,26^ Umemoto *et al.* addressed this issue with an elegant electrophilic O–R_F_ bond formation strategy *via* radical intermediates,^20^ yet the non-selective formation of *O*- and *C*-perfluoroalkylated products limited its synthetic utility. Although new strategies for the synthesis of perfluoroalkoxylated (hetero)arenes have emerged over the past few years,^23,27–32^ a general and mild catalytic process has yet to be developed. As a result, the full potential of perfluoroalkoxylated (hetero)aromatic compounds has not been fully exploited across a broad spectrum of technological applications.

To address this challenge, we recently developed trifluoromethoxylation reactions of aromatic compounds using *N*-(hetero)aryl-*N*-hydroxylamides and Togni reagents under mild reaction conditions.^33,34^ Our operationally simple and scalable protocols provide access to a diverse array of trifluoromethoxylated (hetero)aromatics with complex molecular architectures. Nevertheless, the high cost and multi-step synthesis of Togni reagents (*e.g.* Togni reagent I costs $55 980 mol^–1^)^35^ might hinder their synthetic application. Furthermore, preparation of other *O*-perfluoroalkylated analogues requires the use of unique hypervalent iodine(iii) perfluorinating reagents, which are commercially unavailable, synthetically-inaccessible, and thermally unstable. In order to develop a general method to access perfluoroalkoxylated (hetero)arenes, we turned our attention to R_F_–I reagents (R_F_ = perfluoroalkyl) that are commercially available and cost efficient (*e.g.* CF_3_I costs $83 mol^–1^).^35^ Based on our prior mechanistic studies,^36^ selective O–R_F_ bond formation is feasible if *N*-hydroxyl and R_F_ radicals are generated simultaneously.^37^ Although direct single electron transfer (SET) from *N*-(hetero)aryl-*N*-hydroxylamides to R_F_–I is kinetically and thermodynamically unfavorable, we hypothesize that such a SET process could be facilitated by using an appropriate photoredox catalyst.^15,38,39^ Herein, we describe our efforts to develop the first photocatalytic radical coupling reaction of *N*-(hetero)aryl-*N*-hydroxylamides with R_F_–I to form N–OR_F_ compounds, which then undergo OR_F_-migration to afford a wide variety of perfluoroalkoxylated (hetero)arenes (Fig. 1b).

## Results and discussion

To examine the feasibility of our hypothesis, we started our investigation using *N*-(*p-tert*-butylphenyl)-*N*-hydroxylamide (**1a**) and perfluoroisopropyl iodide (**2a**) as model substrates. Pleasingly, after exposure of **1a** (1.00 equiv.) and **2a** (8.00 equiv.) to visible light irradiation [3 W blue light-emitting diodes (LEDs)] in the presence of a ruthenium photoredox catalyst [Ru(bpy)_3_(PF_6_)_2_, (0.500 mol%)] and potassium carbonate (3.00 equiv.) in acetonitrile (0.100 M) at 23 °C for 12 hours, we obtained the desired product **3a** in 38% yield (Table 1, entry 1). Exploration of photoredox catalysts, solvents, bases, concentrations, reactant stoichiometry and catalyst loading did not improve the product yield (entries 2–5). A breakthrough in optimization came when we lowered the reaction temperature to 0 °C, at which an 80% yield of the desired product **3a** was obtained (entry 6). It is noteworthy that we did not observe addition of R_F_ radicals directly to arenes even though such a reaction has been developed under photoredox-catalyzed reaction conditions.^15,40^ Apparently, this is due to the persistent radical effect that coupling of O- and R_F_-radicals is more favorable than the addition of R_F_ radicals to arenes.^41,42^ Finally, control experiments showed that a photoredox catalyst, a base, light, and an oxygen-free atmosphere are critical for the success of the perfluoroalkylation reaction (entries 7–10).

**Table 1:** Optimization of the perfluoroalkoxylation reaction

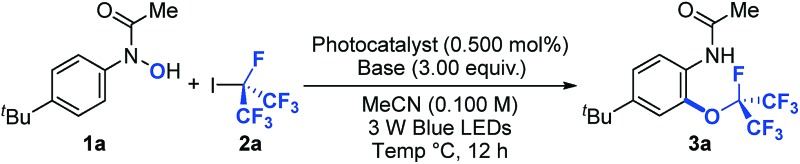				
Entry	Photocatalyst	Base	Temp (°C)	Yield^*a*^ (%)
1	Ru(bpy)_3_(PF_6_)_2_	K_2_CO_3_	23	38
2	Rhodamine 6-G	K_2_CO_3_	23	<5
3	*fac*-Ir(ppy)_3_	K_2_CO_3_	23	17
4	Ru(bpy)_3_(PF_6_)_2_	K_3_PO_4_	23	12
5	Ru(bpy)_3_(PF_6_)_2_	2,6-Lutidine	23	12
6	Ru(bpy)_3_(PF_6_)_2_	K_2_CO_3_	0	80
7	—	K_2_CO_3_	0	<5
8	Ru(bpy)_3_(PF_6_)_2_	—	0	<5
9	Ru(bpy)_3_(PF_6_)_2_	K_2_CO_3_	0	<5^*b*^
10	Ru(bpy)_3_(PF_6_)_2_	K_2_CO_3_	0	<5^*c*^

^*a*^Reaction conditions: **1a** (1.00 equiv.), **2a** (8.00 equiv.), photocatalyst (0.500 mol%) and base (3.00 equiv.) in MeCN (0.100 M) for 12 h. Yields were determined by ^19^F NMR using trifluorotoluene as the internal standard.

^*b*^No light.

^*c*^Exposed to air.

With the optimized reaction conditions in hand, we explored the scope of the perfluoroisopropylation reaction with respect to *N*-(hetero)aryl-*N*-hydroxylamides (**1a–1t**) (Table 2).^43^ The optimized reaction conditions were compatible with both aromatic and heteroaromatic hydroxylamides bearing a wide variety of functional groups and molecular scaffolds. For example, substrates with benzylic hydrogens, which are often prone to hydrogen atom abstraction in the presence of radical species, are tolerated (**3b–3e** and **3q–3s**). Presumably, the rate of O- and R_F_-radical coupling is faster than that of benzylic hydrogen atom abstraction. These results further demonstrate the chemoselectivity of our protocol. In addition, halogen functionalities (**3f–3i**, **3n**, **3o** and **3q**) remained intact after the reaction, providing easy handles for further synthetic elaborations. Substrates containing polyfluoromethyl ethers were also viable and afforded good yields of the desired products (**3j** and **3k**). Moreover, products derived from the heterocyclic *N*-hydroxylamides such as benzofuran (**3l**) and benzothiophene (**3m**) were formed smoothly with high levels of regioselectivity. Other functional groups such as esters (**3e** and **3m**), ketones (**3l** and **3s**), ethers (**3q–3t**), carbamates (**3e**), 1,2,4-oxadiazoles (**3n**), oxindoles (**3o**), pyrazoles (**3p**), pyridines (**3p–3t**) and ketals (**3t**) were susceptible to OR_F_ addition as well. Importantly, more complex *N*-pyridinyl-*N*-hydroxylamides derived from estrone and diacetone-d-glucose could be effectively converted to their perfluoroisopropylated analogs (**3s** and **3t**), demonstrating that this method can be used in the preparation of pharmaceutically relevant compounds. Notably, none of the perfluoroisopropylated arenes and pyridines reported herein have been prepared prior to this study.

**Table 2:** Selected examples of the perfluoroisopropylation of arenes and heteroarenes^*a*^

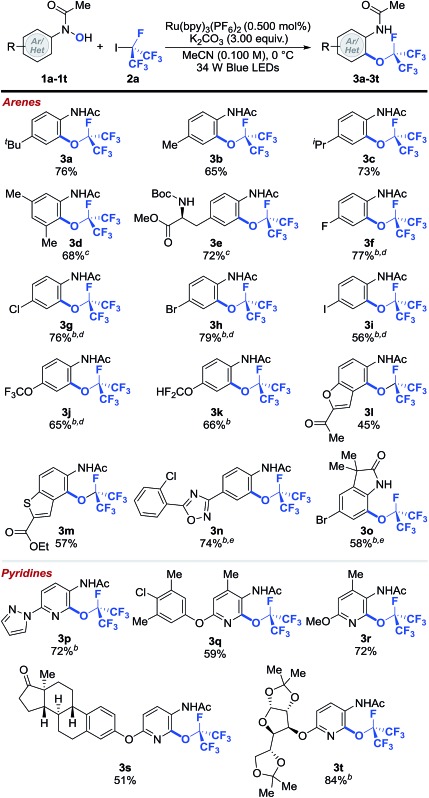

^*a*^Reaction conditions: **1** (1.00 equiv.), **2a** (8.00 equiv.), Ru(bpy)_3_(PF_6_) (0.500 mol%), K_2_CO_3_ (3.00 equiv.) in MeCN (0.100 M) at 0 °C. Cited yields are for isolated material.

^*b*^–40 °C.

^*c*^Following perfluoroalkylation, the reaction was heated to 40 °C.

^*d*^Following *O*-perfluoroalkylation, the reaction was filtered, concentrated and the residue was dissolved in MeCN and heated to 80 °C.

^*e*^Following *O*-perfluoroalkylation, the reaction was filtered, concentrated and the residue was dissolved in MeNO_2_ and heated to 120 °C. See ESI for further experimental details.†

Trifluoromethoxy aryl ethers (Ar–OCF_3_) are constituents of several pharmaceutically active compounds, agrochemicals, and functional materials.^3,5,6,8,9^ As a result, significant effort has recently been directed towards uncovering general and practical protocols for their preparation,^6,24,25^ yet methods that use commercially available CF_3_I for their preparation have not been developed. We were pleased to see that our photocatalytic protocol can also be used for the synthesis of trifluoromethoxylated arenes (**4a–4d**) from CF_3_I (Table 3). In general, *O*-trifluoromethylation required a longer reaction time (48 h *vs.* 12–24 h for heptafluoroalkoxylation), possibly due to the lower reduction potential of CF_3_I (*E*red1/2 = –1.52 V *vs.* SCE)^44^ in comparison with (CF_3_)_2_CFI (*E*red1/2 = –0.66 V *vs.* SCE),^44^ which required an over-potential of 0.19 V for the reduction of CF_3_I to generate the CF_3_ radical using Ru(bpy)_3_^+^ (*E*red1/2 = –1.33 V *vs.* SCE).^45^ In addition, other perfluoroalkyl iodides such as 1,1,1,2,2,3,4,4,4-nonafluoro-3-iodobutane and *n*-perfluorohexyliodide coupled smoothly to afford the desired products (**4e** and **4f**) in synthetically useful yields. Importantly, our reaction is applicable to polyfluoroalkyl iodides such as 1-chloro-2-iodo-tetrafluoroethane and 1-bromo-2-iodotetrafluoroethane, albeit that **4h** was obtained in a lower yield. This may be due to the instability of the 1-bromotetrafluoroethoxide species generated during the OR_F_-migration process. It is worth noting that the anilide moiety of the products could serve as a versatile handle for further synthetic functionalizations.^34^

**Table 3:** Selected examples of the polyfluoroalkoxylation of arenes^*a*^

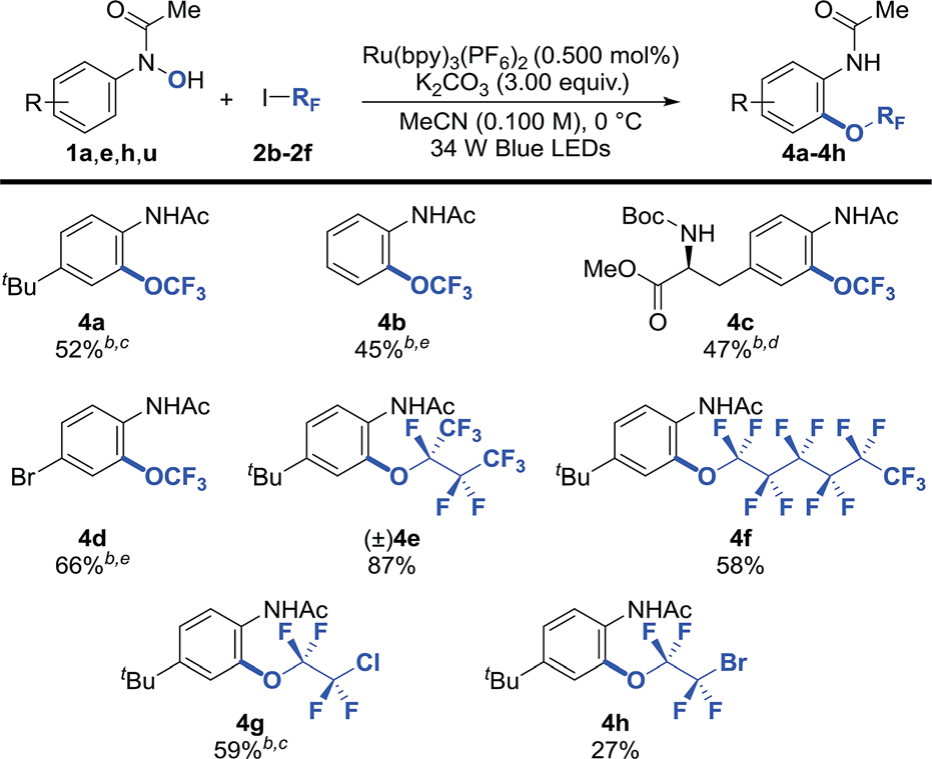

^*a*^Reaction conditions: **1** (1.00 equiv.), **2** (8.00 equiv.), Ru(bpy)_3_(PF_6_) (0.500 mol%), K_2_CO_3_ (3.00 equiv.) in MeCN (0.100 M) at 0 °C. Cited yields are for isolated material.

^*b*^–40 °C.

^*c*^Following *O*-perfluoroalkylation, the reaction was heated to 40 °C.

^*d*^Following *O*-perfluoroalkylation, the reaction was filtered, concentrated and the residue was dissolved in MeCN and heated to 40 °C.

^*e*^Following *O*-perfluoroalkylation, the reaction was filtered, concentrated and the residue was dissolved in MeCN and heated to 80 °C. See the ESI for further experimental details.†

**Fig. 2:**
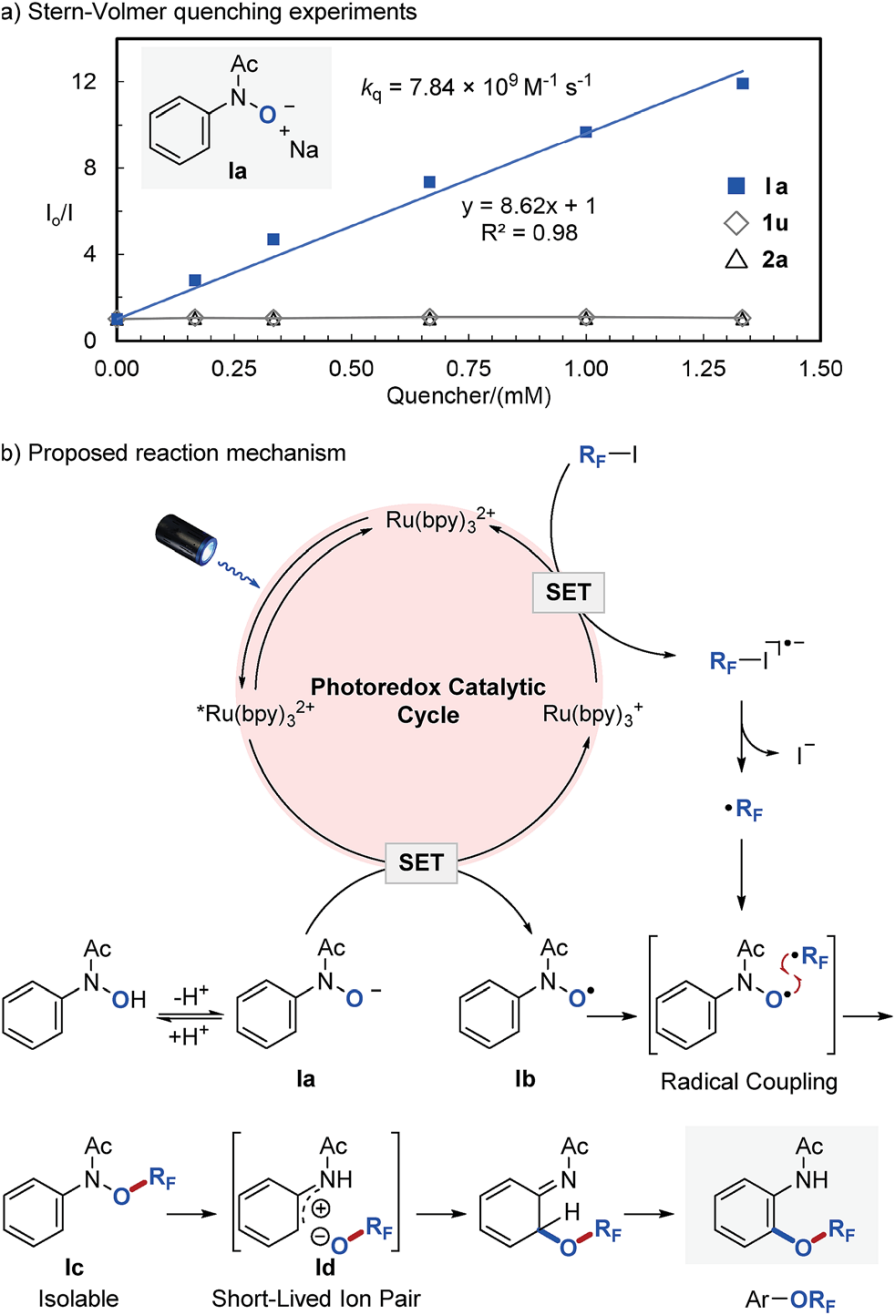
a) Stern–Volmer quenching experiments. (b) The proposed reaction mechanism.

In order to get an insight into the mechanism of the photocatalytic reaction, we performed a series of Stern–Volmer quenching experiments (Fig. 2a). While deprotonated *N*-phenyl-*N*-hydroxylamide (**Ia**, *E*red1/2 = 0.62 V *vs.* SCE)^46^ efficiently quenched *Ru(bpy)_3_^2+^ in MeCN with a quenching constant of *k*_q_ = 7.84 × 10^9^ M^–1^ s^–1^, *N*-phenyl-*N*-hydroxylamide (**1u**) and perfluoroisopropyl iodide (**2a**) quenched the photoexcited photocatalyst (*Ru(bpy)_3_^2+^) only to a minor extent. We also observed that the OR_F_ migration is slower with more electron deficient aromatics, which is consistent with our previous observations and suggests an ionic OR_F_-migration pathway.^36^ Based on these results, a detailed description of our proposed photocatalytic cycle for selective O–R_F_ bond formation and the consequent OR_F_-migration is outlined in Fig. 2b. Irradiation of Ru(bpy)_3_^2+^ with visible light produces a long-lived (1.10 μs) photoexcited state, *Ru(bpy)_3_^2+^,^45^ which engages in a SET with **Ia** to give *N*-hydroxyl radical (**Ib**) and a strong reductant Ru(bpy)_3_^+^ (*E*red1/2 = –1.33 V *vs.* SCE).^45^ A single electron reduction of perfluoroalkyl iodide (R_F_I) with Ru(bpy)_3_^+^ forms a perfluoroalkyl radical (˙R_F_) and regenerates Ru(bpy)_3_^2+^. Subsequent radical–radical coupling between **Ib** and ˙R_F_ affords *O*-perfluoroalkylated *N*-phenyl-*N*-hydroxylamide **Ic**, which undergoes heterolytic N–OR_F_ bond cleavage^47,48^ followed by recombination of the resulting short-lived ion pair (**Id**) and then tautomerization to yield the final perfluoroalkoxylated arene product.^36^

## Conclusions

In conclusion, we have developed the first photocatalytic protocol for the synthesis of structurally diverse perfluoroalkoxylated (hetero)arenes. The key to the success of our approach is the ability to concomitantly generate persistent and transient radicals under photoredox-catalyzed reaction conditions, which provide direct access to the challenging O–R_F_ bond formation. Our approach is one of the mildest and most general perfluoroalkoxylations of (hetero)arenes reported to date.^9,20–25^ It features a broad substrate scope and high functional group compatibility. In addition, the use of commercially available R_F_I reagents and the excellent chemoselectivity of this reaction represents a considerable advance in the construction of the O–R_F_ bond and should have a significant impact on the approach towards the synthesis of perfluoroalkoxylated aromatic building blocks. The success of this method not only provides access to unexplored chemical spaces to aid the discovery and development of novel drugs, agrochemicals, and functional materials, but also establishes a solid framework for further development of the photocatalytic radical coupling strategy using *N*-(hetero)aryl-*N*-hydroxylamides.
