# Supplementary material for: Selective C–O bond formation via a photocatalytic radical coupling strategy: access to perfluoroalkoxylated (ORF) arenes and heteroarenes
Source: Chem Sci. 2017 Jun 5;8(9):6066–70. doi: 10.1039/c7sc01684k (PMC5625593; doi:10.1039/c7sc01684k)

Electronic Supplementary Material (ESI) for Chemical Science.
This journal is © The Royal Society of Chemistry 2017

Supporting Information

Selective C–O Bond Formation via Photocatalytic Radical Coupling Strategy: Access to Perfluoroalkoxylated (OR_F) Arenes and Heteroarenes

Johnny W. Lee, Dominique N. Spiegowski, and Ming-Yu Ngai*

*ming-yu.ngai@stonybrook.edu

*Department of Chemistry, Stony Brook University,
Stony Brook, New York 11794-3400*

*Institute of Chemical Biology and Drug Discovery, Stony Brook University,
Stony Brook, New York 11794-3400*

Table of Contents

General Information 3

Optimization for Substrates with Electron Deficient *N*-Aromatic Ring 4

Cryogenic Reaction Set-Up 7

Reaction Set-Up at 0 °C 7

Reaction Set-Up at -40 °C 7

Experimental Data 8

N-(4-(*tert*-Butyl)phenyl)-*N*-hydroxyacetamide (1a) 8

N-Hydroxy-*N*-(4-isopropylphenyl)acetamide (1c) 9

N-(3,5-Dimethylphenyl)-*N*-hydroxyacetamide (1d) 9

Methyl (*S*)-2-((*tert*-butoxycarbonyl)amino)-3-(4-(*N*-hydroxyacetamido)phenyl)propanoate (1e) 10

N-Hydroxy-*N*-(4-(trifluoromethoxy)phenyl)acetamide (1j) 11

N-(4-(Difluoromethoxy)phenyl)-*N*-hydroxyacetamide (1k) 11

N-(2-Acetylbenzofuran-5-yl)-*N*-hydroxyacetamide (1l) 12

Ethyl 5-(*N*-hydroxyacetamido)benzo[*β*]thiophene-2-carboxylate (1m) 13

N-(4-(5-(2-Chlorophenyl)-1,2,4-oxadiazol-3-yl)phenyl)-*N*-hydroxyacetamide (1n) 13

5-(2-Chlorophenyl)-3-(4-nitrophenyl)-1,2,4-oxadiazole (S1) 14

N-(6-(4-Chloro-3,5-dimethylphenoxy)-4-methylpyridin-3-yl)-*N*-hydroxyacetamide (1q) 15

2-(((3*αR*,5*R*,6*S*,6*αR*)-5-((*R*)-2,2-dimethyl-1,3-dioxolan-4-yl)-2,2-dimethyltetrahydrofuro[2,3-*d*][1,3]dioxol-6-yl)oxy)-5-nitropyridine (S2) 15

N-(6-(((3*αR*,5*R*,6*S*,6*αR*)-5-((*R*)-2,2-Dimethyl-1,3-dioxolan-4-yl)-2,2-dimethyltetrahydrofuro[2,3-*d*][1,3]dioxol-6-yl)oxy)pyridin-3-yl)-*N*-hydroxyacetamide (1t) 16

*N-(*4-(*tert*-Butyl)-2-((perfluoropropan-2-yl)oxy)phenyl)acetamide (3a) 17

*N-(*4-Methyl-2-((perfluoropropan-2-yl)oxy)phenyl)acetamide (3b) 17

*N-(*4-Isopropyl-2-((perfluoropropan-2-yl)oxy)phenyl)acetamide (3c) 18

*N-(*3,5-Dimethyl-2-((perfluoropropan-2-yl)oxy)phenyl)acetamide (3d) 19

Methyl-(*S*)-3-(4-acetamido-3-((perfluoropropan-2-yl)oxy)phenyl)-2-((*tert*-butoxycarbonyl)amino)propanoate (3e) 20

*N-(*4-Fluoro-2-((perfluoropropan-2-yl)oxy)phenyl)acetamide (3f) 21

*N-(*4-Chloro-2-((perfluoropropan-2-yl)oxy)phenyl)acetamide (3g) 22

*N-(*4-Bromo-2-((perfluoropropan-2-yl)oxy)phenyl)acetamide (3h) 23

N-(4-Iodo-2-((perfluoropropan-2-yl)oxy)phenyl)acetamide (3i) 24

N-(2-((Perfluoropropan-2-yl)oxy)-4-(trifluoromethoxy)phenyl)acetamide (3j) 24

N-(4-(Difluoromethoxy)-2-((perfluoropropan-2-yl)oxy)phenyl)acetamide (3k) 25

N-(2-Acetyl-4-((perfluoropropan-2-yl)oxy)benzofuran-5-yl)acetamide (3l) 26

Ethyl 5-acetamido-4-((perfluoropropan-2-yl)oxy)benzo[*β*]thiophene-2-carboxylate (3m) 27

N-(4-(5-(2-Chlorophenyl)-1,2,4-oxadiazol-3-yl)-2-((perfluoropropan-2-yl)oxy)phenyl)acetamide (3n) 28

5-Bromo-3,3-dimethyl-7-((perfluoropropan-2-yl)oxy)indolin-2-one (3o) 29

N-(2-((Perfluoropropan-2-yl)oxy)-6-(1*H*-pyrazol-1-yl)pyridin-3-yl)acetamide (3p) 29

N-(6-(4-Chloro-3,5-dimethylphenoxy)-4-methyl-2-((perfluoropropan-2-yl)oxy)pyridin-3-yl)acetamide (3q) 30

N-(6-Methoxy-4-methyl-2-((perfluoropropan-2-yl)oxy)pyridin-3-yl)acetamide (3r) 31

N-(6-(((8*R*,9*S*,13*S*,14*S*)-13-Methyl-17-oxo-7,8,9,11,12,13,14,15,16,17-decahydro-6*H*-cyclopenta[*α*]phenanthren-3-yl)oxy)-2-((perfluoropropan-2-yl)oxy)pyridin-3-yl)acetamide (3s) 32

N-(6-(((3*αR*,5*R*,6*S*,6*αR*)-5-((*R*)-2,2-dimethyl-1,3-dioxolan-4-yl)-2,2-dimethyltetrahydrofuro[2,3-*d*][1,3]dioxol-6-yl)oxy)-2-((perfluoropropan-2-yl)oxy)pyridin-3-yl)acetamide (3t) 33

N-(4-(*tert*-Butyl)-2-(trifluoromethoxy)phenyl)acetamide (4a) 34

N-(2-(Trifluoromethoxy)phenyl)acetamide (4b) 34

Methyl (*S*)-3-(4-acetamido-3-(trifluoromethoxy)phenyl)-2-((*tert*-butoxycarbonyl)amino)propanoate (4c) 35

N-(4-Bromo-2-(trifluoromethoxy)phenyl)acetamide (4d) 36

(±)-*N*-(4-(*tert*-Butyl)-2-((perfluorobutan-2-yl)oxy)phenyl)acetamide (4e) 37

N-(4-(*tert*-Butyl)-2-((perfluorohexyl)oxy)phenyl)acetamide (4f) 38

N-(4-(*tert*-Butyl)-2-(2-chloro-1,1,2,2-tetrafluoroethoxy)phenyl)acetamide (4g) 38

N-(2-(2-Bromo-1,1,2,2-tetrafluoroethoxy)-4-(*tert*-butyl)phenyl)acetamide (4h) 39

Mechanistic Studies 41

Stern–Volmer Luminescence Quenching 41

Spectroscopic Data 43

General Information

All air- and moisture-insensitive reactions were carried out under an ambient atmosphere, magnetically stirred, and monitored by thin layer chromatography (TLC) using Agela Technologies TLC plates pre-coated with 250 μm thickness silica gel 60 F254 plates and visualized by fluorescence quenching under UV light. Flash chromatography was performed on SiliaFlash® Silica Gel 40-63μm 60Å particle size using a forced flow of eluent at 0.3–0.5 bar pressure.[1] Preparative TLC was performed on Uniplate® UV254 (20 x 20 cm) with 1000 μm thickness and visualized fluorescence quenching under UV light.

All air and moisture-sensitive manipulations were performed using oven-dried glassware, including standard Schlenk and glovebox techniques under an atmosphere of nitrogen. All reaction vials were capped using green caps with F-217 PTFE liners. THF was distilled from deep purple sodium benzophenone ketyl. Acetonitrile was dried over CaH_2 and distilled. Dried acetonitrile was degassed via three freeze-pump-thaw cycles. Polyfluoroalkyl iodides were purified by passing through a small plug of basic alumina. Then the polyfluoroalkyl iodides were degassed via three freeze-pump-thaw cycles. All other chemicals were used as received.

All deuterated solvents were purchased from Cambridge Isotope Laboratories. NMR spectra were recorded on either a Bruker Ascend 700 spectrometer operating at 700 MHz for ^{1}H acquisitions and 175 MHz for ^{13}C acquisitions, a Bruker 500 Advance spectrometer operating at 500 MHz, 125 MHz, and 470 MHz for ^{1}H, ^{13}C, and ^{19}F acquisitions, respectively, a Bruker 400 Nanobay spectrometer operating at 400 MHz, 100 MHz, and 376 MHz for ^{1}H, ^{13}C, and ^{19}F acquisitions, respectively. Chemical shifts were referenced to the residual proton solvent peaks [^{1}H: $CDCl_3$, δ 7.26; $(CD_3)_2SO$, δ 2.50], solvent ^{13}C signals [$CDCl_3$, δ 77.16; $(CD_3)_2SO$, δ 39.52],[2] dissolved or external neat $PhCF_3$ (^{19}F, δ –63.3 relative to $CFCl_3$).[3] Signals are listed in ppm, and multiplicity identified as s = singlet, br = broad, d = doublet, t = triplet, q = quartet, sep = septet, dd = doublet of doublet, tt = triplet of triplet, qd = quartet of doublet, and m = multiplet; coupling constants in Hz; integration.

Concentration was performed under reduced pressure using a rotary evaporator at 25–30 ºC at appropriate pressure. Purified compounds were further dried under high vacuum (0.01–0.05 Torr). Yields refer to purified and spectroscopically pure compounds.

[1] Still, W. C.; Kahn, M.; Mitra, A. *J. Org. Chem.* **1978**, *43*, 2925.
[2] Fulmer, G. R.; Miller, A. J. M.; Sherden, N. H.; et al.; *Organometallics.* **2010**, *29*, 2176.
[3] Wang, X.; Xu, Y.; Mo, F.; Ji, G.; Qiu, D.; Feng, J.; et al.; *J. Am. Chem. Soc.* **2013**, *135*, 10330.

Optimization for Substrates with Electron Deficient *N*-Aromatic Ring

For substrates with electron deficient *N*-aromatic ring where heating is needed for the OR_F-migration step, further reaction optimizations were performed (Table S1 and Table S2). We found that removal of inorganic salts by filteration through a pad of Celite before heating the intermediate **1h'** in MeCN afforded a higher yield (Table S2, entry 4).

Table S1. Selected optimization experiments of *O*-perfluoroisopropylation of *N*-(4-bromophenyl)-*N*-hydroxyacetamide.

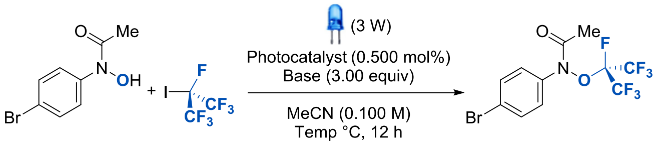

1h (1.00 equiv) **2a** (8.00 equiv) **1h'**

Entry	Photocatalyst	Base	Temperature (°C)	Yield (%)[a]
1	Rh-6G	K_2CO_3	23	0
2	*fac*-Ir(ppy)$_3$	K_2CO_3	23	0
3	Ru(bpy)$_3$(PF$_6$)$_2$	K_2CO_3	23	15
4	Ru(bpy)$_3$(PF$_6$)$_2$	K_3PO_4	23	0
5	Ru(bpy)$_3$(PF$_6$)$_2$	2,6-Lutidine	23	0
6	Ru(bpy)$_3$(PF$_6$)$_2$	K_2CO_3	0	37
7	Ru(bpy)$_3$(PF$_6$)$_2$	K_2CO_3	-20	73
8	Ru(bpy)$_3$(PF$_6$)$_2$	K_2CO_3	-40	96

[a] Yields were determined by ^{19}F NMR using trifluorotoluene as the internal standard.

In a glovebox, to an oven-dried 4 mL screw cap vial was added *N*-(4-bromophenyl)-*N*-hydroxyacetamide (6.90 mg, 30.0 μmol, 1.00 equiv), anhydrous base (3.00 equiv), photoredox catalyst (0.500 mol%), and MeCN (0.300 mL, 0.100 M). To this suspension was added perfluoroisopropyl iodide (34.2 μL, 0.480 mmol, 8.00 equiv) and a magnetic stir bar. Next, the vial was capped and taken out of the glovebox. The reaction mixture was stirred at 23 °C (or lower temperature using a cryogenic ethanol bath) and irradiated with a 3 W blue LED strip for 12 h. After that time, to the reaction mixture was added trifluorotoluene as the internal standard (3.68 μL, 30.0 μmol, 1.00 equiv) (*Note*: if the reaction was performed at lower temperature, the reaction vial was first removed from the bath and warmed up to 23 °C before addition of the internal standard). The yield of the reaction was determined by ^{19}F NMR using trifluorotoluene as the internal standard.

Table S2. Selected optimization experiments of the two-step process.

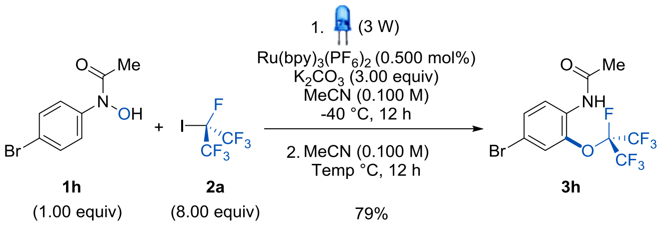

Entry	Temperture for Step 2 (°C)	Conditions After Step 1	Yield (%)[a]
1	40	No Filtering of Reaction	38
2	60	No Filtering of Reaction	48
3	80	No Filtering of Reaction	52
4	80	Filtering Reaction	79

[a] Yields were over two steps and determined by ^{19}F NMR using trifluorotoluene as the internal standard.

Without filtration:

In a glovebox, to an oven-dried 4 mL screw cap vial was added *N*-(4-bromophenyl)-*N*-hydroxyacetamide (6.90 mg, 30.0 μmol, 1.00 equiv),, anhydrous K_2CO_3 (12.4 mg, 90.0 μmol, 3.00 equiv), $Ru(bpy)_3(PF_6)_2$ (0.130 mg, 0.150 μmol, 0.500 mol%), and MeCN (0.300 mL, 0.100 M). To this suspension was added perfluoroisopropyl iodide (34.2 μL, 0.480 mmol, 8.00 equiv) and a magnetic stir bar. Next, the vial was capped, taken out of the glovebox, and cooled to -40 °C in a cryogenic ethanol bath. The reaction mixture was stirred at -40 °C and irradiated with a 3 W blue LED lamp for 12 h. After that time, the reaction vial was removed from the bath and heated at an indicated temperature for 12 h. After that period, the reaction mixture was cooled to room temperature and trifluorotoluene (3.68 μL, 30.0 μmol, 1.00 equiv) was added as the internal standard. The yield of the reaction was determined by ^{19}F NMR using trifluorotoluene as the internal standard.

With filtration:

In a glovebox, to an oven-dried 4 mL screw cap vial was added *N*-(4-bromophenyl)-*N*-hydroxyacetamide (6.90 mg, 30.0 μmol, 1.00 equiv),, anhydrous K_2CO_3 (12.4 mg, 90.0 μmol, 3.00 equiv), $Ru(bpy)_3(PF_6)_2$ (0.130 mg, 0.150 μmol, 0.500 mol%), and MeCN (0.300 mL, 0.100 M). To this suspension was added perfluoroisopropyl iodide (34.2 μL, 0.480 mmol, 8.00 equiv) and a magnetic stir bar. Next, the vial was capped, taken out of the glovebox, and cooled to -40 °C in a cryogenic ethanol bath. The reaction mixture was stirred at -40 °C and irradiated with a 3 W blue LED lamp for 12 h. After that time, the reaction vial was removed from the bath and warmed up to 23 °C. The reaction mixture was passed through a plug of Celite to a 4 mL vial. The Celite was then washed with EtOAc (3 x 0.500 mL) and the combined filtrate in the vial was concentrated *in vacuo*. Under ambient atmosphere the residue in the vial was dissolved in MeCN (0.300 mL, 0.100 M) and a stir bar was added. The vial was capped and the reaction mixture was stirred at 80 °C for 12 h. After that period, the reaction mixture was cooled to room temperature and

trifluorotoluene (3.68 μL, 30.0 μmol, 1.00 equiv) was added as the internal standard. The yield of the reaction was determined by ^{19}F NMR using trifluorotoluene as the internal standard.

Cryogenic Reaction Set-Up

Note: Although the blue LED lamp (Kessil KSH150B LED Grow Light) could be simply held above the cryogenic ethanol bath, it was deemed safer to place it perpendicular to the sidewall of the cryogenic bath. In addition, it allowed the light source to be in closer proximity to the reaction vial (Figure S1).

Reaction Set-Up at 0 °C

A 4 mL or 20 mL capped vial was placed in cryogenic ethanol bath at 0 °C. Then a 34 W blue LED lamp was placed perpendicular to the sidewall of the cryogenic bath. The distance between the blue LED lamp and the sidewall of the cryogenic bath was 2.00 cm (Figure S1).

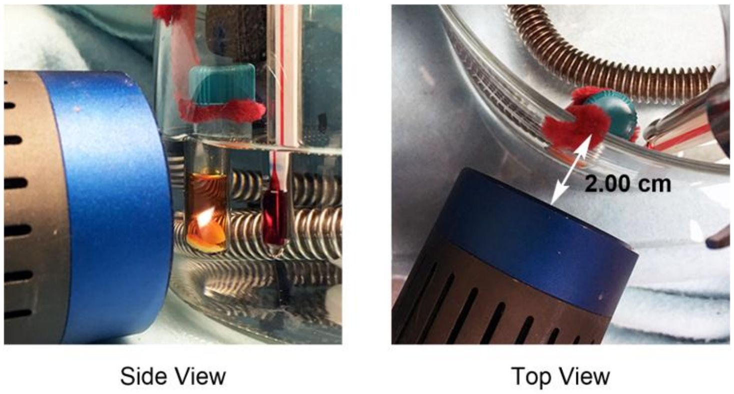

Figure S1. Schematic representation of the reaction setup at 0 °C with a side view of the setup (left) and a top view (right).

Reaction Set-Up at -40 °C

A 4 mL or 20 mL capped vial was placed in cryogenic ethanol bath at –40 °C. Then a square piece of glass (5.00 x 5.00 cm) was taped to the front of the blue LED lamp. Subsequently, the blue LED lamp was pressed against the sidewall of the bath. The piece of glass between the LED lamp and the cryogenic bath was necessary in order to prevent the formation of ice on the sidewall of the bath (Figure S2).

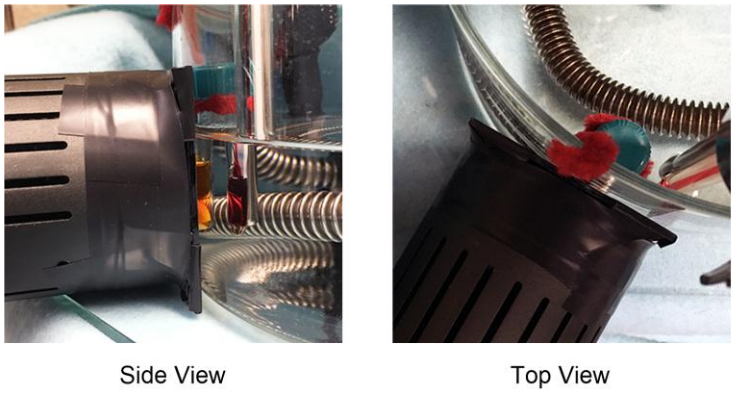

Figure S2 Schematic representation of the reaction setup at -40 °C with a side view of the setup (left) and a top view (right).

Experimental Data

Note: Compounds **1b**,[4] **1c**–**e**,[4] **1o**,[5] **1p**,[6] and **1s**[6] were synthesized according to literature reports. The R_f of compounds **3a**–**e** and **4a**–**h** is reported in both MeOH:DCM and EtOAc:Hexanes. In EtOAc:Hexanes several *N*-(hetero)aryl-*N*-hydroxylamides have identical R_f with a side product, *N*-(hetero)arylacetamides, making TLC analysis of the reaction mixture problematic. TLC analysis of the reaction mixture in MeOH:DCM, however, resolves these two spots.

N-(4-(*tert*-Butyl)phenyl)-*N*-hydroxyacetamide (1a)

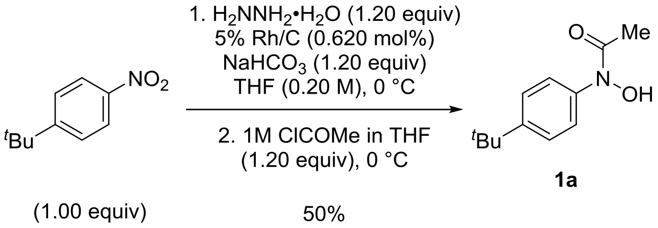

Under nitrogen atmosphere, a suspension of 1-(*tert*-butyl)-4-nitrobenzene (3.41 g, 19.0 mmol, 1.00 equiv), 5% Rh/C (243 mg, 0.118 mmol, 0.620 mol%), and $NaHCO_3$ (1.92 g, 22.8 mmol, 1.20 equiv) in THF (0.200 M, 95.0 mL) was cooled to 0 °C in an ice bath. Hydrazine monohydrate (1.12 mL, 22.8 mmol, 1.20 equiv) was then added dropwise and the reaction mixture was stirred vigorously with a stir bar at 0 °C for 1 h. While the reaction flask was still in the ice bath, a solution of acetyl chloride (1.62 mL, 22.8 mol, 1.20 equiv) in THF (0.100 M, 22.8 mL) was added dropwise and the resulting mixture was stirred at 0 °C for 2 h and then warmed up to 23 °C. After that time, the reaction mixture was concentrated in *vacuo* and the residue was purified by flash column chromatography on silica gel eluting with EtOAc:Hexanes [1:1 (v/v)] to afford the title compound as a light orange solid (1.95 g, 9.41 mmol, 50% yield).

R_f = 0.33 EtOAc:Hexanes [1:1 (v/v)].

^{1}H NMR (700 MHz, $(CD_3)_2SO$, 25 ºC, δ): 10.55 (br. s, 1H), 7.51 (d, *J* = 7.7 Hz, 2H), 7.38 (d, *J* = 7.7 Hz, 2H), 2.19 (s, 3H), 1.28 (s, 9H).

^{13}C NMR (175 MHz, $(CD_3)_2SO$, 25 ºC, δ): 169.49, 146.83, 139.02, 124.87, 119.94, 33.94, 30.97, 22.15.

HRMS (ESI-TOF) (*m/z*): calcd for $C_{12}H_{18}NO_2$ ($[M + H]^+$): 208.1332, found: 208.1332.

[4] Hojczyk, K. N.; Feng, P.; Zhan, C.; Ngai, M.-Y., *Angew. Chem. Int. Ed.* **2014,** *53*, 14559.
[5] Ji, W.; Liu, Y. A.; Liao, X., *Angew. Chem. Int. Ed.* **2016,** *55*, 13286.
[6] Feng, P. J.; Lee, K. N.; Lee, J. W.; Zhan, C. B.; Ngai, M. Y., *Chem. Sci.* **2016,** *7*, 424.

N-Hydroxy-N-(4-isopropylphenyl)acetamide (1c)

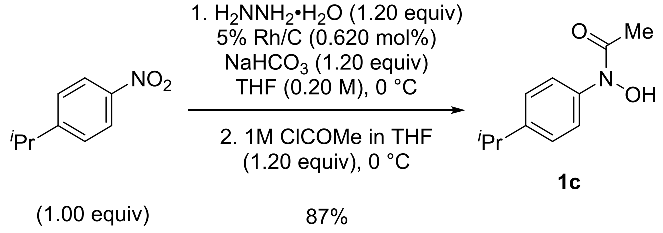

Under nitrogen atmosphere, a suspension of 1-isopropyl-4-nitrobenzene (1.05 mL, 6.93 mmol, 1.00 equiv), 5% Rh/C (88.5 mg, 0.0430 mmol, 0.620 mol%), and $NaHCO_3$ (0.700 g, 8.32 mmol, 1.20 equiv) in THF (0.200 M, 34.6 mL) was cooled to 0 °C in an ice bath. Hydrazine monohydrate (0.403 mL, 8.32 mmol, 1.20 equiv) was then added dropwise and the reaction mixture was stirred vigorously with a stir bar at 0 °C for 1 h. While the reaction flask was still in the ice bath, a solution of acetyl chloride (0.591 mL, 8.32 mmol, 1.20 equiv) in THF (0.100 M, 8.32 mL) was added dropwise and the resulting mixture was stirred at 0 °C for 2 h and then warmed up to 23 °C. After that time, the reaction mixture was concentrated in *vacuo* and the residue was purified by flash column chromatography on silica gel eluting with EtOAc:Hexanes [1:4 (v/v)] to afford the title compound as a light orange solid (1.16 g, 6.02 mmol, 87% yield).

R_f = 0.40 EtOAc:Hexanes [1:1 (v/v)].

^{1}H NMR (700 MHz, $(CD_3)_2SO$, 25 ºC, δ): 10.53 (s, 1H), 7.49 (d, J = 8.4 Hz, 2H), 7.21 (d, J = 8.4 Hz, 2H), 2.83 (sep, J = 6.9 Hz, 1H), 2.17 (s, 3H), 1.18 (d, J = 6.9 Hz, 6H).

^{13}C NMR (175 MHz, $(CD_3)_2SO$, 25 ºC, δ): 169.39, 144.51, 139.26, 125.86, 120.24, 32.64, 23.62, 22.03.

HRMS (ESI-TOF) (*m/z*): calcd for $C_{11}H_{16}NO_2$ ($[M + H]^+$): 194.1176, found: 194.1178.

N-(3,5-Dimethylphenyl)-N-hydroxyacetamide (1d)

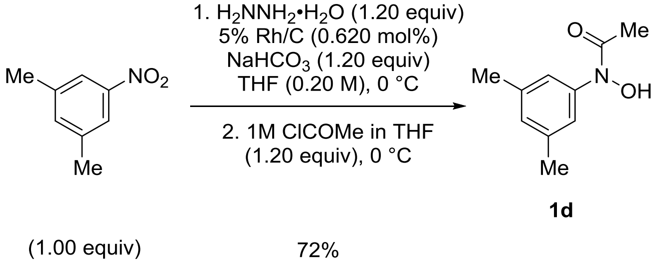

Under nitrogen atmosphere, a suspension of 1,3-dimethyl-5-nitrobenzene (1.10 g, 7.29 mmol, 1.00 equiv), 5% Rh/C (92.0 mg, 0.0452 mmol, 0.620 mol%), and $NaHCO_3$ (0.726 g, 8.75 mmol, 1.20 equiv) in THF (0.200 M, 36.5 mL) was cooled to 0 °C in an ice bath. Hydrazine monohydrate (0.423 mL, 8.75 mmol, 1.20 equiv) was then added dropwise and the reaction mixture was stirred vigorously with a stir bar at 0 °C for 1 h. While the reaction flask was still in the ice bath, a solution of acetyl chloride (0.622 mL, 8.75 mol, 1.20 equiv) in THF (0.100 M, 8.75 mL) was added dropwise and the resulting mixture was stirred at 0 °C for 2 h and then warmed up to 23 °C. After that time, the reaction mixture was concentrated in vacuo and the

residue was purified by flash column chromatography on silica gel eluting with EtOAc:Hexanes [1:1 (v/v)] to afford the title compound as a light orange solid (0.939 g, 5.24 mmol, 72% yield).

R*f* = 0.30 EtOAc:Hexanes [1:1 (v/v)].

^{1}H NMR (700 MHz, $(CD_3)_2SO$, 25 ºC, δ): 10.50 (s, 1H), 7.27 (s, 2H), 6.78 (s, 1H), 2.26 (s, 6H), 2.19 (s, 3H).

^{13}C NMR (175 MHz, $(CD_3)_2SO$, 25 ºC, δ): 169.67, 141.65, 137.36, 126.07, 118.01, 22.48, 21.03.

HRMS (ESI-TOF) (*m/z*): calcd for $C_{10}H_{14}NO_2$ ([M + H]$^+$): 180.1019, found: 180.1014.

Methyl (*S*)-2-((*tert*-butoxycarbonyl)amino)-3-(4-(*N*-hydroxyacetamido)phenyl)propanoate (1e)

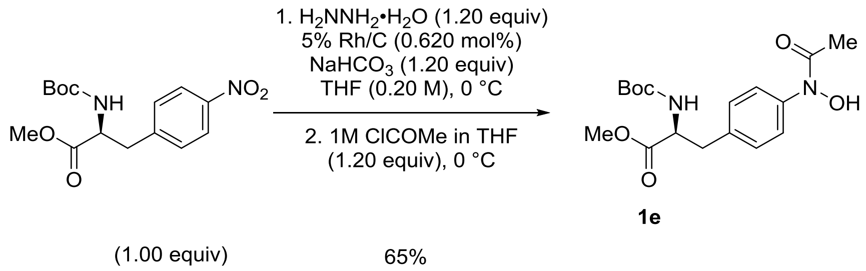

Under nitrogen atmosphere, a suspension of Methyl (*S*)-2-((*tert*-butoxycarbonyl)amino)-3-(4-nitrophenyl)propanoate (0.870 g, 2.68 mmol, 1.00 equiv), 5% Rh/C (34.2 mg, 0.0166 mmol, 0.620 mol%), and $NaHCO_3$ (0.271 g, 3.22 mmol, 1.20 equiv) in THF (0.200 M, 13.4 mL) was cooled to 0 °C in an ice bath. Hydrazine monohydrate (0.229 mL, 3.22 mmol, 1.20 equiv) was then added dropwise and the reaction mixture was stirred vigorously with a stir bar at 0 °C for 1 h. While the reaction flask was still in the ice bath, a solution of acetyl chloride (0.230 mL, 3.22 mmol, 1.20 equiv) in THF (0.100 M, 3.22 mL) was added dropwise and the resulting mixture was stirred at 0 °C for 2 h and then warmed up to 23 °C. After that time, the reaction mixture was concentrated in *vacuo* and the residue was purified by flash column chromatography on silica gel eluting with EtOAc:Hexanes [1:1 (v/v)] to afford the title compound as a beige solid (0.620 g, 1.74 mmol, 65% yield).

R*f* = 0.14 EtOAc:Hexanes [1:4 (v/v)].

^{1}H NMR (700 MHz, $(CD_3)_2SO$) δ 10.57 (s, 1H), 7.53 (s, 1H), 7.30 (d, *J* = 8.0 Hz, 1H), 7.22 (d, *J* = 8.0 Hz, 1H), 4.16 (m, 1H), 3.61 (s, 1H), 2.97 (dd, *J* = 4.9, 13.7 Hz, 1H), 2.86 (s, 1H), 2.84 (d, *J* = 3.2 Hz, 1H), 2.83 (s, 1H), 2.19 (s, 1H), 1.34 (s, 1H).

^{13}C NMR (175 MHz, $(CD_3)_2SO$, 25 ºC, δ): 172.42, 169.57, 155.25, 140.01, 133.57, 128.83, 119.73, 78.15, 55.08, 51.61, 35.70, 27.96, 22.24.

HRMS (ESI-TOF) (*m/z*): calcd for $C_{17}H_{25}N_2O_4$ ([M + H]$^+$): 370.1973 , found: 370.1978.

_N_-Hydroxy-_N_-(4-(trifluoromethoxy)phenyl)acetamide (1j)

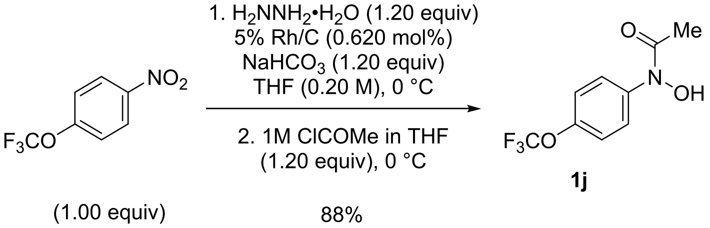

1j

Under nitrogen atmosphere, a suspension of 1-nitro-4-(trifluoromethoxy)benzene (0.716 mL, 5.00 mmol, 1.00 equiv), 5% Rh/C (64.0 mg, 0.0311 mmol, 0.620 mol%), and $NaHCO_3$ (498 mg, 6.00 mmol, 1.20 equiv) in THF (0.200 M, 25.0 mL) was cooled to 0 °C in an ice bath. Hydrazine monohydrate (0.294 mL, 6.00 mmol, 1.20 equiv) was then added dropwise and the reaction mixture was stirred vigorously with a stir bar at 0 °C for 1 h. While the reaction flask was still in the ice bath, a solution of acetyl chloride (0.427 mL, 22.8 mol, 1.20 equiv) in THF (0.100 M, 6.00 mL) was added dropwise and the resulting mixture was stirred at 0 °C for 2 h and then warmed up to 23 °C. After that time, the reaction mixture was concentrated in *vacuo* and the residue was purified by flash column chromatography on silica gel eluting with EtOAc:Hexanes [1:1 (v/v)] to afford the title compound as a light orange solid (1.04 g, 4.39 mol, 88% yield).

R_f = 0.47 EtOAc:Hexanes [1:1 (v/v)]

^{1}H NMR (700 MHz, $(CD_3)_2SO$, 25 ºC, δ): 10.76 (s, 1H), 7.74 (d, *J* = 8.8 Hz, 2H), 7.35 (d, *J* = 8.8 Hz, 2H), 2.21 (s, 3H).

^{13}C NMR (175 MHz, $(CD_3)_2SO$, 25 ºC, δ): 170.18, 144.26, 140.55, 121.09, 119.30, 117.85, 22.36.

^{19}F NMR (376 MHz, $(CD_3)_2SO$, 25 ºC, δ): -57.04 (s, 3F).

HRMS (ESI-TOF) (*m/z*): calcd for $C_9H_9F_3NO_3$ ($[M + H]^+$): 236.0529, found: 236.0532.

_N_-(4-(Difluoromethoxy)phenyl)-_N_-hydroxyacetamide (1k)

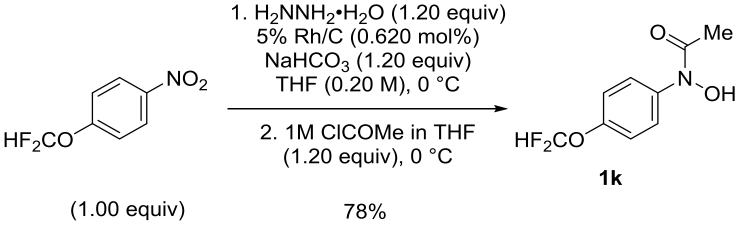

1k

Under nitrogen atmosphere, a suspension of 1-(difluoromethoxy)-4-nitrobenzene (0.700 g, 3.70 mmol, 1.00 equiv), 5% Rh/C (47.3 mg, 0.0230 mmol, 0.620 mol%), and $NaHCO_3$ (0.373 g, 4.44 mmol, 1.20 equiv) in THF (0.200 M, 18.5 mL) was cooled to 0 °C in an ice bath. Hydrazine monohydrate (0.215 mL, 4.44 mmol, 1.20 equiv) was then added dropwise and the reaction mixture was stirred vigorously with a stir bar at 0 °C for 1 h. While the reaction flask was still in the ice bath, a solution of acetyl chloride (0.315 mL, 4.44 mol, 1.20 equiv) in THF (0.100 M, 4.44 mL) was added dropwise and the resulting mixture was stirred at 0 °C for 2 h and then warmed up to 23 °C. After that time, the reaction mixture was concentrated in

vacuo and the residue was purified by flash column chromatography on silica gel eluting with EtOAc:Hexanes [1:10 (v/v)] to afford the title compound as a light orange solid (0.626 g, 2.89 mmol, 78% yield).

R*f* = 0.34 EtOAc:Hexanes [1:1 (v/v)]

^{1}H NMR (700 MHz, $(CD_3)_2SO$, 25 ºC, δ): 10.69 (s, 1H), 7.67 (d, *J* = 7.8 Hz, 2H), 7.25 (t, *J* = 75.5 Hz, 1H), 7.19 (d, *J* = 7.8 Hz, 2H), 2.21 (s, 3H).

^{13}C NMR (175 MHz, $(CD_3)_2SO$, 25 ºC, δ): 138.60, 121.41, 118.68, 117.63, 116.16, 114.70, 22.03.

^{19}F NMR (376 MHz, $(CD_3)_2SO$, 25 ºC, δ): -81.80 (d, *J* = 74.1 Hz, 2F).

HRMS (ESI-TOF) (*m/z*): calcd for $C_9H_{10}F_2NO_3$ ($[M + H]^+$): 218.0623, found: 218.0627.

***N*-(2-Acetylbenzofuran-5-yl)-*N*-hydroxyacetamide (1l)**

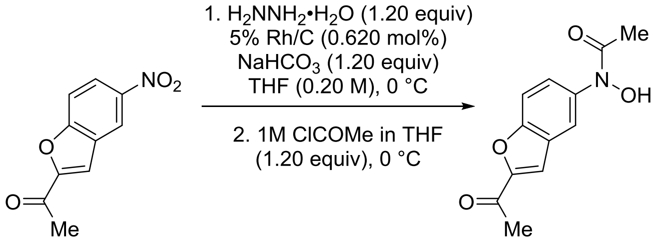

1l

(1.00 equiv) 82%

Under nitrogen atmosphere, a suspension of 1-(5-Nitrobenzofuran-2-yl)ethan-1-one (0.923 g, 4.50 mmol, 1.00 equiv), 5% Rh/C (57.6 mg, 0.0280 mmol, 0.620 mol%), and $NaHCO_3$ (0.448 g, 5.40 mmol, 1.20 equiv) in THF (0.200 M, 22.5 mL) was cooled to 0 °C in an ice bath. Hydrazine monohydrate (0.265 mL, 5.40 mmol, 1.20 equiv) was then added dropwise and the reaction mixture was stirred vigorously with a stir bar at 0 °C for 1 h. While the reaction flask was still in the ice bath, a solution of acetyl chloride (0.384 mL, 5.40 mmol, 1.20 equiv) in THF (0.100 M, 22.5 mL) was added dropwise and the resulting mixture was stirred at 0 °C for 2 h and then warmed up to 23 °C. After that time, the reaction mixture was concentrated in *vacuo* and the residue was purified by flash column chromatography on silica gel eluting with EtOAc:Hexanes [1:1 (v/v)] to afford the title compound as a white solid (0.860 g, 3.69 mmol, 82% yield).

R*f* = 0.55 EtOAc

^{1}H NMR (700 MHz, $(CD_3)_2SO$, 25 ºC, δ): 10.75 (s, 1H), 7.99 (s, 1H), 7.89 (s, 1H), 7.76 (d, *J* = 9.0 Hz, 1H), 7.69 (d, *J* = 9.0 Hz, 1H), 2.54 (d, *J* = 0.9 Hz, 3H), 2.21 (s, 3H).

^{13}C NMR (175 MHz, $(CD_3)_2SO$, 25 ºC, δ): 187.75, 169.81, 152.82, 152.06, 137.93, 126.58, 122.29, 115.00, 114.34, 111.84, 26.31, 22.11.

HRMS (ESI-TOF) (*m/z*): calcd for $C_{12}H_{12}NO_4$ ($[M + H]^+$): 234.0761, found: 234.0766.

Ethyl 5-(*N*-hydroxyacetamido)benzo[*β*]thiophene-2-carboxylate (1m)

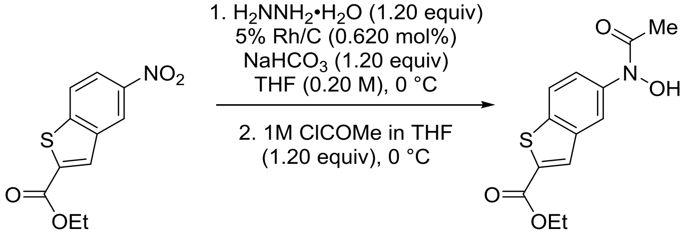

1m

(1.00 equiv) 51%

Under nitrogen atmosphere, a suspension of ethyl 5-nitrobenzo[*β*]thiophene-2-carboxylate (980 mg, 3.90 mmol, 1.00 equiv), 5% Rh/C (49.4.8 mg, 0.0240 mmol, 0.620 mol%), and $NaHCO_3$ (388 mg, 4.68 mmol, 1.20 equiv) in THF (0.200 M, 19.5 mL) was cooled to 0 °C in an ice bath. Hydrazine monohydrate (0.230 mL, 4.68 mmol, 1.20 equiv) was then added dropwise and the reaction mixture was stirred vigorously with a stir bar at 0 °C for 1 h. While the reaction flask was still in the ice bath, a solution of acetyl chloride (0.333 mL, 4.68 mol, 1.20 equiv) in THF (0.100 M, 4.68 mL) was added dropwise and the resulting mixture was stirred at 0 °C for 2 h and then warmed up to 23 °C. After that time, the reaction mixture was concentrated in *vacuo* and the residue was purified by flash column chromatography on silica gel eluting with EtOAc:Hexanes [1:1 (v/v)] to afford the title compound as a beige solid (560 mg, 2.00 mmol, 51% yield).

R*f* = 0.57 EtOAc

^{1}H NMR (700 MHz, $(CD_3)_2SO$, 25 ºC, δ): 10.79 (s, 1H), 8.22 (d, *J* = 4.8 Hz, 1H), 8.03 (d, *J* = 8.0 Hz, 1H), 7.85 (d, *J* = 8.0 Hz, 1H), 4.35 (q, *J* = 7.0 Hz, 1H), 2.25 (s, 1H), 1.33 (t, *J* = 7.0 Hz, 1H).

^{13}C NMR (175 MHz, $(CD_3)_2SO$, 25 ºC, δ): 169.87, 161.71, 139.19, 138.37, 137.18, 133.86, 130.76, 122.61, 120.89, 116.47, 61.29, 22.19, 13.93.

HRMS (ESI-TOF) (*m/z*): calcd for $C_{13}H_{14}NO_4S$ ($[M + H]^+$): 280.0638, found: 280.0642.

***N*-(4-(5-(2-Chlorophenyl)-1,2,4-oxadiazol-3-yl)phenyl)-*N*-hydroxyacetamide (1n)**

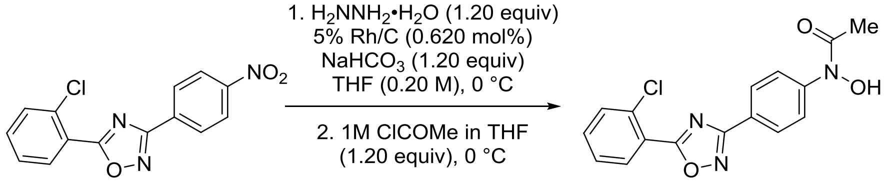

1n

(1.00 equiv) 76%

Under nitrogen atmosphere, a suspension of 5-(2-Chlorophenyl)-3-(4-nitrophenyl)-1,2,4-oxadiazole (480 mg, 1.59 mmol, 1.00 equiv), 5% Rh/C (20.3 mg, 9.86 µmol, 0.620 mol%), and $NaHCO_3$ (158 mg, 1.91 mmol, 1.20 equiv) in THF (0.200 M, 7.95 mL) was cooled to 0 °C in an ice bath. Hydrazine monohydrate (0.0935 mL, 1.91 mmol, 1.20 equiv) was then added dropwise and the reaction mixture was stirred

vigorously with a stir bar at 0 °C for 1 h. While the reaction flask was still in the ice bath, a solution of acetyl chloride (0.136 mL, 1.91 mmol, 1.20 equiv) in THF (0.100 M, 1.91 mL) was added dropwise and the resulting mixture was stirred at 0 °C for 2 h and then warmed up to 23 °C. After that time, the reaction mixture was concentrated in *vacuo* and the residue was purified by flash column chromatography on silica gel eluting with EtOAc:Hexanes [1:1 (v/v)] to afford the title compound as a light yellow solid (398 mg, 1.21 mmol, 76% yield).

R*f* = 0.14 EtOAc:Hexanes [1:5 (v/v)]

^{1}H NMR (700 MHz, $(CD_3)_2SO$) δ 10.84 (s, 1H), 8.13 (dd, *J* = 1.5, 7.8 Hz, 1H), 8.06 (d, *J* = 9.0 Hz, 2H), 7.90 (d, *J* = 8.8 Hz, 2H), 7.72 (dd, *J* = 0.9, 8.1 Hz, 1H), 7.68 (dd, *J* = 1.6, 7.3 Hz, 1H), 7.67 (d, *J* = 1.6 Hz, 1H), 7.58 (dt, *J* = 1.1, 7.5 Hz, 1H), 2.26 (s, 3H).

^{13}C NMR (175 MHz, $(CD_3)_2SO$) δ 173.80, 170.55, 167.48, 144.19, 134.10, 132.30, 132.06, 131.25, 127.87, 127.48, 122.67, 121.13, 119.29, 22.79.

HRMS (ESI-TOF) (*m/z*): calcd for $C_{16}H_{13}N_3O_3Cl$ ($[M + H]^+$): 330.0640, found: 330.0642.

5-(2-Chlorophenyl)-3-(4-nitrophenyl)-1,2,4-oxadiazole (S1)

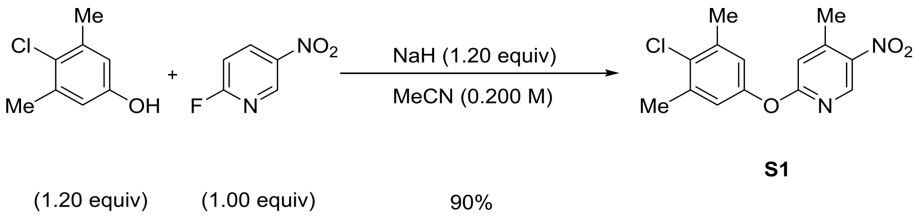

Under nitrogen atmosphere, NaH (60.8 mg, 1.52 mmol, 1.20 equiv, 60% dispersion in mineral oil), 4-chloro-3,5-dimethylphenol (1.00 g, 6.388 mmol, 1.00 equiv) and MeCN (0.200 M, 32.0 mL) was added to a 50 mL round bottom flask. To this suspension, 2-fluoro-5-nitropyridine (0.880 mL, 7.64 mmol. 1.02 equiv) was added dropwise and the resulting mixture was stirred vigorously with a stir bar at 60 °C for 12 h. After that time, the reaction mixture was concentrated in *vacuo* and the residue was purified by chromatography on silica gel, eluting with EtOAc:Hexanes [1:5 to 1:1 (v/v)], to afford the title compound as an orange solid (1.68 g, 5.74 mmol, 90% yield).

R*f* = 0.53 EtOAc:Hexanes [1:1 (v/v)]

^{1}H NMR (700 MHz, $(CD_3)_2SO$, 25 ºC, δ): 8.85 (s, 1H), 7.20 (s, 1H), 7.08 (s, 1H), 3.34 (s, 1H), 2.34 (s, 1H)

^{13}C NMR (175 MHz, $(CD_3)_2SO$, 25 ºC, δ): 165.45, 151.16, 147.94, 146.01, 142.38, 137.80, 130.71, 122.10, 113.91, 20.75, 20.26.

HRMS (ESI-TOF) (*m/z*): calcd for $C_{14}H_{14}N_2O_3Cl$ ($[M + H]^+$): 293.0687, found 293.0691.

N-(6-(4-Chloro-3,5-dimethylphenoxy)-4-methylpyridin-3-yl)-N-hydroxyacetamide (1q)

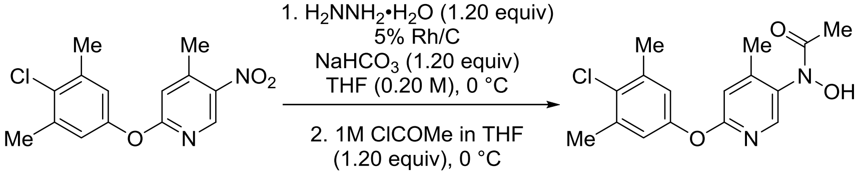

S1 (1.00 equiv) 76% **1q**

Under nitrogen atmosphere, a suspension of 5-(2-chlorophenyl)-3-(4-nitrophenyl)-1,2,4-oxadiazole (480 mg, 1.59 mmol, 1.00 equiv), 5% Rh/C (20.3 mg, 9.86 µmol, 0.620 mol%), and $NaHCO_3$ (158 mg, 1.91 mmol, 1.20 equiv) in THF (0.200 M, 7.95 mL) was cooled to 0 °C in an ice bath. Hydrazine monohydrate (0.0935 mL, 1.91 mmol, 1.20 equiv) was then added dropwise and the reaction mixture was stirred vigorously with a stir bar at 0 °C for 1 h. While the reaction flask was still in the ice bath, a solution of acetyl chloride (0.136 mL, 1.91 mmol, 1.20 equiv) in THF (0.100 M, 1.91 mL) was added dropwise and the resulting mixture was stirred at 0 °C for 2 h and then warmed up to 23 °C. After that time, the reaction mixture was concentrated in *vacuo* and the residue was purified by flash column chromatography on silica gel eluting with EtOAc:Hexanes [1:1 (v/v)] to afford the title compound as a light yellow solid (398 mg, 1.21 mmol, 76% yield).

$\mathbf{R}_f$ = 0.34 EtOAc:Hexanes [1:1 (v/v)]

^{1}H NMR (700 MHz, $(CD_3)_2SO$, 25 ºC, δ): 10.45 (s, 1H), 8.00 (s, 1H), 6.97 (s, 2H), 6.93 (s, 1H), 2.32 (s, 9H), 2.21 (s, 3H).

^{13}C NMR (175 MHz, $(CD_3)_2SO$, 25 ºC, δ): 170.91, 162.18, 151.71, 149.12, 146.09, 137.12, 133.86, 129.46, 121.50, 111.97, 20.79, 20.30, 17.31.

HRMS (ESI-TOF) (*m/z*): calcd for $C_{16}H_{18}N_2O_3Cl$ ([M + H]$^+$): 321.1000, found 321.1002.

2-(((3*aR*,5*R*,6*S*,6*aR*)-5-((*R*)-2,2-dimethyl-1,3-dioxolan-4-yl)-2,2-dimethyltetrahydrofuro[2,3-*d*][1,3]dioxol-6-yl)oxy)-5-nitropyridine (S2)

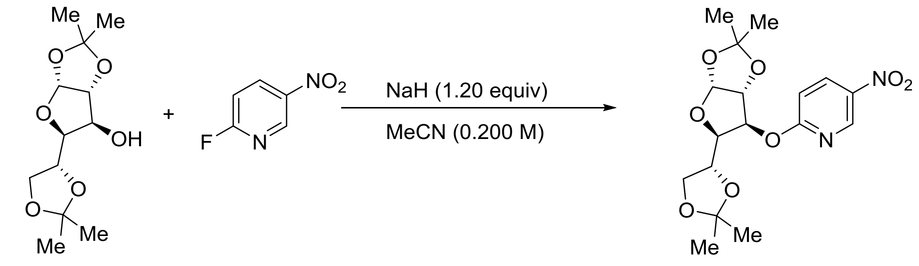

S2

(1.20 equiv) (1.00 equiv) 52%

Under nitrogen atmosphere, NaH (0.289 g, 7.20 mmol, 1.20 equiv, 60% dispersion in mineral oil), diacetone-*D*-glucose (1.00 g, 6.00 mmol, 1.00 equiv) and MeCN (0.200 M, 30.0 mL) was added to a 50 mL round bottom flask. To this suspension, 2-fluoro-5-nitropyridine (0.725 mL, 7.20 mmol. 1.00 equiv)

was added dropwise and the resulting mixture was stirred vigorously with a stir bar at 60 °C for 12 h. After that time, the reaction mixture was concentrated in *vacuo* and the residue was purified by chromatography on silica gel, eluting with EtOAc:Hexanes [1:5 to 1:1 (v/v)], to afford the title compound as white solid (1.19 g, 3.10 mmol, 52% yield).

R$_f$ = 0.15 EtOAc:Hexanes [1:5 (v/v)]

^{1}H NMR (700 MHz, $(CD_3)_2SO$, 25 ºC, δ): 9.14 (d, *J* = 2.7 Hz, 1H), 8.52 (dd, *J* = 2.8, 9.1 Hz, 1H), 7.13 (d, *J* = 9.1 Hz, 1H), 5.94 (d, *J* = 3.6 Hz, 1H), 5.45 (d, *J* = 2.8 Hz, 1H), 4.65 (d, *J* = 3.7 Hz, 1H), 4.37 (q, *J* = 6.2 Hz, 2H), 4.26 (d, *J* = 2.9 Hz, 1H), 4.25 (d, *J* = 2.9 Hz, 1H), 4.07 (q, *J* = 4.9 Hz, 1H), 3.93 (q, *J* = 4.6 Hz, 1H), 1.46 (s, 3H), 1.32 (s, 3H), 1.25 (s, 3H), 1.22 (s, 3H).

^{13}C NMR (175 MHz, CDCl3, 25 ºC, δ): 165.63, 144.98, 140.59, 135.69, 112.48, 111.76, 108.82, 105.13, 82.74, 79.50, 79.06, 72.43, 66.45, 27.05, 26.88, 26.44, 25.57.

HRMS (ESI-TOF) (*m/z*): calcd for $C_{17}H_{23}N_2O_8$ ($[M + H]^+$): 383.1449, found: 383.1455.

_N_-(6-(((3_αR_,5_R_,6_S_,6_αR_)-5-((_R_)-2,2-Dimethyl-1,3-dioxolan-4-yl)-2,2-dimethyltetrahydrofuro[2,3-_d_][1,3]dioxol-6-yl)oxy)pyridin-3-yl)-_N_-hydroxyacetamide (1t)

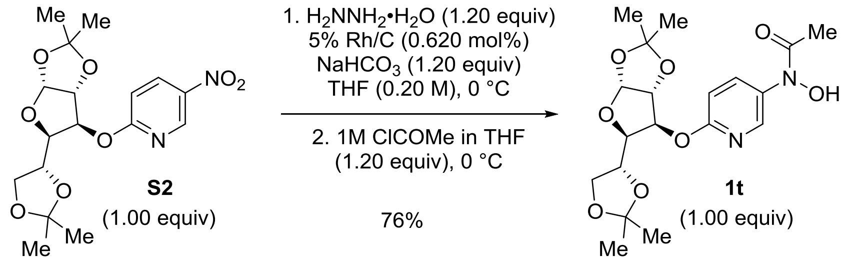

Under nitrogen atmosphere, a suspension of 2-(((3*αR*,5*R*,6*S*,6*αR*)-5-((*R*)-2,2-dimethyl-1,3-dioxolan-4-yl)-2,2-dimethyltetrahydrofuro[2,3-*d*][1,3]dioxol-6-yl)oxy)-5-niropyridine (1.11 g, 2.90 mmol, 1.00 equiv), 5% Rh/C (37.0 mg, 0.0180 mmol, 0.620 mol%), and $NaHCO_3$ (0.289 g, 3.48 mmol, 1.20 equiv) in THF (0.200 M, 14.5 mL) was cooled to 0 °C in an ice bath. Hydrazine monohydrate (0.171 mL, 3.48 mmol, 1.20 equiv) was then added dropwise and the reaction mixture was stirred vigorously with a stir bar at 0 °C for 1 h. While the reaction flask was still in the ice bath, a solution of acetyl chloride (0.247 mL, 3.48 mmol, 1.20 equiv) in THF (0.100 M, 3.48 mL) was added dropwise and the resulting mixture was stirred at 0 °C for 2 h and then warmed up to 23 °C. After that time, the reaction mixture was concentrated in *vacuo* and the residue was purified by flash column chromatography on silica gel eluting with EtOAc:Hexanes [1:1 (v/v)] to afford the title compound as a white solid (0.896g, 2.19 mmol, 76% yield).

R$_f$ = 0.26 EtOAc:Hexanes [1:1 (v/v)]

^{1}H NMR (700 MHz, $(CD_3)_2SO$, 25 ºC, δ): 10.76 (s, 1H), 8.38 (s, 1H), 7.92 (d, *J* = 8.0 Hz, 1H), 6.89 (d, *J* = 8.9 Hz, 1H), 5.90 (d, *J* = 3.7 Hz, 1H), 5.31 (d, *J* = 3.1 Hz, 1H), 4.58 (d, *J* = 3.7 Hz, 1H), 4.35 (q, *J* = 6.2 Hz, 1H), 4.22 (dd, *J* = 3.0, 6.9 Hz, 1H), 4.03 (q, *J* = 4.9 Hz, 1H), 3.92 (q, *J* = 4.6 Hz, 1H), 1.44 (s, 3H), 1.31 (s, 3H), 1.22 (d, *J* = 17.4 Hz, 6H).

^{13}C NMR (175 MHz, CDCl3, 25 ºC, δ): 139.08, 133.51, 132.86, 111.08, 110.70, 108.22, 104.63, 82.42, 79.19, 77.14, 72.07, 65.93, 26.57, 26.47, 25.95, 25.10, 21.74.

HRMS (ESI-TOF) (*m/z*): calcd for $C_{19}H_{27}N_2O_8$ ([M + H]$^+$): 411.1762, found: 411.1763.

N-(4-(*tert*-Butyl)-2-((perfluoropropan-2-yl)oxy)phenyl)acetamide (3a)

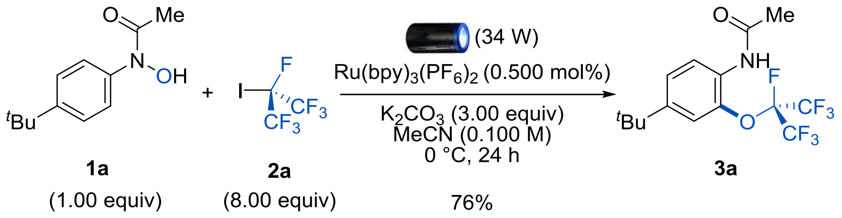

In a glovebox, to an oven-dried 20 mL screw cap vial was added *N*-(4-(*tert*-butyl)phenyl)-*N*-hydroxyacetamide (124 mg, 0.600 mmol, 1.00 equiv), anhydrous K_2CO_3 (249 mg, 1.80 mmol, 3.00 equiv), $Ru(bpy)_3(PF_6)_2$, (2.60 mg, 3.00 μmol, 0.500 mol%), and MeCN (6.00 mL, 0.100 M). To this suspension was added perfluoroisopropyl iodide (0.683 mL, 4.80 mmol, 8.00 equiv) and a magnetic stir bar. Next, the vial was capped, taken out of the glovebox, and cooled to 0 °C in a cryogenic ethanol bath. The reaction mixture was stirred at 0 °C and irradiated with a 34 W blue LED lamp for 24 h. After that time, the reaction vial was removed from the bath and warmed up to 23 °C. The reaction mixture was passed through a plug of Celite. The Celite was then washed with EtOAc (3 x 2.00 mL) and the combined filtrate was concentrated *in vacuo*. The residue was purified by flash chromatography on silica gel, eluting with EtOAc:Hexanes [1:1 (v/v)] to afford the title compound as an off-white solid (172 mg, 0.458 mmol, 76% yield).

R_f = 0.86 MeOH:DCM [1:19 (v/v)].

R_f = 0.73 EtOAc:Hexanes [1:1 (v/v)].

^{1}H NMR (700 MHz, $CDCl_3$, 25 ºC, δ): 8.14 (d, *J* = 8.4 Hz, 1H), 7.36 (br. s, 1H), 7.29 (d, *J* = 8.4 Hz, 1H), 7.23 (s, 1H), 2.17 (s, 3H), 1.30 (s, 9H).

^{13}C NMR (175 MHz, $CDCl_3$, 25 ºC, δ): 168.18, 148.28, 139.91, 127.78, 124.35, 122.39, 119.39 (qd, *J*= 290.2, 35.5Hz), 118.80, 103.66–101.44 (m), 34.59, 31.15, 24.37.

^{19}F NMR (376 MHz, $CDCl_3$, 25 ºC, δ): -78.94 (d, *J* = 3.5 Hz, 6F), -135.33–-135.36 (m, 1F).

HRMS (ESI-TOF) (*m/z*): calcd for $C_{15}H_{17}F_7NO_2$ ([M + H]$^+$): 376.1142, found: 376.1143.

N-(4-Methyl-2-((perfluoropropan-2-yl)oxy)phenyl)acetamide (3b)

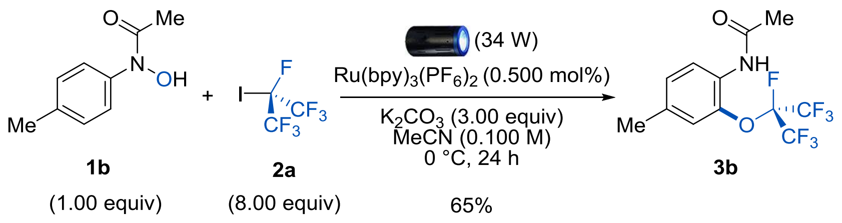

In a glovebox, to an oven-dried 20 mL screw cap vial was added *N*-hydroxy-*N*-(*p*-tolyl)acetamide (99.1 mg, 0.600 mmol, 1.00 equiv), anhydrous K_2CO_3 (249 mg, 1.80 mmol, 3.00 equiv), $Ru(bpy)_3(PF_6)_2$, (2.60 mg, 3.00 μmol, 0.500 mol%), and MeCN (6.00 mL, 0.100 M). To this suspension was added perfluoroisopropyl iodide (0.683 mL, 4.80 mmol, 8.00 equiv) and a magnetic stir bar. Next, the vial was capped, taken out of the glovebox, and cooled to 0 °C in a cryogenic ethanol bath. The reaction mixture was stirred at 0 °C and irradiated with a 34 W blue LED lamp for 24 h. After that time, the reaction vial was removed from the bath and warmed up to 23 °C. The reaction mixture was passed through a plug of Celite. The Celite was then washed with EtOAc (3 x 2.00 mL) and the combined filtrate was concentrated *in vacuo*. The residue was purified by flash chromatography on silica gel, eluting with EtOAc:Hexanes [1:1 (v/v)] to afford the title compound as an off-white solid (131 mg, 0.393 mmol, 65% yield).

R_f = 0.68 MeOH:DCM [1:19 (v/v)].

R_f = 0.70 EtOAc:Hexanes [1:1 (v/v)].

^{1}H NMR (700 MHz, $CDCl_3$, 25 ºC, δ): 8.11 (d, *J* = 8.3 Hz, 1H), 7.30 (br. s, 1H), 7.05 (d, *J* = 8.3 Hz, 1H), 7.02 (s, 1H), 2.30 (s, 3H), 2.15 (s, 3H).

^{13}C NMR (175 MHz, $CDCl_3$, 25 ºC, δ): 168.13, 139.90, 134.72, 128.09, 127.88, 122.53, 122.06, 118.54 (qd, *J* = 289.8, 35.4 Hz), 103.56–101.28 (m), 24.43, 20.95.

^{19}F NMR (376 MHz, $CDCl_3$, 25 ºC, δ): -79.06 (d, *J* = 3.4 Hz, 6F), -135.69–-135.72 (m, 1F).

HRMS (ESI-TOF) (*m/z*): calcd for $C_{12}H_{11}F_7NO_2$ ($[M + H]^+$): 334.0673, found 334.0677.

***N*-(4-Isopropyl-2-((perfluoropropan-2-yl)oxy)phenyl)acetamide (3c)**

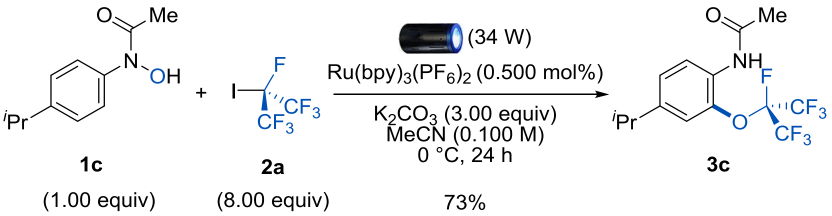

1c (1.00 equiv) **2a** (8.00 equiv) 73% **3c**

In a glovebox, to an oven-dried 20 mL screw cap vial was added *N*-hydroxy-*N*-(4-isopropylphenyl)acetamide (116 mg, 0.600 mmol, 1.00 equiv), anhydrous K_2CO_3 (249 mg, 1.80 mmol, 3.00 equiv), $Ru(bpy)_3(PF_6)_2$, (2.60 mg, 3.00 μmol, 0.500 mol%), and MeCN (6.00 mL, 0.100 M). To this suspension was added perfluoroisopropyl iodide (0.683 mL, 4.80 mmol, 8.00 equiv) and a magnetic stir bar. Next, the vial was capped, taken out of the glovebox, and cooled to 0 °C in a cryogenic ethanol bath. The reaction mixture was stirred at 0 °C and irradiated with a 34 W blue LED lamp for 24 h. After that time, the reaction vial was removed from the bath and warmed up to 23 °C. The reaction mixture was passed through a plug of Celite. The Celite was then washed with EtOAc (3 x 2.00 mL) and the combined filtrate was concentrated *in vacuo*. The residue was purified by flash chromatography on silica gel, eluting with EtOAc:Hexanes [1:1 (v/v)] to afford the title compound as an off-white solid (159 mg, 0.440 mmol, 73% yield).

R$_f$ = 0.84 MeOH:DCM [1:19 (v/v)].

R$_f$ = 0.73 EtOAc:Hexanes [1:1 (v/v)].

^{1}H NMR (700 MHz, $CDCl_3$, 25 ºC, δ): 8.13 (t, *J* = 7.6 Hz, 1H), 7.28 (s, 1H), 7.12 (d, *J* = 7.6 Hz, 1H), 7.06 (s, 1H), 2.86 (sep, *J* = 6.9 Hz, 1H), 2.16 (s, 1H), 1.21 (d, *J* = 6.9 Hz, 1H).

^{13}C NMR (175 MHz, $CDCl_3$, 25 ºC, δ): 168.16, 145.88, 140.03, 128.05, 125.52, 122.64, 119.54, 118.57 (qd, *J* = 289.7, 35.4 Hz), 103.61–101.33 (m), 33.69, 24.47, 23.84.

^{19}F NMR (376 MHz, $CDCl_3$, 25 ºC, δ): -78.99 (d, *J* = 3.5 Hz, 6F), -135.39–-135.42 (m, 1F).

HRMS (ESI-TOF) (*m/z*): calcd for $C_{14}H_{15}F_7NO_2$ ($[M + H]^+$): 362.0986, found 362.0986.

***N-(*3,5-Dimethyl-2-((perfluoropropan-2-yl)oxy)phenyl)acetamide (3d)**

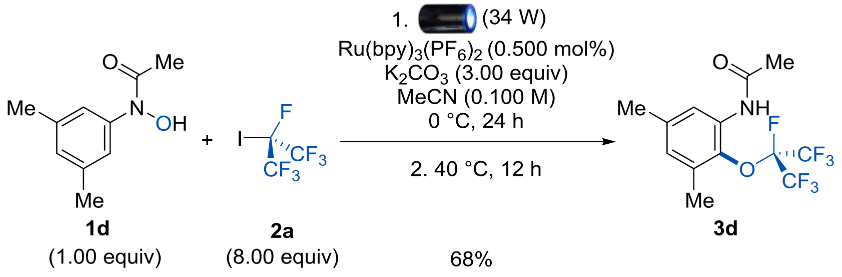

In a glovebox, to an oven-dried 20 mL screw cap vial was added *N*-(3,5-dimethylphenyl)-*N*-hydroxyacetamide (108 mg, 0.600 mmol, 1.00 equiv), anhydrous K_2CO_3 (249 mg, 1.80 mmol, 3.00 equiv), $Ru(bpy)_3(PF_6)_2$, (2.60 mg, 3.00 µmol, 0.500 mol%), and MeCN (6.00 mL, 0.100 M). To this suspension was added perfluoroisopropyl iodide (0.683 mL, 4.80 mmol, 8.00 equiv) and a magnetic stir bar. Next, the vial was capped, taken out of the glovebox, and cooled to 0 °C in a cryogenic ethanol bath. The reaction mixture was stirred at 0 °C and irradiated with a 34 W blue LED lamp for 24 h. After that time, the reaction vial was removed from the bath, warmed up to 23 °C, and stirred at 40 °C for 12 h. The reaction mixture was then cooled to 23 °C and passed through a plug of Celite. The Celite was then washed with EtOAc (3 x 2.00 mL) and the combined filtrate was concentrated *in vacuo*. The residue was purified by flash chromatography on silica gel, eluting with EtOAc:Hexanes [1:1 (v/v)] to afford the title compound as an off-white solid (141 mg, 0.405 mmol, 68% yield).

R$_f$ = 0.63 MeOH:DCM [1:19 (v/v)].

R$_f$ = 0.70 EtOAc:Hexanes [1:1 (v/v)].

^{1}H NMR (700 MHz, $CDCl_3$, 25 ºC, δ): 7.79 (s, 1H), 7.34 (br. s, 1H), 6.76 (s, 1H), 2.27 (s, 3H), 2.24 (s, 3H), 2.13 (s, 3H).

^{13}C NMR (175 MHz, $CDCl_3$, 25 ºC, δ): 168.16, 137.91, 137.35, 131.50, 130.95, 128.04, 121.49, 118.60 (qd, *J* = 290.6, 35.9 Hz), 104.29–102.04 (m), 24.35, 21.13, 16.97.

^{19}F NMR (376 MHz, $CDCl_3$, 25 ºC, δ): -78.96 (d, *J* = 2.9 Hz, 6F), -130.38–-130.45 (m, 1F).

HRMS (ESI-TOF) (*m/z*): calcd for $C_{13}H_{13}F_7NO_2$ ($[M + H]^+$): 348.0829, found 348.0829.

Methyl-(*S*)-3-(4-acetamido-3-((perfluoropropan-2-yl)oxy)phenyl)-2-((*tert*-butoxycarbonyl)amino)propanoate (3e)

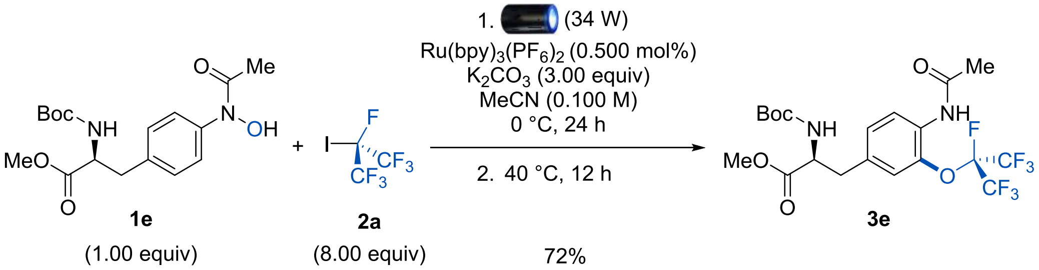

In a glovebox, to an oven-dried 4 mL screw cap vial was added methyl (*S*)-2-((*tert*-butoxycarbonyl)amino)-3-(4-(*N*-hydroxyacetamido)phenyl)propanoate (106 mg, 0.300 mmol, 1.00 equiv), anhydrous K_2CO_3 (124 mg, 0.900 mmol, 3.00 equiv), $Ru(bpy)_3(PF_6)_2$, (1.30 mg, 1.50 µmol, 0.500 mol%), and MeCN (3.00 mL, 0.100 M). To this suspension was added perfluoroisopropyl iodide (0.342 mL, 4.80 mmol, 8.00 equiv) and a magnetic stir bar. Next, the vial was capped, taken out of the glovebox, and cooled to 0 °C in a cryogenic ethanol bath. The reaction mixture was stirred at 0 °C and irradiated with a 34 W blue LED lamp for 24 h. After that time, the reaction vial was removed from the bath, warmed up to 23 °C, and stirred at 40 °C for 12 h. The reaction mixture was then cooled to 23 °C and passed through a plug of Celite. The Celite was then washed with EtOAc (3 x 2.00 mL) and the combined filtrate was concentrated in vacuo. The residue was purified by preparative TLC, developing with EtOAc:Hexanes [1:1 (v/v)] to afford the title compound as a light yellow solid (113 mg, 0.217 mmol, 72% yield).

$\mathbf{R}_f$ = 0.68 MeOH:DCM [1:99 (v/v)].

$\mathbf{R}_f$ = 0.63 EtOAc:Hexanes [1:1 (v/v)].

^{1}H NMR (700 MHz, $CDCl_3$, 25 ºC, δ): 8.19 (d, *J* = 8.3 Hz, 1H), 7.34 (br. s, 1H), 6.99 (d, *J* = 8.3 Hz, 2H), 6.98 (s, 1H), 5.03 (d, *J* = 7.5 Hz, 1H), 4.53 (q, *J* = 6.9 Hz, 1H), 3.68 (s, 3H), 3.04 (m, 2H), 2.14 (s, 3H), 1.40 (s, 9H).

^{13}C NMR (175 MHz, $CDCl_3$, 25 ºC, δ): 171.85, 168.21, 155.03, 139.61, 132.72, 129.35, 128.51, 122.51, 122.40, 116.81 (qd, *J* = 289.1, 35.3 Hz), 103.56–101.25 (m), 80.16, 54.29, 52.37, 37.51, 28.27, 24.48.

^{19}F NMR (376 MHz, $CDCl_3$, 25 ºC, δ): -79.01 (m, 6F), -135.53–-135.60 (m, 1F).

HRMS (ESI-TOF) (*m/z*): calcd for $C_{20}H_{24}F_7NO_2$ ([M + H]$^+$): 521.1517, found 521.1517.

N-(4-Fluoro-2-((perfluoropropan-2-yl)oxy)phenyl)acetamide (3f)

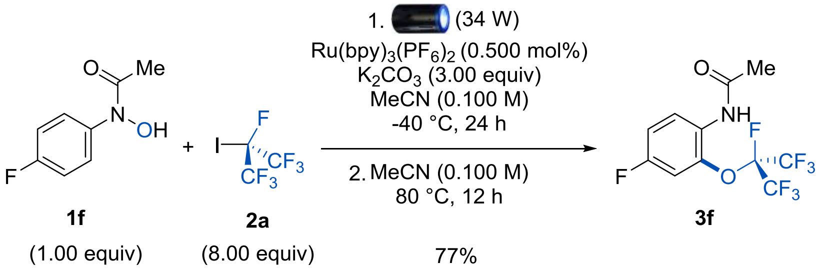

In a glovebox, to an oven-dried 4 mL screw cap vial was added *N*-(4-fluorophenyl)-*N*-hydroxyacetamide (50.7 mg, 0.300 mmol, 1.00 equiv), anhydrous K_2CO_3 (124 mg, 0.900 mmol, 3.00 equiv), $Ru(bpy)_3(PF_6)_2$, (1.30 mg, 1.50 µmol, 0.500 mol%), and MeCN (3.00 mL, 0.100 M). To this suspension was added perfluoroisopropyl iodide (0.342 mL, 4.80 mmol, 8.00 equiv) and a magnetic stir bar. Next, the vial was capped, taken out of the glovebox, and cooled to -40 °C in a cryogenic ethanol bath. The reaction mixture was stirred at -40 °C and irradiated with a 34 W blue LED lamp for 24 h. After that time, the reaction vial was removed from the bath and warmed up to 23 °C. The reaction mixture was passed through a plug of Celite to a 20 mL vial. The Celite was then washed with EtOAc (3 x 1.00 mL) and the combined filtrate in the vial was concentrated *in vacuo*. Under ambient atmosphere the residue in the vial was dissolved in MeCN (3.00 mL, 0.100 M) and a stir bar was added. The vial was capped and stirred at 80 °C for 12 h. After that period, the reaction mixture was directly concentrated in *vacuo* and the residue was purified by preparative TLC, developing with EtOAc:Hexanes [1:1 (v/v)] to afford the title compound as a light yellow solid (78.2 mg, 0.232 mmol, 77% yield).

$\mathbf{R}_f$ = 0.68 MeOH:DCM [1:99 (v/v)].

$\mathbf{R}_f$ = 0.68 EtOAc:Hexanes [1:1 (v/v)].

^{1}H NMR (700 MHz, $CDCl_3$, 25 ºC, δ): 8.20 (m, 1H), 7.32 (br. s, 1H), 6.98 (m, 2H), 2.15 (s, 3H).

^{13}C NMR (175 MHz, $CDCl_3$, 25 ºC, δ): 168.26, 158.36 (d, *J* = 246.7 Hz), 140.24 (d, *J* = 10.5 Hz), 126.89 (d, *J* = 3.1 Hz), 123.98 (d, *J* = 8.7 Hz), 118.40 (qd, *J* = 290.1, 35.1 Hz), 114.40 (d, *J* = 21.7 Hz), 109.72 (d, *J* = 26.4 Hz), 103.58–101.28 (m), 24.30.

^{19}F NMR (376 MHz, $CDCl_3$, 25 ºC, δ): -79.09 (d, *J* = 3.3 Hz, 6F), -115.56–115.64 (m, 1F), -136.64–-136.70 (m, 1F).

HRMS (ESI-TOF) (*m/z*): calcd for $C_{11}H_8F_8NO_2$ ($[M + H]^+$): 338.0422, found 338.0425.

***N*-(4-Chloro-2-((perfluoropropan-2-yl)oxy)phenyl)acetamide (3g)**

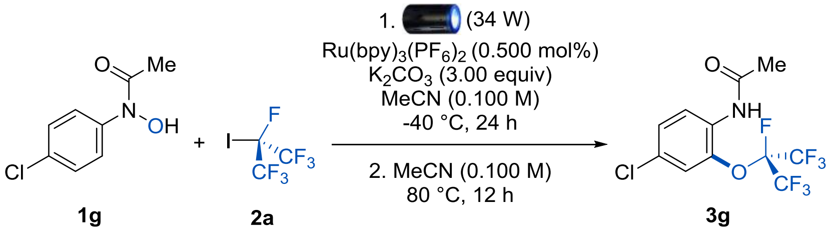

1g (1.00 equiv) **2a** (8.00 equiv) 76% **3g**

In a glovebox, to an oven-dried 4 mL screw cap vial was added *N*-(4-chlorophenyl)-*N*-hydroxyacetamide (55.7 mg, 0.300 mmol, 1.00 equiv), anhydrous K_2CO_3 (124 mg, 0.900 mmol, 3.00 equiv), $Ru(bpy)_3(PF_6)_2$, (1.30 mg, 1.50 µmol, 0.500 mol%), and MeCN (3.00 mL, 0.100 M). To this suspension was added perfluoroisopropyl iodide (0.342 mL, 4.80 mmol, 8.00 equiv) and a magnetic stir bar. Next, the vial was capped, taken out of the glovebox, and cooled to -40 °C in a cryogenic ethanol bath. The reaction mixture was stirred at -40 °C and irradiated with a 34 W blue LED lamp for 24 h. After that time, the reaction vial was removed from the bath and warmed up to 23 °C. The reaction mixture was passed through a plug of Celite to a 20 mL vial. The Celite was then washed with EtOAc (3 x 1.00 mL) and the combined filtrate in the vial was concentrated *in vacuo*. Under ambient atmosphere the residue in the vial was dissolved in MeCN (3.00 mL, 0.100 M) and a stir bar was added. The vial was capped and stirred at 80 °C for 12 h. After that period, the reaction mixture was directly concentrated in *vacuo* and the residue was purified by preparative TLC, developing with EtOAc:Hexanes [1:1 (v/v)] to afford the title compound as an off-white solid (80.7 mg, 0.228 mmol, 76% yield).

$\mathbf{R}_f$ = 0.68 MeOH:DCM [1:19 (v/v)].

$\mathbf{R}_f$ = 0.73 EtOAc:Hexanes [1:1 (v/v)].

^{1}H NMR (700 MHz, $CDCl_3$, 25 ºC, δ): 8.25 (d, *J* = 8.8 Hz, 1H), 7.37 (br. s, 1H), 7.22 (d, *J* = 8.8 Hz, 1H), 7.22 (s, 1H), 2.16 (s, 3H).

^{13}C NMR (175 MHz, $CDCl_3$, 25 ºC, δ): 168.24, 139.81, 129.35, 129.04, 127.77, 123.26, 122.02, 119.22 (qd, *J* = 290.1, 34.7 Hz), 103.49–101.25 (m), 24.46.

^{19}F NMR (376 MHz, $CDCl_3$, 25 ºC, δ): -79.08 (d, *J* = 3.5 Hz, 6F), -136.37–-136.41 (m, 1F).

HRMS (ESI-TOF) (*m/z*): calcd for $C_{11}H_8F_7NO_2Cl$ ([M + H]$^+$): 354.0126 , found 354.0131.

*N-(*4-Bromo-2-((perfluoropropan-2-yl)oxy)phenyl)acetamide (3h)

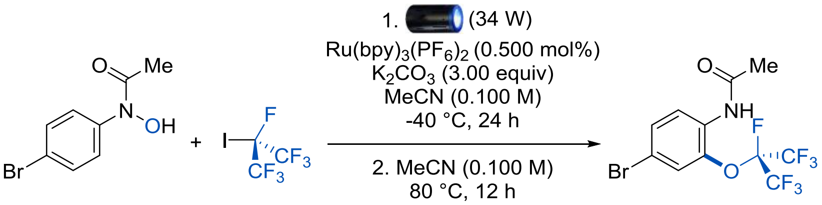

1h (1.00 equiv) **2a** (8.00 equiv) 79% **3h**

In a glovebox, to an oven-dried 4 mL screw cap vial was added *N*-(4-bromophenyl)-*N*-hydroxyacetamide (69.0 mg, 0.300 mmol, 1.00 equiv), anhydrous K_2CO_3 (124 mg, 0.900 mmol, 3.00 equiv), $Ru(bpy)_3(PF_6)_2$, (1.30 mg, 1.50 µmol, 0.500 mol%), and MeCN (3.00 mL, 0.100 M). To this suspension was added perfluoroisopropyl iodide (0.342 mL, 4.80 mmol, 8.00 equiv) and a magnetic stir bar. Next, the vial was capped, taken out of the glovebox, and cooled to -40 °C in a cryogenic ethanol bath. The reaction mixture was stirred at -40 °C and irradiated with a 34 W blue LED lamp for 24 h. After that time, the reaction vial was removed from the bath and warmed up to 23 °C. The reaction mixture was passed through a plug of Celite to a 20 mL vial. The Celite was then washed with EtOAc (3 x 1.00 mL) and the combined filtrate in the vial was concentrated *in vacuo*. Under ambient atmosphere the residue in the vial was dissolved in MeCN (3.00 mL, 0.100 M) and a stir bar was added. The vial was capped and stirred at 80 °C for 12 h. After that period, the reaction mixture was directly concentrated in *vacuo* and the residue was purified by preparative TLC, developing with EtOAc:Hexanes [1:1 (v/v)] to afford the title compound as a light yellow solid (94.5 mg, 0.237 mmol, 79% yield).

R_f = 0.68 MeOH:DCM [1:19 (v/v)].

R_f = 0.73 EtOAc:Hexanes [1:1 (v/v)].

^{1}H NMR (700 MHz, $CDCl_3$, 25 ºC, δ): 8.19 (d, *J* = 8.6 Hz, 1H), 7.39 (br. s, 1H), 7.37 (d, *J* = 8.6 Hz, 1H), 7.35 (s, 1H), 2.15 (s, 3H).

^{13}C NMR (175 MHz, $CDCl_3$, 25 ºC, δ): 168.25, 139.89, 130.71, 129.81, 124.80, 123.56, 118.38 (qd, *J* = 290.1, 34.8 Hz), 116.05, 103.49–101.23 (m), 24.47.

^{19}F NMR (376 MHz, $CDCl_3$, 25 ºC, δ): -79.10 (d, *J* = 3.5 Hz, 6F), -136.31–-136.36 (m, 1F).

HRMS (ESI-TOF) (*m/z*): calcd for $C_{11}H_8F_7NO_2Br$ ($[M + H]^+$): 397.9621, found 397.9630.

*N-(*4-Iodo-2-((perfluoropropan-2-yl)oxy)phenyl)acetamide (3i)

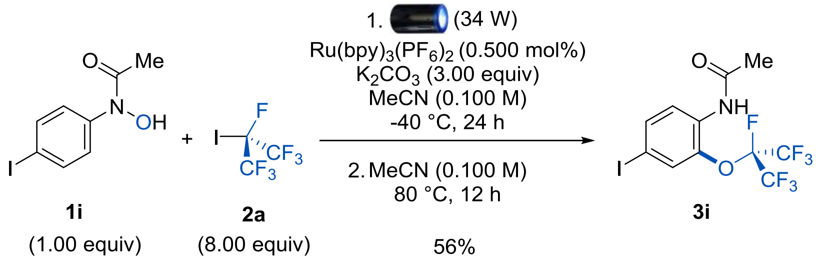

In a glovebox, to an oven-dried 4 mL screw cap vial was added *N*-hydroxy-*N*-(4-iodophenyl)acetamide (83.1 mg, 0.300 mmol, 1.00 equiv), anhydrous K_2CO_3 (124 mg, 0.900 mmol, 3.00 equiv), $Ru(bpy)_3(PF_6)_2$, (1.30 mg, 1.50 μmol, 0.500 mol%), and MeCN (3.00 mL, 0.100 M). To this suspension was added perfluoroisopropyl iodide (0.342 mL, 4.80 mmol, 8.00 equiv) and a magnetic stir bar. Next, the vial was capped, taken out of the glovebox, and cooled to -40 °C in a cryogenic ethanol bath. The reaction mixture was stirred at -40 °C and irradiated with a 34 W blue LED lamp for 24 h. After that time, the reaction vial was removed from the bath and warmed up to 23 °C. The reaction mixture was passed through a plug of Celite to a 20 mL vial. The Celite was then washed with EtOAc (3 x 1.00 mL) and the combined filtrate in the vial was concentrated *in vacuo*. Under ambient atmosphere the residue in the vial was dissolved in MeCN (3.00 mL, 0.100 M) and a stir bar was added. The vial was capped and stirred at 80 °C for 12 h. After that period, the reaction mixture was directly concentrated in *vacuo* and the residue was purified by preparative TLC, developing with EtOAc:Hexanes [1:1 (v/v)] to afford the title compound as a light yellow solid (73.4 mg, 0.167 mmol, 56% yield).

$\mathbf{R}_f$ = 0.80 MeOH:DCM [1:99 (v/v)].

$\mathbf{R}_f$ = 0.73 EtOAc:Hexanes [1:1 (v/v)].

^{1}H NMR (700 MHz, $CDCl_3$, 25 ºC, δ): 8.11 (d, J = 8.8 Hz, 1H), 7.57 (d, J = 8.8 Hz, 1H), 7.53 (s, 1H), 7.31 (s, 1H), 2.17 (s, 3H).

^{13}C NMR (175 MHz, $CDCl_3$, 25 ºC, δ): 168.14, 139.62, 136.75, 130.58, 130.39, 123.63, 118.40 (qd, J = 289.8, 34.9 Hz), 103.49–101.18 (m), 85.69, 24.68.

^{19}F NMR (376 MHz, $CDCl_3$, 25 ºC, δ): -79.12 (d, J = 3.5 Hz, 6F), -136.17–-136.21 (m, 1F).

HRMS (ESI-TOF) (*m/z*): calcd for $C_{11}H_8F_7NO_2I$ ($[M + H]^+$): 445.9482, found 445.9487.

*N-(*2-((Perfluoropropan-2-yl)oxy)-4-(trifluoromethoxy)phenyl)acetamide (3j)

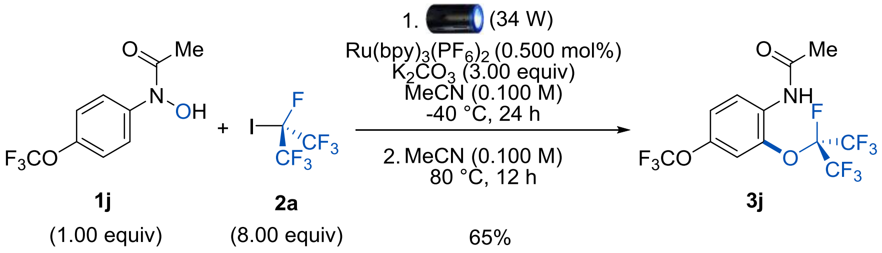

In a glovebox, to an oven-dried 4 mL screw cap vial was added *N*-hydroxy-*N*-(4-(trifluoromethoxy)phenyl)acetamide (70.5 mg, 0.300 mmol, 1.00 equiv), anhydrous K_2CO_3 (124 mg, 0.900 mmol, 3.00 equiv), $Ru(bpy)_3(PF_6)_2$, (1.30 mg, 1.50 µmol, 0.500 mol%), and MeCN (3.00 mL, 0.100 M). To this suspension was added perfluoroisopropyl iodide (0.342 mL, 4.80 mmol, 8.00 equiv) and a magnetic stir bar. Next, the vial was capped, taken out of the glovebox, and cooled to -40 °C in a cryogenic ethanol bath. The reaction mixture was stirred at -40 °C and irradiated with a 34 W blue LED lamp for 24 h. After that time, the reaction vial was removed from the bath and warmed up to 23 °C. The reaction mixture was passed through a plug of Celite to a 20 mL vial. The Celite was then washed with EtOAc (3 x 1.00 mL) and the combined filtrate in the vial was concentrated *in vacuo*. Under ambient atmosphere the residue in the vial was dissolved in MeCN (3.00 mL, 0.100 M) and a stir bar was added. The vial was capped and stirred at 80 °C for 12 h. After that period, the reaction mixture was directly concentrated in *vacuo* and the residue was purified by preparative TLC, developing with EtOAc:Hexanes [1:1 (v/v)] to afford the title compound as an off-white solid (78.6 mg, 0.195 mmol, 65% yield).

R_f = 0.72 MeOH:DCM [1:19 (v/v)].

R_f = 0.73 EtOAc:Hexanes [1:1 (v/v)].

^{1}H NMR (700 MHz, $CDCl_3$, 25 ºC, δ): 8.36 (d, *J* = 9.2 Hz, 1H), 7.37 (br. s, 1H), 7.15 (d, *J* = 9.2 Hz, 1H), 7.12 (s, 1H), 2.18 (s, 3H).

^{13}C NMR (175 MHz, $CDCl_3$, 25 ºC, δ): 168.29, 144.43, 139.57, 129.56, 123.09, 120.47 (q, *J* = 258.1 Hz), 120.38, 116.75 (qd, *J* = 290.1, 34.7 Hz), 115.39, 103.63–101.37 (m), 24.48.

^{19}F NMR (376 MHz, $CDCl_3$, 25 ºC, δ): -59.01 (s, 3F), -79.03 (d, *J* = 3.4 Hz, 6F), -136.49–-136.53 (m, 1F).

HRMS (ESI-TOF) (*m/z*): calcd for $C_{12}H_8F_{10}NO_3$ ($[M + H]^+$): 404.0339, found 404.0342.

***N*-(4-(Difluoromethoxy)-2-((perfluoropropan-2-yl)oxy)phenyl)acetamide (3k)**

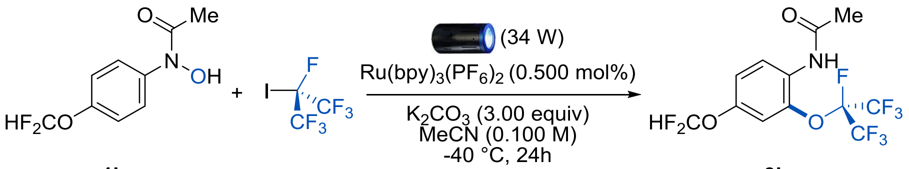

1k (1.00 equiv) **2a** (8.00 equiv) 66% **3k**

In a glovebox, to an oven-dried 20 mL screw cap vial was added *N*-(4-(difluoromethoxy)phenyl)-*N*-hydroxyacetamide (65.2 mg, 0.300 mmol, 1.00 equiv), anhydrous K_2CO_3 (124mg, 0.900 mmol, 3.00 equiv), $Ru(bpy)_3(PF_6)_2$, (1.30 mg, 1.50 µmol, 0.500 mol%), and MeCN (3.00 mL, 0.100 M). To this suspension was added perfluoroisopropyl iodide (0.342 mL, 2.40 mmol, 8.00 equiv) and a magnetic stir bar. Next, the vial was capped, taken out of the glovebox, and cooled to -40 °C in a cryogenic ethanol bath. The reaction mixture was stirred at -40 °C and irradiated with a 34 W blue LED lamp for 24 h. After that time, the reaction vial was removed from the bath and warmed up to 23 °C. The reaction mixture was passed through a plug of Celite. The Celite was then washed with EtOAc (3 x 1.00 mL) and the combined filtrate was

concentrated *in vacuo*. The residue was purified by preparative TLC, developing with EtOAc:Hexanes [1:1 (v/v)] to afford the title compound as an off-white solid (76.0 mg, 0.198 mmol, 66% yield).

R*f* = 0.62 MeOH:DCM [1:19 (v/v)].

R*f* = 0.70 EtOAc:Hexanes [1:1 (v/v)].

^{1}H NMR (700 MHz, $CDCl_3$, 25 ºC, δ): 8.28 (d, *J* = 8.8 Hz, 1H), 7.33 (br. s, 1H), 7.05 (d, *J* = 8.8 Hz, 1H), 7.05 (s, 1H), 6.46 (t, *J* = 73.1 Hz, 1H), 2.17 (s, 3H).

^{13}C NMR (175 MHz, $CDCl_3$, 25 ºC, δ): 168.27, 146.48, 139.97, 128.25, 123.41, 119.01, 116.78 (qd, *J* = 290.2, 35.0 Hz), 115.68 (t, *J* = 262.2 Hz), 114.37, 103.63–101.35 (m), 24.44.

^{19}F NMR (376 MHz, $CDCl_3$, 25 ºC, δ): -79.05 (d, *J* = 3.4 Hz, 6F), -82.05 (d, *J* = 73.1 Hz, 2F), -136.42–-136.48 (m, 1F).

HRMS (ESI-TOF) (*m/z*): calcd for $C_{12}H_9F_9NO_3$ ($[M + H]^+$): 386.0433, found 386.0438.

*N-(*2-Acetyl-4-((perfluoropropan-2-yl)oxy)benzofuran-5-yl)acetamide (3l)

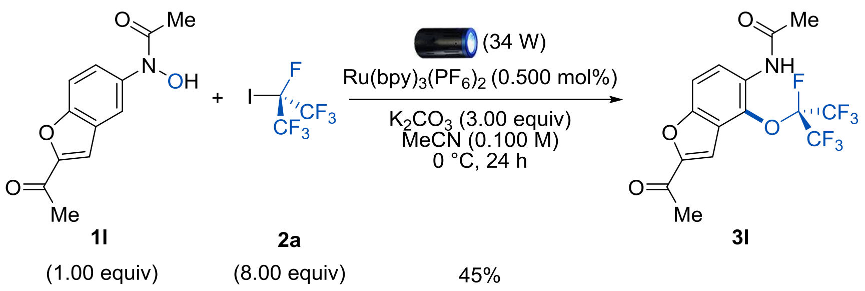

In a glovebox, to an oven-dried 20 mL screw cap vial was added *N*-(2-acetylbenzofuran-5-yl)-*N*-hydroxyacetamide (168 mg, 0.600 mmol, 1.00 equiv), anhydrous K_2CO_3 (249 mg, 1.80 mmol, 3.00 equiv), $Ru(bpy)_3(PF_6)_2$, (2.60 mg, 3.00 µmol, 0.500 mol%), and MeCN (6.00 mL, 0.100 M). To this suspension was added perfluoroisopropyl iodide (0.683 mL, 4.80 mmol, 8.00 equiv) and a magnetic stir bar. Next, the vial was capped, taken out of the glovebox, and cooled to 0 °C in a cryogenic ethanol bath. The reaction mixture was stirred at 0 °C and irradiated with a 34 W blue LED lamp for 24 h. After that time, the reaction vial was removed from the bath and warmed up to 23 °C. The reaction mixture was passed through a plug of Celite. The Celite was then washed with EtOAc (3 x 2.00 mL) and the combined filtrate was concentrated *in vacuo*. The residue was purified by preparative TLC, developing with EtOAc:Hexanes [1:1 (v/v)] to afford the title compound as an off-white solid (108 mg, 0.270 mmol, 45% yield).

R*f* = 0.41 MeOH:DCM [1:19 (v/v)].

R*f* = 0.48 EtOAc:Hexanes [1:1 (v/v)].

^{1}H NMR (700 MHz, $CDCl_3$, 60 ºC, δ): 8.15 (d, *J* = 9.1 Hz, 1H), 7.57 (s, 1H), 7.43 (d, *J* = 9.1 Hz, 1H), 7.40 (d, *J* = 3.2 Hz, 1H), 2.59 (s, 3H), 2.18 (s, 3H).

^{13}C NMR (175 MHz, $CDCl_3$, 60 ºC, δ): 188.53, 168.64, 153.47, 152.81, 134.41, 127.12, 124.63, 121.92, 116.74 (qd, *J* = 290.1, 34.9 Hz), 111.84, 109.94 (d, *J* = 9.0 Hz), 103.89–101.59 (m), 26.61, 24.04.

^{19}F NMR (376 MHz, $CDCl_3$, 60 ºC, δ): -79.29 (d, *J* = 3.1 Hz, 6F), -133.52–-133.62 (m, 1F).

HRMS (ESI-TOF) (*m/z*): calcd for $C_{15}H_{11}F_7NO_4$ ($[M + H]^+$): 402.0571, found 402.0572.

Ethyl 5-acetamido-4-((perfluoropropan-2-yl)oxy)benzo[*β*]thiophene-2-carboxylate (3m)

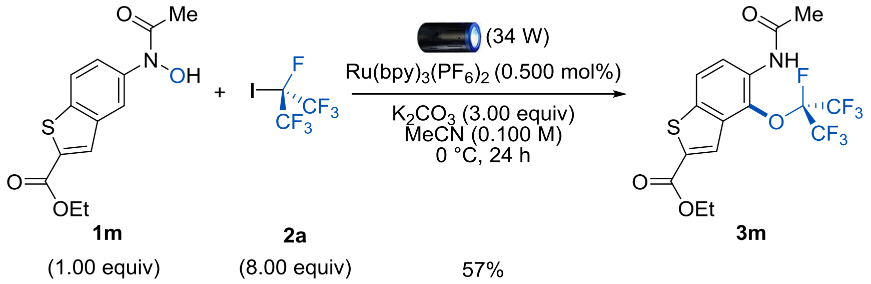

In a glovebox, to an oven-dried 20 mL screw cap vial was added ethyl 5-(*N*-hydroxyacetamido)benzo[*β*]thiophene-2-carboxylate (168 mg, 0.600 mmol, 1.00 equiv), anhydrous K_2CO_3 (249 mg, 1.80 mmol, 3.00 equiv), $Ru(bpy)_3(PF_6)_2$, (2.60 mg, 3.00 μmol, 0.500 mol%), and MeCN (6.00 mL, 0.100 M). To this suspension was added perfluoroisopropyl iodide (0.683 mL, 4.80 mmol, 8.00 equiv) and a magnetic stir bar. Next, the vial was capped, taken out of the glovebox, and cooled to 0 °C in a cryogenic ethanol bath. The reaction mixture was stirred at 0 °C and irradiated with a 34 W blue LED lamp for 24 h. After that time, the reaction vial was removed from the bath and warmed up to 23 °C. The reaction mixture was passed through a plug of Celite. The Celite was then washed with EtOAc (3 x 2.00 mL) and the combined filtrate was concentrated *in vacuo*. The residue was purified by preparative TLC, developing with EtOAc:Hexanes [1:1 (v/v)] to afford the title compound as an off-white solid (154 mg, 0.344 mmol, 57% yield).

$\mathbf{R}_f$ = 0.41 MeOH:DCM [1:19 (v/v)].

$\mathbf{R}_f$ = 0.60 EtOAc:Hexanes [1:1 (v/v)].

^{1}H NMR (700 MHz, $CDCl_3$, 60 ºC, δ): 8.22 (d, *J* = 8.8 Hz, 1H), 7.98 (d, *J* = 2.1 Hz, 1H), 7.70 (d, *J* = 8.8 Hz, 1H), 7.54 (s, 1H), 4.40 (q, *J* = 7.1 Hz, 2H), 2.21 (s, 3H), 1.41 (t, *J* = 7.1 Hz, 3H).

^{13}C NMR (175 MHz, $CDCl_3$, 60 ºC, δ): 168.42, 162.21, 139.50, 136.04, 135.91, 133.12, 128.35, 126.37 (d, *J* = 7.0 Hz), 123.30, 122.17, 118.49 (qd, *J* = 290.0, 34.9 Hz), 104.10–101.87 (m), 62.07, 24.26, 14.33.

^{19}F NMR (376 MHz, $CDCl_3$, 60 ºC, δ): -79.03 (d, *J* = 3.1 Hz, 6F), -132.26–-132.36 (m, 1F).

HRMS (ESI-TOF) (*m/z*): calcd for $C_{16}H_{16}F_7N_2O_4S$ ($[M + NH_4]^+$): 465.0714, found 465.0697.

N-(4-(5-(2-Chlorophenyl)-1,2,4-oxadiazol-3-yl)-2-((perfluoropropan-2-yl)oxy)phenyl)acetamide (3n)

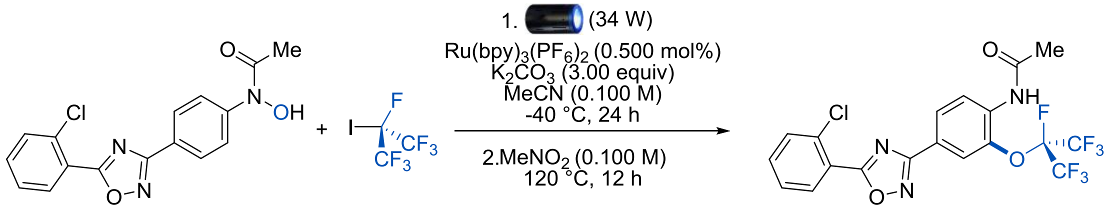

1n (1.00 equiv) **2a** (8.00 equiv) 74% **3n**

In a glovebox, to an oven-dried 4 mL screw cap vial was added *N*-(4-(5-(2-chlorophenyl)-1,2,4-oxadiazol-3-yl)phenyl)-*N*-hydroxyacetamide (65.9 mg, 0.200 mmol, 1.00 equiv), anhydrous K_2CO_3 (82.9 mg, 0.600 mmol, 3.00 equiv), $Ru(bpy)_3(PF_6)_2$, (0.860 mg, 1.00 µmol, 0.500 mol%), and MeCN (2.00 mL, 0.100 M). To this suspension was added perfluoroisopropyl iodide (0.228 mL, 1.60 mmol, 8.00 equiv) and a magnetic stir bar. Next, the vial was capped, taken out of the glovebox, and cooled to -40 °C in a cryogenic ethanol bath. The reaction mixture was stirred at -40 °C and irradiated with a 34 W blue LED lamp for 24 h. After that time, the reaction vial was removed from the bath and warmed up to 23 °C. The reaction mixture was passed through a plug of Celite to a 20 mL vial. The Celite was then washed with EtOAc (3 x 1.00 mL) and the combined filtrate in the vial was concentrated *in vacuo*. Under ambient atmosphere the residue in the vial was dissolved in $MeNO_2$ (2.00 mL, 0.100 M) and a stir bar was added. The vial was capped and stirred at 120 °C for 12 h. After that period, the reaction mixture was directly concentrated in *vacuo* and the residue was purified by preparative TLC, developing with EtOAc:Hexanes [1:1 (v/v)] to afford the title compound as an off-white solid (73.5 mg, 0.148 mmol, 74% yield).

$\mathbf{R}_f$ = 0.72 MeOH:DCM [1:19 (v/v)].

$\mathbf{R}_f$ = 0.75 EtOAc:Hexanes [1:1 (v/v)].

^{1}H NMR (700 MHz, $CDCl_3$, 25 ºC, δ): 8.53 (d, *J* = 7.4 Hz, 1H), 8.12 (d, *J* = 8.5 Hz, 1H), 8.07 (d, *J* = 8.5 Hz, 1H), 8.03 (s, 1H), 7.56 (d, *J* = 7.4 Hz, 1H), 7.55 (s, 1H), 7.50 (t, *J* = 7.4 Hz, 1H), 7.42 (t, *J* = 7.4 Hz, 1H), 2.22 (s, 3H).

^{13}C NMR (175 MHz, $CDCl_3$, 25 ºC, δ): 174.71, 168.28, 167.37, 139.39, 134.00, 133.39, 133.18, 132.07, 131.58, 127.21, 126.95, 123.47, 122.74, 122.04, 120.66, 118.48 (qd, *J* = 290.0, 35.0 Hz), 103.63–101.25 (m), 24.74.

^{19}F NMR (376 MHz, $CDCl_3$, 25 ºC, δ): -78.98 (d, *J* = 3.5 Hz, 6F), -136.05–-136.13 (m, 1F).

HRMS (ESI-TOF) (*m/z*): calcd for $C_{19}H_{12}F_7N_3O_3Cl$ ($[M + H]^+$): 498.0450, found 498.0450.

5-Bromo-3,3-dimethyl-7-((perfluoropropan-2-yl)oxy)indolin-2-one (3o)

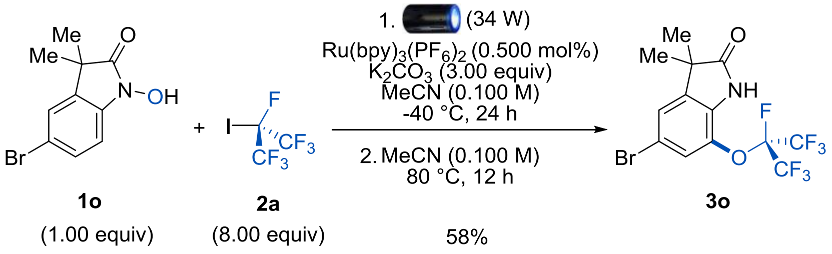

In a glovebox, to an oven-dried 4 mL screw cap vial was added 5-bromo-1-hydroxy-3,3-dimethylindolin-2-one (51.2 mg, 0.200 mmol, 1.00 equiv), anhydrous K_2CO_3 (82.9 mg, 0.600 mmol, 3.00 equiv), $Ru(bpy)_3(PF_6)_2$, (0.860 mg, 1.00 µmol, 0.500 mol%), and MeCN (2.00 mL, 0.100 M). To this suspension was added perfluoroisopropyl iodide (0.228 mL, 1.60 mmol, 8.00 equiv) and a magnetic stir bar. Next, the vial was capped, taken out of the glovebox, and cooled to -40 °C in a cryogenic ethanol bath. The reaction mixture was stirred at -40 °C and irradiated with a 34 W blue LED lamp for 24 h. After that time, the reaction vial was removed from the bath and warmed up to 23 °C. The reaction mixture was passed through a plug of Celite to a 20 mL vial. The Celite was then washed with EtOAc (3 x 1.00 mL) and the combined filtrate in the vial was concentrated *in vacuo*. Under ambient atmosphere the residue in the vial was dissolved in MeCN (3.00 mL, 0.100 M) and a stir bar was added. The vial was capped and stirred at 80 °C for 12 h. After that period, the reaction mixture was directly concentrated in *vacuo* and the residue was purified by HPLC (Luna PFP column), eluting with H_2O:MeCN [1:1 (v/v)] to afford the title compound as an off-white solid (49.4 mg, 0.117 mmol, 58% yield).

R*f* = 0.68 MeOH:DCM [1:19 (v/v)].

R*f* = 0.88 EtOAc:Hexanes [1:1 (v/v)].

^{1}H NMR (700 MHz, $CDCl_3$, 25 ºC, δ): 8.45 (br. s, 1H), 7.27 (s, 1H), 7.24 (s, 1H), 1.40 (s, 6H).

^{13}C NMR (175 MHz, $CDCl_3$, 25 ºC, δ): 181.95, 140.24, 134.05, 131.76, 124.85, 124.54, 118.37 (qd, *J* = 289.8, 34.9 Hz), 114.62, 103.82–101.52 (m), 45.44, 24.34.

^{19}F NMR (376 MHz, $CDCl_3$, 25 ºC, δ): -77.07 (d, *J* = 2.4 Hz, 6F), -136.06–-136.12 (m, 1F).

HRMS (ESI-TOF) (*m/z*): calcd for $C_{13}H_{10}F_7NO_2Br$ ($[M + H]^+$): 423.9778, found 423.9782.

***N*-(2-((Perfluoropropan-2-yl)oxy)-6-(1*H*-pyrazol-1-yl)pyridin-3-yl)acetamide (3p)**

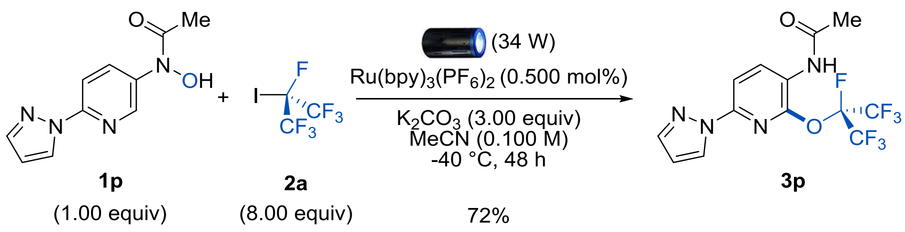

In a glovebox, to an oven-dried 20 mL screw cap vial was added *N*-(6-(1*H*-pyrazol-1-yl)pyridin-3-yl)-*N*-hydroxyacetamide (109 mg, 0.500 mmol, 1.00 equiv), anhydrous K_2CO_3 (207 mg, 1.50 mmol, 3.00 equiv),

$Ru(bpy)_3(PF_6)_2$, (2.10 mg, 2.50 µmol, 0.500 mol%), and MeCN (5.00 mL, 0.100 M). To this suspension was added perfluoroisopropyl iodide (0.569 mL, 4.00 mmol, 8.00 equiv) and a magnetic stir bar. Next, the vial was capped, taken out of the glovebox, and cooled to -40 °C in a cryogenic ethanol bath. The reaction mixture was stirred at -40 °C and irradiated with a 34 W blue LED lamp for 48 h. After that time, the reaction vial was removed from the bath and warmed up to 23 °C. The reaction mixture was passed through a plug of Celite. The Celite was then washed with EtOAc (3 x 2.00 mL) and the combined filtrate was concentrated *in vacuo*. The residue was purified by flash chromatography on silica gel, eluting with EtOAc:Hexanes [1:1 (v/v)] to afford the title compound as an off-white solid (139 mg, 0.360 mmol, 72% yield).

R*f* = 0.68 MeOH:DCM [1:19 (v/v)].

R*f* = 0.70 EtOAc:Hexanes [1:1 (v/v)].

^{1}H NMR (700 MHz, $CDCl_3$, 25 ºC, δ): 8.73 (d, *J* = 8.8 Hz, 1H), 8.26 (d, *J* = 2.5 Hz, 1H), 7.80 (d, *J* = 8.8 Hz, 1H), 7.67 (s, 1H), 7.44 (s, 1H), 6.40 (d, *J* = 1.7 Hz, 1H), 2.18 (s, 3H).

^{13}C NMR (175 MHz, $CDCl_3$, 25 ºC, δ): 168.60, 144.54, 144.06, 142.49, 133.84, 127.10, 121.75, 118.64 (qd, *J* = 289.3, 33.6, Hz), 110.42, 108.22, 103.75–100.99 (m), 24.36.

^{19}F NMR (376 MHz, $CDCl_3$, 25 ºC, δ): -77.97 (d, *J* = 2.7 Hz, 6F), -137.94–-137.97 (m, 1F).

HRMS (ESI-TOF) (*m/z*): calcd for $C_{13}H_{10}F_7N_4O_2$ ($[M + H]^+$): 387.0686, found 387.0688.

*N-(*6-(4-Chloro-3,5-dimethylphenoxy)-4-methyl-2-((perfluoropropan-2-yl)oxy)pyridin-3-yl)acetamide (3q)

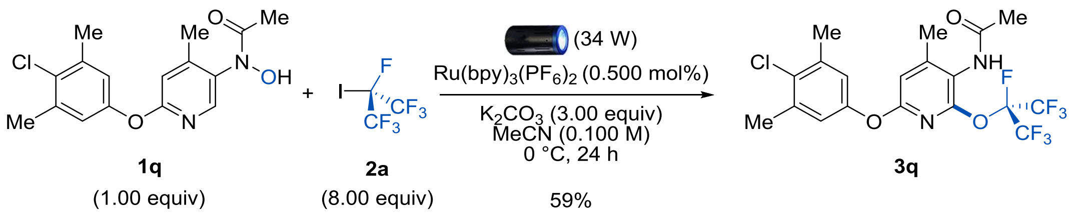

In a glovebox, to an oven-dried 20 mL screw cap vial was added *N*-(6-(4-chloro-3,5-dimethylphenoxy)-4-methylpyridin-3-yl)-*N*-hydroxyacetamide (193 mg, 0.600 mmol, 1.00 equiv), anhydrous K_2CO_3 (249 mg, 1.80 mmol, 3.00 equiv), $Ru(bpy)_3(PF_6)_2$, (2.60 mg, 3.00 µmol, 0.500 mol%), and MeCN (6.00 mL, 0.100 M). To this suspension was added perfluoroisopropyl iodide (0.683 mL, 4.80 mmol, 8.00 equiv) and a magnetic stir bar. Next, the vial was capped, taken out of the glovebox, and cooled to 0 °C in a cryogenic ethanol bath. The reaction mixture was stirred at 0 °C and irradiated with a 34 W blue LED lamp for 24 h. After that time, the reaction vial was removed from the bath and warmed up to 23 °C. The reaction mixture was passed through a plug of Celite. The Celite was then washed with EtOAc (3 x 2.00 mL) and the combined filtrate was concentrated *in vacuo*. The residue was purified by flash chromatography on silica gel, eluting with EtOAc:Hexanes [1:1 (v/v)] to afford the title compound as an off-white solid (174 mg, 0.356 mmol, 59% yield).

R*f* = 0.41 MeOH:DCM [1:19 (v/v)].

R$_f$ = 0.51 EtOAc:Hexanes [1:1 (v/v)].

^{1}H NMR (700 MHz, $CDCl_3$, 25 ºC, δ): 6.84 (s, 2H), 6.81 (br. s, 1H), 6.63 (s, 1H), 2.36 (s, 3H), 2.27 (s, 3H), 2.16 (s, 3H).

^{13}C NMR (175 MHz, $CDCl_3$, 25 ºC, δ): 169.15, 159.60, 153.19, 151.07, 150.73, 137.53, 130.77, 121.10, 118.55 (qd, *J* = 290.4, 34.4 Hz), 116.66, 110.30, 103.56–101.02 (m), 23.01, 20.87, 18.73.

^{19}F NMR (376 MHz, $CDCl_3$, 25 ºC, δ): -78.51 (d, *J* = 2.8 Hz, 6F), -137.77—137.80 (m, 1F).

HRMS (ESI-TOF) (*m/z*): calcd for $C_{19}H_{17}F_7N_2O_3Cl$ ($[M + H]^+$): 489.0810, found 489.0811.

N-(6-Methoxy-4-methyl-2-((perfluoropropan-2-yl)oxy)pyridin-3-yl)acetamide (3r)

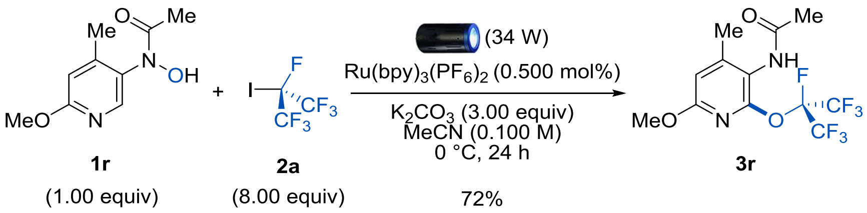

In a glovebox, to an oven-dried 20 mL screw cap vial was added *N*-hydroxy-*N*-(6-methoxy-4-methylpyridin-3-yl)acetamide (118 mg, 0.600 mmol, 1.00 equiv), anhydrous K_2CO_3 (249 mg, 1.80 mmol, 3.00 equiv), $Ru(bpy)_3(PF_6)_2$, (2.60 mg, 3.00 µmol, 0.500 mol%), and MeCN (6.00 mL, 0.100 M). To this suspension was added perfluoroisopropyl iodide (0.683 mL, 4.80 mmol, 8.00 equiv) and a magnetic stir bar. Next, the vial was capped, taken out of the glovebox, and cooled to 0 °C in a cryogenic ethanol bath. The reaction mixture was stirred at 0 °C and irradiated with a 34 W blue LED lamp for 24 h. After that time, the reaction vial was removed from the bath and warmed up to 23 °C. The reaction mixture was passed through a plug of Celite. The Celite was then washed with EtOAc (3 x 2.00 mL) and the combined filtrate was concentrated *in vacuo*. The residue was purified by flash chromatography on silica gel, eluting with EtOAc:Hexanes [1:1 (v/v)] to afford the title compound as an off-white solid (156 mg, 0.429 mmol, 72% yield).

R$_f$ = 0.41 MeOH:DCM [1:19 (v/v)].

R$_f$ = 0.70 EtOAc:Hexanes [1:1 (v/v)].

^{1}H NMR (700 MHz, $CDCl_3$, 60 ºC, δ): 6.64 (br. s, 1H), 6.52 (s, 1H), 3.84 (s, 3H), 2.23 (s, 3H), 2.14 (s, 3H).

^{13}C NMR (175 MHz, $CDCl_3$, 60 ºC, δ): 169.27, 160.89, 152.20, 150.99, 118.86 (qd, *J* = 290.0, 34.8 Hz), 109.61, 103.63–101.35 (m), 53.96, 22.65, 18.17.

^{19}F NMR (376 MHz, $CDCl_3$, 60 ºC, δ): -78.23 (d, *J* = 2.7 Hz, 6F), -137.41—137.44(m, 1F).

HRMS (ESI-TOF) (*m/z*): calcd for $C_{12}H_{12}F_7N_2O_3$ ($[M + H]^+$): 365.0731, found 365.0734.

***N*-(6-(((8*R*,9*S*,13*S*,14*S*)-13-Methyl-17-oxo-7,8,9,11,12,13,14,15,16,17-decahydro-6*H*-cyclopenta[*α*]phenanthren-3-yl)oxy)-2-((perfluoropropan-2-yl)oxy)pyridin-3-yl)acetamide (3s)**

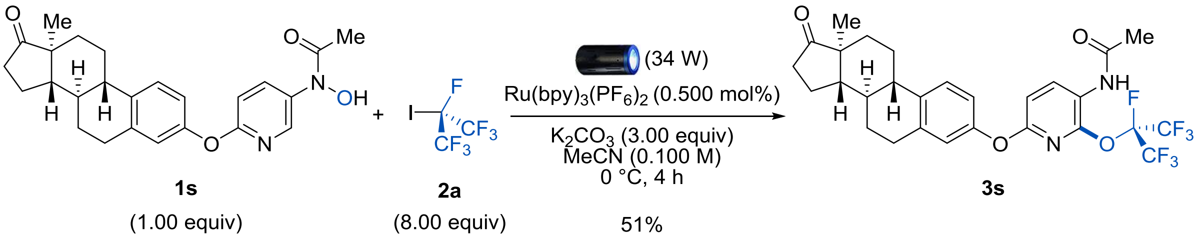

In a glovebox, to an oven-dried 20 mL screw cap vial was added *N*-hydroxy-*N*-(6-(((8*R*,9*S*,13*S*,14*S*)-13-methyl-17-oxo-7,8,9,11,12,13,14,15,16,17-decahydro-6*H*-cyclopenta[*α*]phenanthren-3-yl)oxy)pyridin-3-yl)acetamide (126 mg, 0.300 mmol, 1.00 equiv), anhydrous K_2CO_3 (124 mg, 0.900 mmol, 3.00 equiv), $Ru(bpy)_3(PF_6)_2$, (1.30 mg, 1.50 μmol, 0.500 mol%), and MeCN (3.00 mL, 0.100 M). To this suspension was added perfluoroisopropyl iodide (0.342 mL, 2.40 mmol, 8.00 equiv) and a magnetic stir bar. Next, the vial was capped, taken out of the glovebox, and cooled to 0 °C in a cryogenic ethanol bath. The reaction mixture was stirred at 0 °C and irradiated with a 34 W blue LED lamp for 24 h. After that time, the reaction vial was removed from the bath and warmed up to 23 °C. The reaction mixture was passed through a plug of Celite. The Celite was then washed with EtOAc (3 x 1.00 mL) and the combined filtrate was concentrated *in vacuo*. The residue was purified by preparative TLC, developing with EtOAc:Hexanes [1:1 (v/v)] to afford the title compound as an off-white solid (89.2 mg, 0.152 mmol, 51% yield).

$\mathbf{R}_f$ = 0.46 MeOH:DCM [1:19 (v/v)].

$\mathbf{R}_f$ = 0.56 EtOAc:Hexanes [1:1 (v/v)].

^{1}H NMR (700 MHz, $CDCl_3$, 25 °C, δ): 8.56 (d, *J* = 8.8 Hz, 1H), 7.27 (br. s, 1H), 7.25 (d, *J* = 4.0 Hz, 1H), 6.87 (m, 1H), 6.84 (d, *J* = 2.2 Hz, 1H), 6.75 (d, *J* = 8.8 Hz, 1H), 2.87 (m, 2H), 2.50 (m, 1H), 2.41 (d, *J* = 2.2 Hz, 1H), 2.29 (m, 1H), 2.18 (s, 3H), 2.06 (m, 4H), 1.54 (m, 6H), 0.92 (s, 3H).

^{13}C NMR (175 MHz, $CDCl_3$, 25 °C, δ): 220.96, 168.45, 157.12, 151.61, 144.43, 138.10, 136.46, 135.25, 126.47, 120.94, 119.12, 118.48 (qd, *J* = 289.8, 34.2 Hz), 118.11, 109.25, 103.37–101.04 (m), 50.51, 48.04, 44.19, 38.18, 35.92, 31.62, 29.43, 26.46, 25.88, 24.31, 21.65, 13.90.

^{19}F NMR (376 MHz, $CDCl_3$, 25 °C, δ): -78.45 (d, *J* = 2.7 Hz, 6F), -137.60–-137.63 (m, 1F).

HRMS (ESI-TOF) (*m/z*): calcd for $C_{28}H_{28}F_7N_2O_4$ ($[M + H]^+$): 589.1932, found 589.1934.

***N*-(6-(((3α*R*,5*R*,6*S*,6α*R*)-5-((*R*)-2,2-dimethyl-1,3-dioxolan-4-yl)-2,2-dimethyltetrahydrofuro[2,3-*d*][1,3]dioxol-6-yl)oxy)-2-((perfluoropropan-2-yl)oxy)pyridin-3-yl)acetamide (3t)**

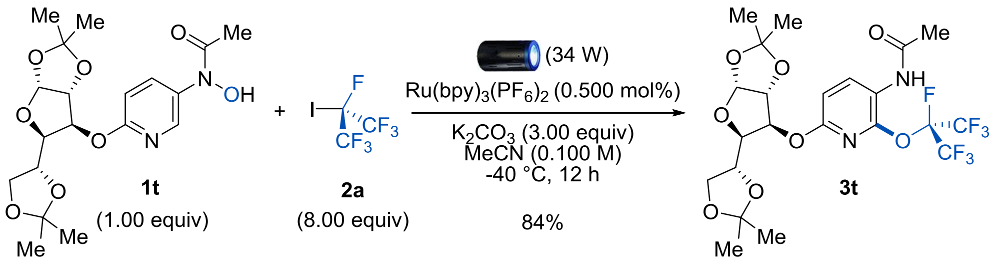

In a glovebox, to an oven-dried 4 mL screw cap vial was added *N*-(6-(((3α*R*,5*R*,6*S*,6α*R*)-5-((*R*)-2,2-dimethyl-1,3-dioxolan-4-yl)-2,2-dimethyltetrahydrofuro[2,3-*d*][1,3]dioxol-6-yl)oxy)pyridin-3-yl)-*N*-hydroxyacetamide (61.6 mg, 0.150 mmol, 1.00 equiv), anhydrous K_2CO_3 (62.2 mg,0.450 mmol, 3.00 equiv), $Ru(bpy)_3(PF_6)_2$, (0.600 mg, 0.750 µmol, 0.500 mol%), and MeCN (1.50 mL, 0.100 M). To this suspension was added perfluoroisopropyl iodide (0.171 mL, 1.20 mmol, 8.00 equiv) and a magnetic stir bar. Next, the vial was capped, taken out of the glovebox, and cooled to -40 °C in a cryogenic ethanol bath. The reaction mixture was stirred at -40 °C and irradiated with a 34 W blue LED lamp for 12 h. After that time, the reaction vial was removed from the bath and warmed up to 23 °C. The reaction mixture was passed through a plug of Celite. The Celite was then washed with EtOAc (3 x 1.00 mL) and the combined filtrate was concentrated *in vacuo*. The residue was purified by preparative TLC, developing with EtOAc:Hexanes [1:1 (v/v)] to afford the title compound as an off-white solid (72.8 mg, 0.126 mmol, 84% yield).

$\mathbf{R}_f$ = 0.51 MeOH:DCM [1:19 (v/v)].

$\mathbf{R}_f$ = 0.56 EtOAc:Hexanes [1:1 (v/v)].

^{1}H NMR (700 MHz, $CDCl_3$, 25 ºC, δ): 8.52 (d, *J* = 8.8 Hz, 1H), 7.12 (s, 1H), 6.70 (d, *J* = 8.8 Hz, 1H), 5.87 (d, *J* = 3.4 Hz, 1H), 5.32 (d, *J* = 2.3 Hz, 1H), 4.56 (d, *J* = 3.4 Hz, 1H), 4.32 (m, 2H), 4.07 (d, *J* = 5.5 Hz, 2H), 2.18 (s, 3H), 1.53 (s, 3H), 1.39 (s, 3H), 1.28 (s, 3H), 1.26 (s, 3H).

^{13}C NMR (175 MHz, $CDCl_3$, 25 ºC, δ): 168.44, 156.15, 144.20, 135.60, 118.68 (qd, *J* = 290.6, 34.4 Hz), 118.61 (qd, *J* = 288.7, 33.9 Hz), 118.38, 112.23, 109.57, 109.31, 105.16, 103.46–101.16 (m), 82.91, 80.21, 78.35, 72.57, 67.18, 26.88, 26.12, 25.33, 24.37.

^{19}F NMR (376 MHz, $CDCl_3$, 25 ºC, δ): -78.42 (m, 6F), -137.81—137.84 (m, 1F).

HRMS (ESI-TOF) (*m/z*): calcd for $C_{22}H_{26}F_7N_2O_8$ ($[M + H]^+$): 579.1572, found 579.1570.

N-(4-(*tert*-Butyl)-2-(trifluoromethoxy)phenyl)acetamide (4a)

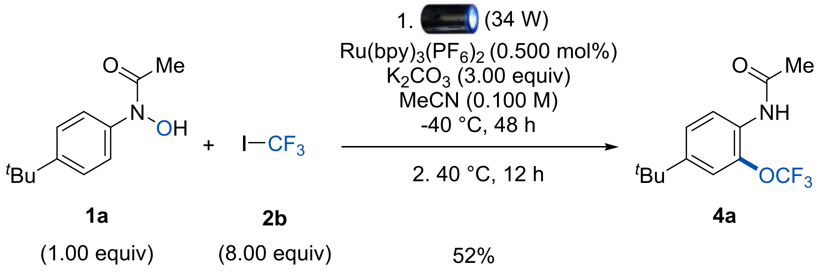

In a glovebox, to an oven-dried 4 mL screw cap vial was added *N*-(4-(*tert*-butyl)phenyl)-*N*-hydroxyacetamide (62.2 mg, 0.300 mmol, 1.00 equiv), anhydrous K_2CO_3 (124 mg, 0.900 mmol, 3.00 equiv), $Ru(bpy)_3(PF_6)_2$, (1.30 mg, 1.50 µmol, 0.500 mol%), and a 0.800 M solution of trifluoromethyl iodide (0.185 mL, 2.40 mmol, 8.00 equiv) in MeCN (3.00 mL, 0.100 M). To this suspension was added a magnetic stir bar. Next, the vial was capped, taken out of the glovebox, and cooled to -40 °C in a cryogenic ethanol bath. The reaction mixture was stirred at -40 °C and irradiated with a 34 W blue LED lamp for 48 h. After that time, the reaction vial was removed from the bath, warmed up to 23 °C, and stirred at 40 °C for 12 h. The reaction mixture was then cooled to 23 °C and passed through a plug of Celite. The Celite was then washed with EtOAc (3 x 2.00 mL) and the combined filtrate was concentrated in vacuo. The residue was purified by preparative TLC, developing with EtOAc:Hexanes [1:1 (v/v)] to afford the title compound as a light yellow solid (42.5 mg, 0.156 mmol, 52% yield).

R_f = 0.63 MeOH:DCM [1:19 (v/v)].

R_f = 0.74 EtOAc:Hexanes [1:1 (v/v)].

^{1}H NMR (700 MHz, $CDCl_3$, 25 ºC, δ): 8.20 (d, *J* = 8.7 Hz, 1H), 7.41 (br. s, 1H), 7.29 (dd, *J* = 1.6, 8.7 Hz, 1H), 7.22 (s, 1H), 2.20 (s, 3H), 1.29 (s, 9H).

^{13}C NMR (175 MHz, $CDCl_3$, 25 ºC, δ): 168.40, 148.29, 138.17, 127.96, 124.55, 122.06, 120.73 (q, *J* = 258.8 Hz), 117.86, 34.64, 31.25, 24.72.

^{19}F NMR (376 MHz, $CDCl_3$, 25 ºC, δ): -57.46 (s, 3F).

HRMS (ESI-TOF) (*m/z*): calcd for $C_{13}H_{17}F_3NO_2$ ($[M + H]^+$): 276.1206, found 276.1209.

N-(2-(Trifluoromethoxy)phenyl)acetamide (4b)

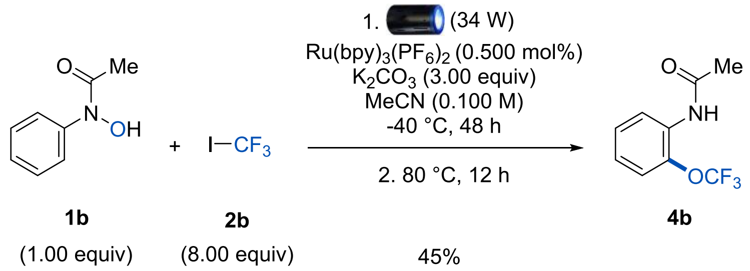

In a glovebox, to an oven-dried 4 mL screw cap vial was added *N*-hydroxy-*N*-phenylacetamide (45.3 mg, 0.300 mmol, 1.00 equiv), anhydrous K_2CO_3 (124 mg, 0.900 mmol, 3.00 equiv), $Ru(bpy)_3(PF_6)_2$, (1.30 mg,

1.50 μmol, 0.500 mol%), and a 0.800 M solution of trifluoromethyl iodide (0.185 mL, 2.40 mmol, 8.00 equiv) in MeCN (3.00 mL, 0.100 M). To this suspension was added a magnetic stir bar. Next, the vial was capped, taken out of the glovebox, and cooled to -40 °C in a cryogenic ethanol bath. The reaction mixture was stirred at -40 °C and irradiated with a 34 W blue LED lamp for 48 h. After that time, the reaction vial was removed from the bath, warmed up to 23 °C, and stirred at 80 °C for 12 h. The reaction mixture was then cooled to 23 °C and passed through a plug of Celite. The Celite was then washed with EtOAc (3 x 2.00 mL) and the combined filtrate was concentrated in vacuo. The residue was purified by preparative TLC, developing with EtOAc:Hexanes [1:1 (v/v)] to afford the title compound as a light yellow solid (29.4 mg, 0.135 mmol, 45% yield).

R*f* = 0.63 MeOH:DCM [1:19 (v/v)].

R*f* = 0.59 EtOAc:Hexanes [1:1 (v/v)].

^{1}H NMR (700 MHz, $CDCl_3$, 25 ºC, δ): 8.37 (d, *J* = 8.1 Hz, 1H), 7.48 (br. s, 1H), 7.27 (t, *J* = 7.7 Hz, 1H), 7.24 (d, *J* = 8.1 Hz, 1H), 7.09 (t, *J* = 7.7 Hz, 1H), 2.22 (s, 3H).

^{13}C NMR (175 MHz, $CDCl_3$, 25 ºC, δ): 168.46, 138.16, 130.65, 127.60, 124.32, 122.19, 120.68 (q, *J* = 259.0 Hz), 120.39, 24.86.

^{19}F NMR (376 MHz, $CDCl_3$, 25 ºC, δ): -57.57 (s, 3F).

HRMS (ESI-TOF) (*m/z*): calcd for $C_9H_9F_3NO_2$ ($[M + H]^+$): 220.0580, found 220.0582.

Methyl (*S*)-3-(4-acetamido-3-(trifluoromethoxy)phenyl)-2-((*tert*-butoxycarbonyl)amino)propanoate (4c)

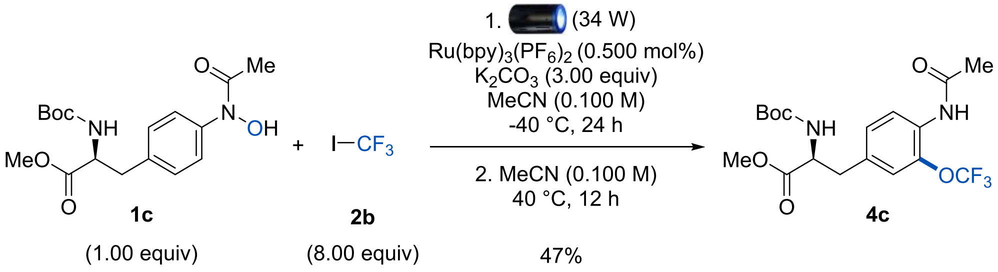

In a glovebox, to an oven-dried 4 mL screw cap vial was added methyl (*S*)-2-((*tert*-butoxycarbonyl)amino)-3-(4-(*N*-hydroxyacetamido)phenyl)propanoate (70.5 mg, 0.200 mmol, 1.00 equiv), anhydrous K_2CO_3 (82.9 mg, 0.600 mmol, 3.00 equiv), $Ru(bpy)_3(PF_6)_2$, (0.860 mg, 1.00 μmol, 0.500 mol%), and a 0.800 M solution of trifluoromethyl iodide (0.123 mL, 1.60 mmol, 8.00 equiv) in MeCN (2.00 mL, 0.100 M). To this suspension was added a magnetic stir bar. Next, the vial was capped, taken out of the glovebox, and cooled to -40 °C in a cryogenic ethanol bath. The reaction mixture was stirred at -40 °C and irradiated with a 34 W blue LED lamp for 48 h. After that time, the reaction vial was removed from the bath and warmed up to 23 °C. The reaction mixture was passed through a plug of Celite to a 20 mL vial. The Celite was then washed with EtOAc (3 x 1.00 mL) and the combined filtrate in the vial was concentrated *in vacuo*. Under ambient atmosphere the residue in the vial was dissolved in MeCN (2.00 mL, 0.100 M) and a stir bar was added. The vial was capped and stirred at 40 °C for 12 h. After that period, the reaction mixture was directly

concentrated in *vacuo* and the residue was purified by preparative TLC, developing with EtOAc:Hexanes [1:1 (v/v)] to afford the title compound as a light yellow solid (32.4 mg, 0.0920 mmol, 47% yield).

R*f* = 0.63 MeOH:DCM [1:19 (v/v)].

R*f* = 0.56 EtOAc:Hexanes [1:1 (v/v)].

^{1}H NMR (700 MHz, $CDCl_3$, 25 ºC, δ): 8.28 (d, *J* = 8.3 Hz, 1H), 7.44 (br. s, 1H), 7.02 (d, *J* = 8.3 Hz, 1H), 6.99 (s, 1H), 5.03 (d, *J* = 7.5 Hz, 1H), 4.55 (d, *J* = 7.5 Hz, 1H), 3.70 (s, 3H), 3.06 (m, 2H), 2.20 (s, 3H), 1.41 (s, 9H).

^{13}C NMR (175 MHz, $CDCl_3$, 25 ºC, δ): 171.91, 168.39, 155.06, 137.95, 132.61, 129.46, 128.52, 122.05, 121.18, 120.62 (q, *J* = 259.4 Hz), 80.19, 54.32, 52.42, 37.61, 28.35, 24.82.

^{19}F NMR (376 MHz, $CDCl_3$, 25 ºC, δ): -57.40 (s, 3F).

HRMS (ESI-TOF) (*m/z*): calcd for $C_{18}H_{24}F_3N_2O_6$ ($[M + H]^+$): 421.1581, found 421.1581.

N-(4-Bromo-2-(trifluoromethoxy)phenyl)acetamide (4d)

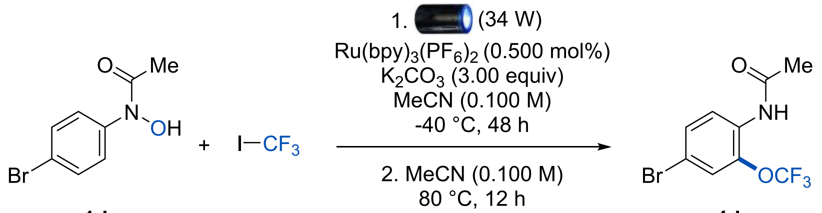

1d (1.00 equiv) **2b** (8.00 equiv) 66% **4d**

In a glovebox, to an oven-dried 4 mL screw cap vial was added *N*-(4-bromophenyl)-*N*-hydroxyacetamide (70.5 mg, 0.200 mmol, 1.00 equiv), anhydrous K_2CO_3 (82.9 mg, 0.600 mmol, 3.00 equiv), $Ru(bpy)_3(PF_6)_2$, (0.860 mg, 1.00 µmol, 0.500 mol%), and a 0.800 M solution of trifluoromethyl iodide (0.123 mL, 1.60 mmol, 8.00 equiv) in MeCN (3.00 mL, 0.100 M). To this suspension was added a magnetic stir bar. Next, the vial was capped, taken out of the glovebox, and cooled to -40 °C in a cryogenic ethanol bath. The reaction mixture was stirred at -40 °C and irradiated with a 34 W blue LED lamp for 48 h. After that time, the reaction vial was removed from the bath and warmed up to 23 °C. The reaction mixture was passed through a plug of Celite to a 20 mL vial. The Celite was then washed with EtOAc (3 x 1.00 mL) and the combined filtrate in the vial was concentrated *in vacuo*. Under ambient atmosphere the residue in the vial was dissolved in MeCN (2.00 mL, 0.100 M) and a stir bar was added. The vial was capped and stirred at 80 °C for 12 h. After that period, the reaction mixture was directly concentrated in *vacuo* and the residue was purified by preparative TLC, developing with EtOAc:Hexanes [1:1 (v/v)] to afford the title compound as a light yellow solid (32.4 mg, 0.0920 mmol, 66% yield).

R*f* = 0.63 MeOH:DCM [1:19 (v/v)].

R*f* = 0.72 EtOAc:Hexanes [1:1 (v/v)].

^{1}H NMR (700 MHz, $CDCl_3$, 25 ºC, δ): 8.29 (d, *J* = 9.2 Hz, 1H), 7.46 (br. s, 1H), 7.39 (d, *J* = 9.2 Hz, 1H), 7.38 (s, 1H), 2.21 (s, 3H).

^{13}C NMR (175 MHz, $CDCl_3$, 25 ºC, δ): 168.44, 138.20, 130.69, 129.86, 123.54, 123.18, 120.51 (q, J = 260.7 Hz), 115.94, 24.85.

^{19}F NMR (376 MHz, $CDCl_3$, 25 ºC, δ): -57.64 (s, 3F).

HRMS (ESI-TOF) (m/z): calcd for $C_9H_8F_3NO_2$ ($[M + H]^+$): 297.9685, found 297.9693.

(±)-*N*-(4-(*tert*-Butyl)-2-((perfluorobutan-2-yl)oxy)phenyl)acetamide (4e)

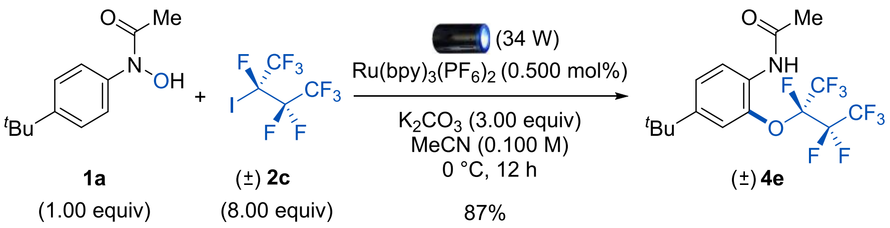

In a glovebox, to an oven-dried 4 mL screw cap vial was added *N*-(4-(*tert*-butyl)phenyl)-*N*-hydroxyacetamide (62.2 mg, 0.300 mmol, 1.00 equiv), anhydrous K_2CO_3 (124 mg, 0.900 mmol, 3.00 equiv), $Ru(bpy)_3(PF_6)_2$, (1.30 mg, 1.50 µmol, 0.500 mol%), and MeCN (3.00 mL, 0.100 M). To this suspension was added (±)-1,1,1,2,2,3,4,4,4-nonafluoro-3-iodobutane (0.396 mL, 2.40 mmol, 8.00 equiv) and a magnetic stir bar. Next, the vial was capped, taken out of the glovebox, and cooled to 0 °C in a cryogenic ethanol bath. The reaction mixture was stirred at 0 °C and irradiated with a 34 W blue LED lamp for 12 h. After that time, the reaction vial was removed from the bath and warmed up to 23 °C. The reaction mixture was passed through a plug of Celite. The Celite was then washed with EtOAc (3 x 2.00 mL) and the combined filtrate was concentrated in vacuo. The residue was purified by flash chromatography on silica gel, eluting with EtOAc:Hexanes [1:1 (v/v)] to afford the title compound as an off-white solid (111 mg, 0.261 mmol, 87% yield).

R_f = 0.63 MeOH:DCM [1:19 (v/v)].

R_f = 0.74 EtOAc:Hexanes [1:1 (v/v)].

^{1}H NMR (700 MHz, $CDCl_3$, 25 ºC, δ): 8.19 (d, J = 8.6 Hz, 1H), 7.32 (dd, J = 1.6, 8.6 Hz, 1H), 7.23 (br. s, 2H), 2.15 (s, 3H), 1.30 (s, 9H).

^{13}C NMR (175 MHz, $CDCl_3$, 25 ºC, δ): 168.12, 148.31, 140.10, 127.50, 124.38, 122.41, 118.47, 121.33–116.12 (m), 120.46–115.21 (m), 111.34–107.57 (m), 105.31–103.09 (m), 34.59, 31.12, 24.31.

^{19}F NMR (376 MHz, $CDCl_3$, 25 ºC, δ): -75.01 (t, J = 10.9 Hz, 3F), -79.82 (dd, J = 1.9, 6.8 Hz, 3F), -123.42–-126.25 (m, 2F), -129.64 (s, 1F).

HRMS (ESI-TOF) (m/z): calcd for $C_{16}H_{17}F_9NO_2$ ($[M + H]^+$): 426.1110, found 426.1111.

N-(4-(*tert*-Butyl)-2-((perfluorohexyl)oxy)phenyl)acetamide (4f)

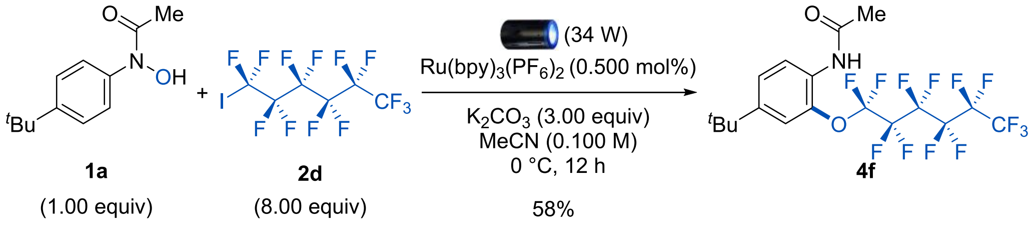

In a glovebox, to an oven-dried 4 mL screw cap vial was added *N*-(4-(*tert*-butyl)phenyl)-*N*-hydroxyacetamide (41.5 mg, 0.200 mmol, 1.00 equiv), anhydrous K_2CO_3 (82.9 mg, 0.600 mmol, 3.00 equiv), $Ru(bpy)_3(PF_6)_2$, (0.860 mg, 1.00 µmol, 0.500 mol%), and MeCN (3.00 mL, 0.100 M). To this suspension was added 1,1,1,2,2,3,3,4,4,5,5,6,6-tridecafluoro-6-iodohexane (0.346 mL, 1.60 mmol, 8.00 equiv) and a magnetic stir bar. Next, the vial was capped, taken out of the glovebox, and cooled to 0 °C in a cryogenic ethanol bath. The reaction mixture was stirred at 0 °C and irradiated with a 34 W blue LED lamp for 12 h. After that time, the reaction vial was removed from the bath and warmed up to 23 °C. The reaction mixture was passed through a plug of Celite. The Celite was then washed with EtOAc (3 x 1.00 mL) and the combined filtrate was concentrated *in vacuo*. The residue was purified by flash chromatography on silica gel, eluting with EtOAc:Hexanes [1:1 (v/v)] to afford the title compound as an off-white solid (59.6 mg, 0.116 mmol, 58% yield).

R*f* = 0.63 MeOH:DCM [1:19 (v/v)].

R*f* = 0.76 EtOAc:Hexanes [1:1 (v/v)].

^{1}H NMR (700 MHz, $CDCl_3$, 25 ºC, δ): 8.14 (d, *J* = 8.7 Hz, 1H), 7.27 (dd, *J* = 1.9, 8.7 Hz, 1H), 7.21 (s, 1H), 2.14 (s, 3H), 1.27 (s, 9H).

^{13}C NMR (175 MHz, $CDCl_3$, 25 ºC, δ): 168.19, 148.51, 137.46, 128.24, 124.88, 122.29, 118.70, 119.92–114.55 (m, 2C), 112.56–106.76 (m, 4C), 34.67, 31.25, 24.40.

^{19}F NMR (376 MHz, $CDCl_3$, 25 ºC, δ): -80.82 (t, *J* = 15.0 Hz, 3F), -82.31 (s, 2F), -121.84 (s, 2F), -122.74 (d, *J* = 5.9 Hz, 2F), -124.76–-124.98 (m, 2F), -126.02–-126.18 (m, 2F).

HRMS (ESI-TOF) (*m/z*): calcd for $C_{18}H_{17}F_{13}NO_2$ ($[M + H]^+$): 526.1046, found 526.1047.

N-(4-(*tert*-Butyl)-2-(2-chloro-1,1,2,2-tetrafluoroethoxy)phenyl)acetamide (4g)

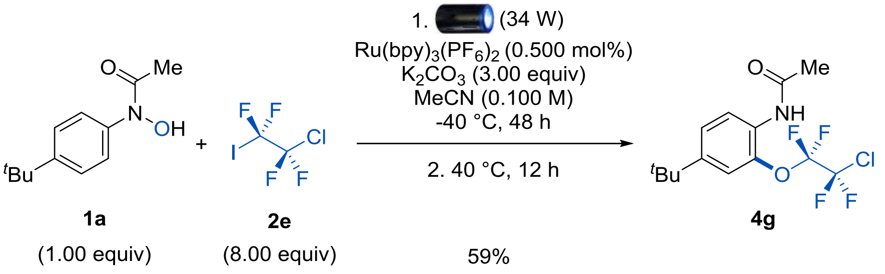

In a glovebox, to an oven-dried 4 mL screw cap vial was added *N*-(4-(*tert*-butyl)phenyl)-*N*-hydroxyacetamide (62.2 mg, 0.300 mmol, 1.00 equiv), anhydrous K_2CO_3 (124 mg, 0.900 mmol, 3.00

equiv), $Ru(bpy)_3(PF_6)_2$, (1.30 mg, 1.50 μmol, 0.500 mol%), and MeCN (3.00 mL, 0.100 M). To this suspension was added 1-chloro-1,1,2,2-tetrafluoro-2-iodoethane (0.342 mL, 2.40 mmol, 8.00 equiv) and a magnetic stir bar. Next, the vial was capped, taken out of the glovebox, and cooled to -40 °C in a cryogenic ethanol bath. The reaction mixture was stirred at -40 °C and irradiated with a 34 W blue LED lamp for 48 h. After that time, the reaction vial was removed from the bath, warmed up to 23 °C, and stirred at 40 °C for 12 h. The reaction mixture was then cooled to 23 °C and passed through a plug of Celite. The Celite was then washed with EtOAc (3 x 2.00 mL) and the combined filtrate was concentrated in vacuo. The residue was purified by preparative TLC, developing with EtOAc:Hexanes [1:1 (v/v)] to afford the title compound as a light yellow solid (60.6 mg, 0.177 mmol, 59% yield).

R*f* = 0.63 MeOH:DCM [1:19 (v/v)].

R*f* = 0.69 EtOAc:Hexanes [1:1 (v/v)].

^{1}H NMR (700 MHz, $CDCl_3$, 25 ºC, δ): 8.20 (d, *J* = 8.6 Hz, 1H), 7.33 (s, 1H), 7.31 (dd, *J* = 2.0, 8.6 Hz, 1H), 7.24 (s, 1H), 2.16 (s, 3H), 1.30 (s, 9H).

^{13}C NMR (175 MHz, CDCl3, 25 ºC, δ): 168.18, 148.33, 137.56, 128.55, 124.78, 121.97, 120.83 (tt, *J* = 44.14, 299.7 Hz), 118.82, 116.11 (tt, *J* = 34.3, 277.0 Hz), 34.64, 31.26, 24.62.

^{19}F NMR (376 MHz, $CDCl_3$, 25 ºC, δ): -72.61 (t, *J* = 4.0 Hz, 2F), -86.78 (t, *J* = 4.0 Hz, 2F).

HRMS (ESI-TOF) (*m/z*): calcd for $C_{14}H_{17}F_4NO_2Cl$ ($[M + H]^+$): 342.0878, found 342.0881.

***N*-(2-(2-Bromo-1,1,2,2-tetrafluoroethoxy)-4-(*tert*-butyl)phenyl)acetamide (4h)**

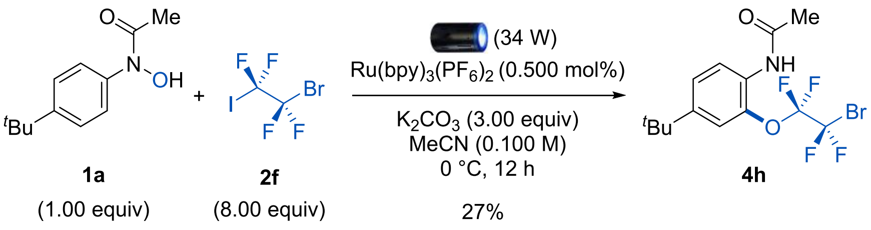

1a (1.00 equiv) **2f** (8.00 equiv) **4h**

In a glovebox, to an oven-dried 4 mL screw cap vial was added *N*-(4-(*tert*-butyl)phenyl)-*N*-hydroxyacetamide (62.2 mg, 0.300 mmol, 1.00 equiv), anhydrous K_2CO_3 (124 mg, 0.900 mmol, 3.00 equiv), $Ru(bpy)_3(PF_6)_2$, (1.30 mg, 1.50 μmol, 0.500 mol%), and MeCN (3.00 mL, 0.100 M). To this suspension was added 1-bromo-1,1,2,2-tetrafluoro-2-iodoethane (0.446 mL, 2.40 mmol, 8.00 equiv) and a magnetic stir bar. Next, the vial was capped, taken out of the glovebox, and cooled to 0 °C in a cryogenic ethanol bath. The reaction mixture was stirred at 0 °C and irradiated with a 34 W blue LED lamp for 12 h. After that time, the reaction vial was removed from the bath and warmed up to 23 °C. The reaction mixture was passed through a plug of Celite. The Celite was then washed with EtOAc (3 x 2.00 mL) and the combined filtrate was concentrated in vacuo. The residue was purified by flash chromatography on silica gel, eluting with EtOAc:Hexanes [1:1 (v/v)] to afford the title compound as an off-white solid (31.8 mg, 0.0810 mmol, 27% yield).

R*f* = 0.63 MeOH:DCM [1:19 (v/v)].

R*f* = 0.68 EtOAc:Hexanes [1:1 (v/v].

^{1}H NMR (700 MHz, $CDCl_3$, 25 ºC, δ): 8.22 (d, *J* = 8.7 Hz, 1H), 7.37 (s, 1H), 7.31 (dd, *J* = 2.0, 8.7 Hz, 1H), 7.24 (s, 1H), 2.18 (s, 3H), 1.30 (s, 9H).

^{13}C NMR (175 MHz, $CDCl_3$, 25 ºC, δ): 168.17, 148.25, 137.46, 128.60, 121.83, 118.80, 116.08 (tt, *J* = 31.8, 277.2 Hz), 113.74 (tt, *J* = 44.1, 312.8 Hz), 34.66, 31.28, 24.73.

^{19}F NMR (376 MHz, $CDCl_3$, 25 ºC, δ): -67.82 (t, *J* = 4.9 Hz, 2F), -86.30 (t, *J* = 4.9 Hz, 2F).

HRMS (ESI-TOF) (*m/z*): calcd for $C_{14}H_{17}F_4NO_2Br$ ($[M + H]^+$): 386.0373, found 386.0378.

Mechanistic Studies

Stern–Volmer Luminescence Quenching

Emission intensities were recorded using a Perkin Elmer LS50B Luminescence spectrometer. All quenching data was recorded using a 1.00 cm screw-top quartz cuvette at 23 °C in the presence of $Ru(bpy)_3(PF_6)_2$ (10.0 μM) and varying concentration of quencher in degassed MeCN. Excitation of the sample was performed at 452 nm with a slit width of 10.0 nm and emission was detected at 615 nm. After acquisition, the data was plotted according to the Stern-Volmer equation shown below.

$$\frac{\mathbf{I_o}}{\mathbf{I}} = \mathbf{1 + k_q \tau_o [Q]}$$

Where I_o is the luminescence intensity in the absence of the quencher, I is the intensity in the presence of the quencher, k_q is the quenching rate, τ_o is the life-time of the photoredox catalyst, and [Q] is the concentration of the quencher.

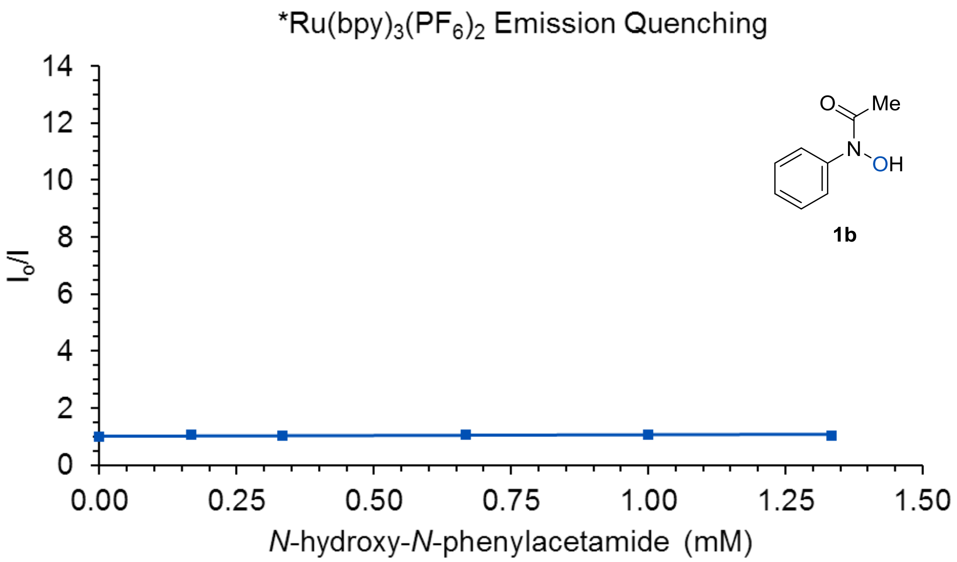

Figure S3. $Ru(bpy)_3^{2+}$ emission quenching by *N*-hydroxy-*N*-phenylacetamide. No observable quenching was detected.

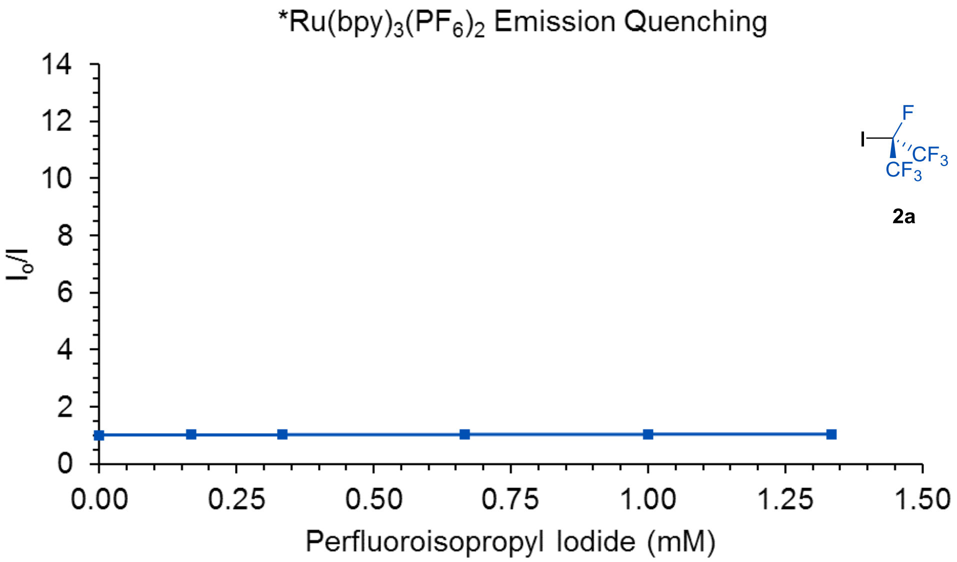

Figure S4. $Ru(bpy)_3^{2+}$ emission quenching by perfluoroisopropyl iodide. No observable quenching was detected.

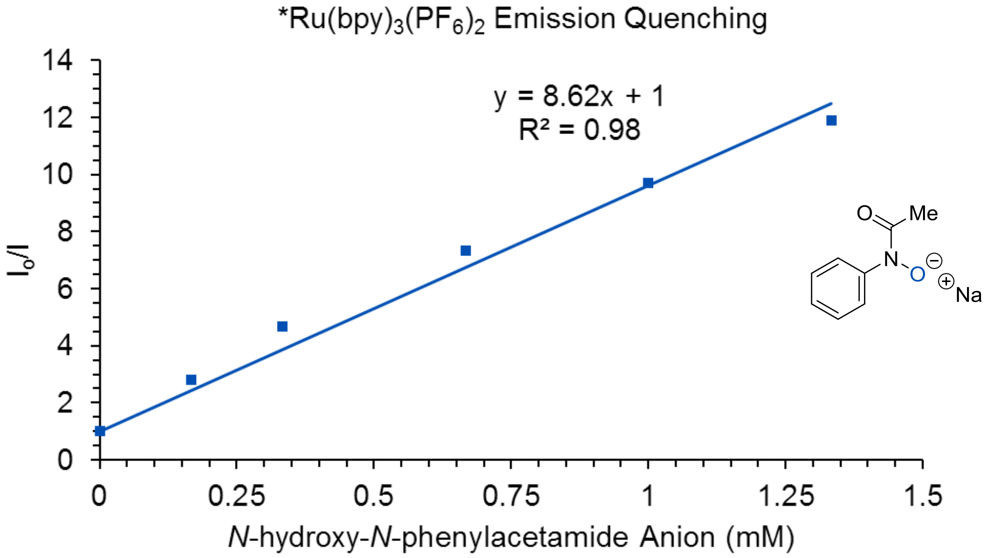

Figure S5. $Ru(bpy)_3^{2+}$ emission quenching by *N*-hydroxy-*N*-phenylacetamide anion. For the *N*-hydroxy-*N*-phenylacetamide anion, $k_q = 7.84 \times 10^9$ M^{-1} s^{-1} was observed.

Spectroscopic Data

^{1}H NMR (700 MHz, $(CD_3)_2SO$, 25 °C) of (1a)

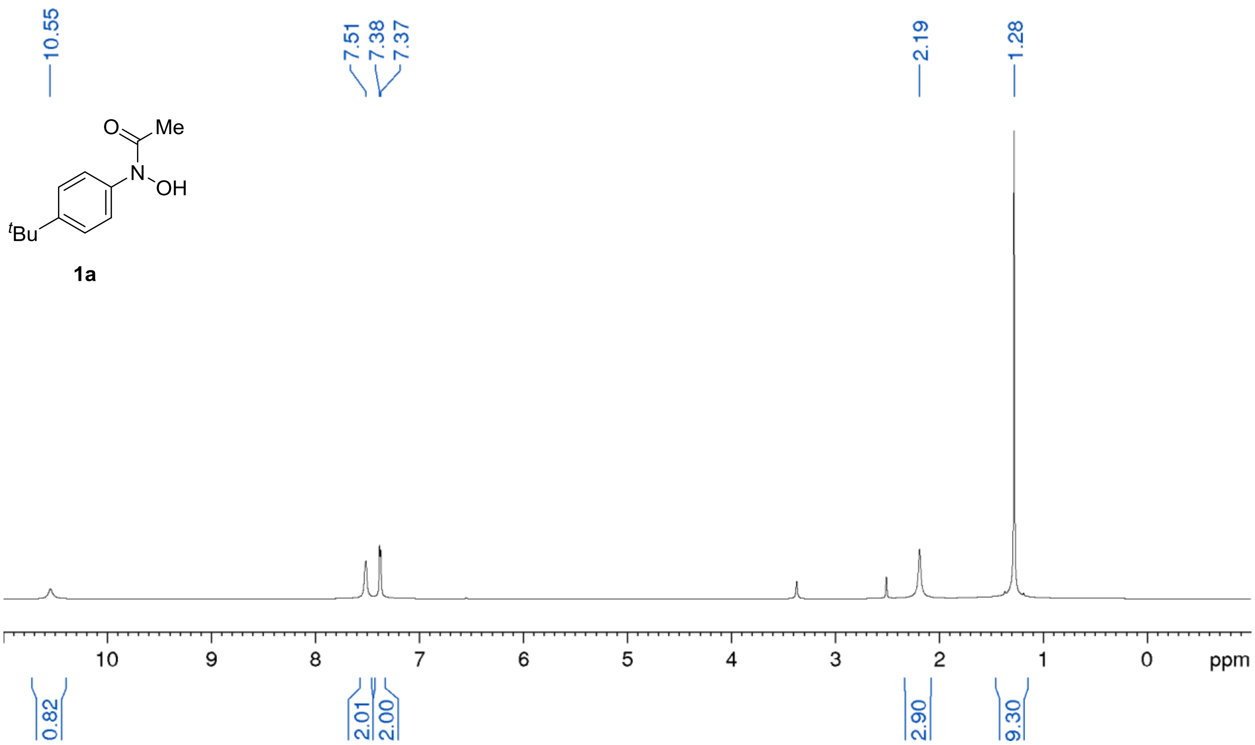

^{13}C NMR (175 MHz, $(CD_3)_2SO$, 25 °C) of (1a)

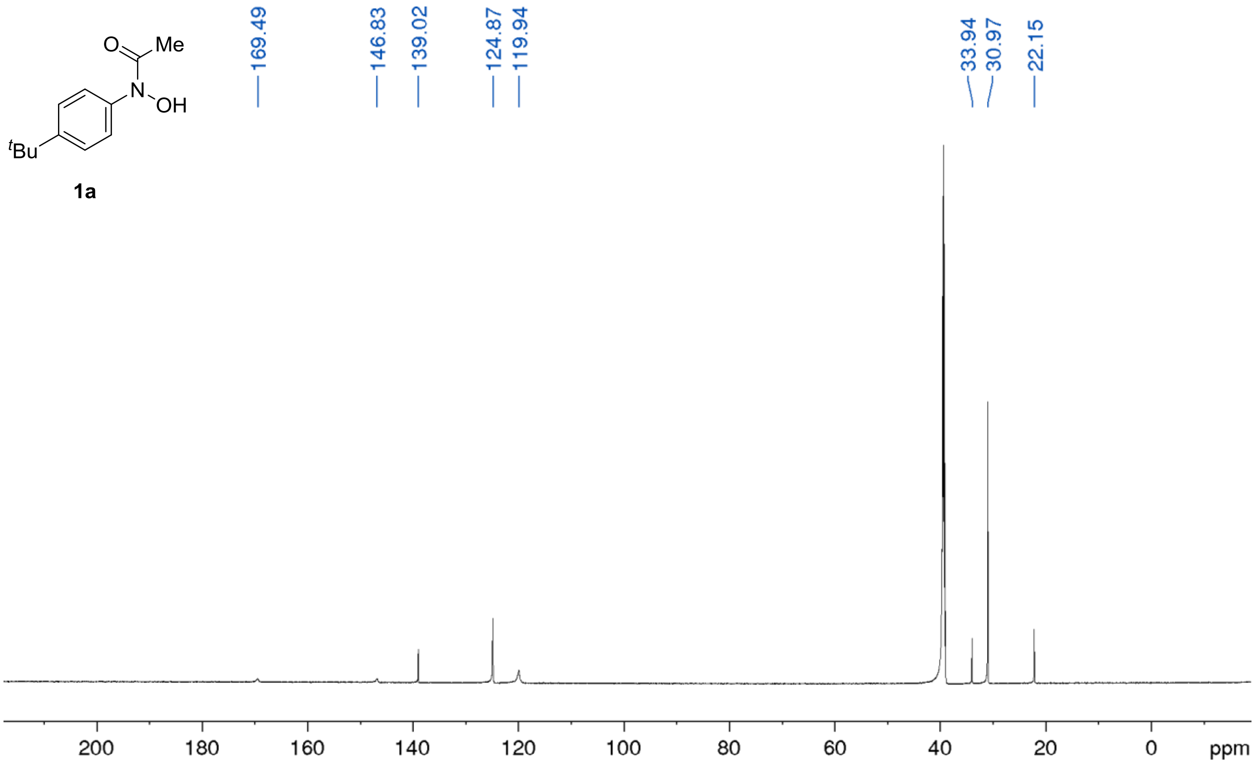

^{1}H NMR (700 MHz, $(CD_3)_2SO$, 25 ºC) of (1c)

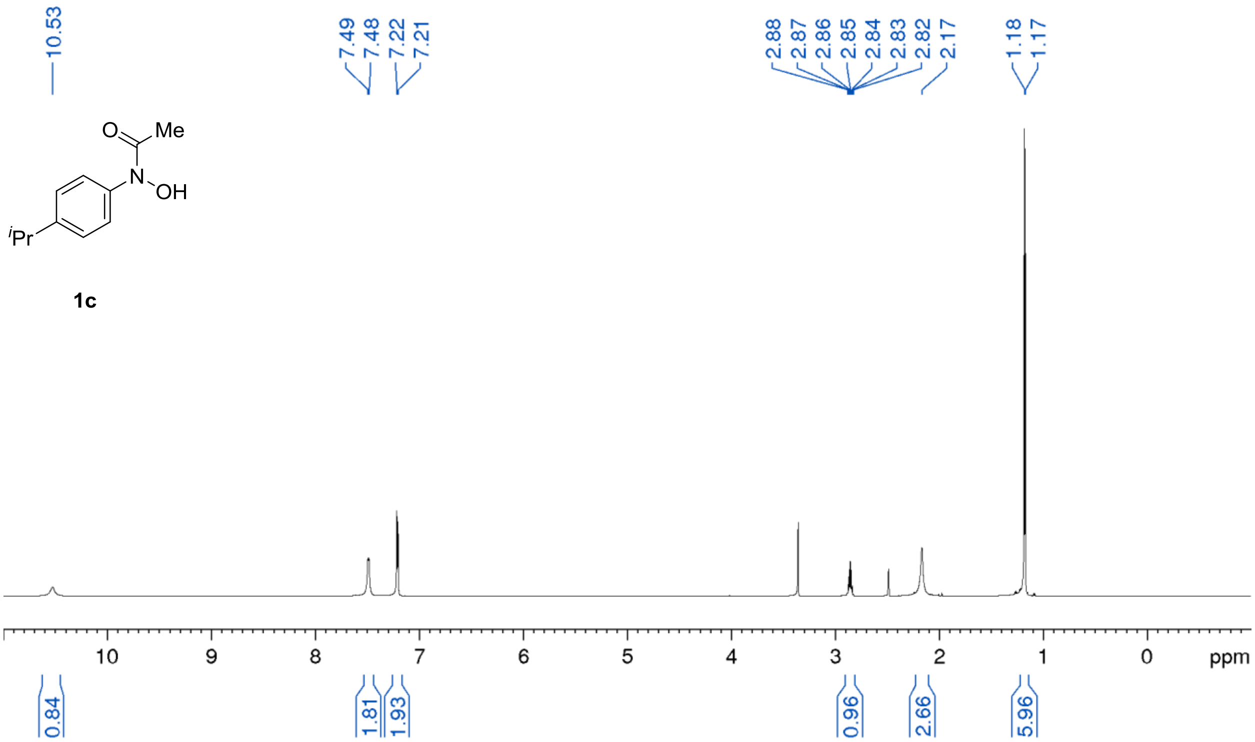

^{13}C NMR (175 MHz, $(CD_3)_2SO$, 25 ºC) of (1c)

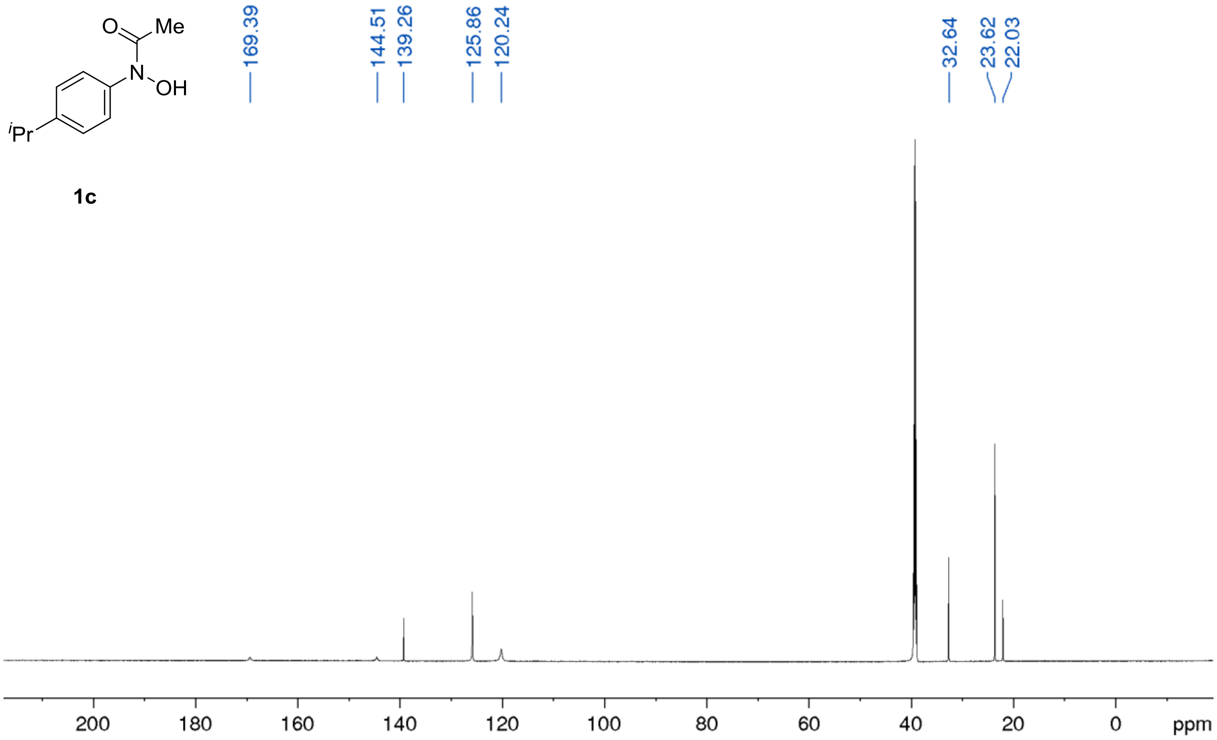

^{1}H NMR (700 MHz, $(CD_3)_2SO$, 25 ºC) of (1d)

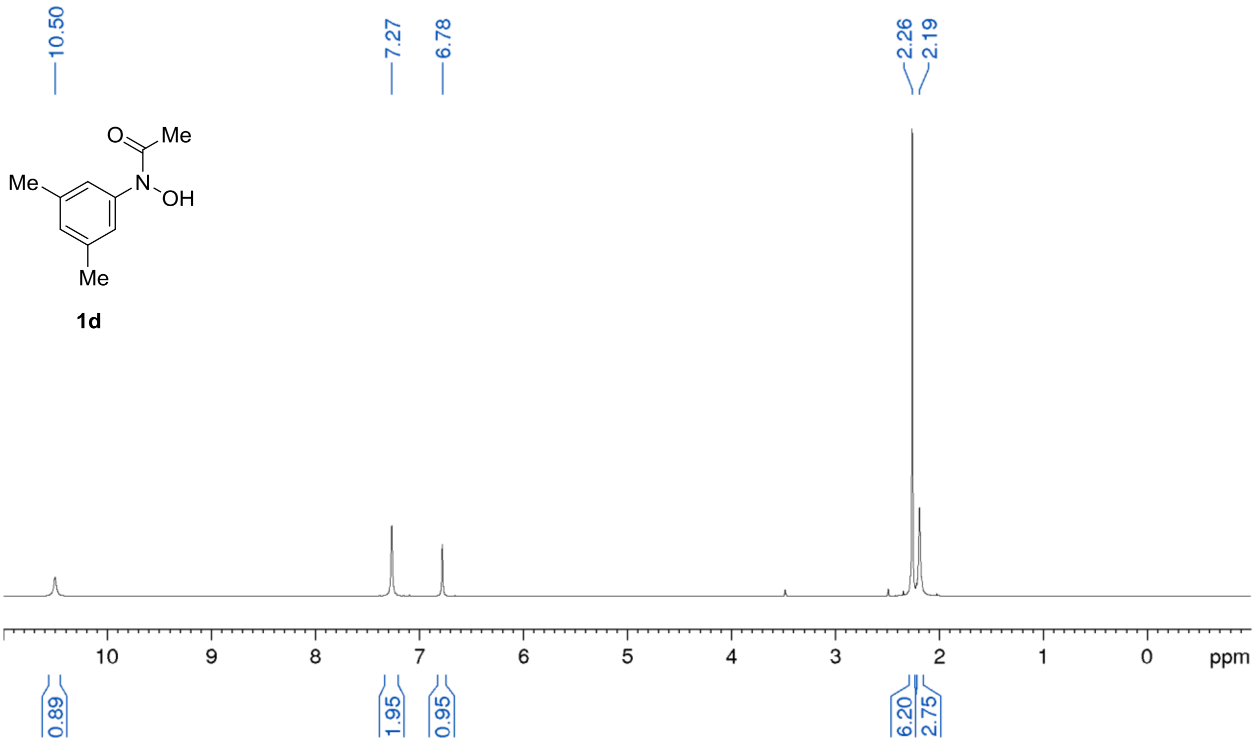

^{13}C NMR (175 MHz, $(CD_3)_2SO$, 25 ºC) of (1d)

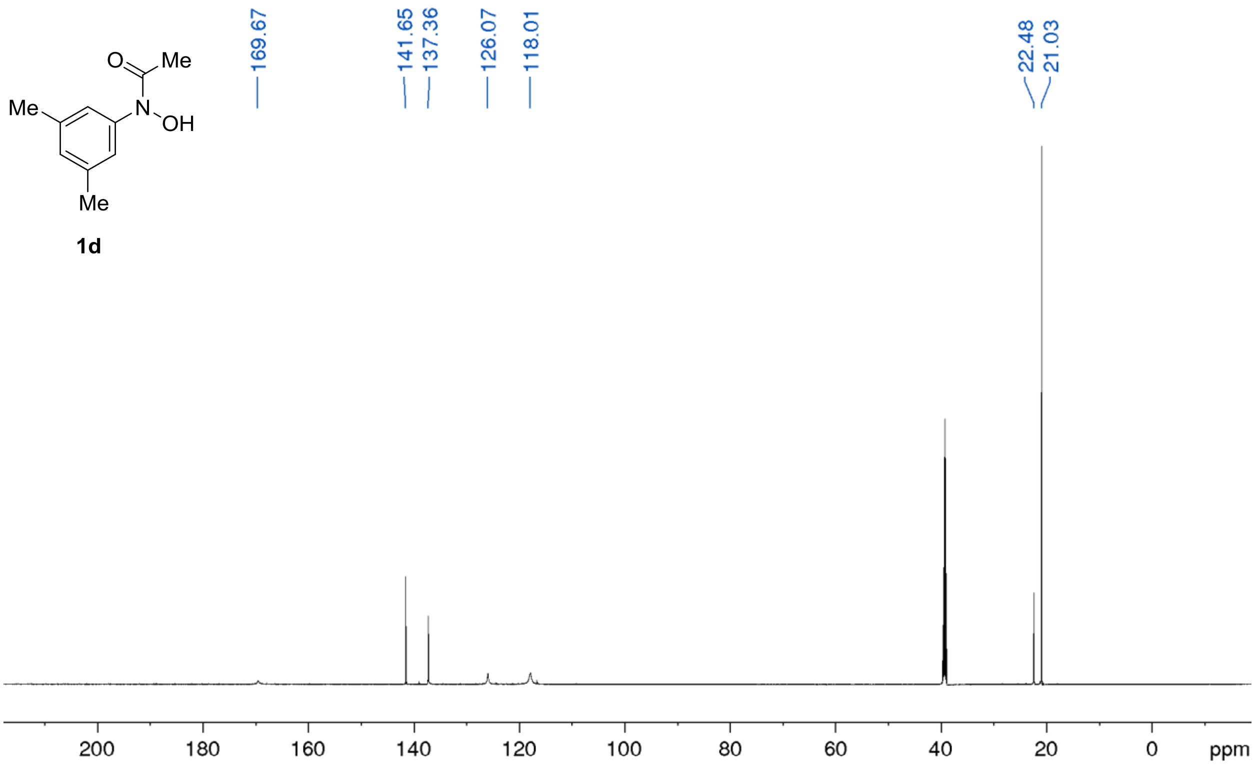

^{1}H NMR (700 MHz, $(CD_3)_2SO$, 25 ºC) of (1e)

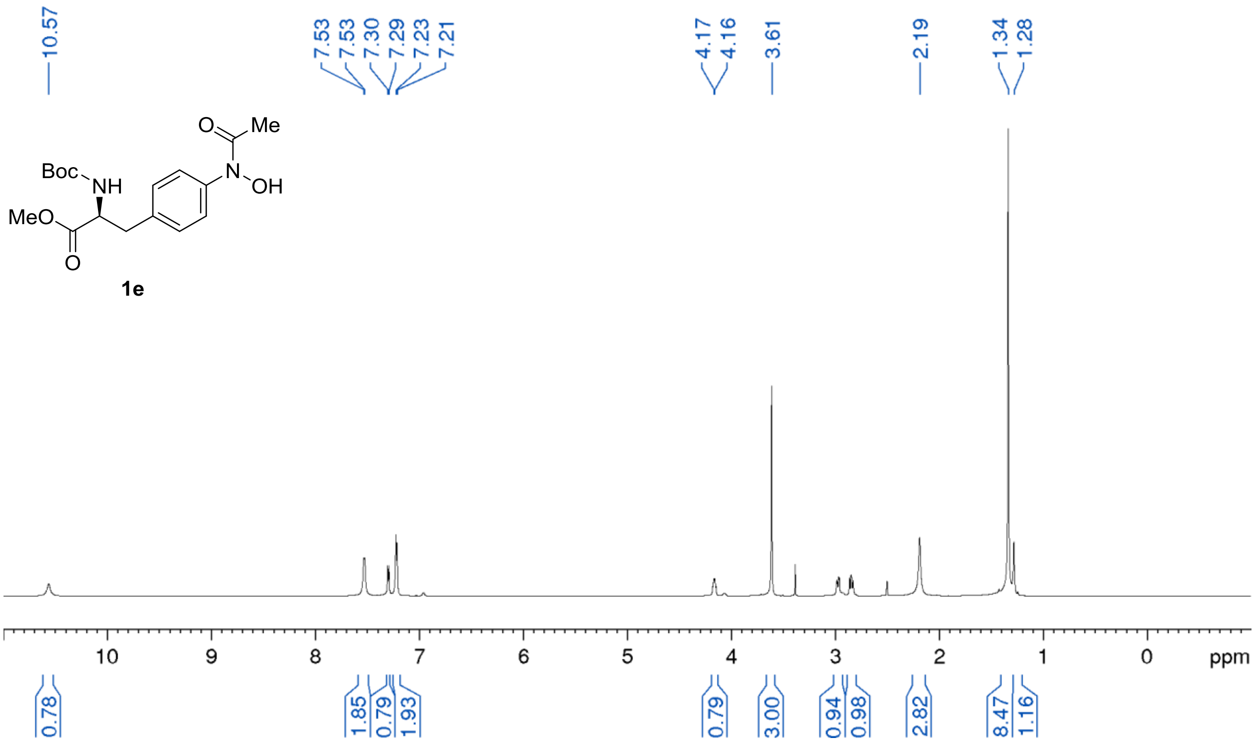

^{13}C NMR (175 MHz, $(CD_3)_2SO$, 25 ºC) of (1e)

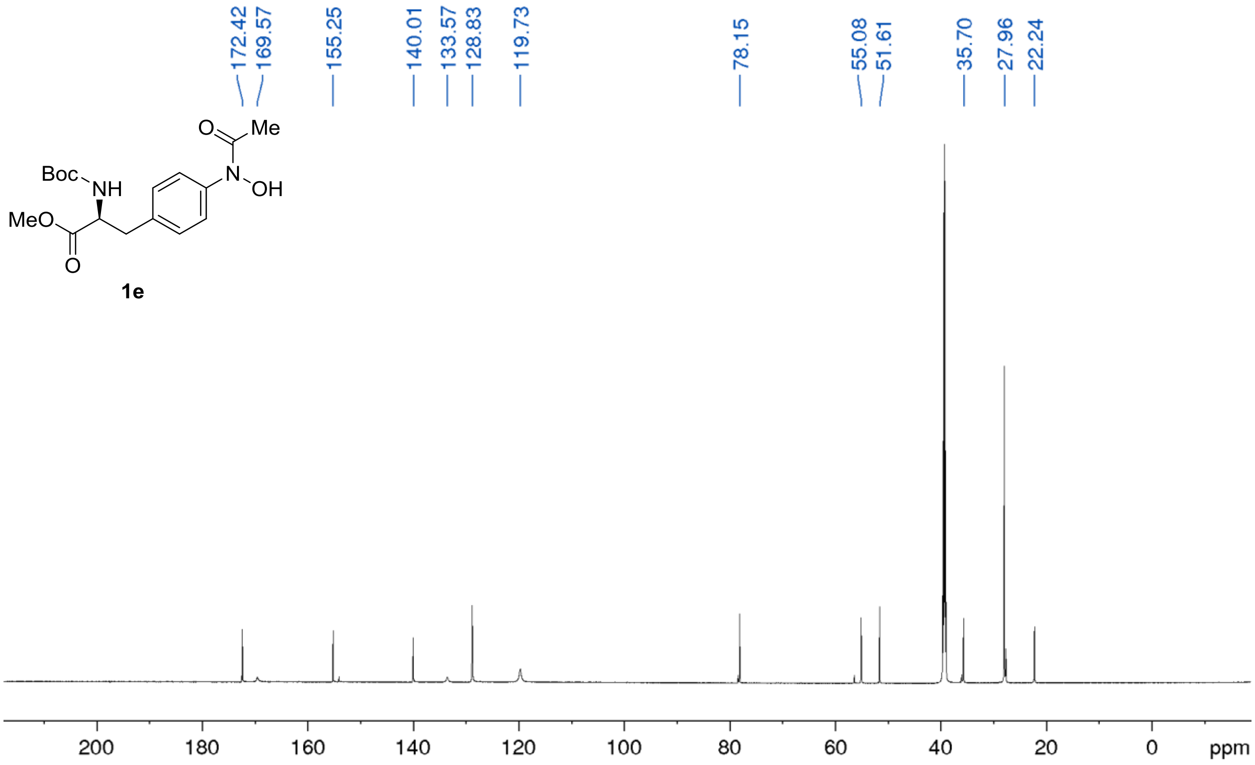

^{1}H NMR (700 MHz, $(CD_3)_2SO$, 25 ºC) of (1j)

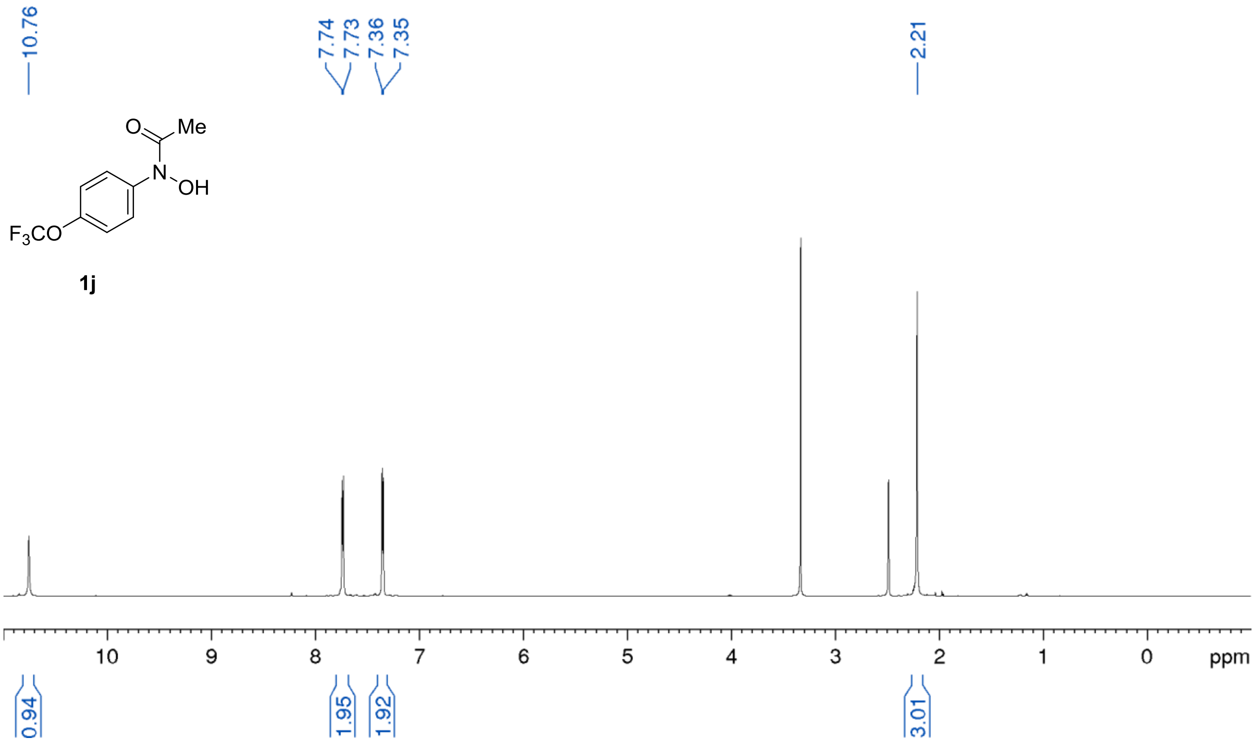

^{13}C NMR (175 MHz, $(CD_3)_2SO$, 25 ºC) of (1j)

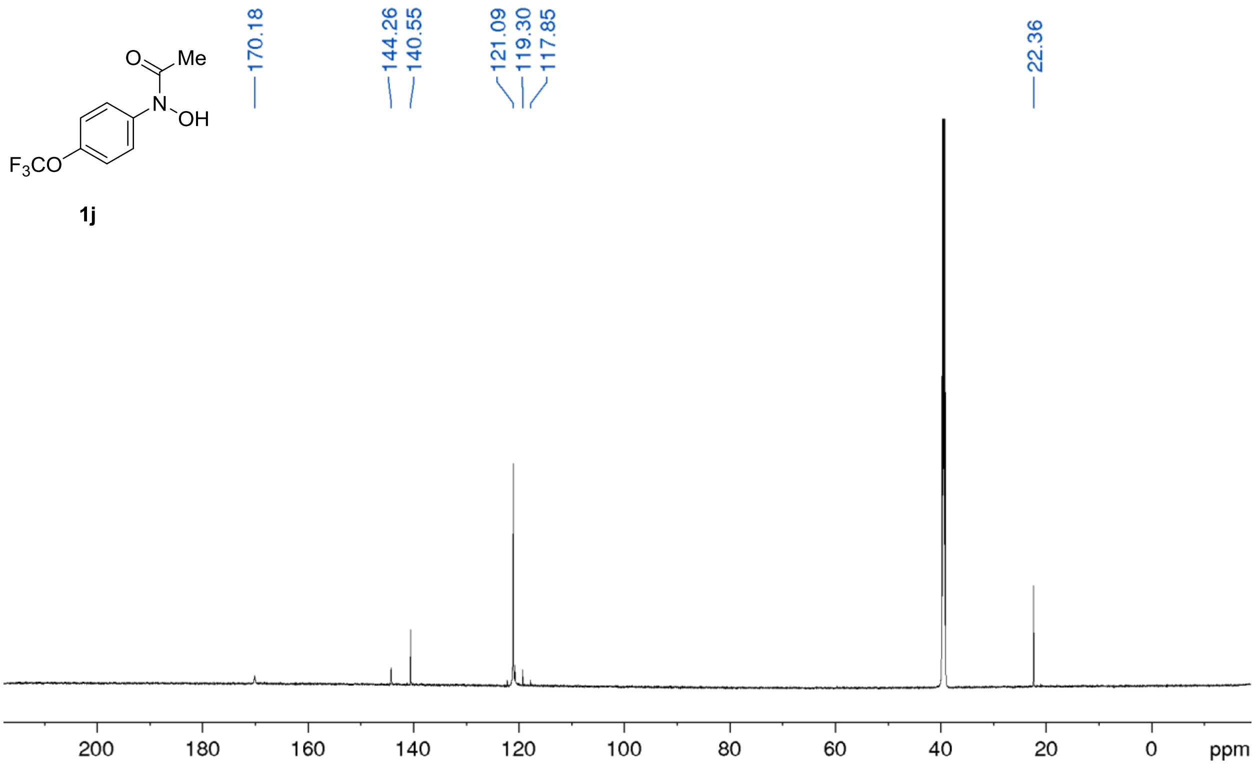

^{19}F NMR (376 MHz, $(CD_3)_2SO$, 25 ºC) of (1j)

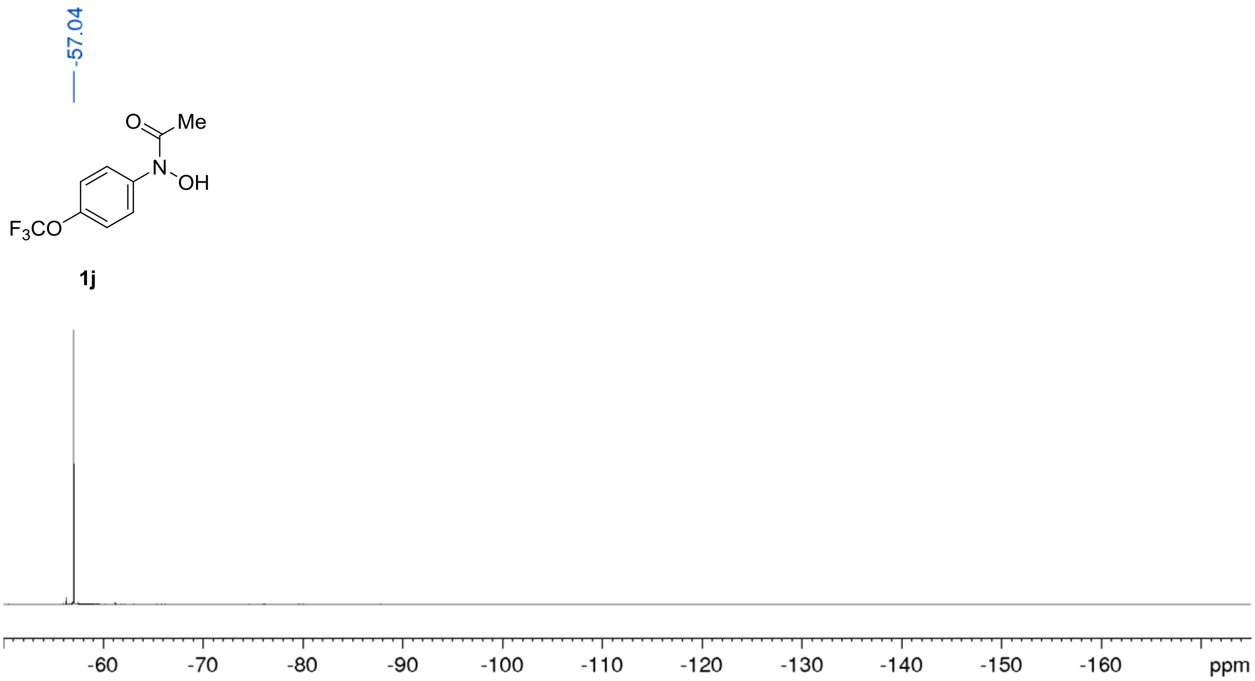

^{1}H NMR (700 MHz, $(CD_3)_2SO$, 25 ºC) of (1k)

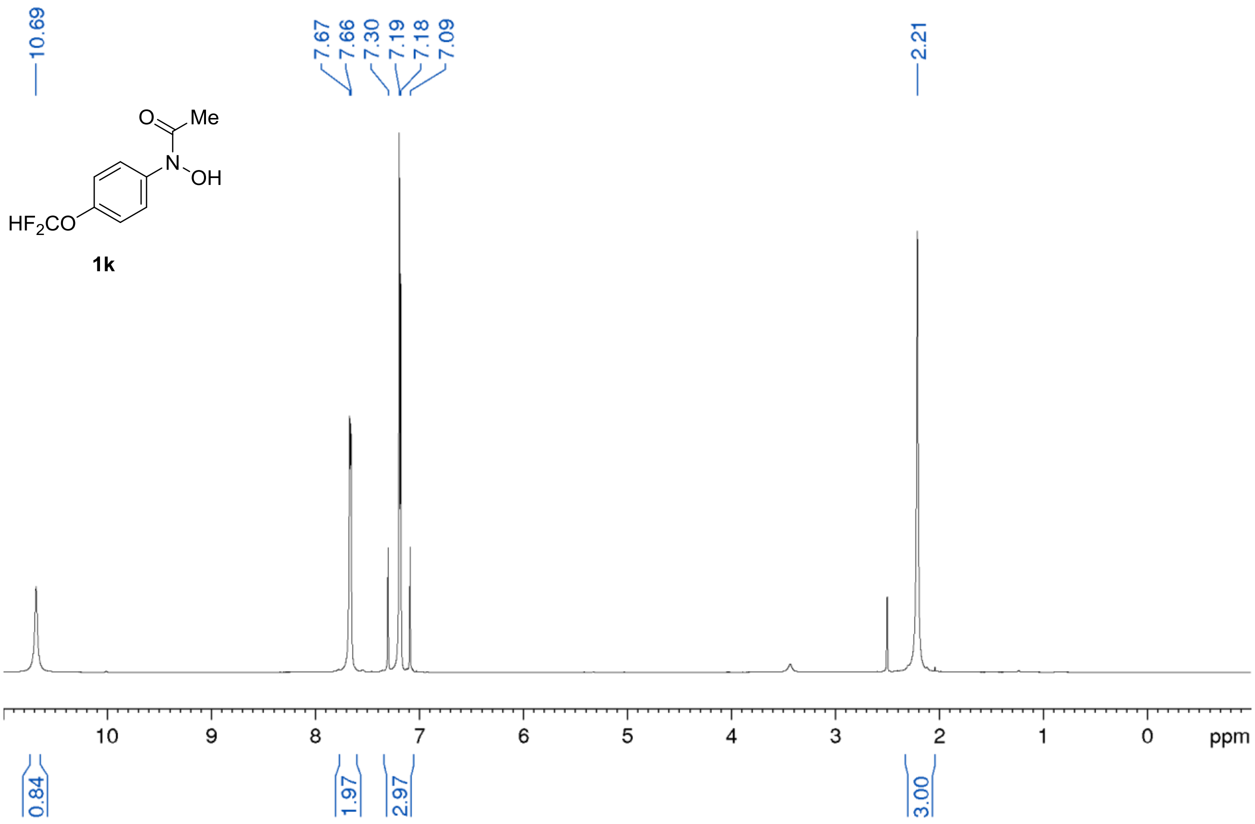

^{13}C NMR (175 MHz, $(CD_3)_2SO$, 25 ºC) of (1k)

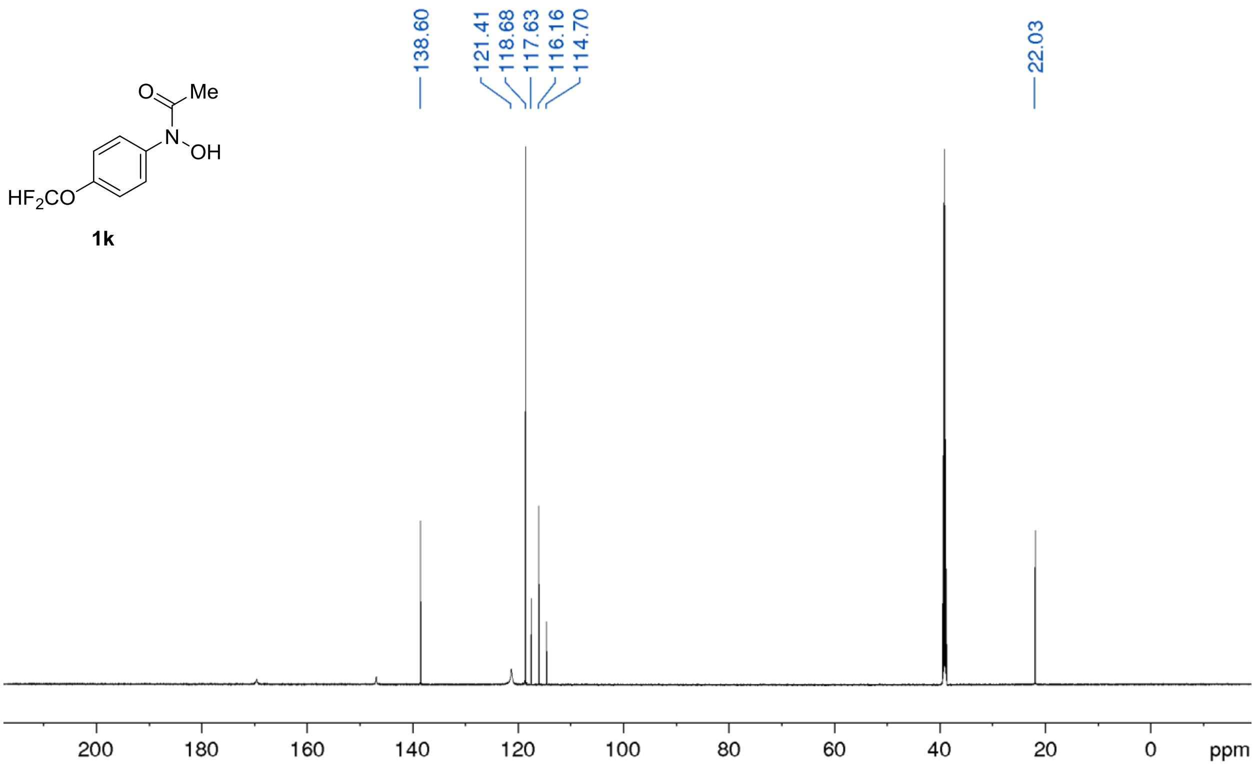

^{19}F NMR (376 MHz, $(CD_3)_2SO$, 25 ºC) of (1k)

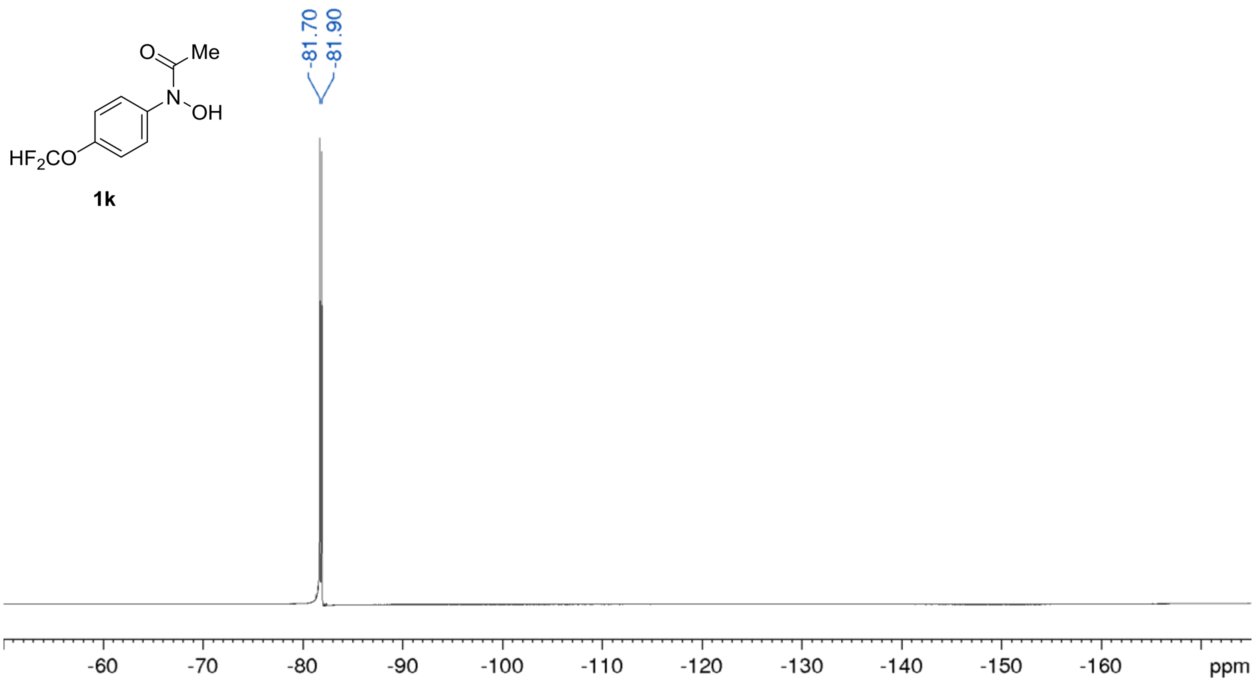

^{1}H NMR (700 MHz, $(CD_3)_2SO$, 25 ºC) of (1l)

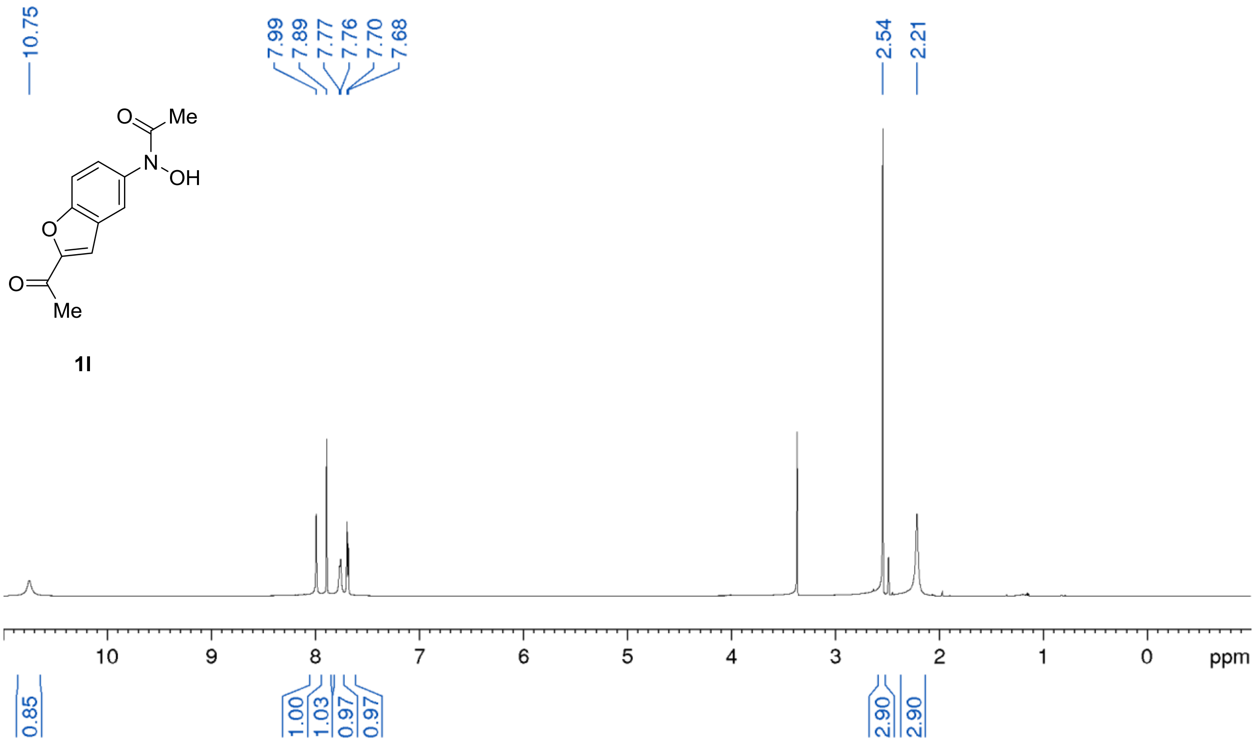

^{13}C NMR (175 MHz, $(CD_3)_2SO$, 25 ºC) of (1l)

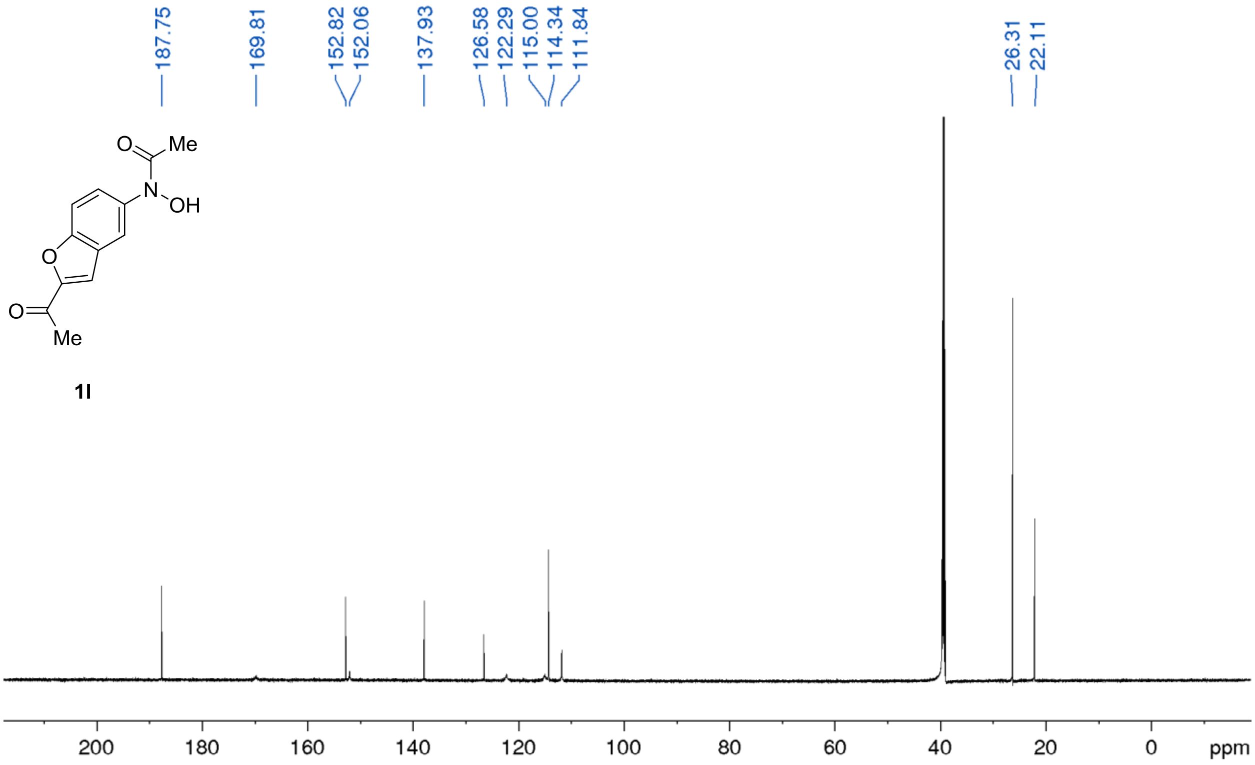

^{1}H NMR (700 MHz, $(CD_3)_2SO$, 25 ºC) of (1m)

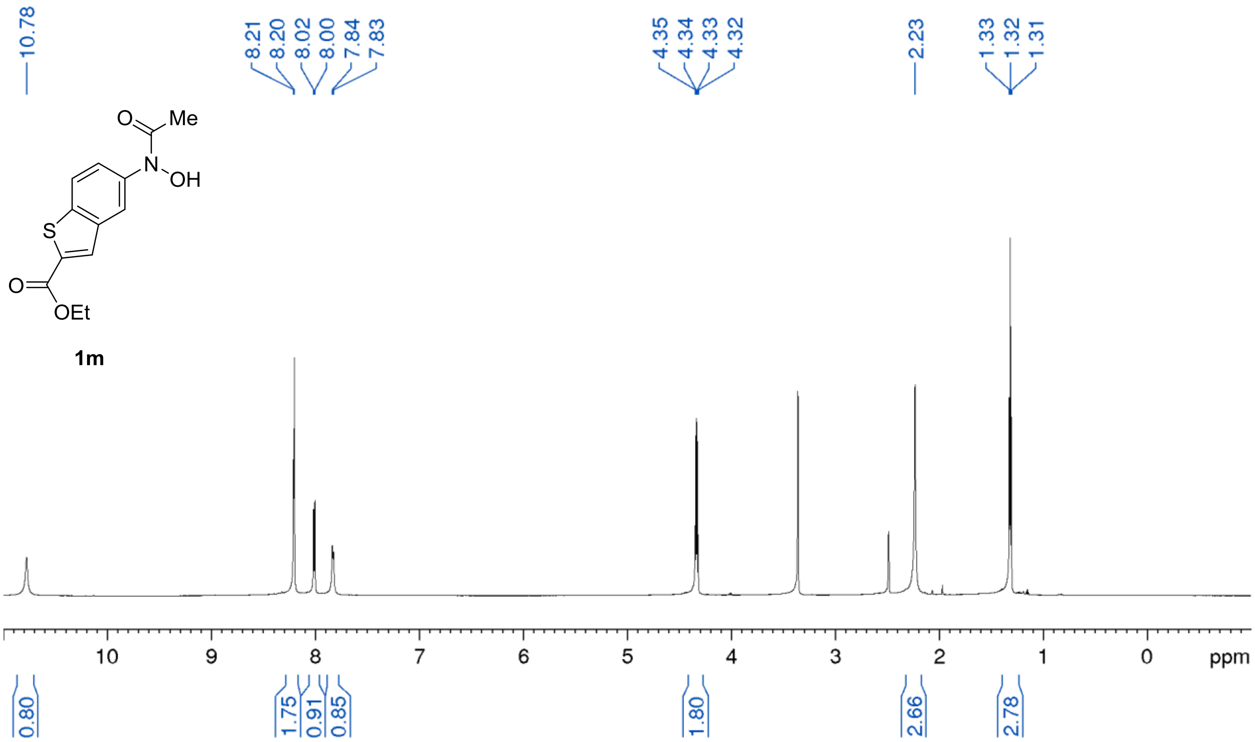

^{13}C NMR (175 MHz, $(CD_3)_2SO$, 25 ºC) of (1m)

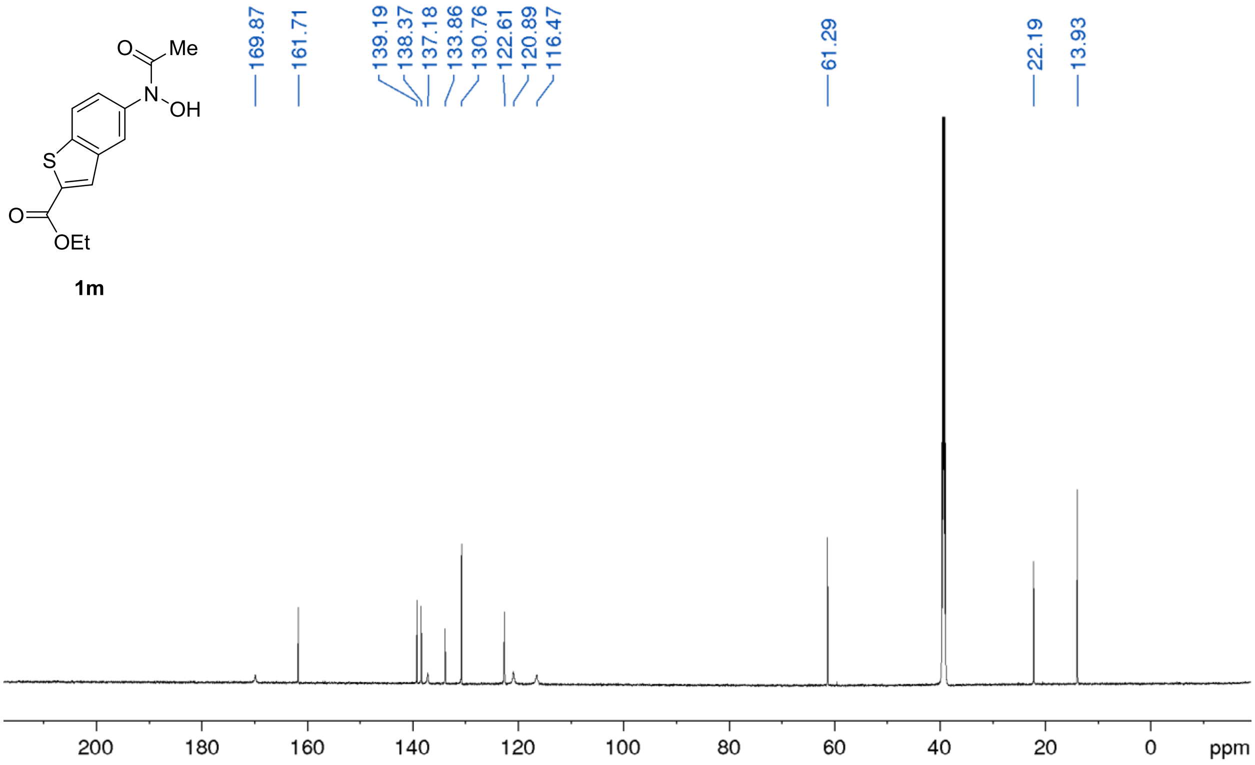

^{1}H NMR (700 MHz, $(CD_3)_2SO$, 25 °C) of (1n)

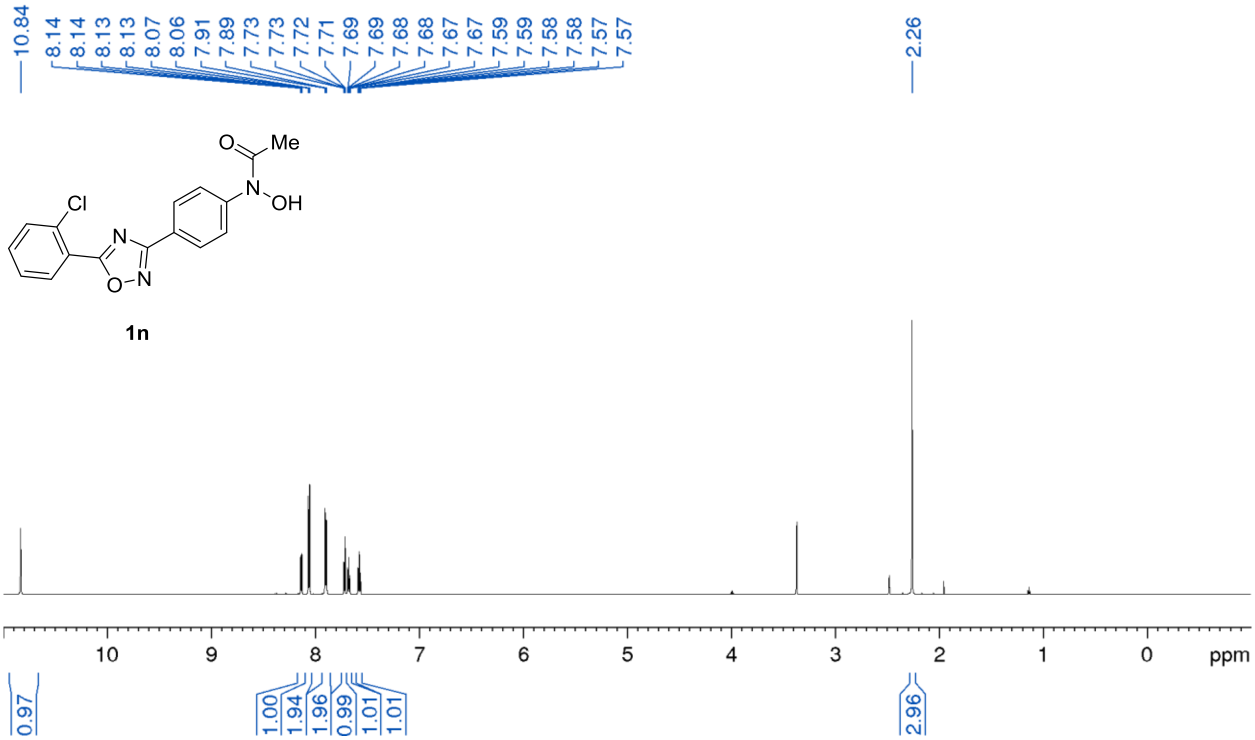

^{13}C NMR (175 MHz, $(CD_3)_2SO$, 25 °C) of (1n)

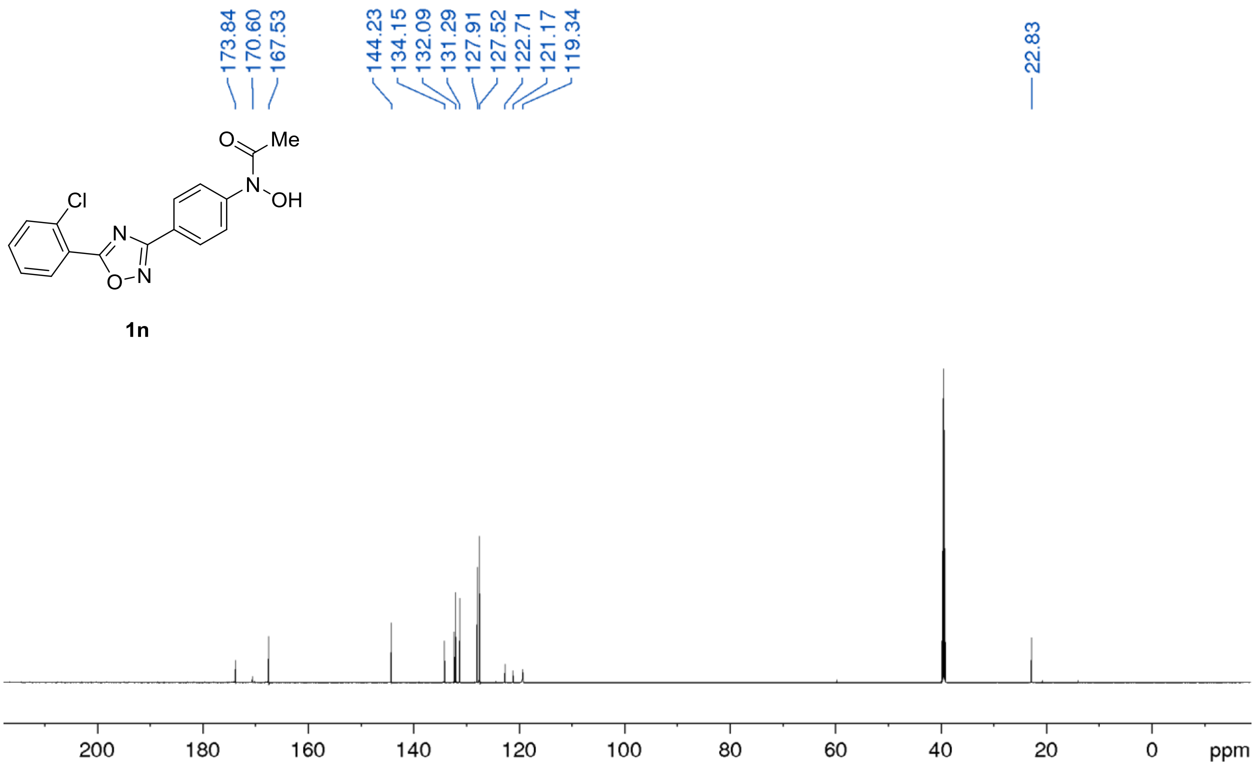

^{1}H NMR (700 MHz, $(CD_3)_2SO$, 25 °C) of (S1)

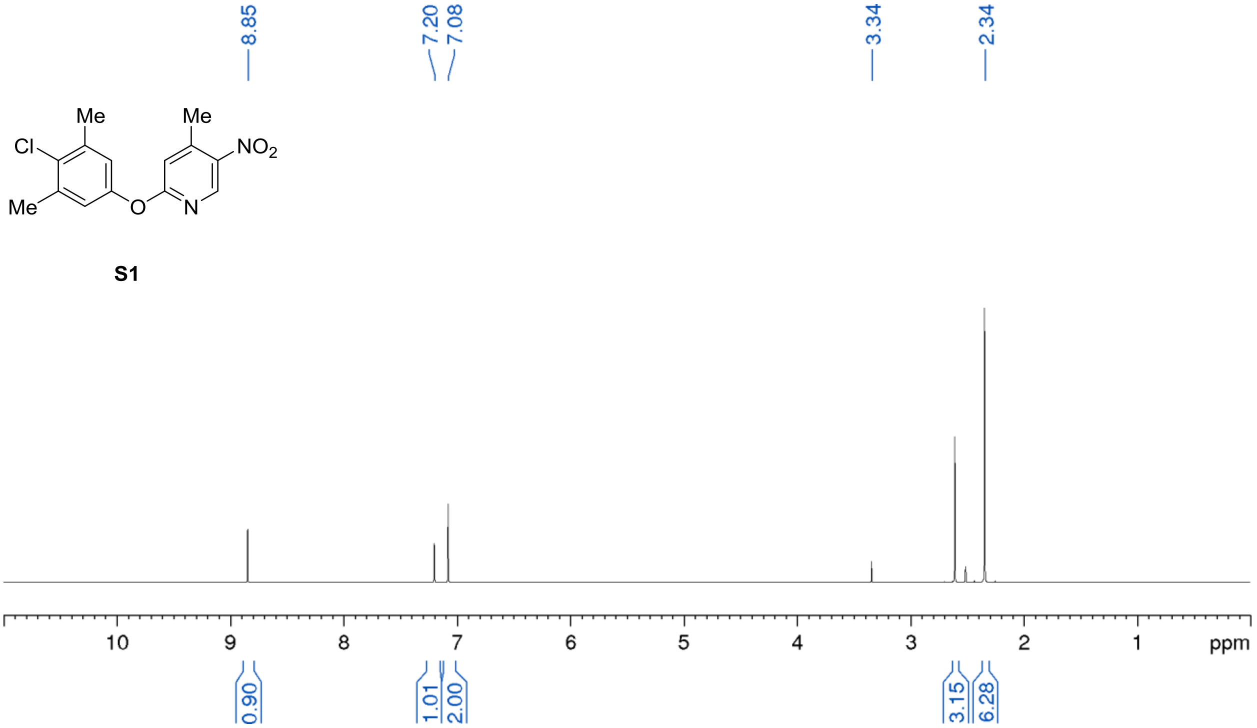

^{13}C NMR (175 MHz, $(CD_3)_2SO$, 25 °C) of (S1)

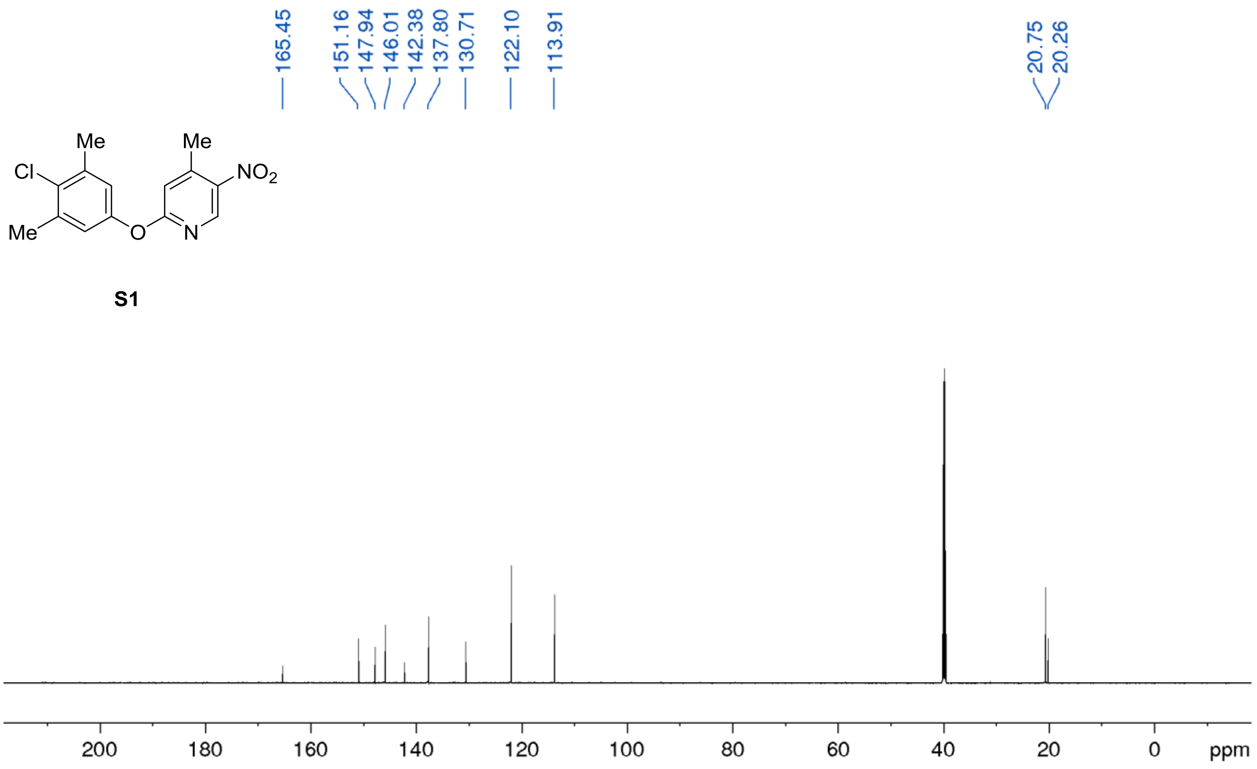

^{1}H NMR (700 MHz, $(CD_3)_2SO$, 25 ºC) of (1q)

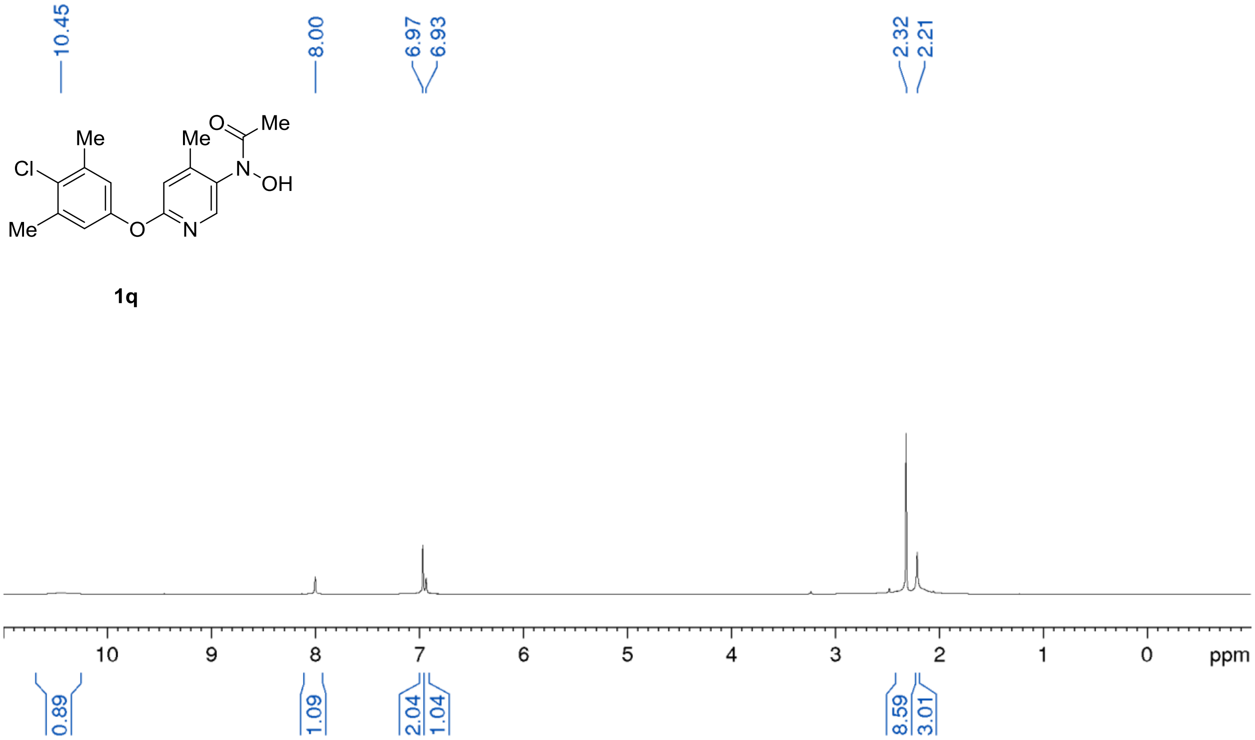

^{13}C NMR (175 MHz, $(CD_3)_2SO$, 25 ºC) of (1q)

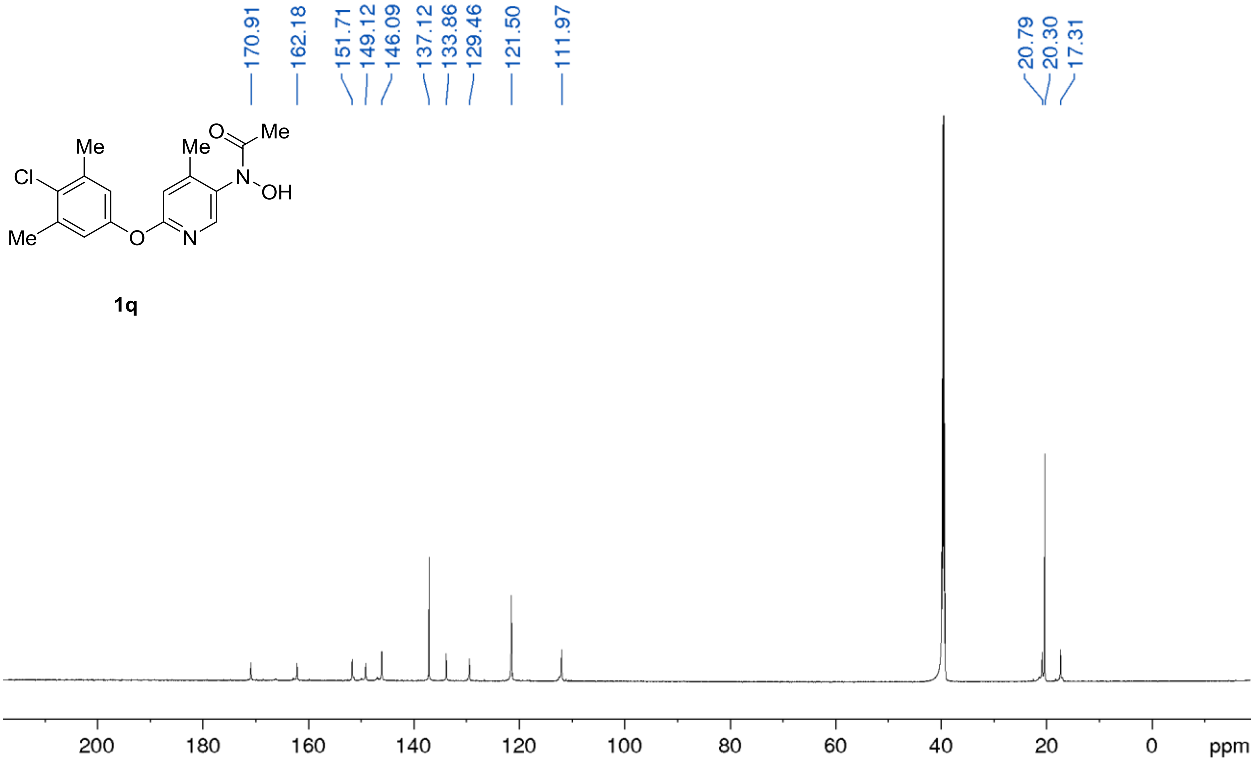

^{1}H NMR (700 MHz, $(CD_3)_2SO$, 25 ºC) of (S2)

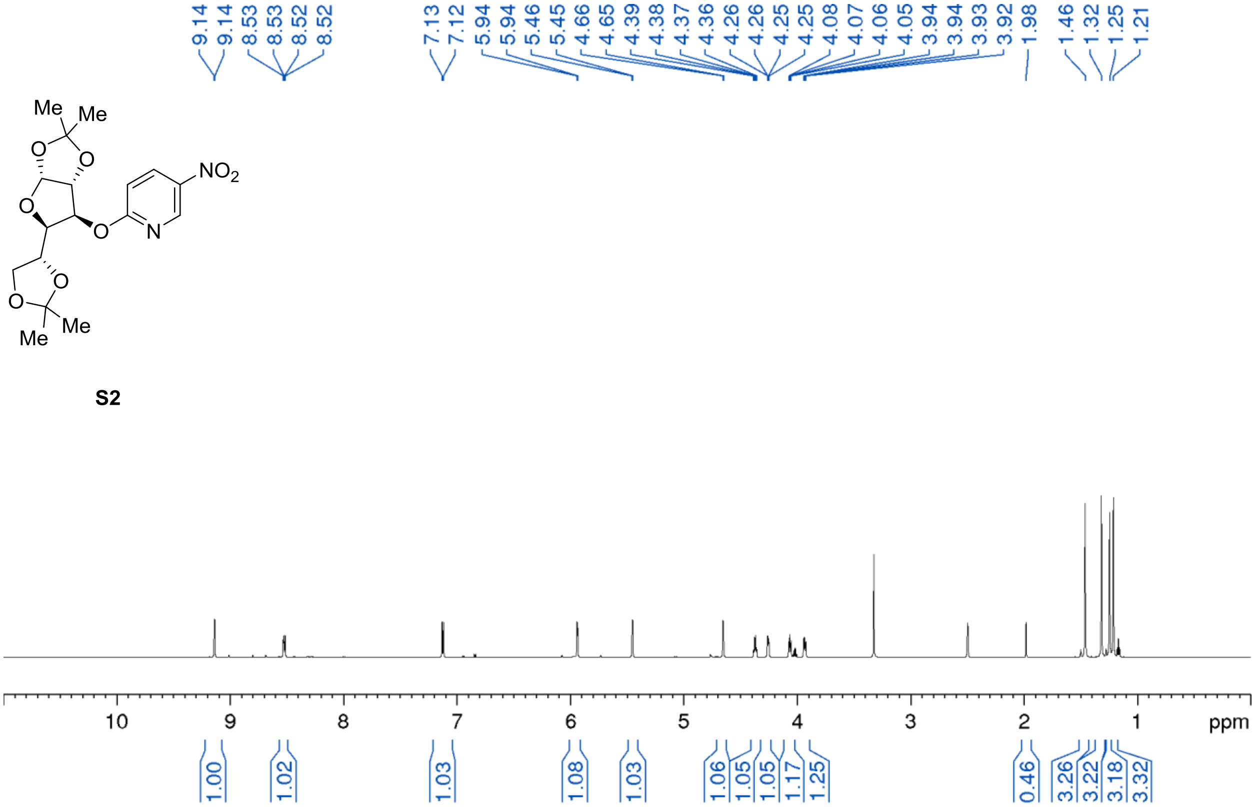

^{13}C NMR (175 MHz, $(CD_3)_2SO$, 25 ºC) of (S2)

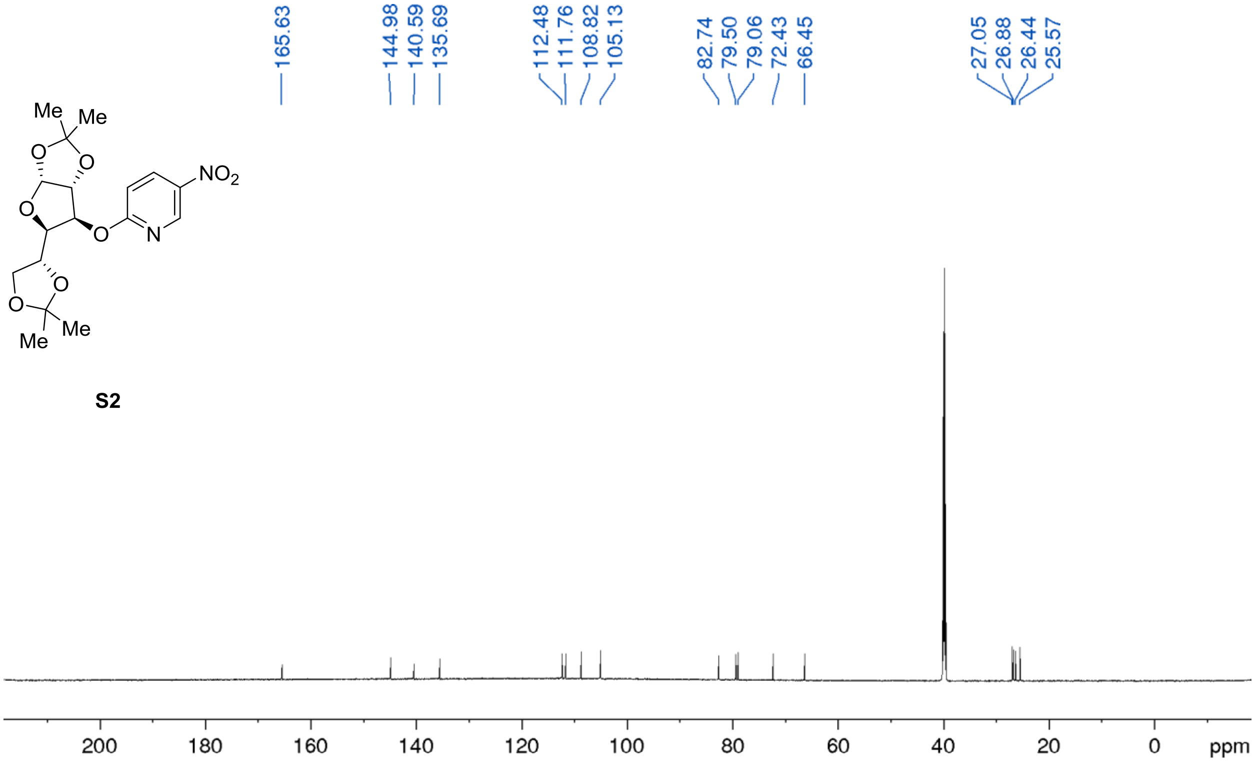

^{1}H NMR (700 MHz, $(CD_3)_2SO$, 25 °C) of (1t)

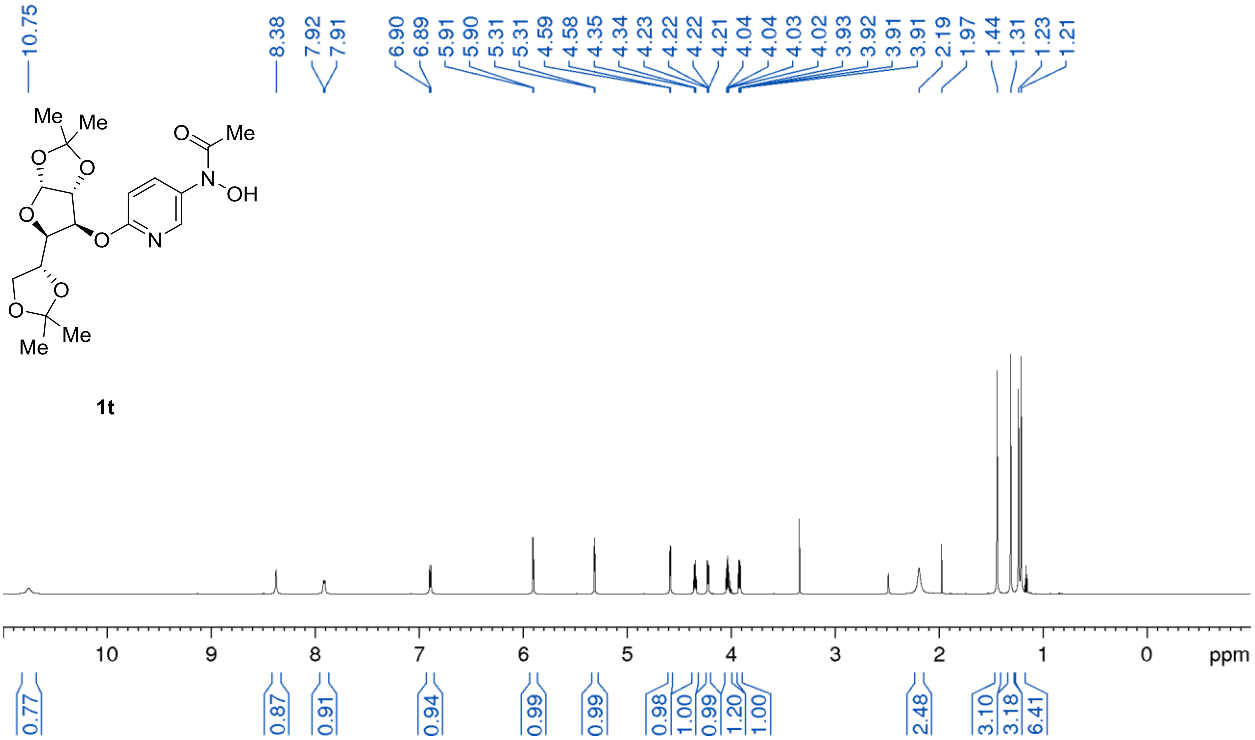

^{13}C NMR (175 MHz, $(CD_3)_2SO$, 25 °C) of (1t)

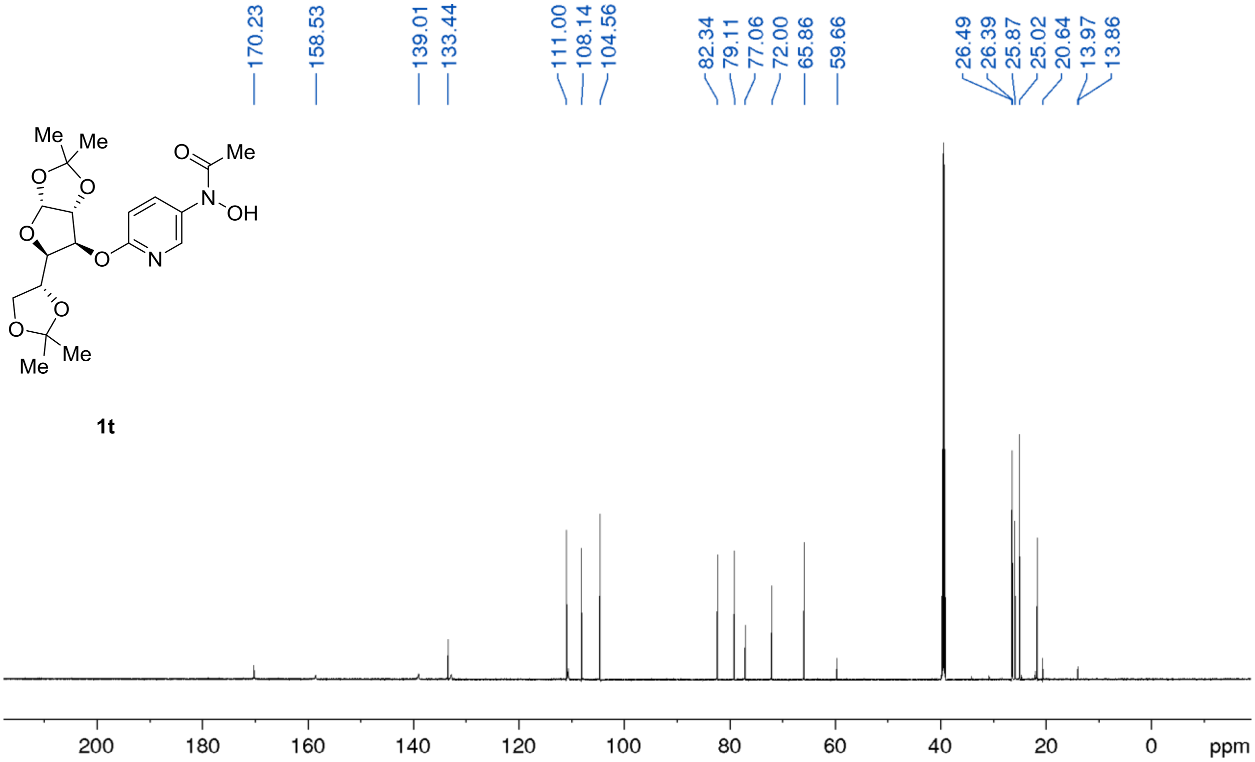

^{1}H NMR (700 MHz, $CDCl_3$, 25 ºC) of (3a)

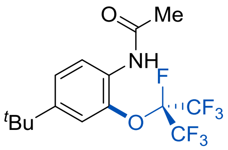

3a

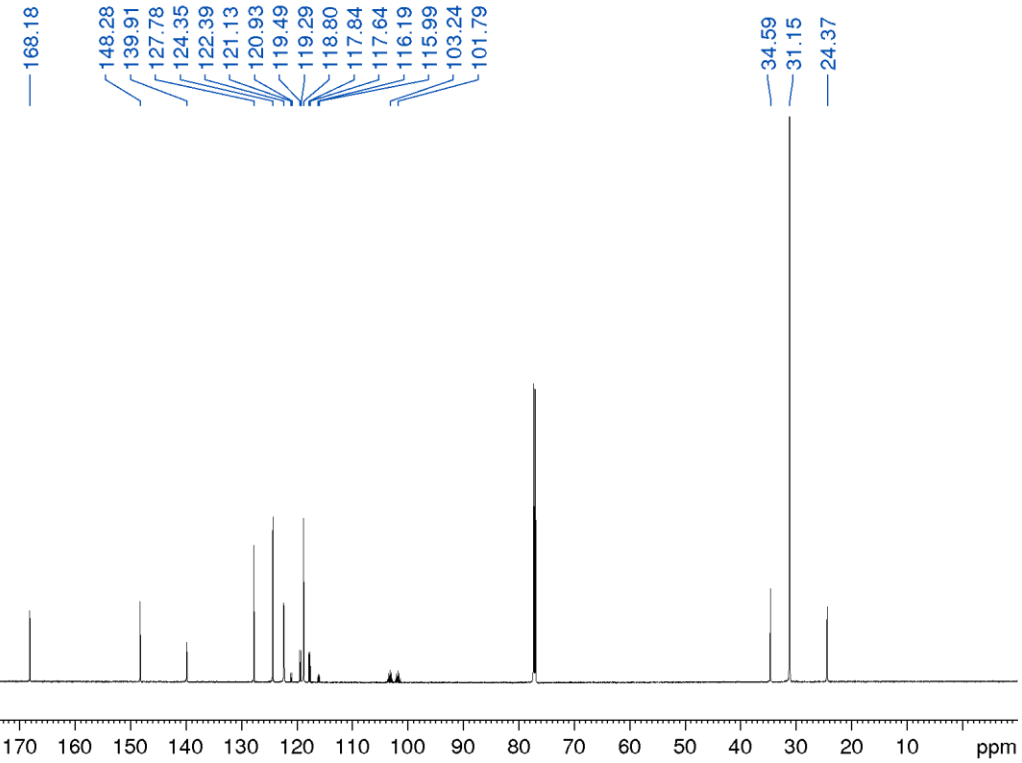

^{19}F NMR (376 MHz, $CDCl_3$, 25 ºC) of (3a)

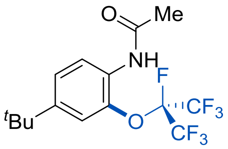

3a

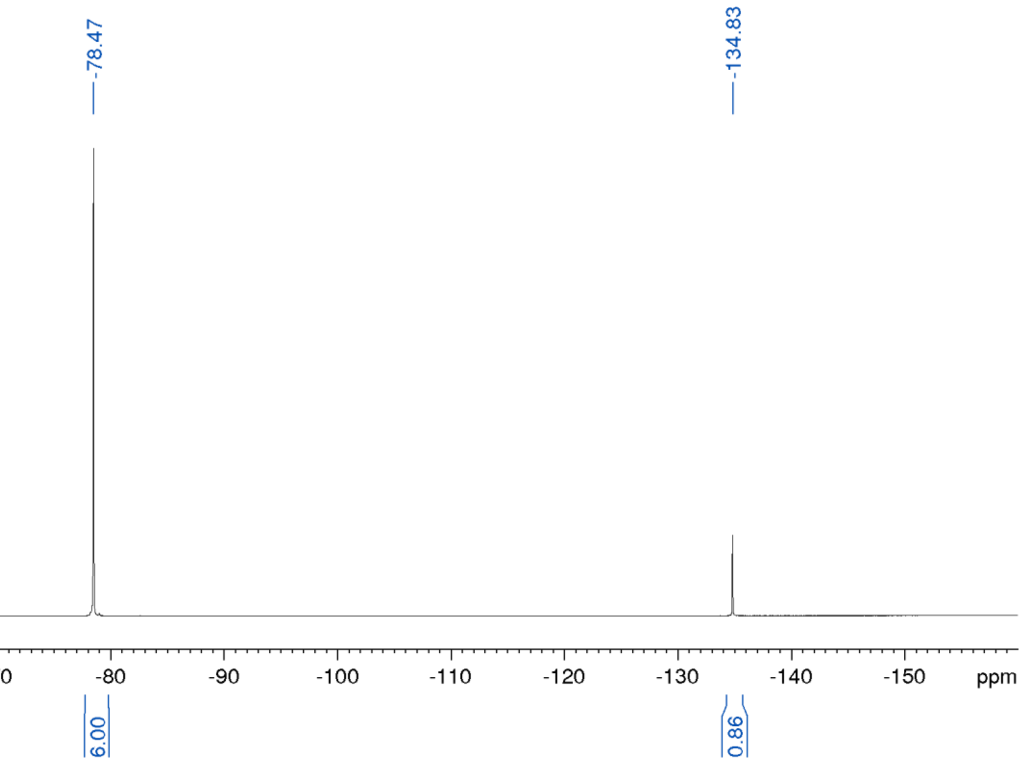

^{13}C NMR (175 MHz, $CDCl_3$, 25 ºC) of (3a)

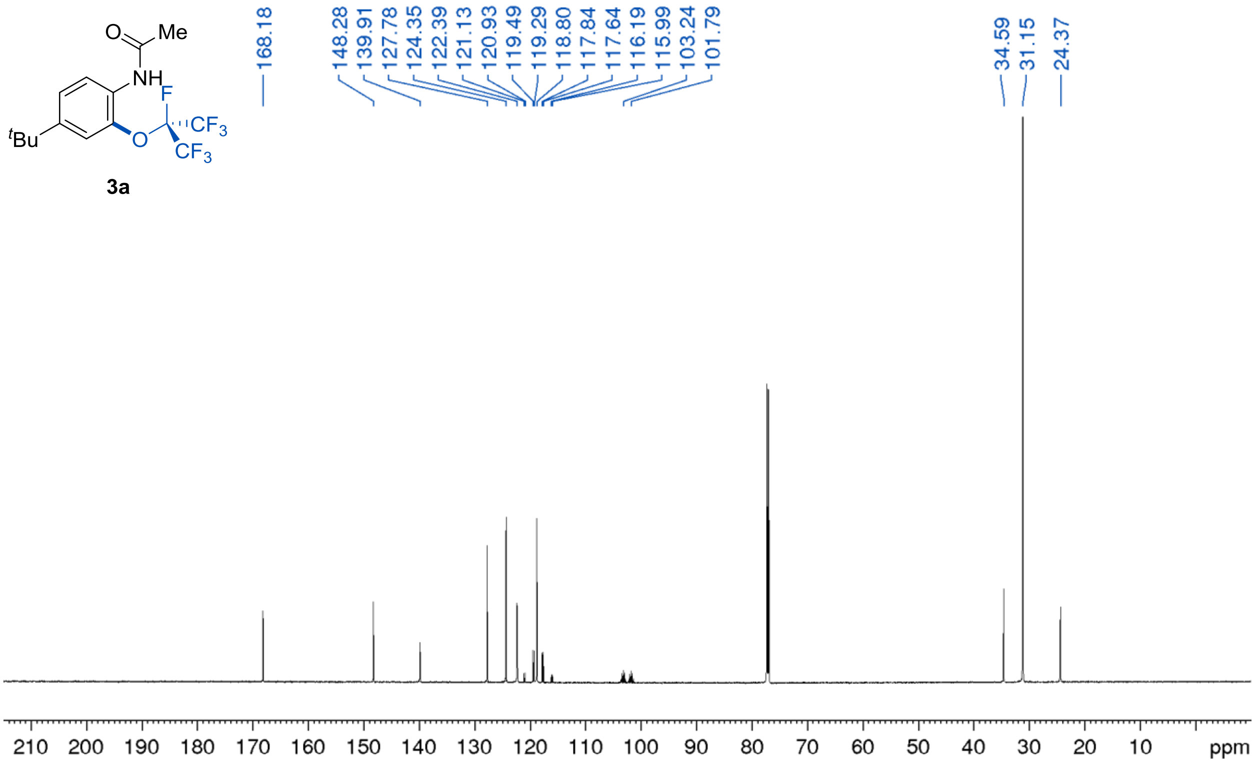

^{1}H NMR (700 MHz, $CDCl_3$, 25 ºC) of (3b)

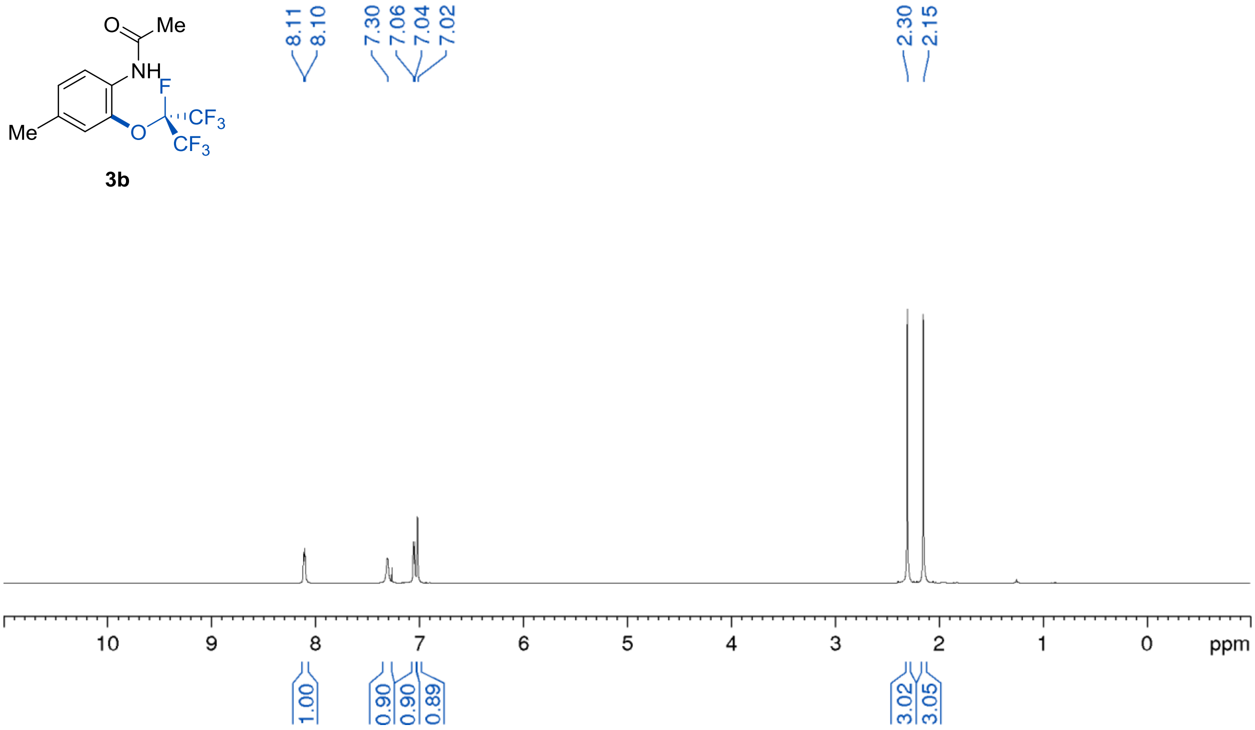

^{13}C NMR (175 MHz, $CDCl_3$, 25 ºC) of (3b)

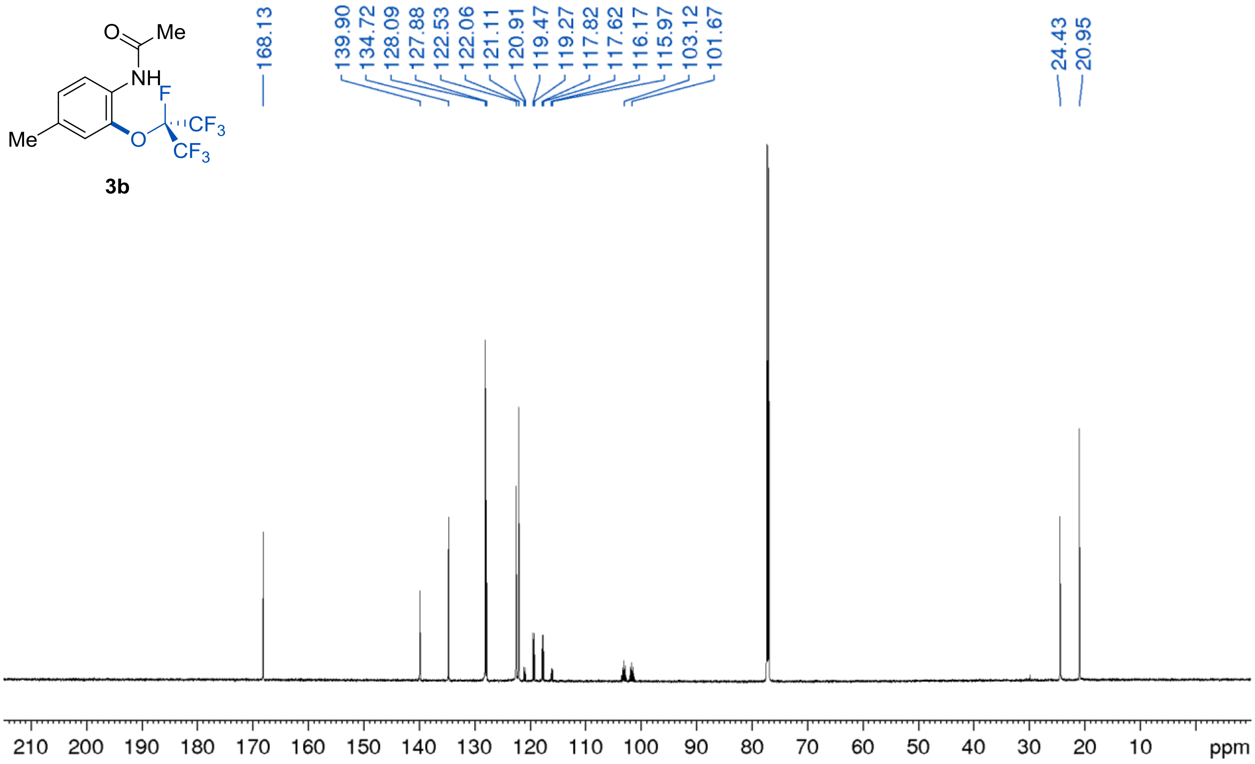

^{19}F NMR (376 MHz, CDCl3, 25 ºC) of (3b)

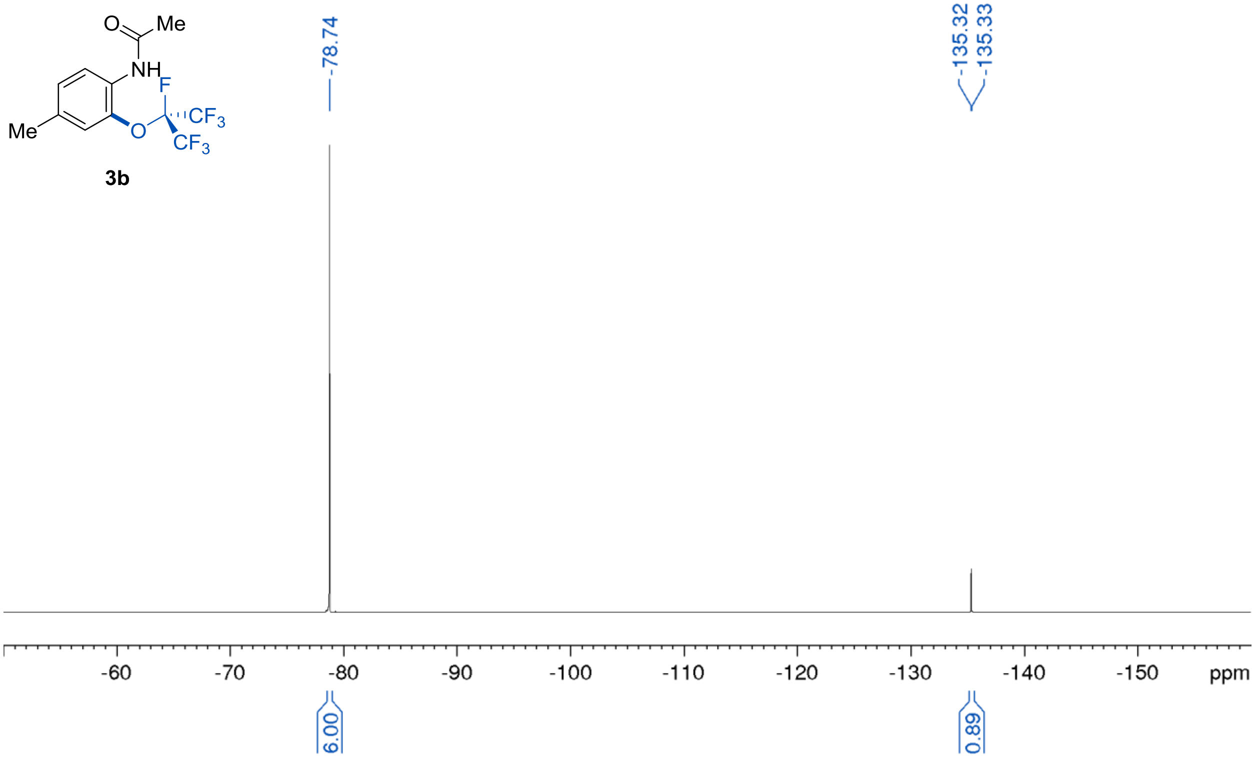

^{1}H NMR (700 MHz, $CDCl_3$, 25 ºC) of (3c)

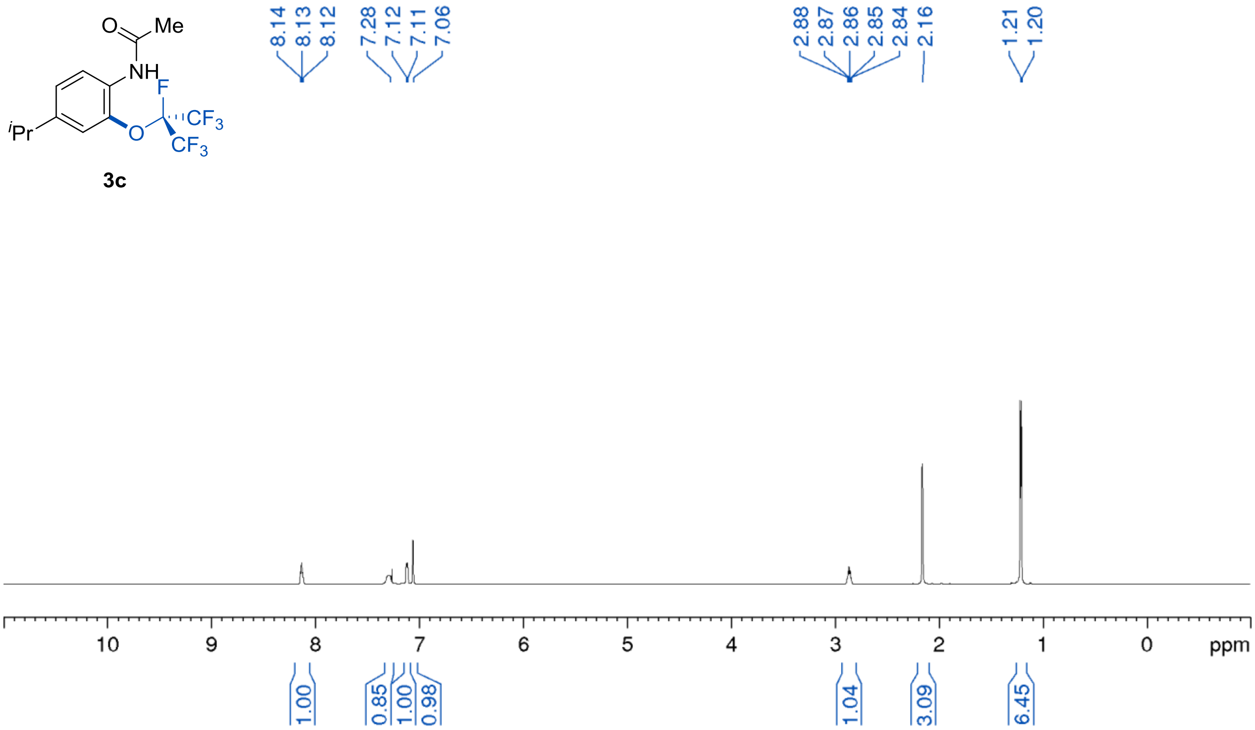

^{13}C NMR (175 MHz, $CDCl_3$, 25 ºC) of (3c)

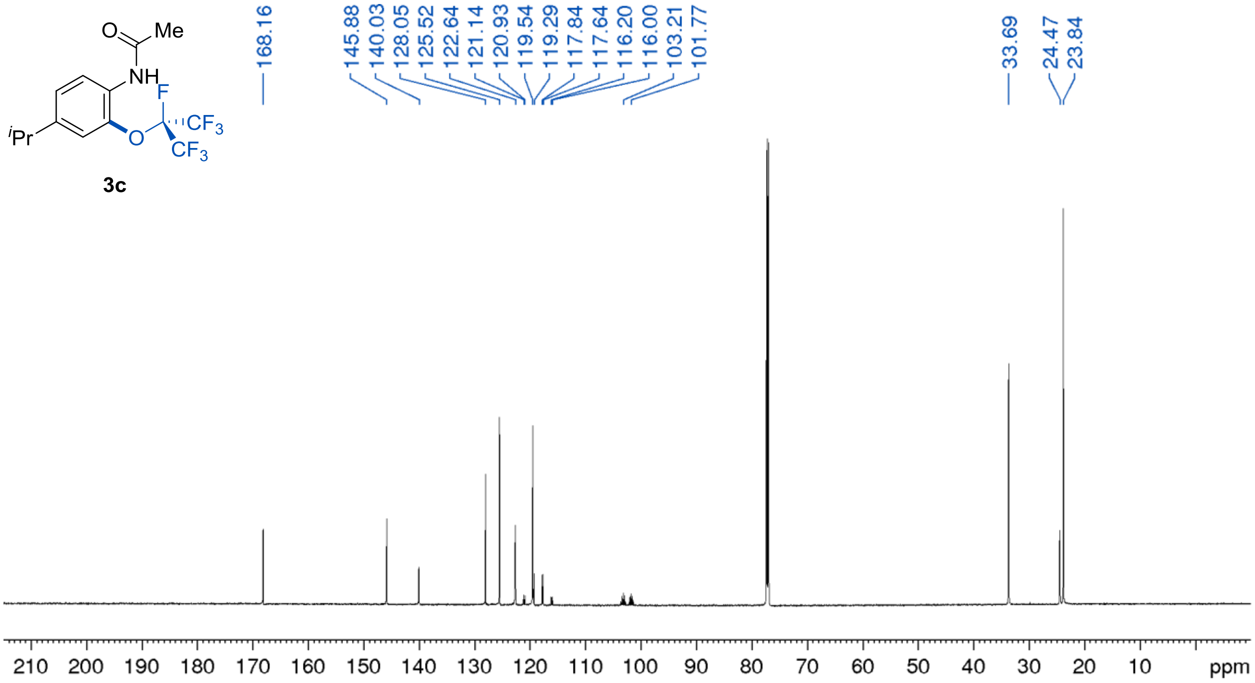

^{19}F NMR (376 MHz, $CDCl_3$, 25 ºC) of (3c)

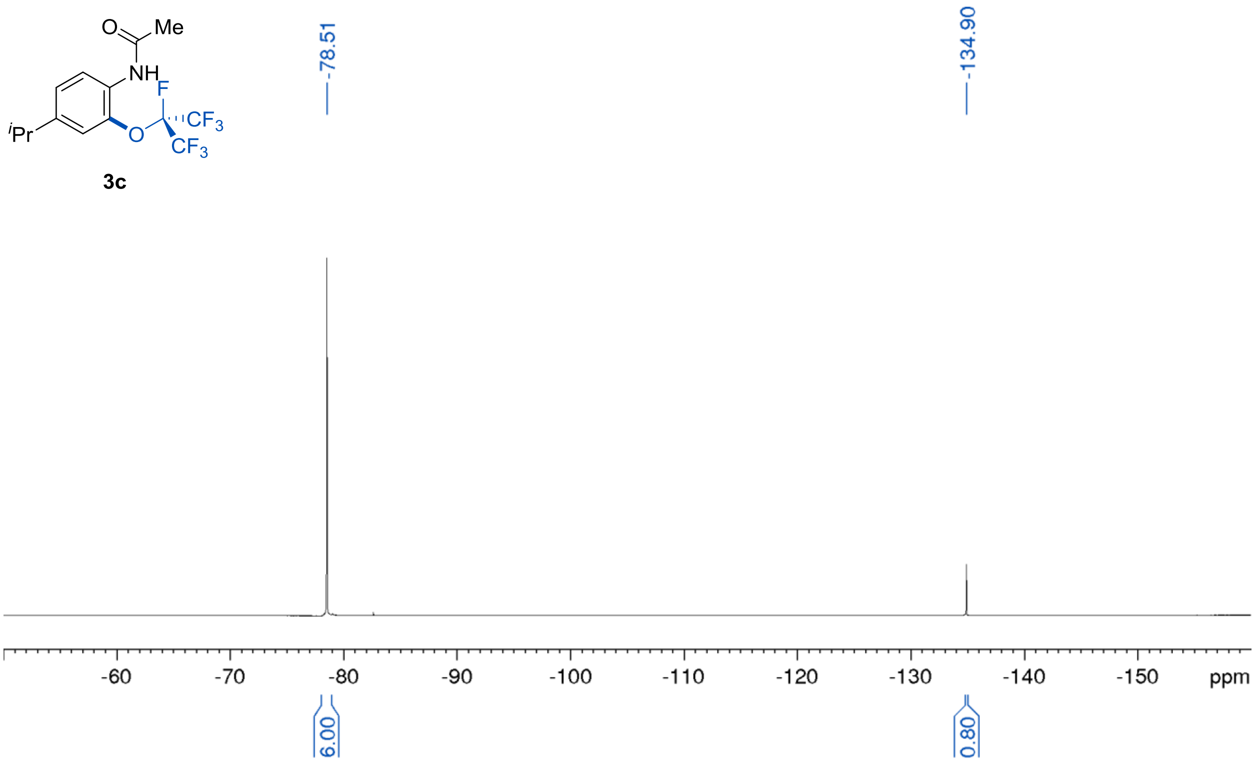

^{1}H NMR (700 MHz, $CDCl_3$, 25 ºC) of (3d)

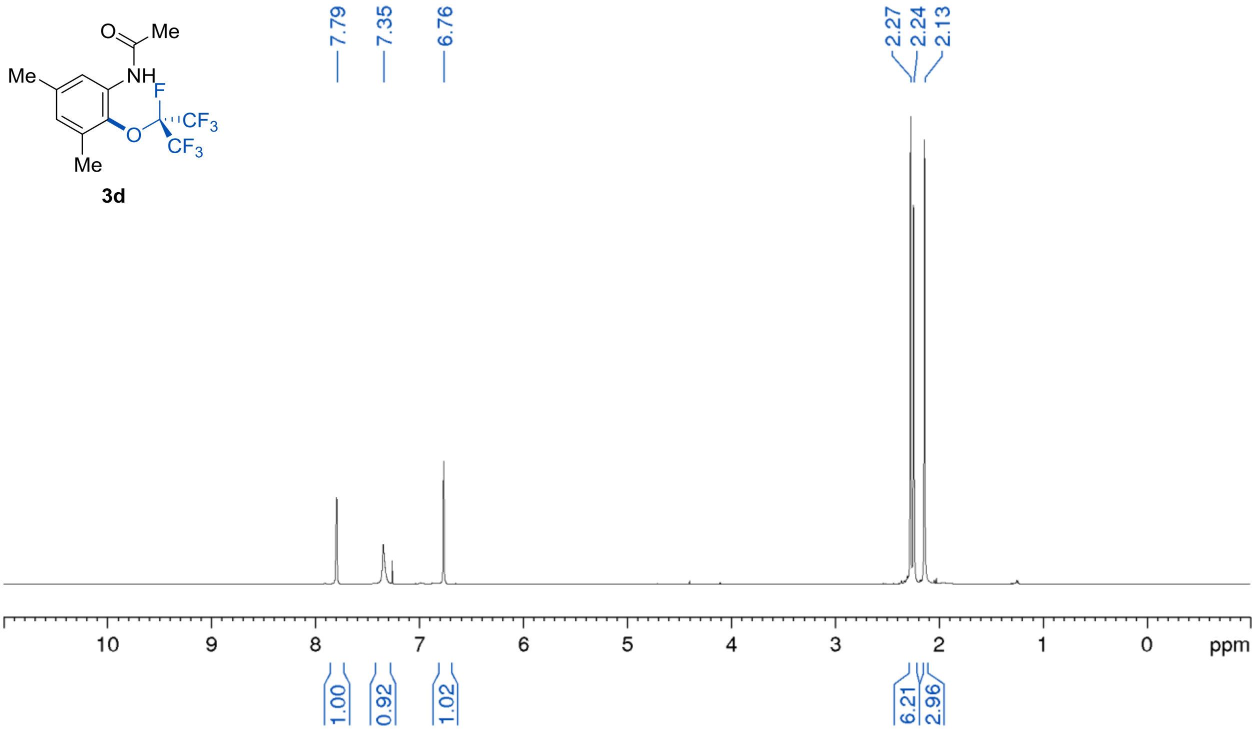

^{13}C NMR (175 MHz, $CDCl_3$, 25 ºC) of (3d)

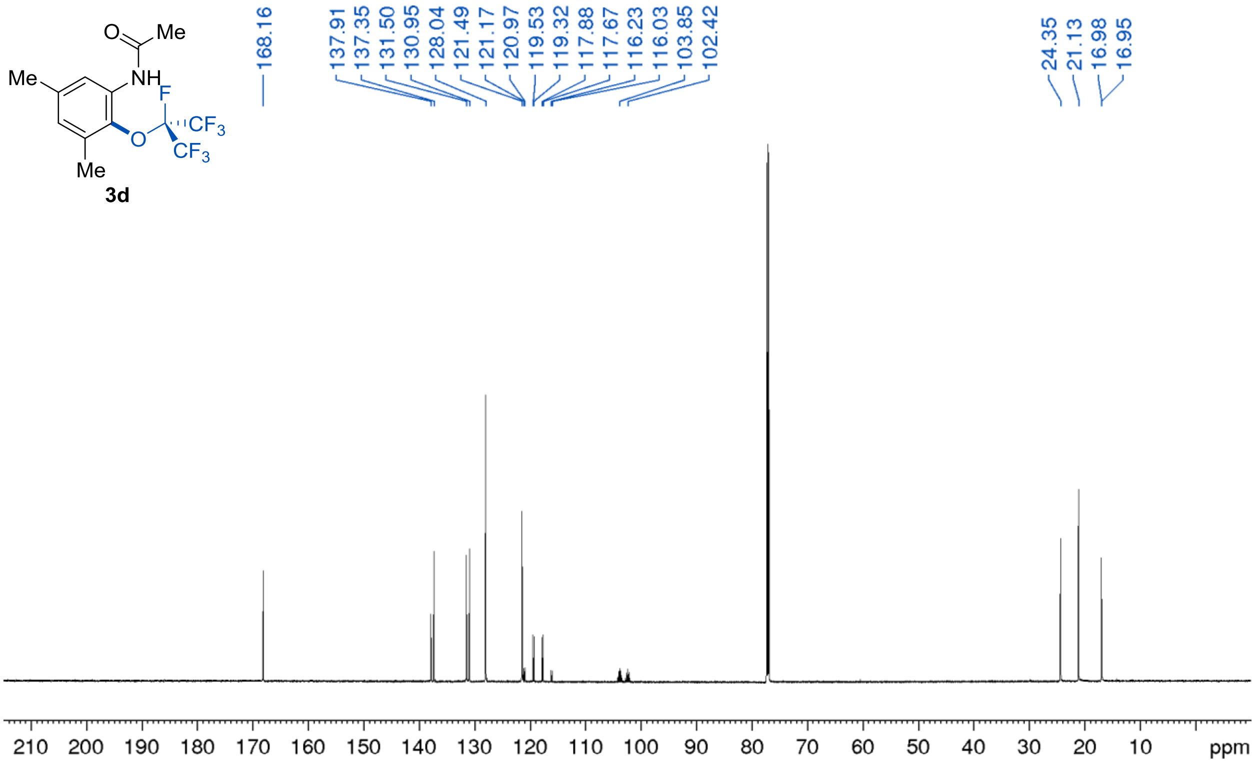

^{19}F NMR (376 MHz, $CDCl_3$, 25 ºC) of (3d)

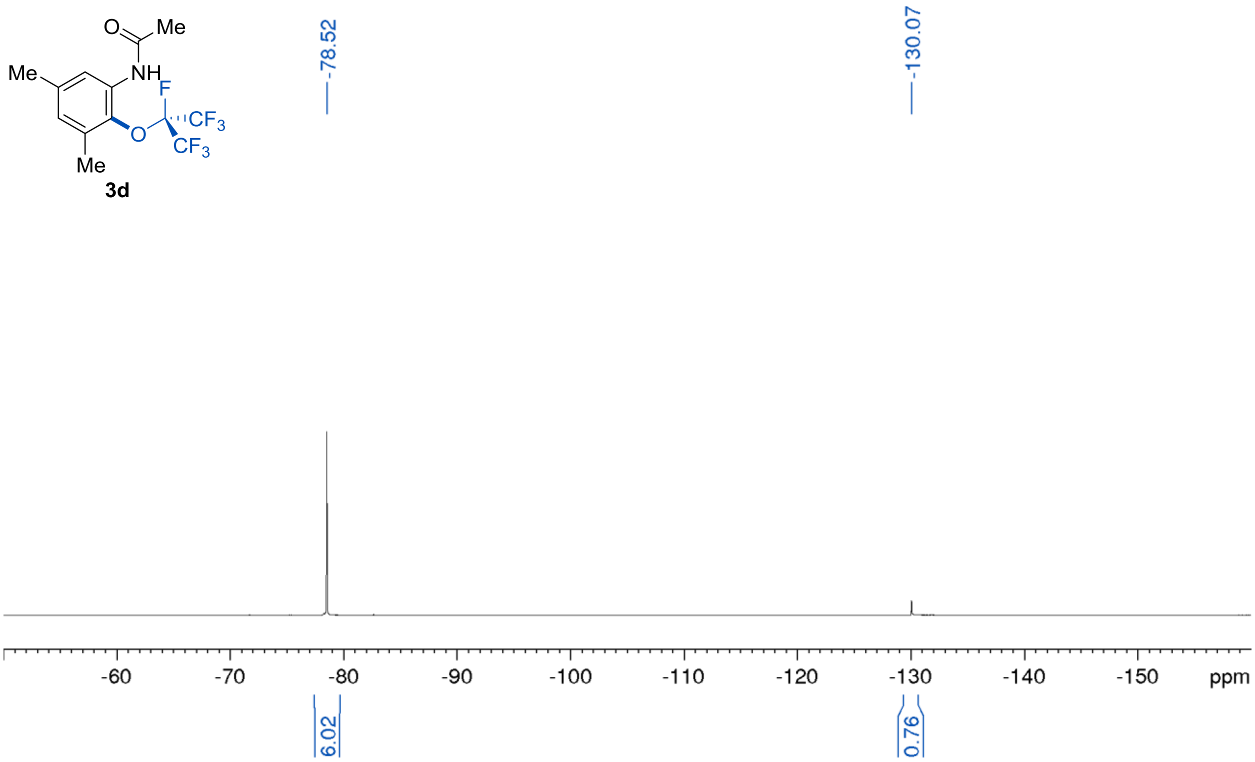

^{1}H NMR (700 MHz, $CDCl_3$, 25 ºC) of (3e)

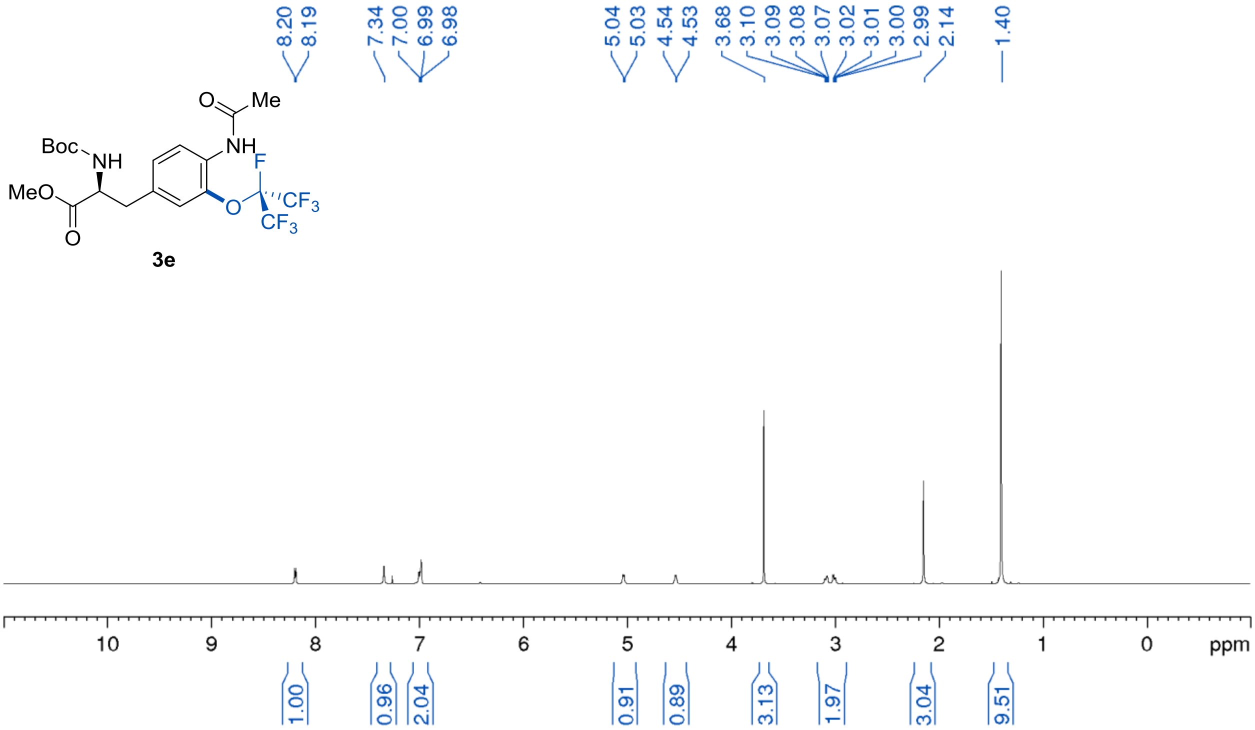

^{13}C NMR (175 MHz, $CDCl_3$, 25 ºC) of (3e)

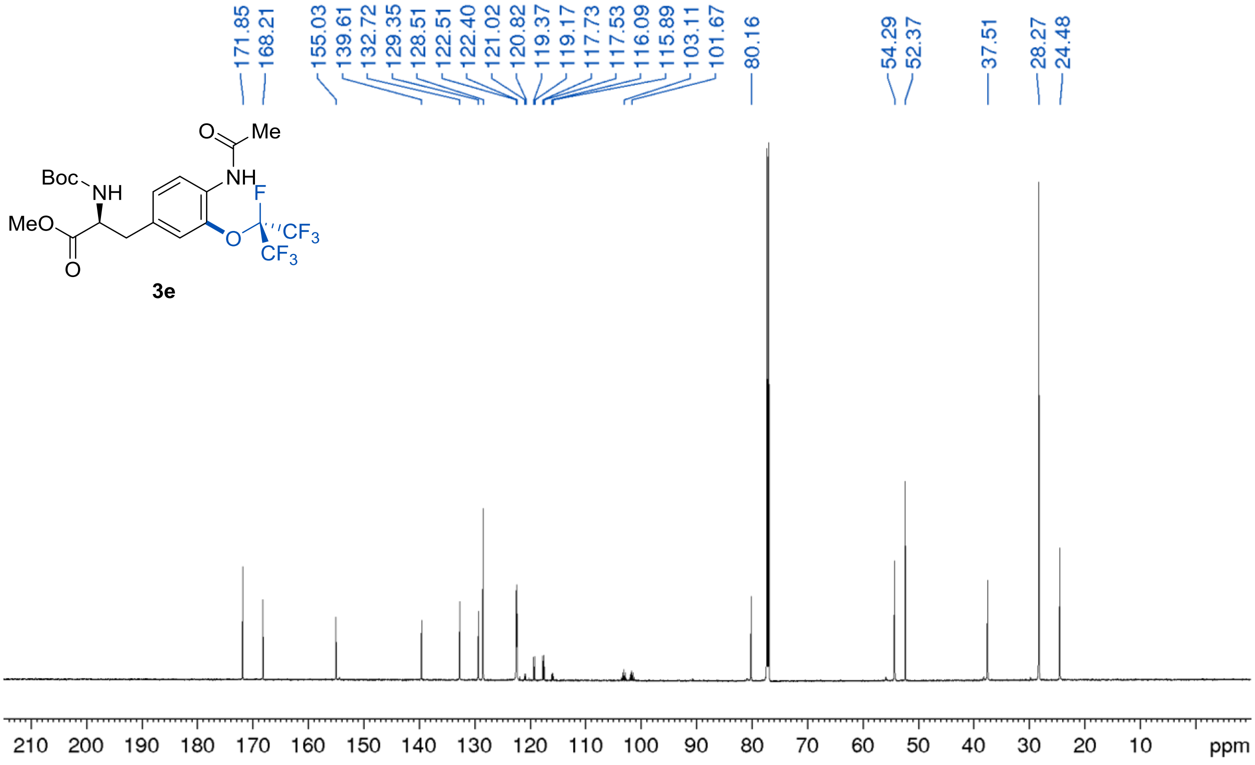

^{19}F NMR (376 MHz, $CDCl_3$, 25 ºC) of (3e)

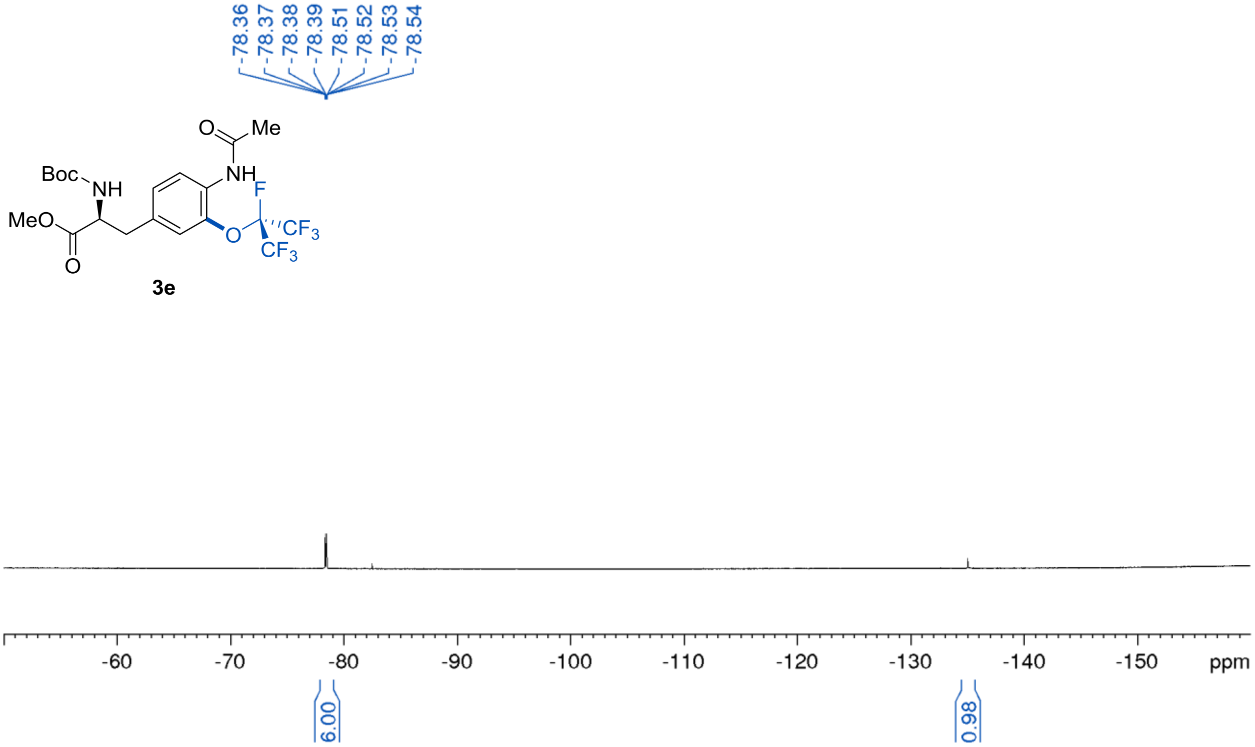

^{1}H NMR (700 MHz, $CDCl_3$, 25 ºC) of (3f)

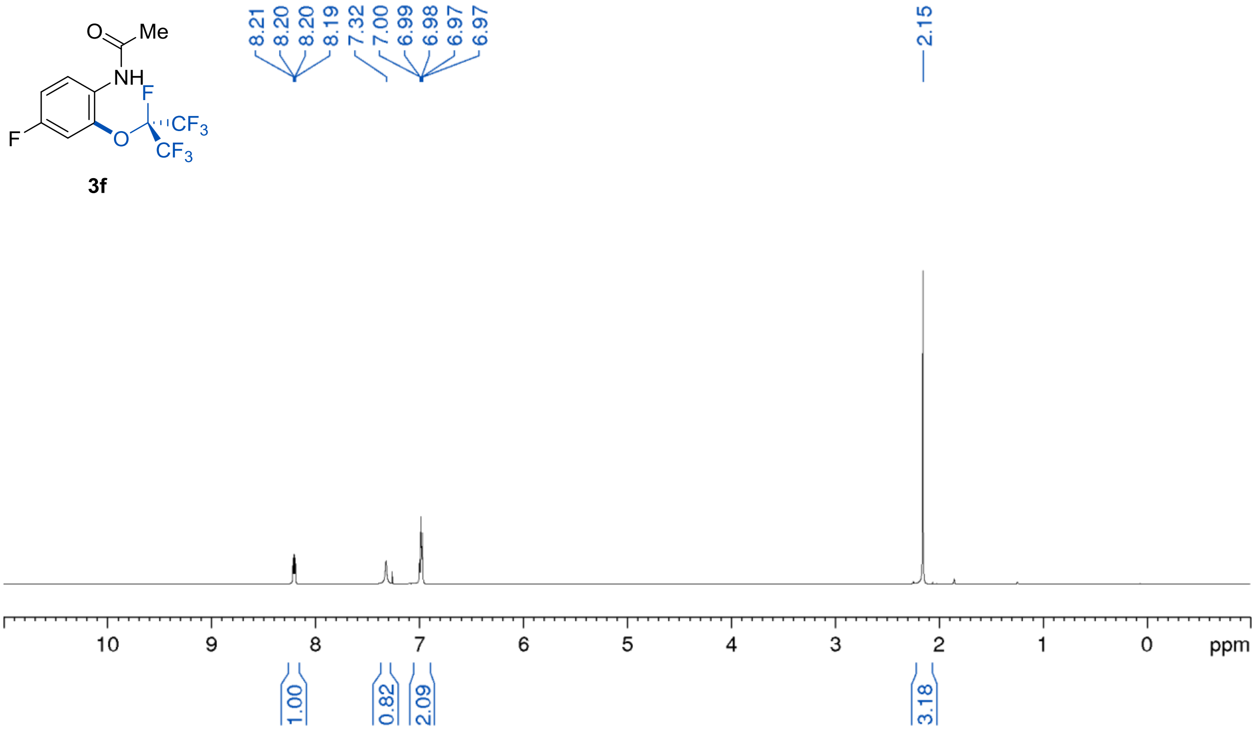

^{13}C NMR (175 MHz, $CDCl_3$, 25 ºC) of (3f)

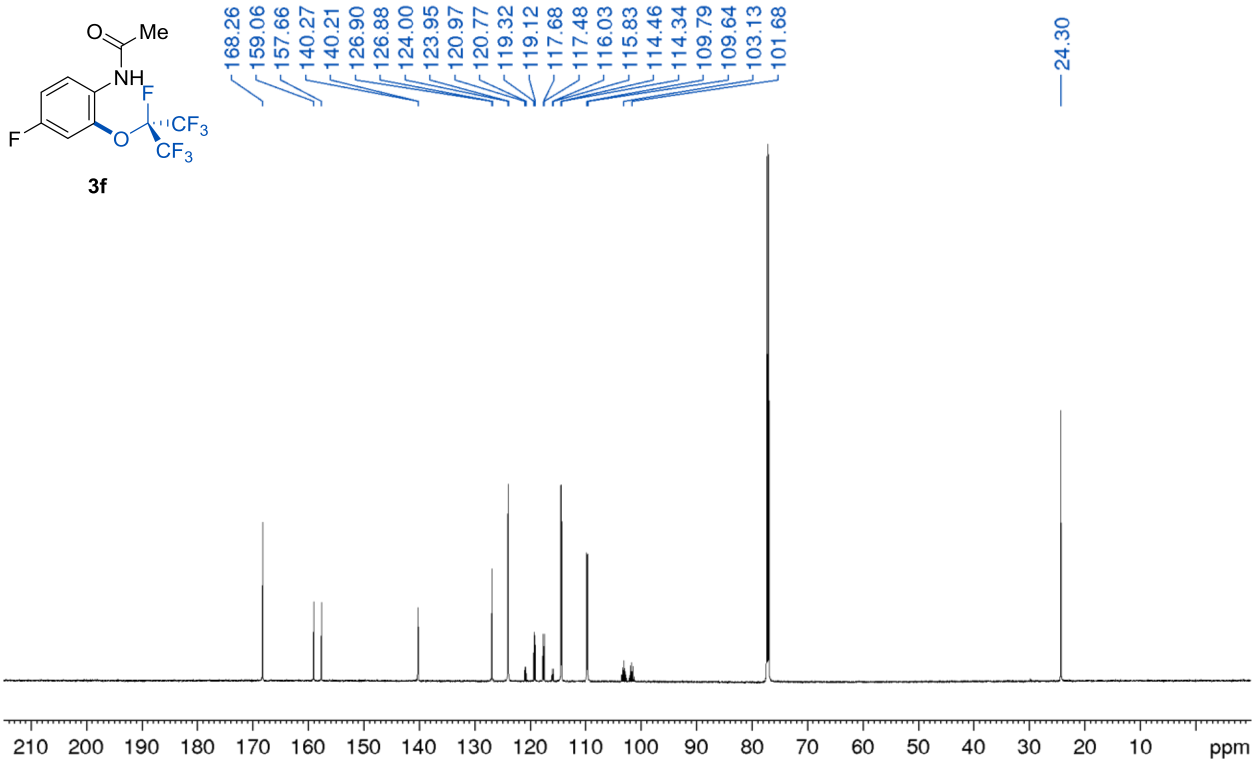

^{19}F NMR (376 MHz, $CDCl_3$, 25 ºC) of (3f)

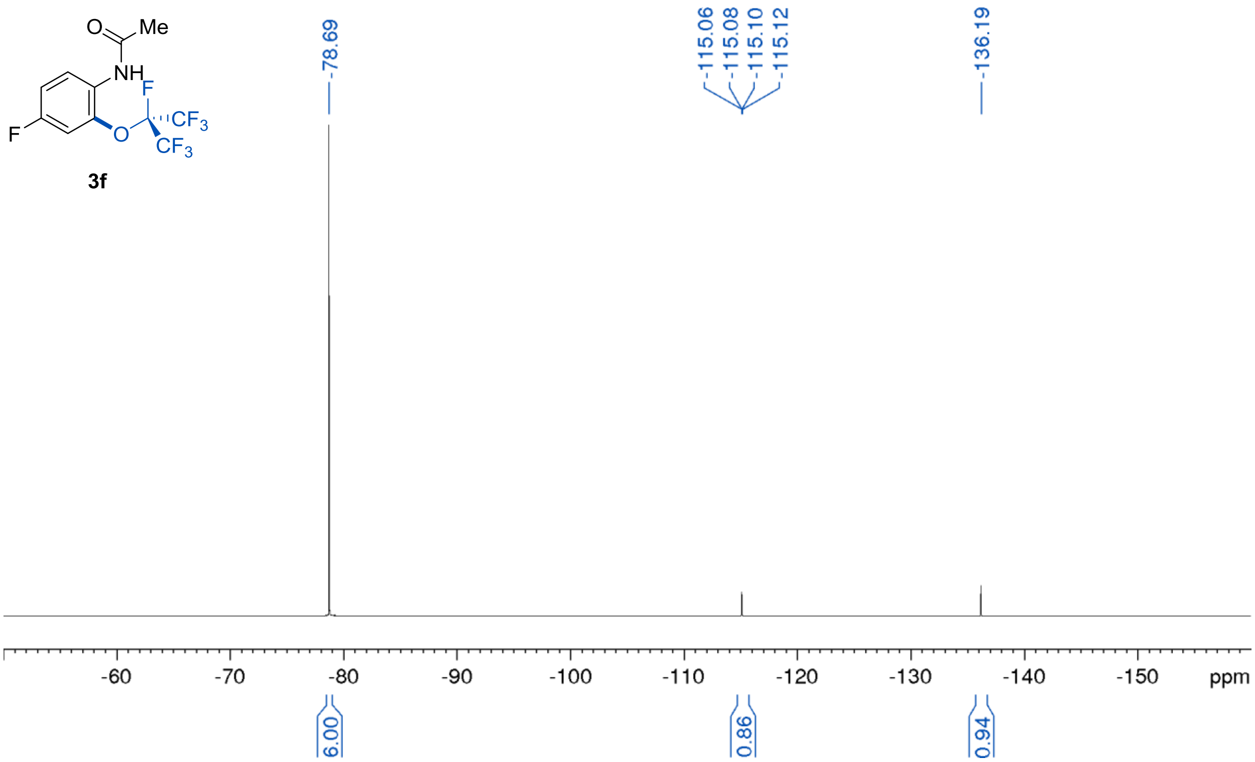

^{1}H NMR (700 MHz, $CDCl_3$, 25 ºC) of (3g)

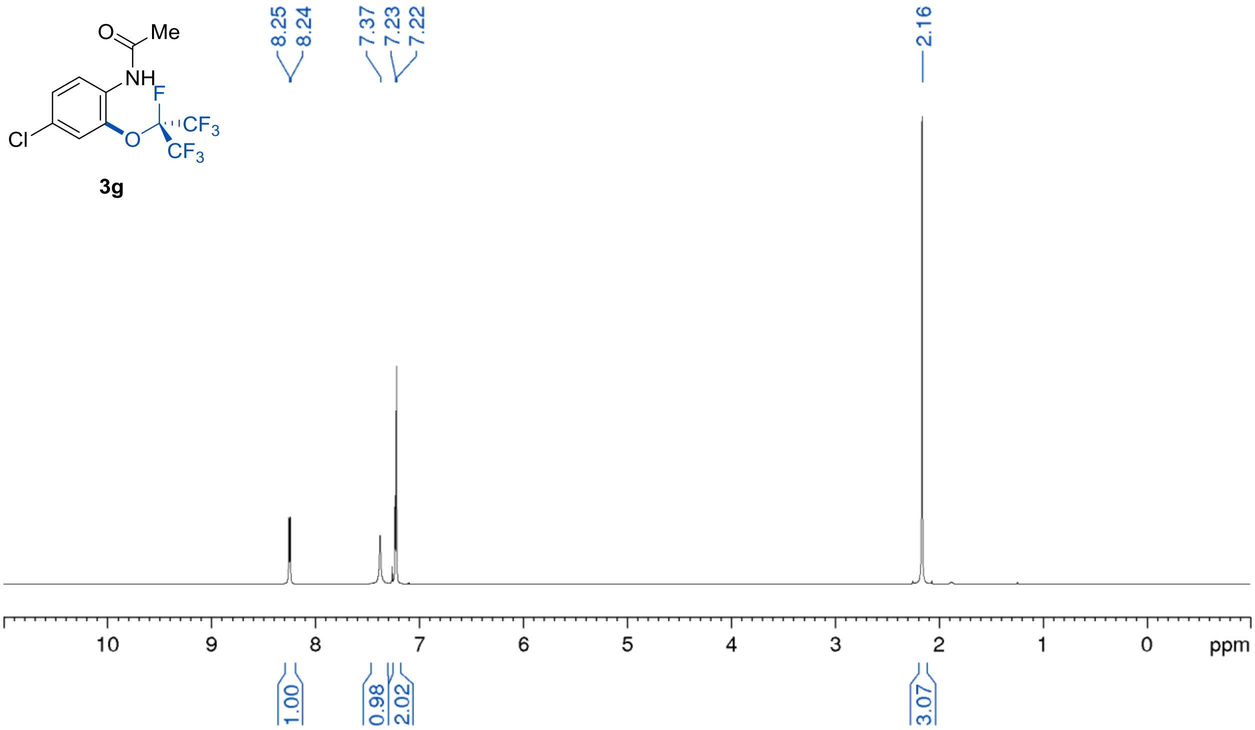

^{13}C NMR (175 MHz, $CDCl_3$, 25 ºC) of (3g)

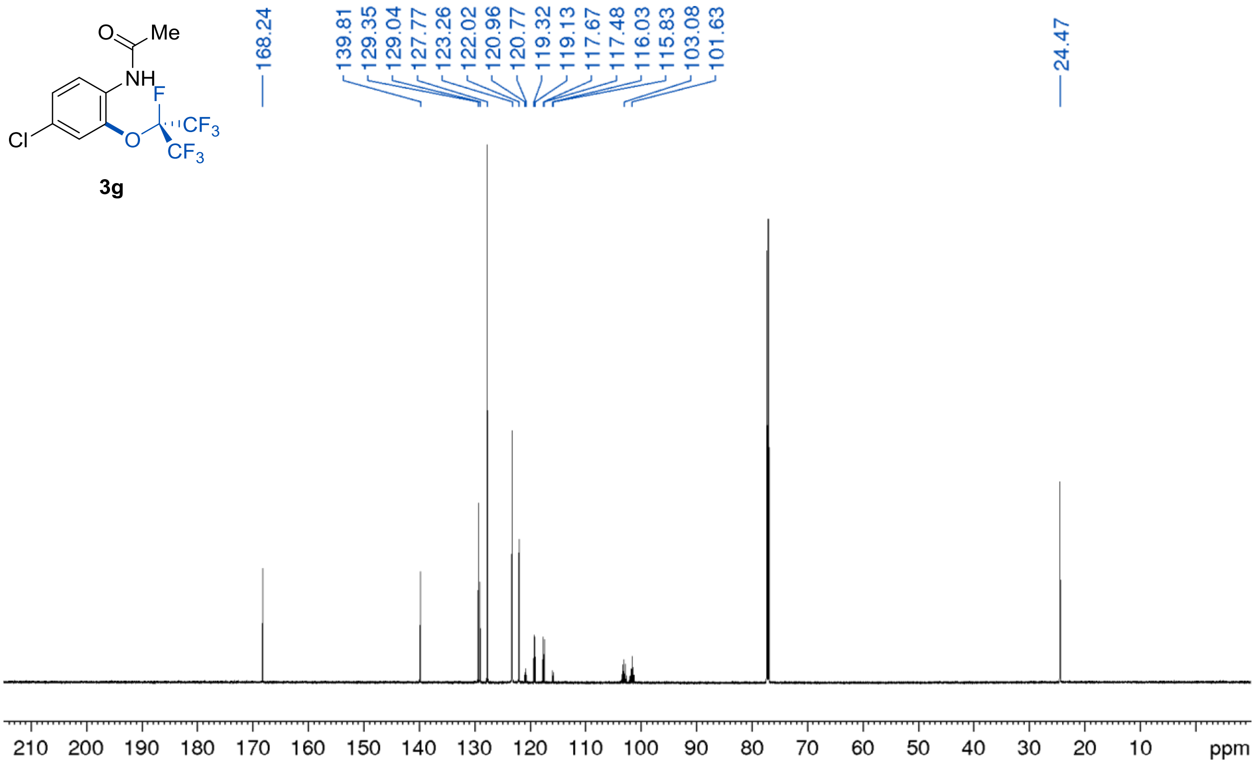

^{19}F NMR (376 MHz, $CDCl_3$, 25 ºC) of (3g)

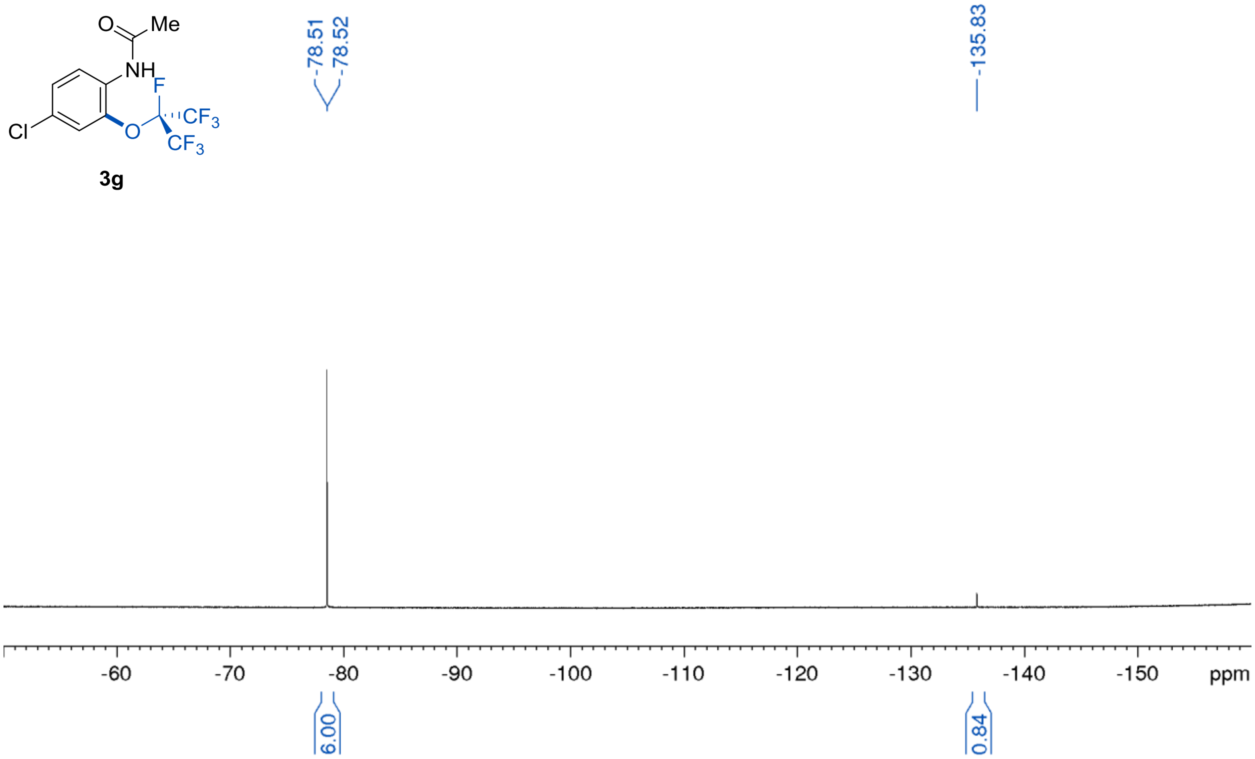

^{1}H NMR (700 MHz, $CDCl_3$, 25 ºC) of (3h)

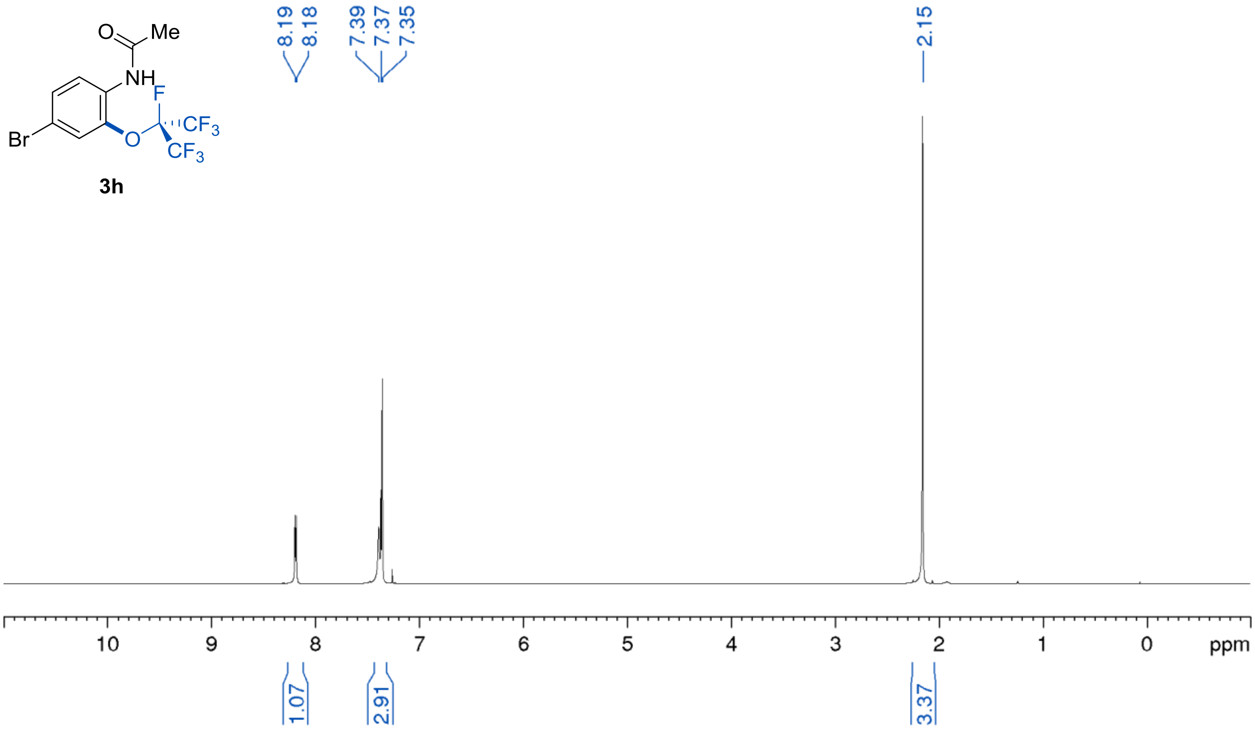

^{13}C NMR (175 MHz, $CDCl_3$, 25 ºC) of (3h)

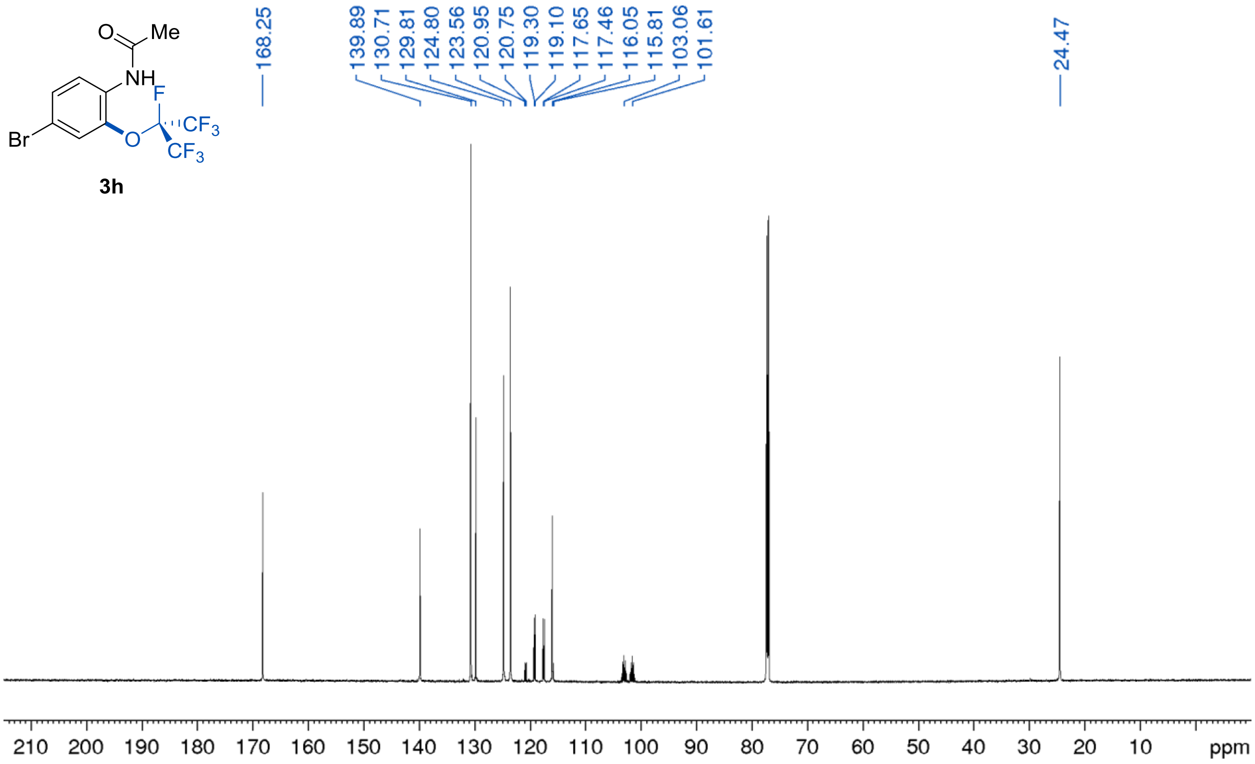

^{19}F NMR (376 MHz, $CDCl_3$, 25 ºC) of (3h)

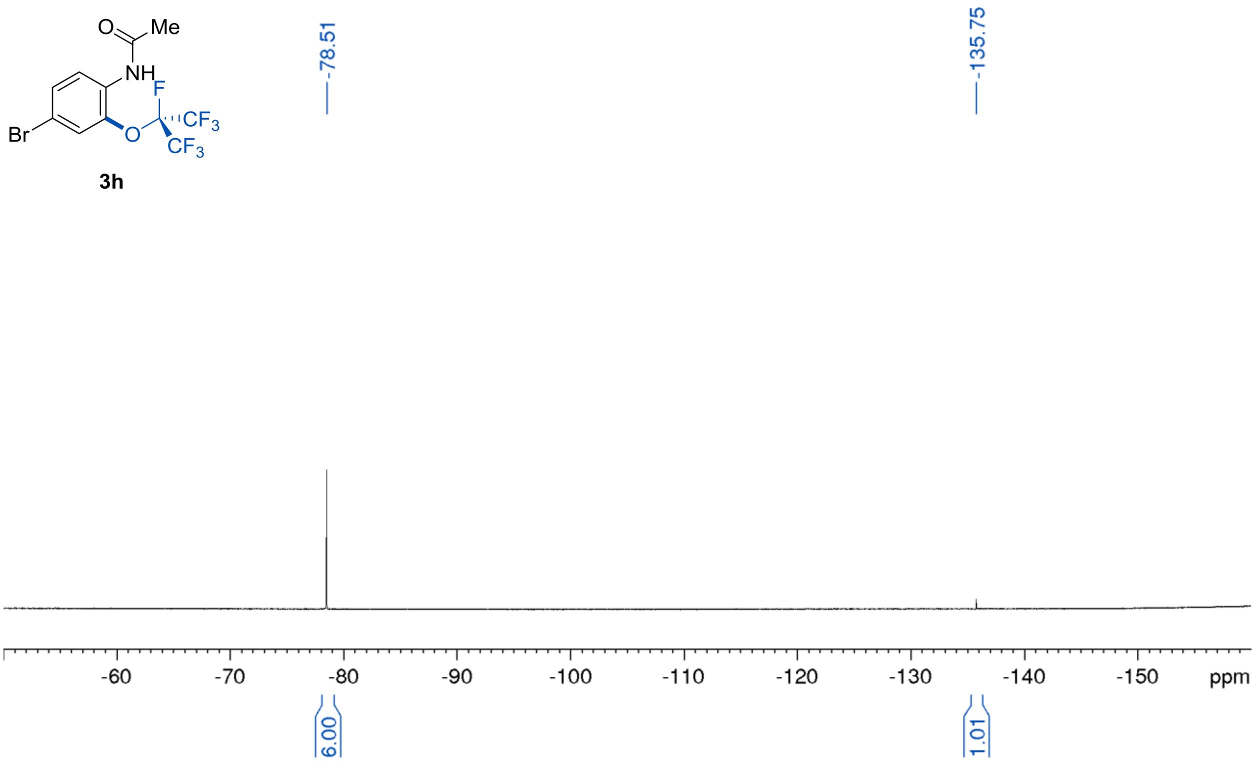

^{1}H NMR (700 MHz, $CDCl_3$, 25 ºC) of (3i)

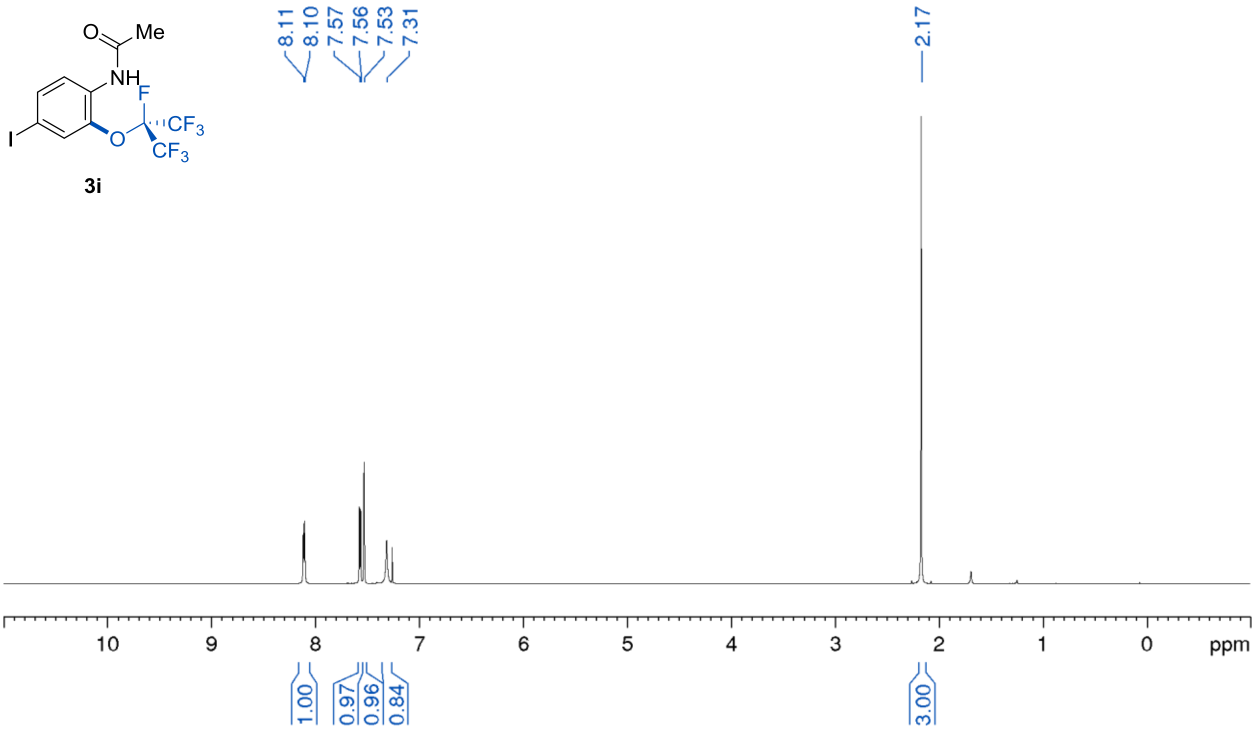

^{13}C NMR (175 MHz, $CDCl_3$, 25 ºC) of (3i)

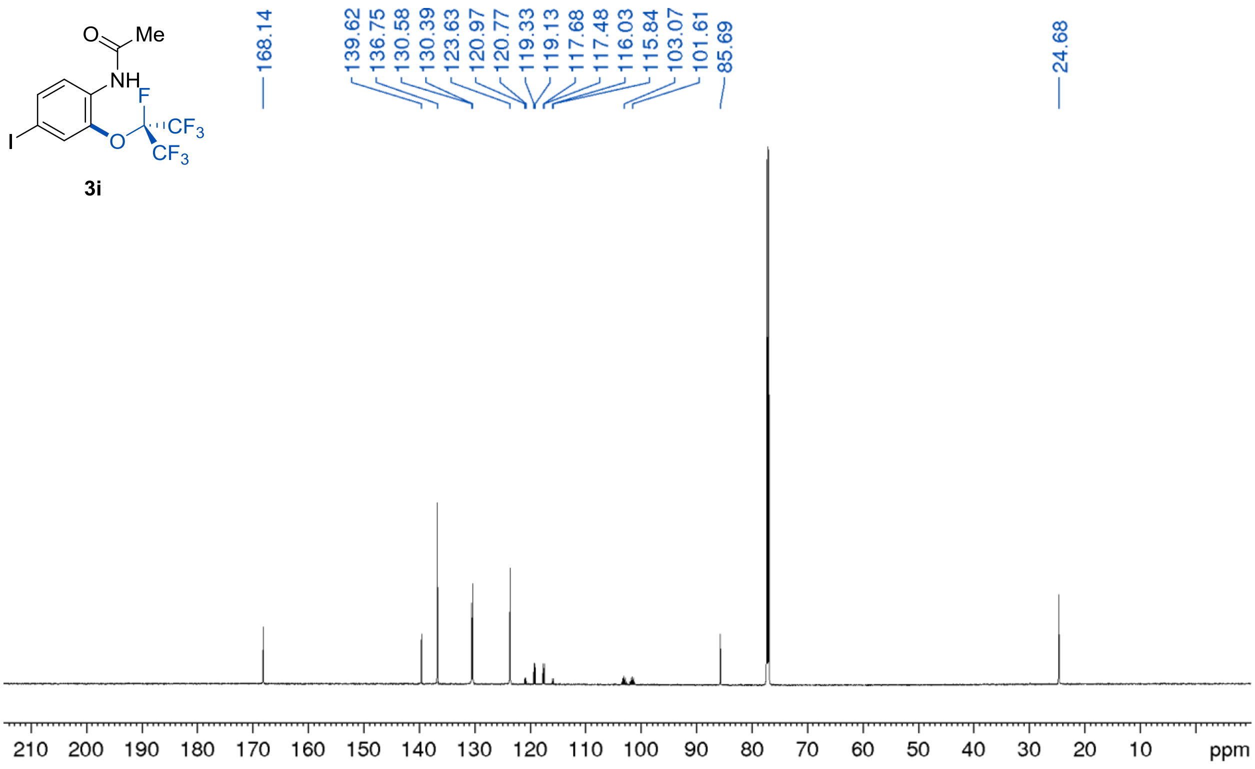

^{19}F NMR (376 MHz, $CDCl_3$, 25 ºC) of (3i)

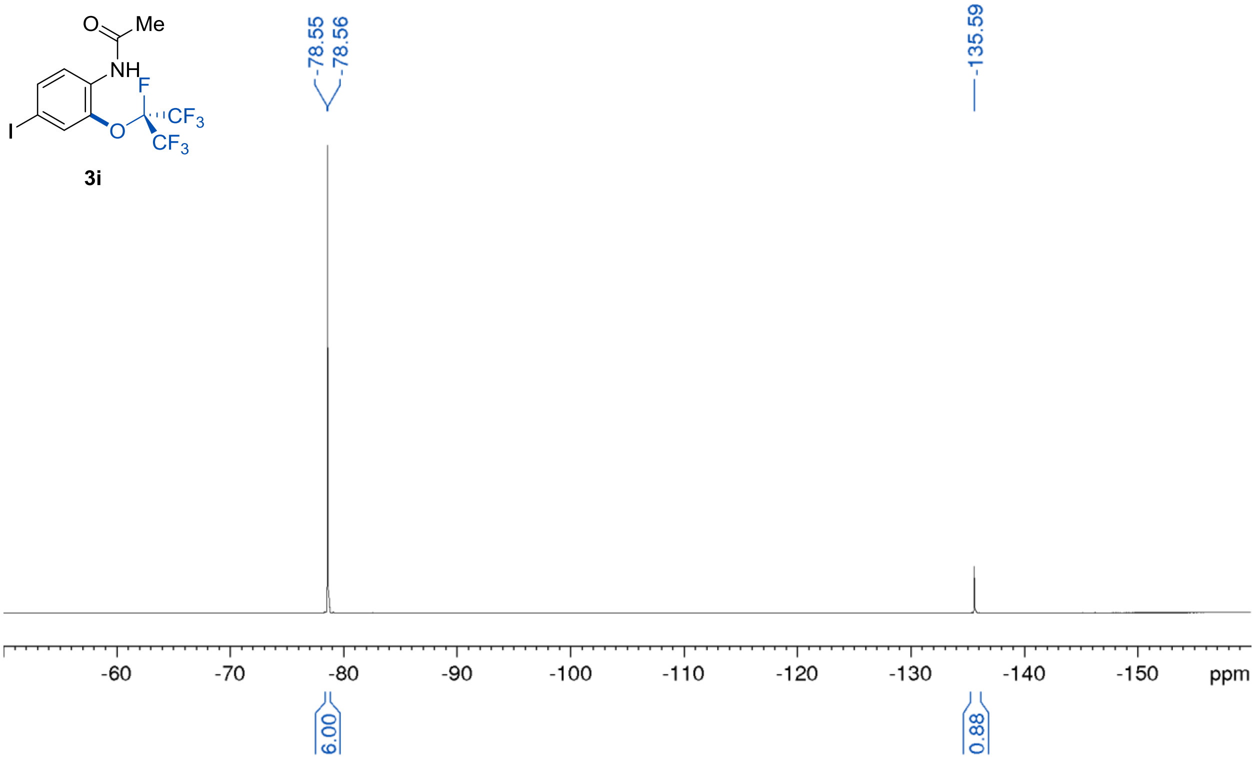

^{1}H NMR (700 MHz, $CDCl_3$, 25 ºC) of (3j)

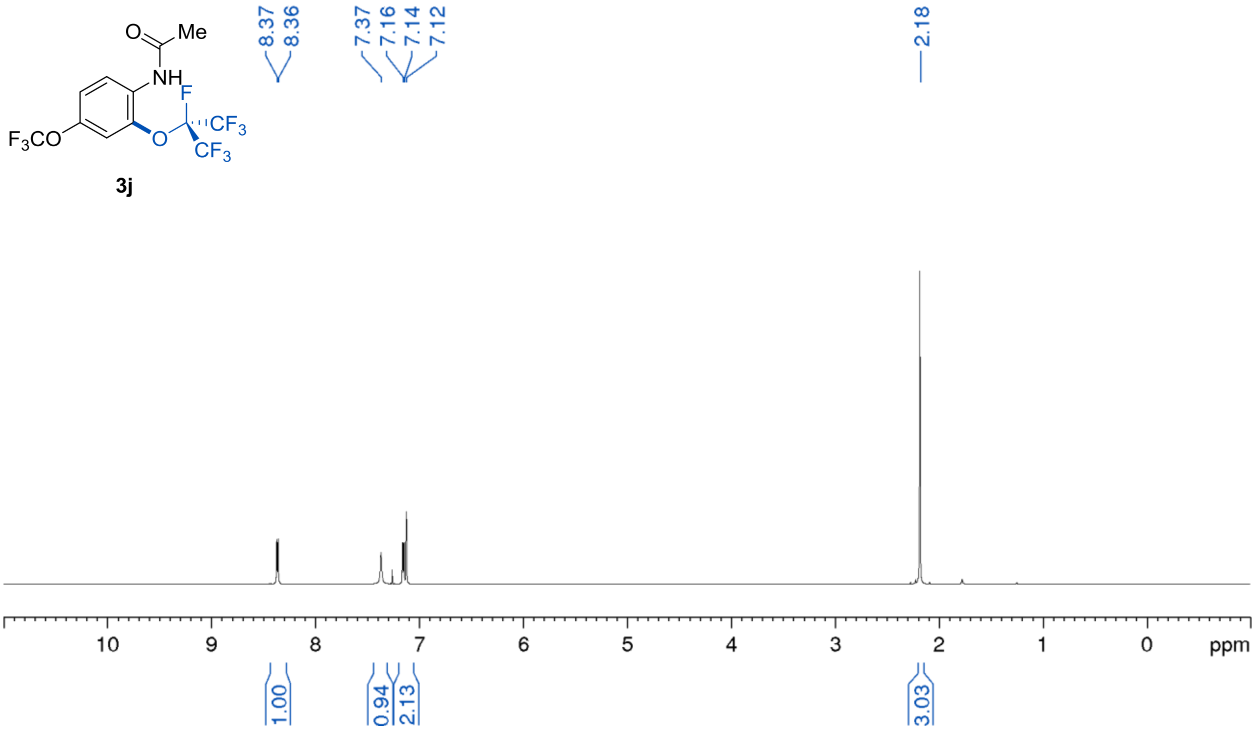

^{13}C NMR (175 MHz, $CDCl_3$, 25 ºC) of (3j)

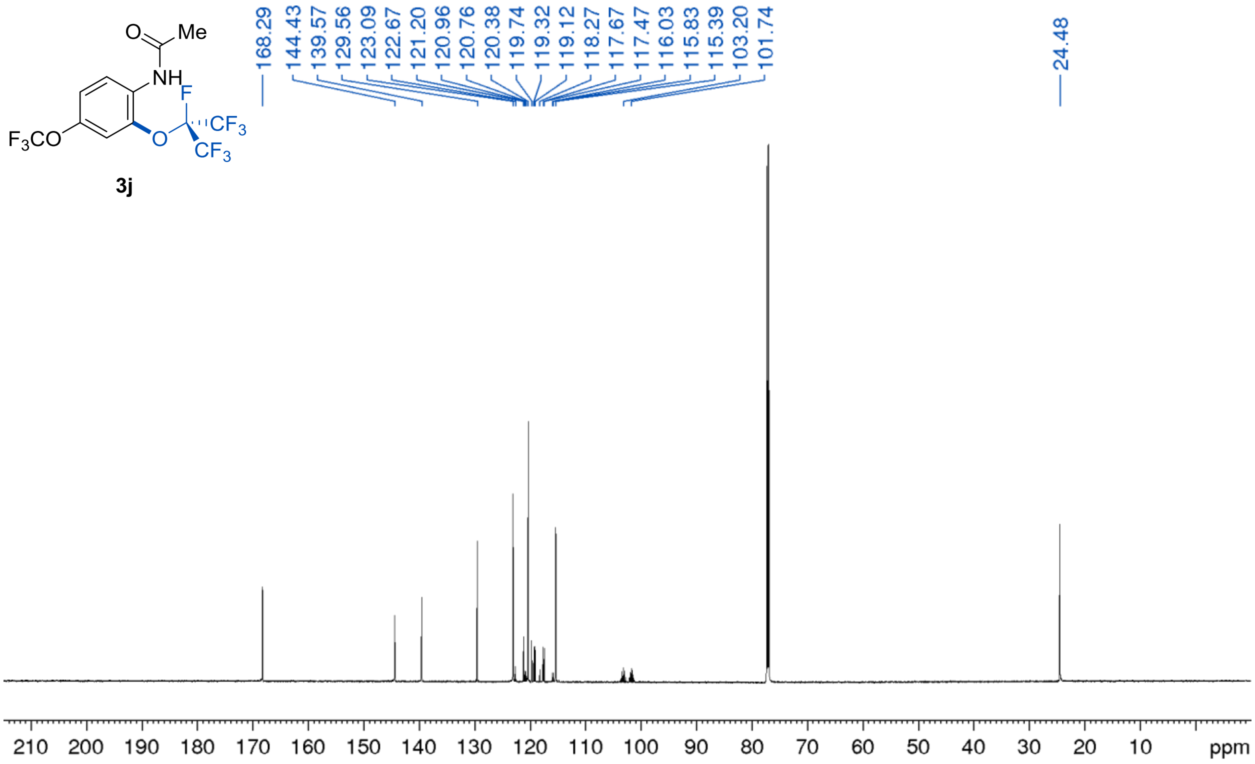

^{19}F NMR (376 MHz, $CDCl_3$, 25 ºC) of (3j)

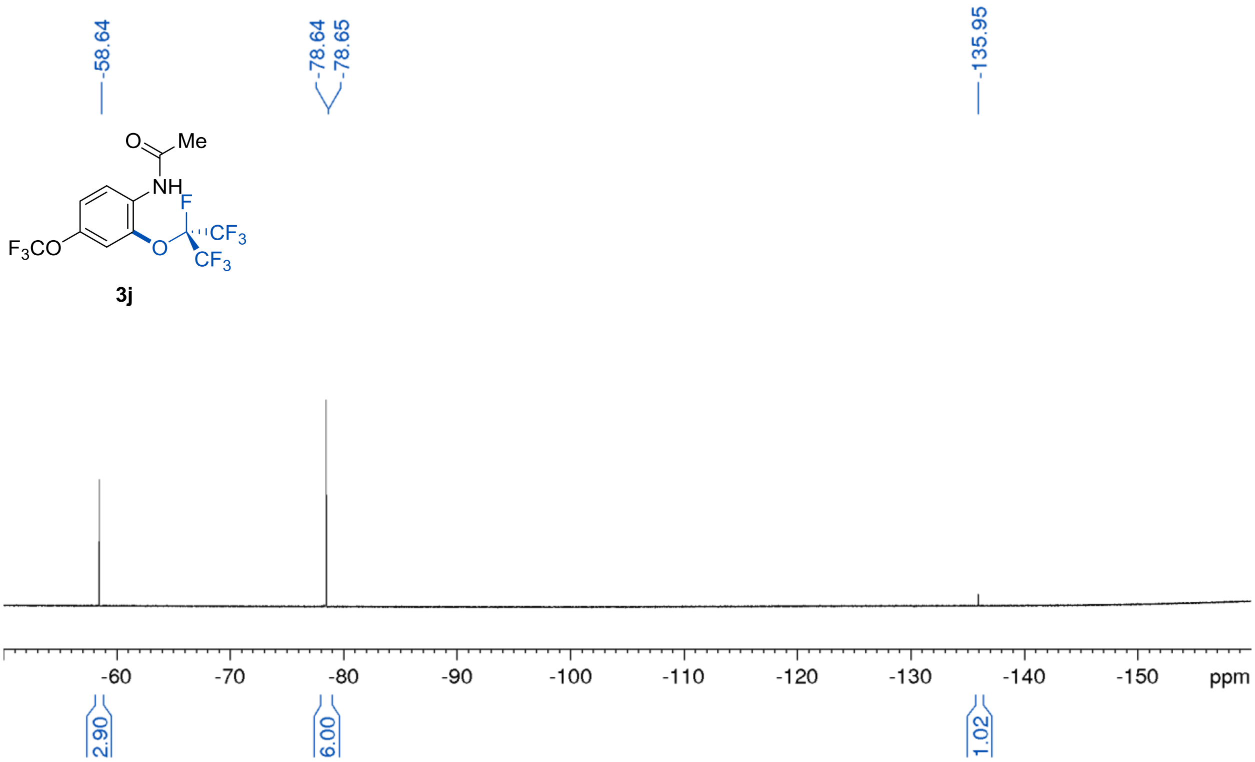

¹H NMR (700 MHz, $CDCl_3$, 25 °C) of (3k)

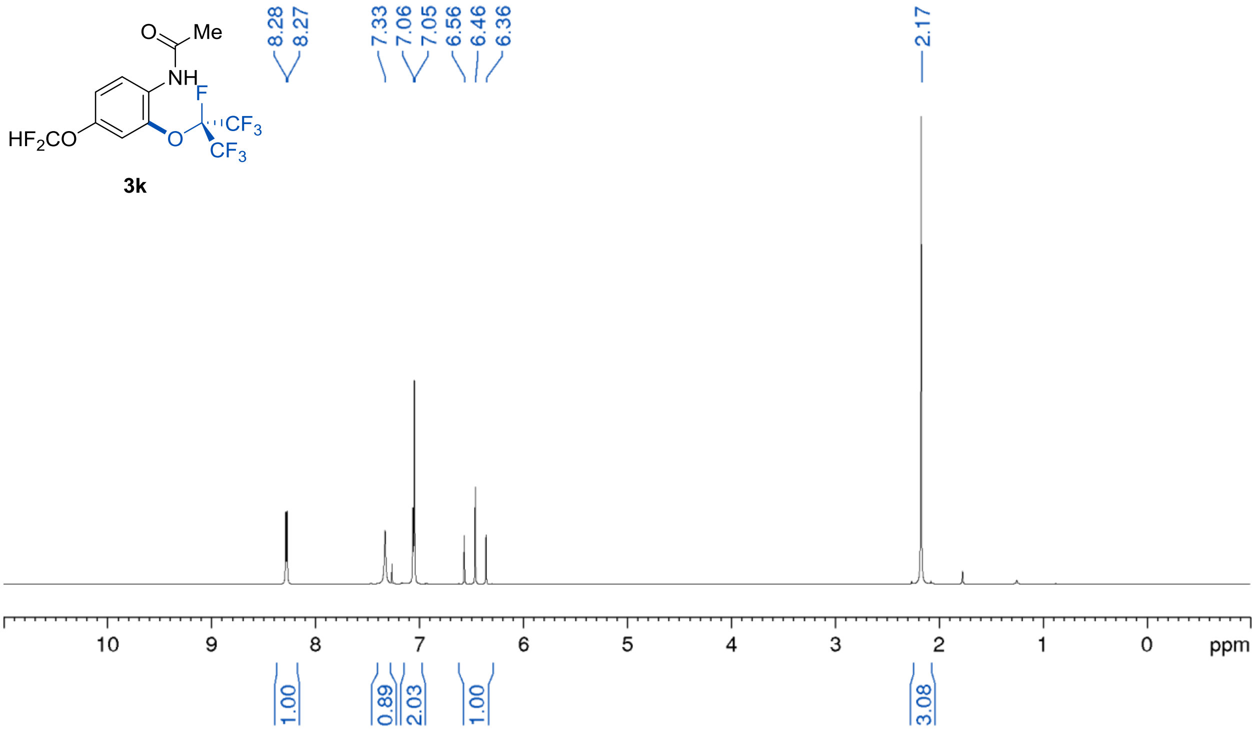

¹³C NMR (175 MHz, $CDCl_3$, 25 °C) of (3k)

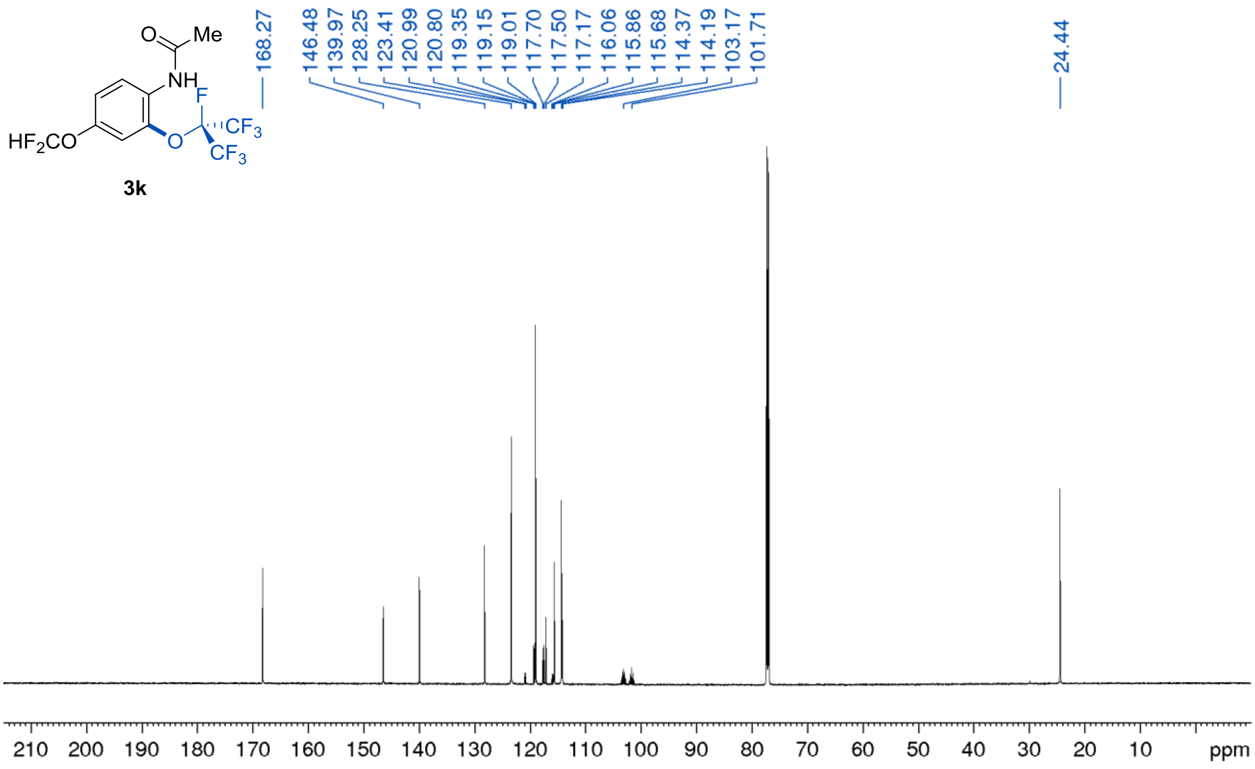

^{19}F NMR (376 MHz, $CDCl_3$, 25 ºC) of (3k)

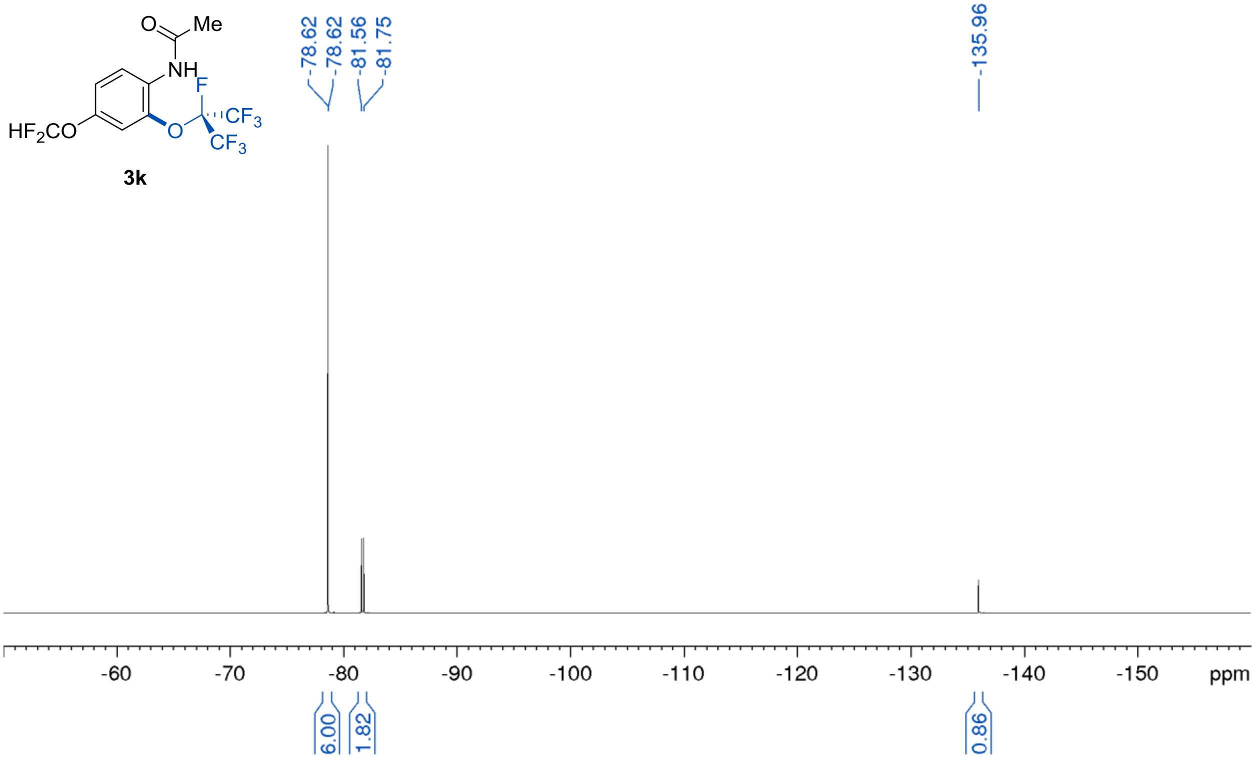

^{1}H NMR (700 MHz, $CDCl_3$, 25 ºC) of (3l)

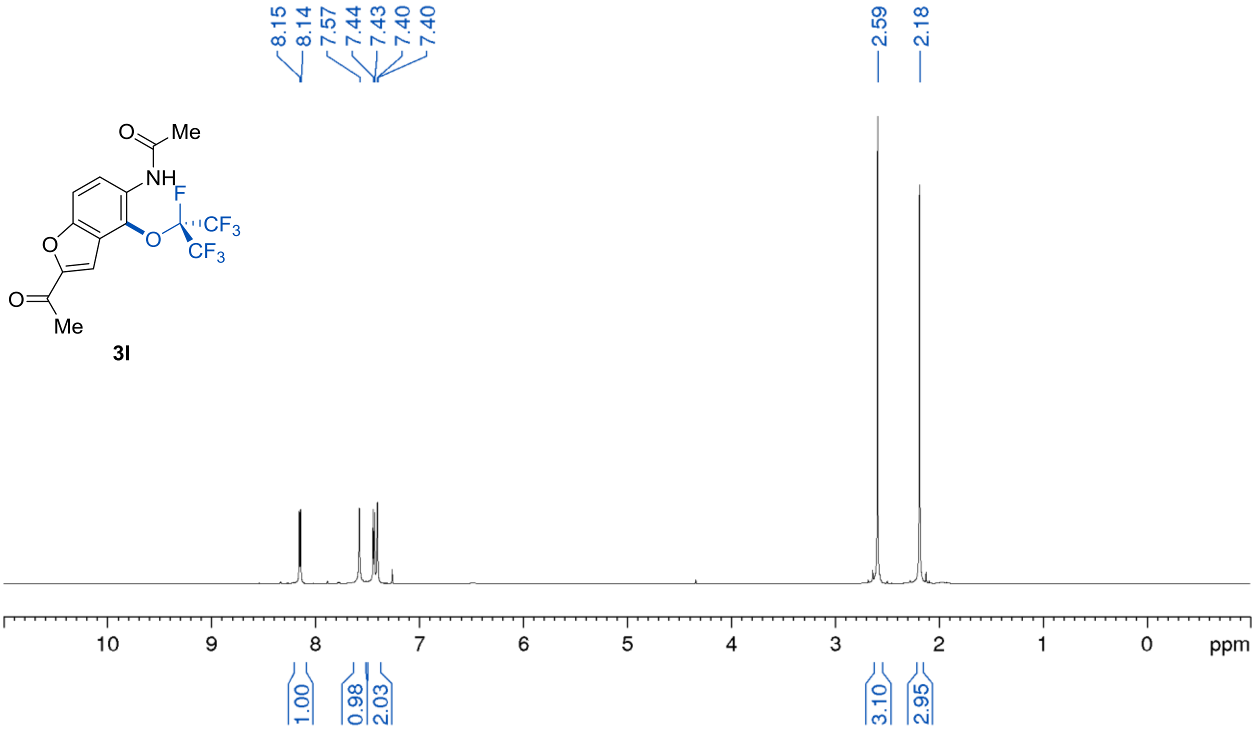

^{13}C NMR (175 MHz, $CDCl_3$, 25 ºC) of (3l)

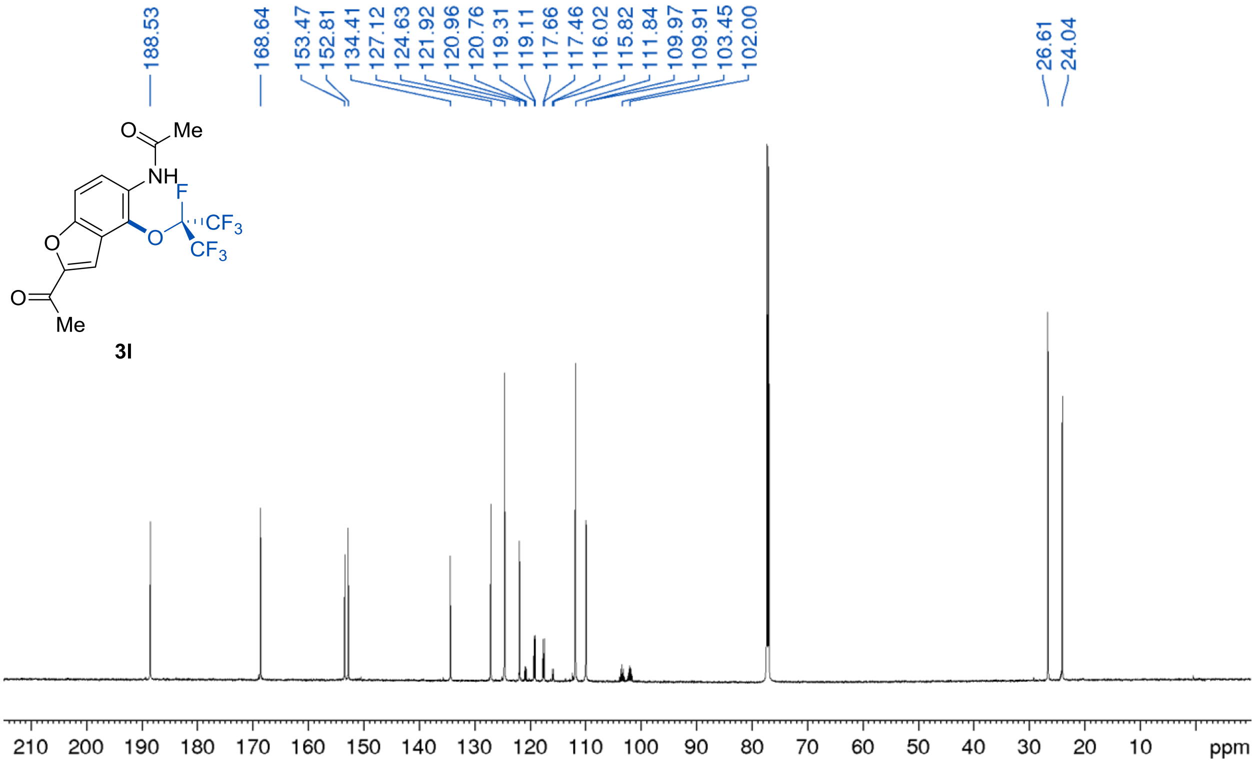

^{19}F NMR (376 MHz, $CDCl_3$, 25 ºC) of (3l)

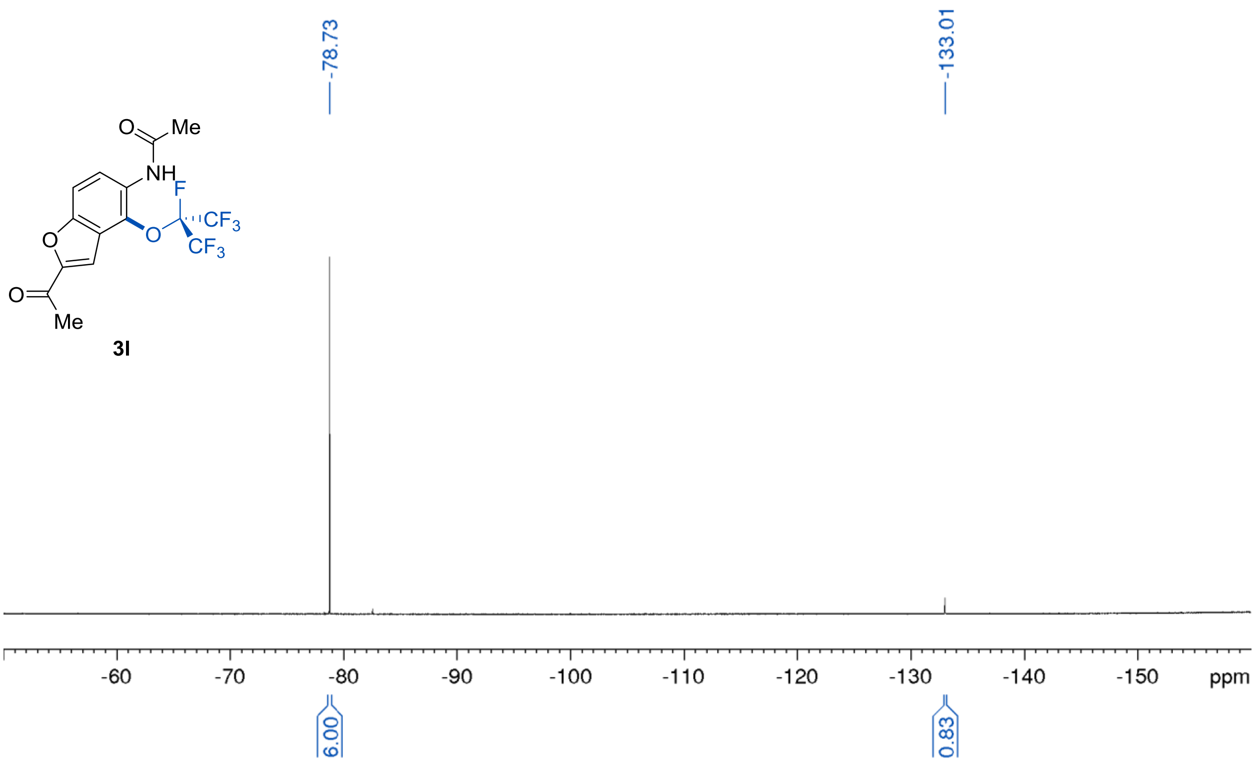

^{1}H NMR (700 MHz, $CDCl_3$, 25 ºC) of (3m)

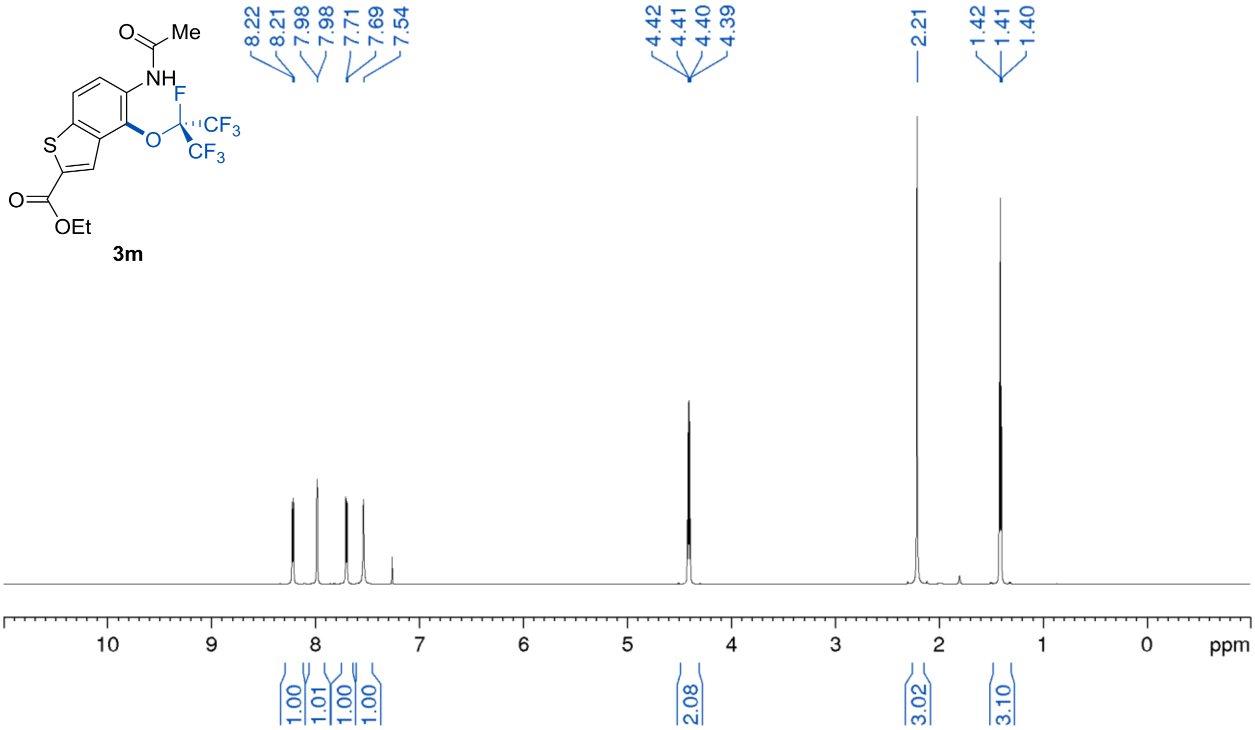

^{13}C NMR (175 MHz, $CDCl_3$, 25 ºC) of (3m)

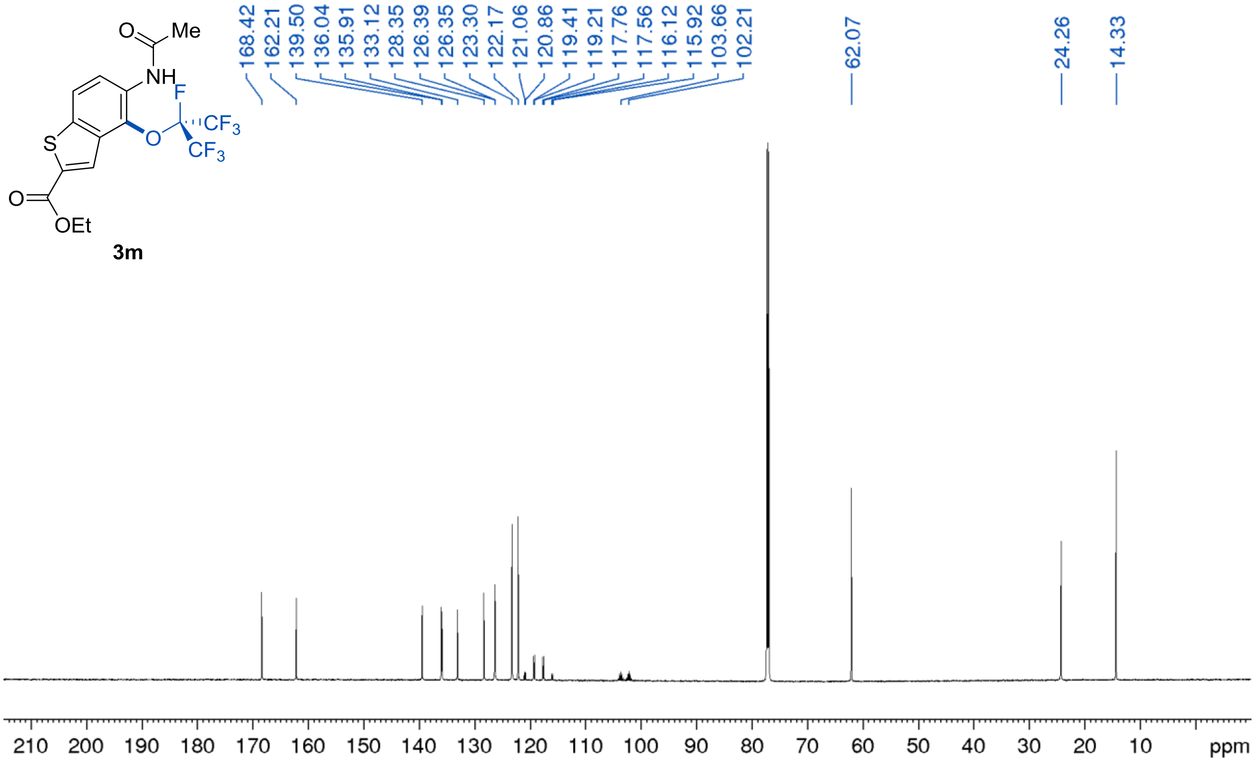

^{19}F NMR (376 MHz, $CDCl_3$, 25 ºC) of (3m)

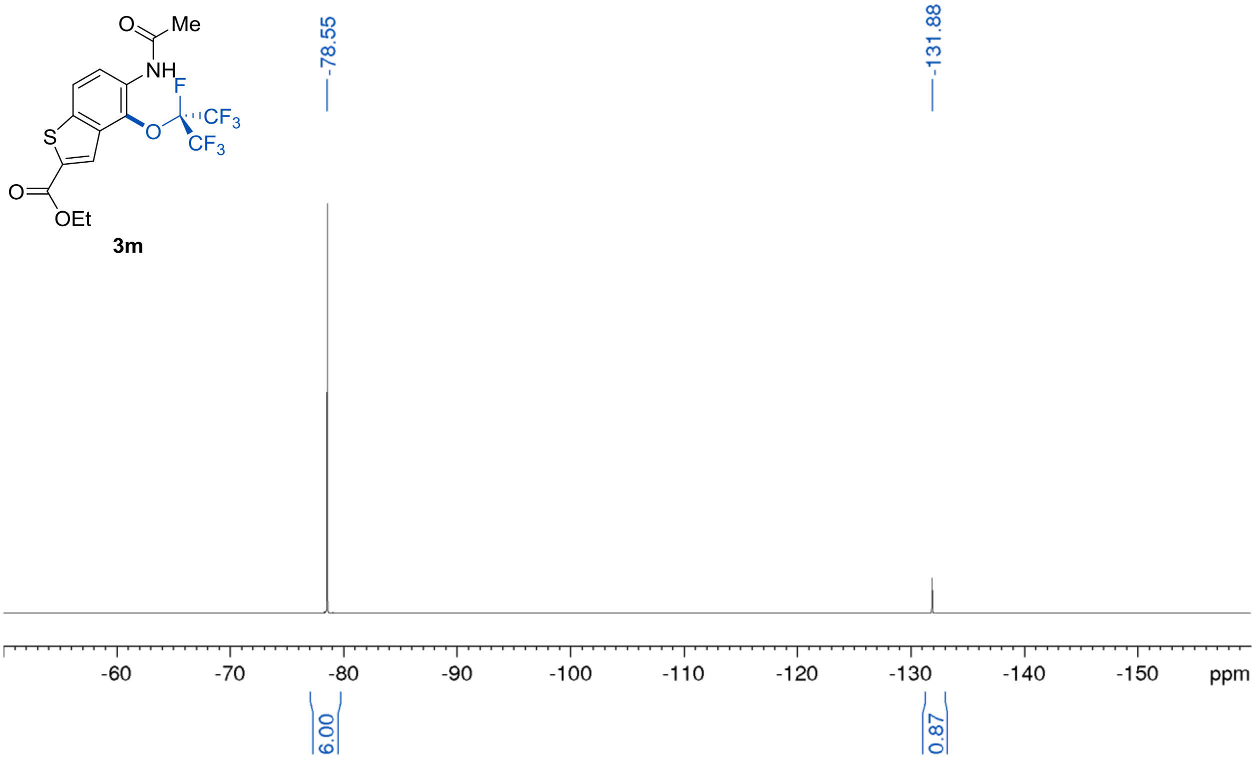

^{1}H NMR (700 MHz, $CDCl_3$, 25 ºC) of (3n)

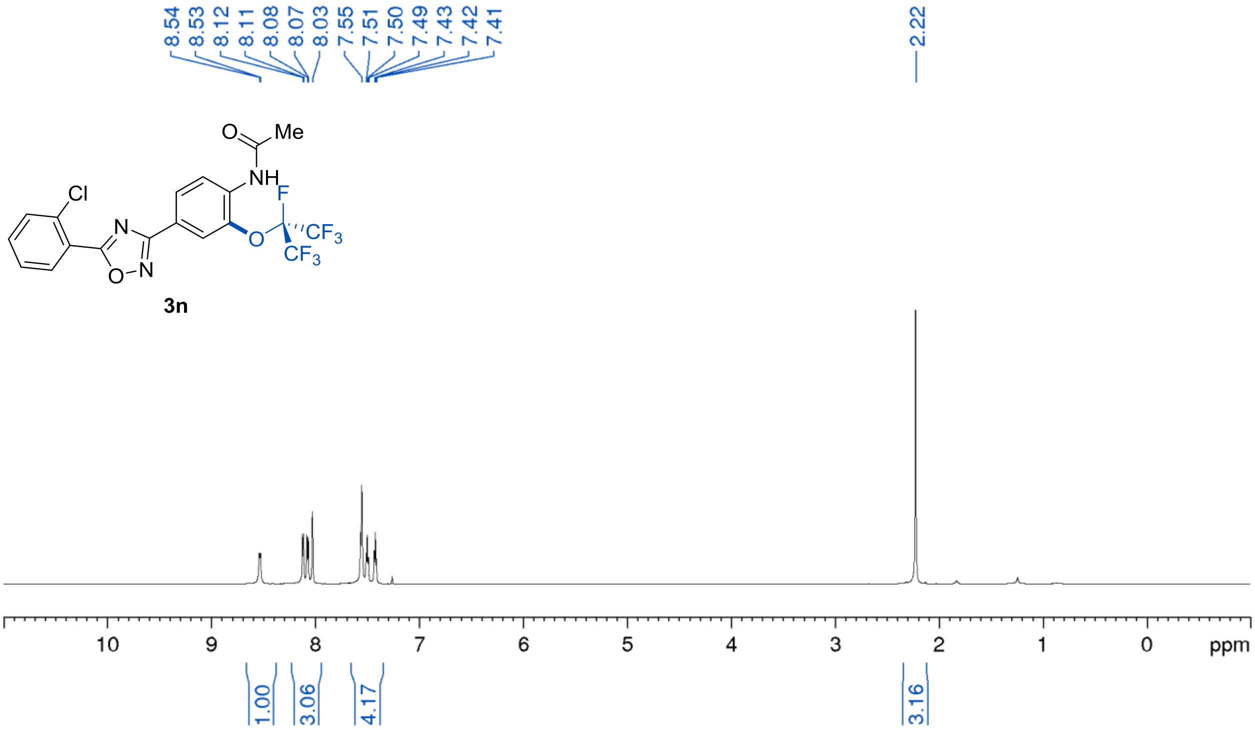

^{13}C NMR (175 MHz, $CDCl_3$, 25 °C) of (3n)

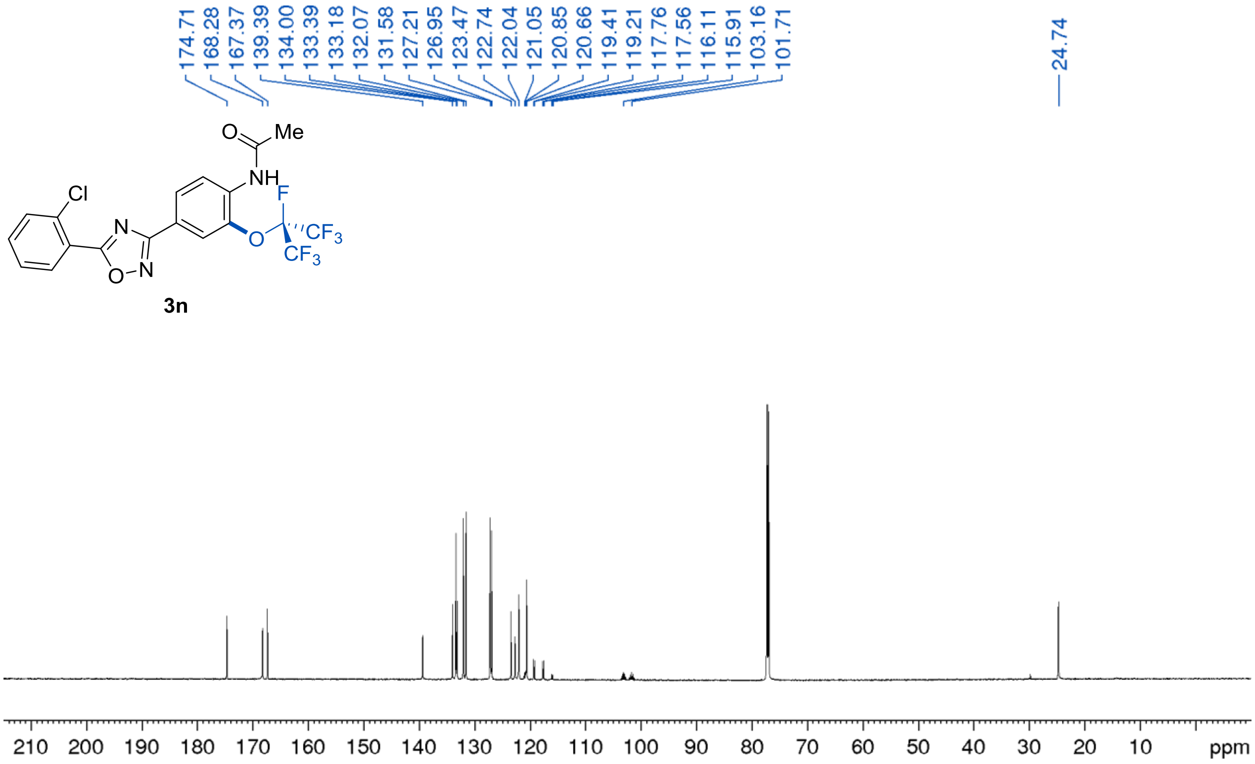

^{19}F NMR (376 MHz, $CDCl_3$, 25 °C) of (3n)

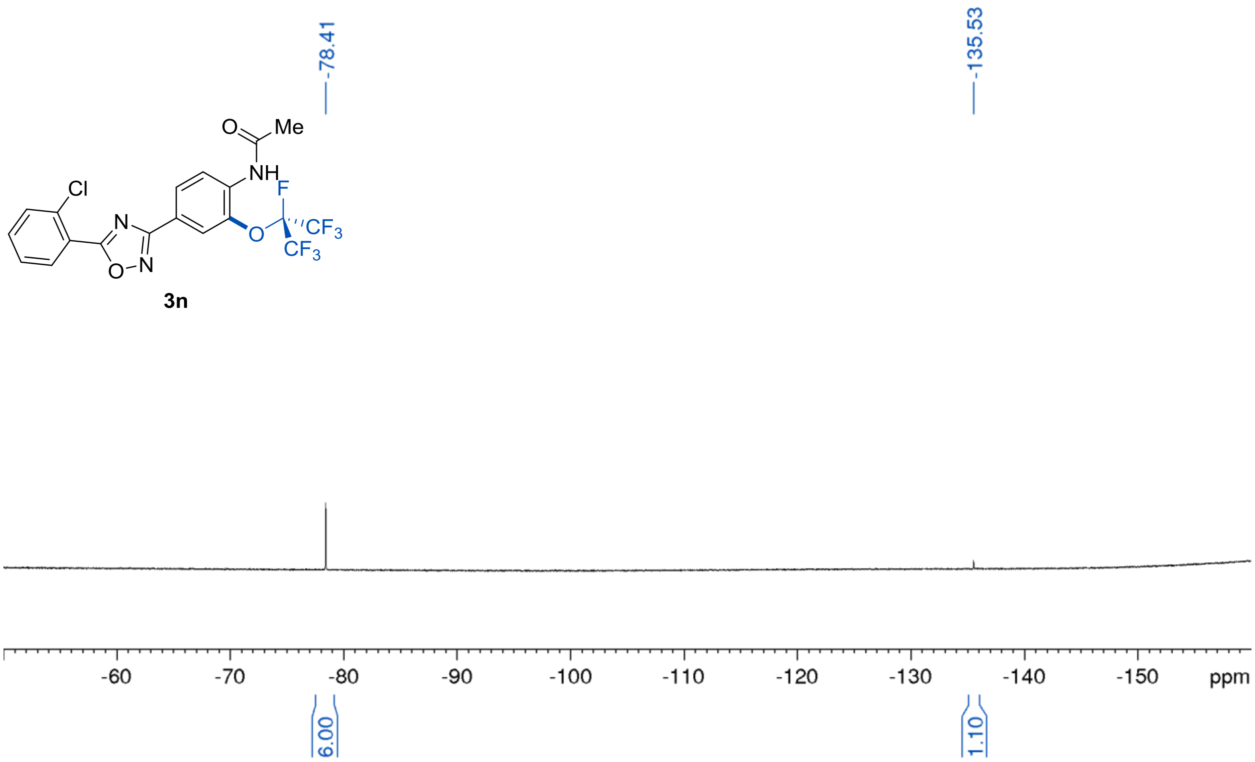

^{1}H NMR (700 MHz, $CDCl_3$, 25 °C) of (3o)

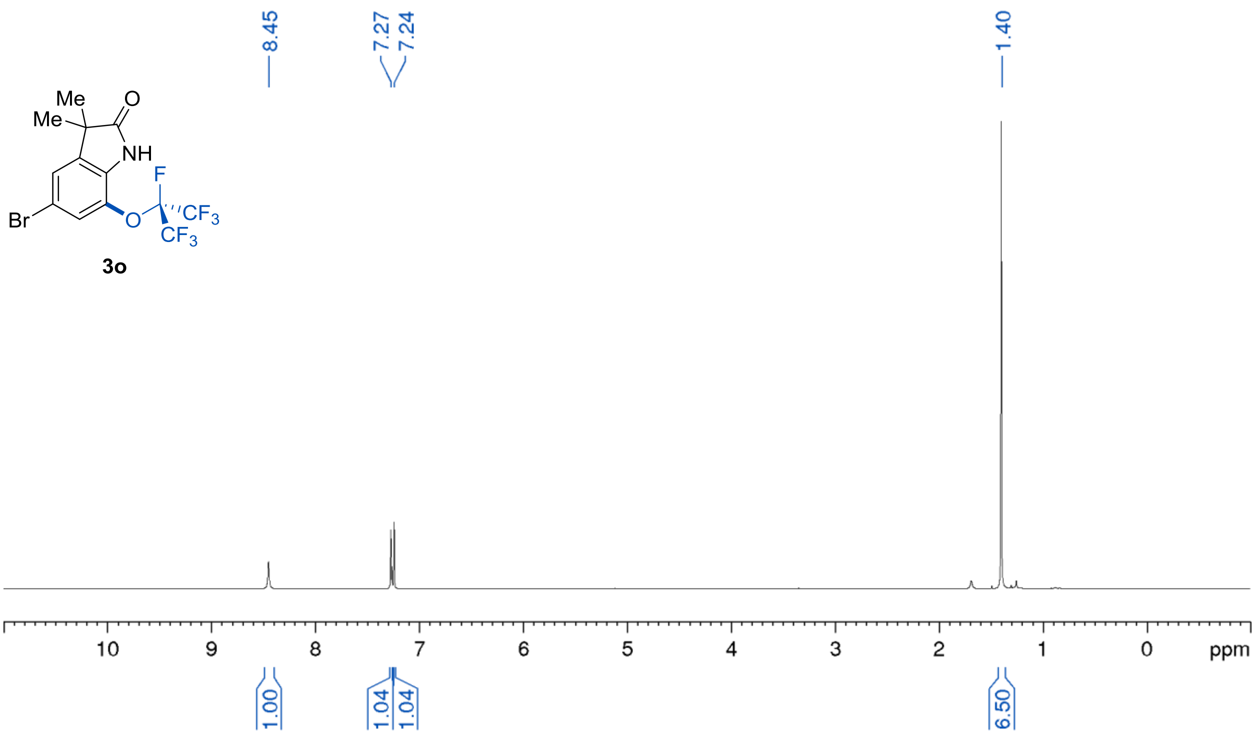

^{13}C NMR (175 MHz, $CDCl_3$, 25 °C) of (3o)

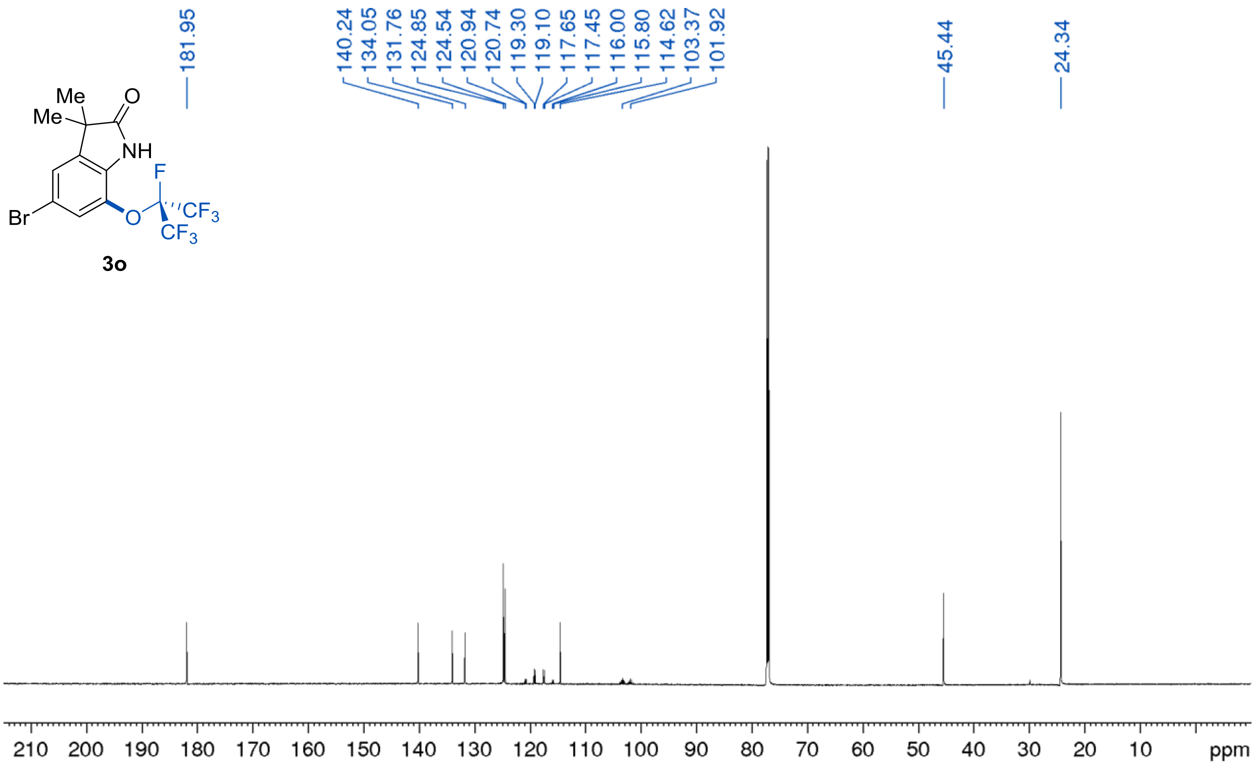

^{19}F NMR (376 MHz, $CDCl_3$, 25 ºC) of (3o)

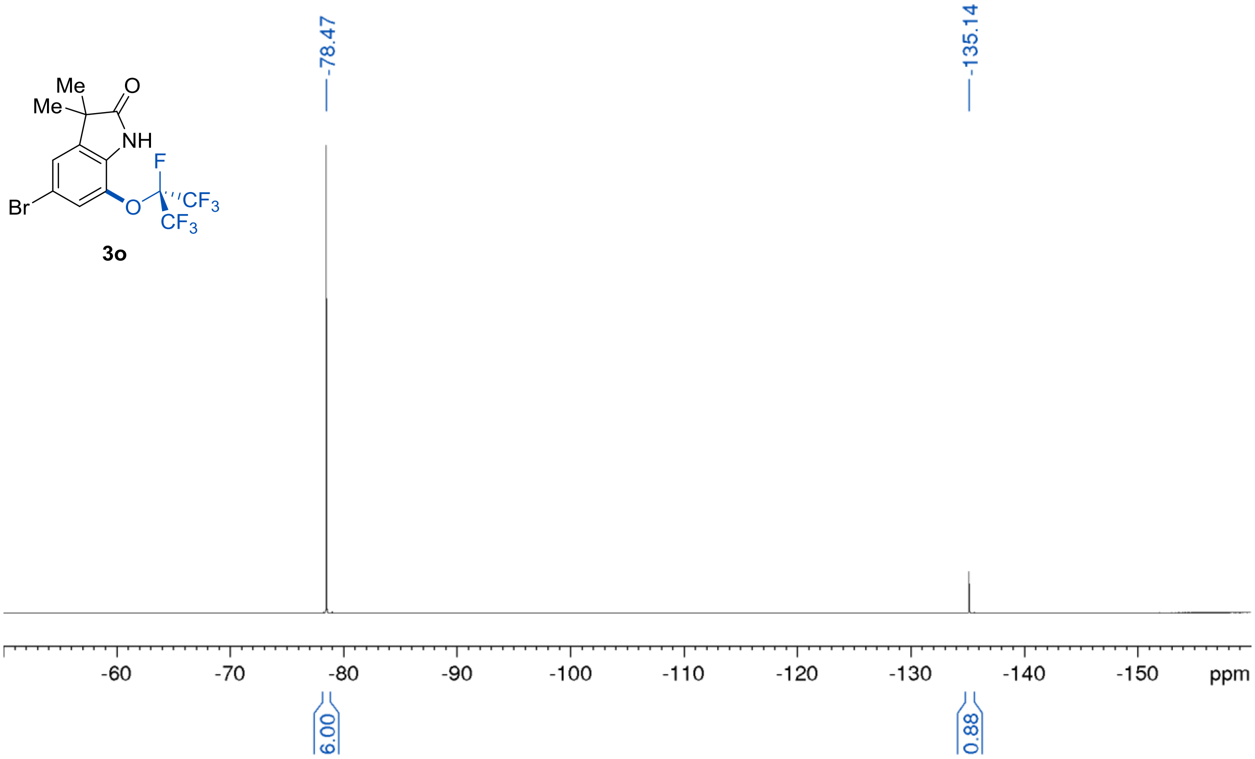

^{1}H NMR (700 MHz, $CDCl_3$, 25 ºC) of (3p)

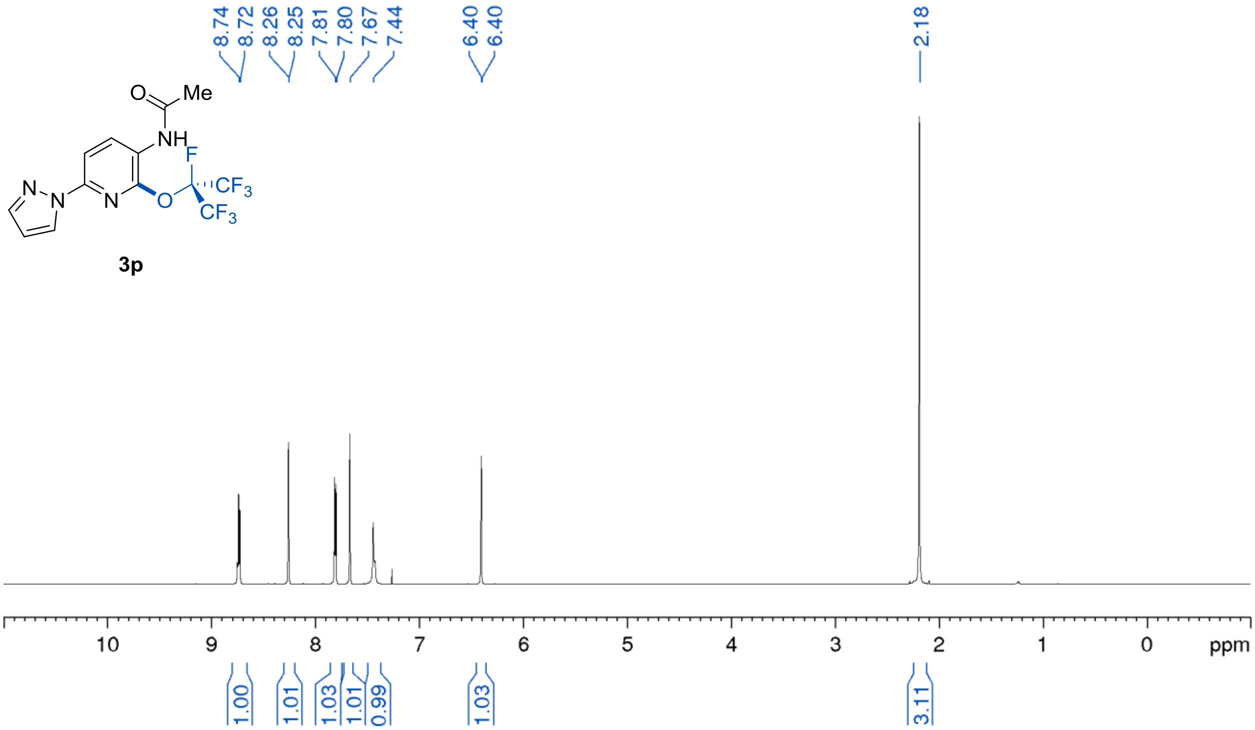

^{13}C NMR (175 MHz, $CDCl_3$, 25 ºC) of (3p)

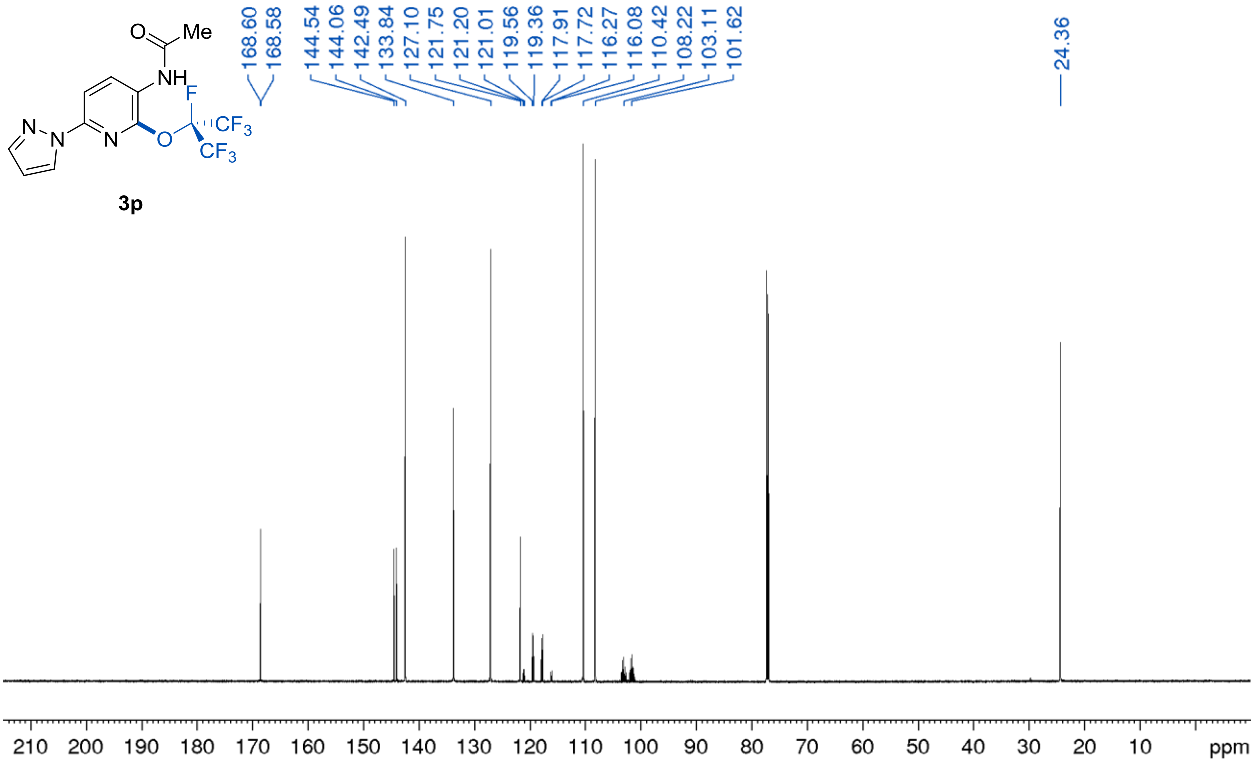

^{19}F NMR (376 MHz, $CDCl_3$, 25 ºC) of (3p)

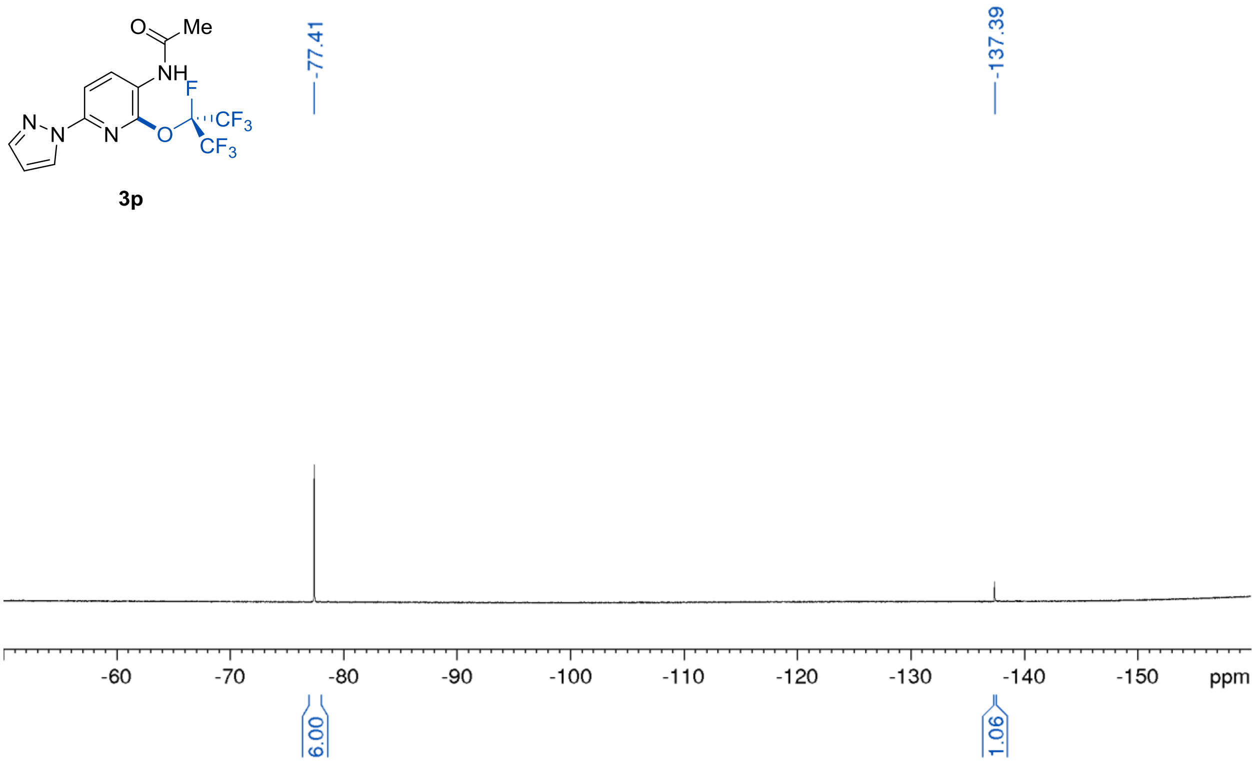

^{1}H NMR (700 MHz, $CDCl_3$, 25 ºC) of (3q)

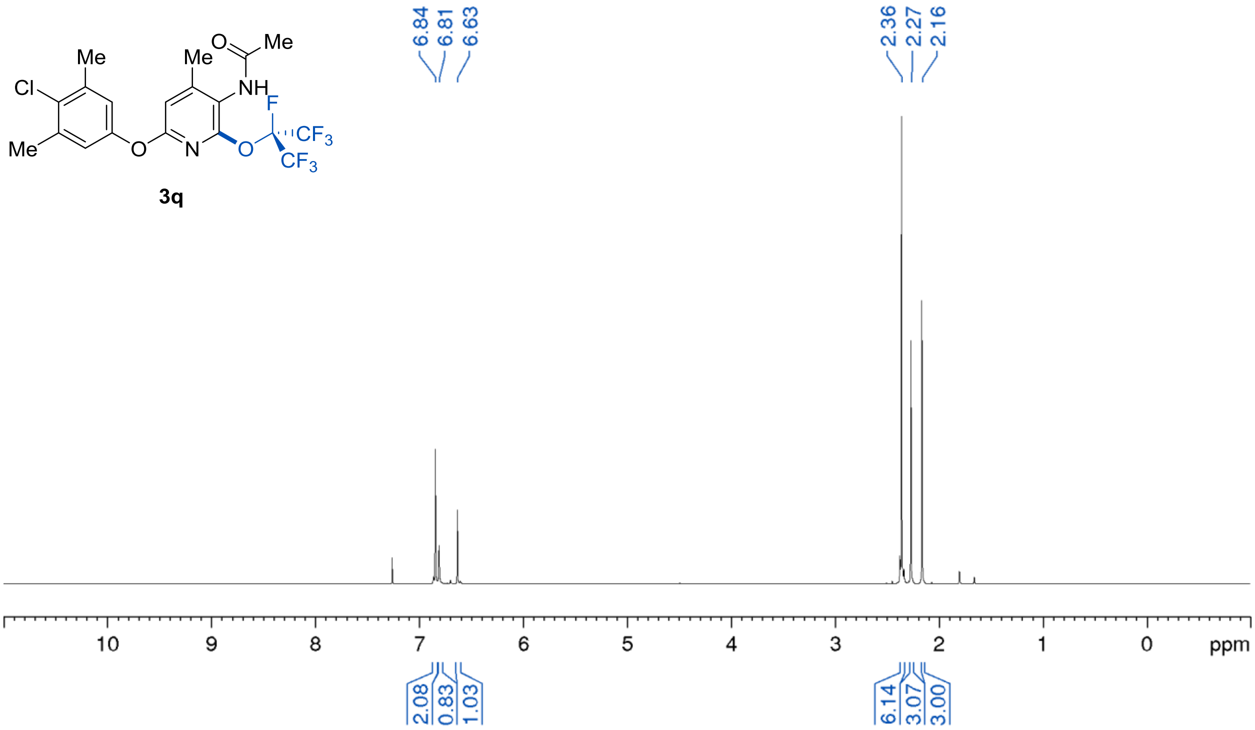

^{13}C NMR (175 MHz, $CDCl_3$, 25 ºC) of (3q)

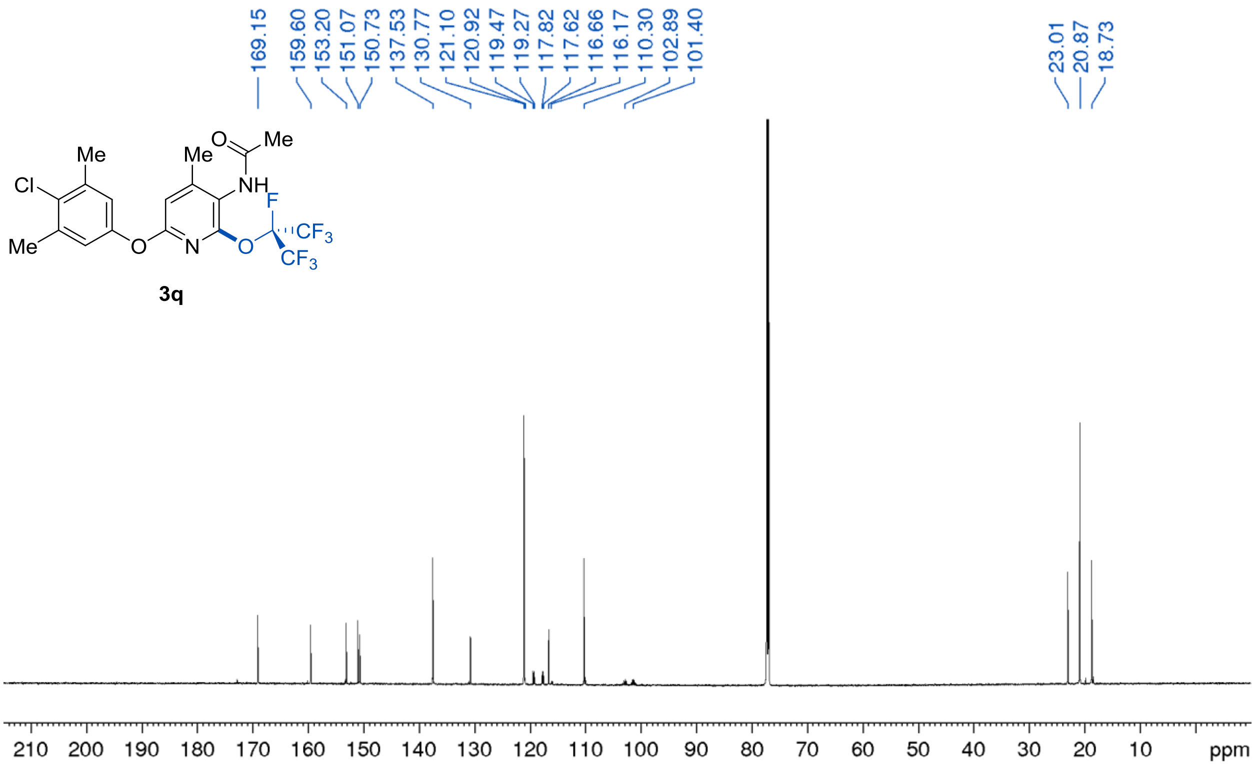

^{19}F NMR (376 MHz, $CDCl_3$, 25 °C) of (3q)

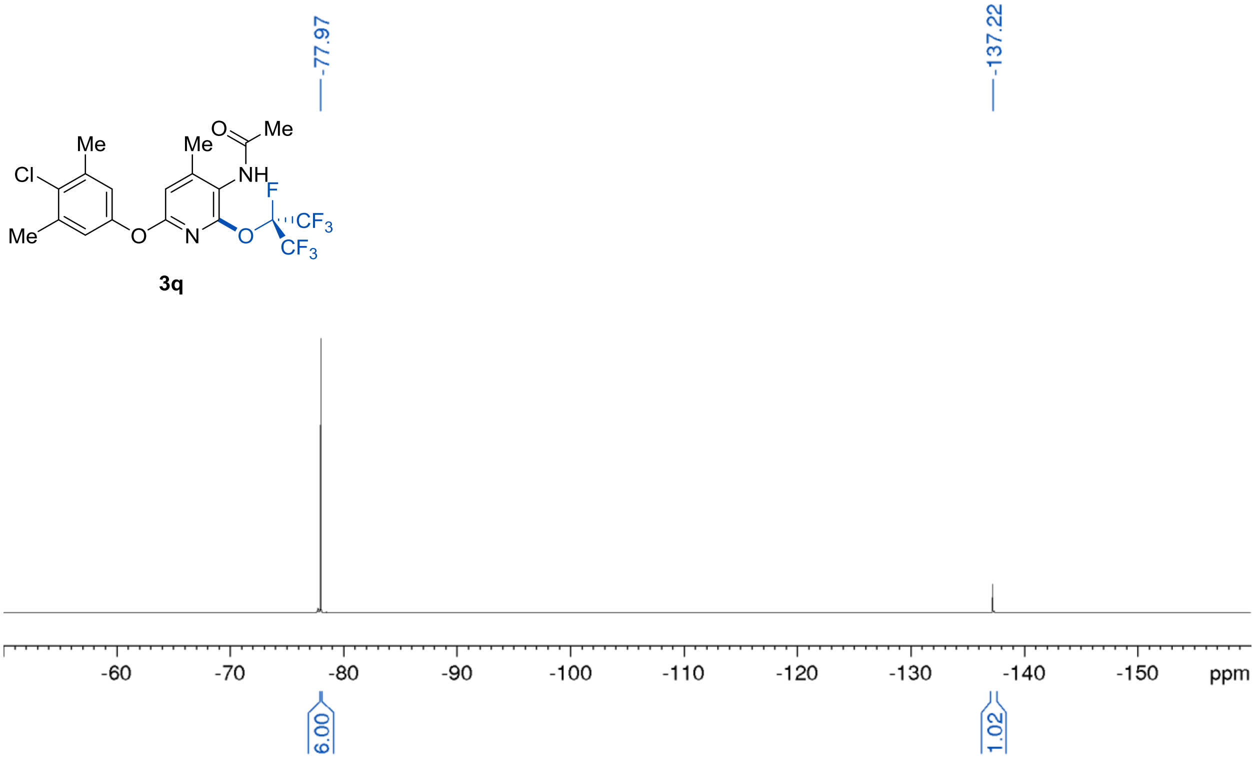

^{1}H NMR (700 MHz, $CDCl_3$, 60 °C) of (3r)

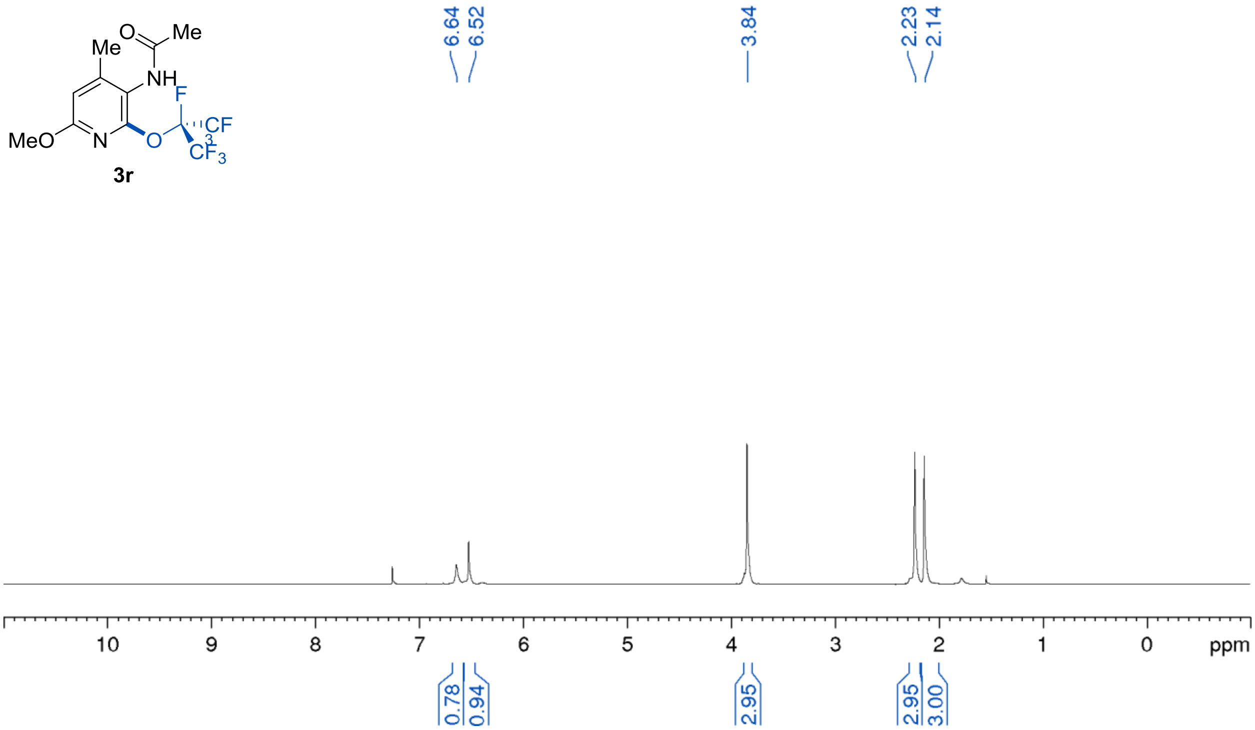

^{13}C NMR (175 MHz, $CDCl_3$, 60 ºC) of (3r)

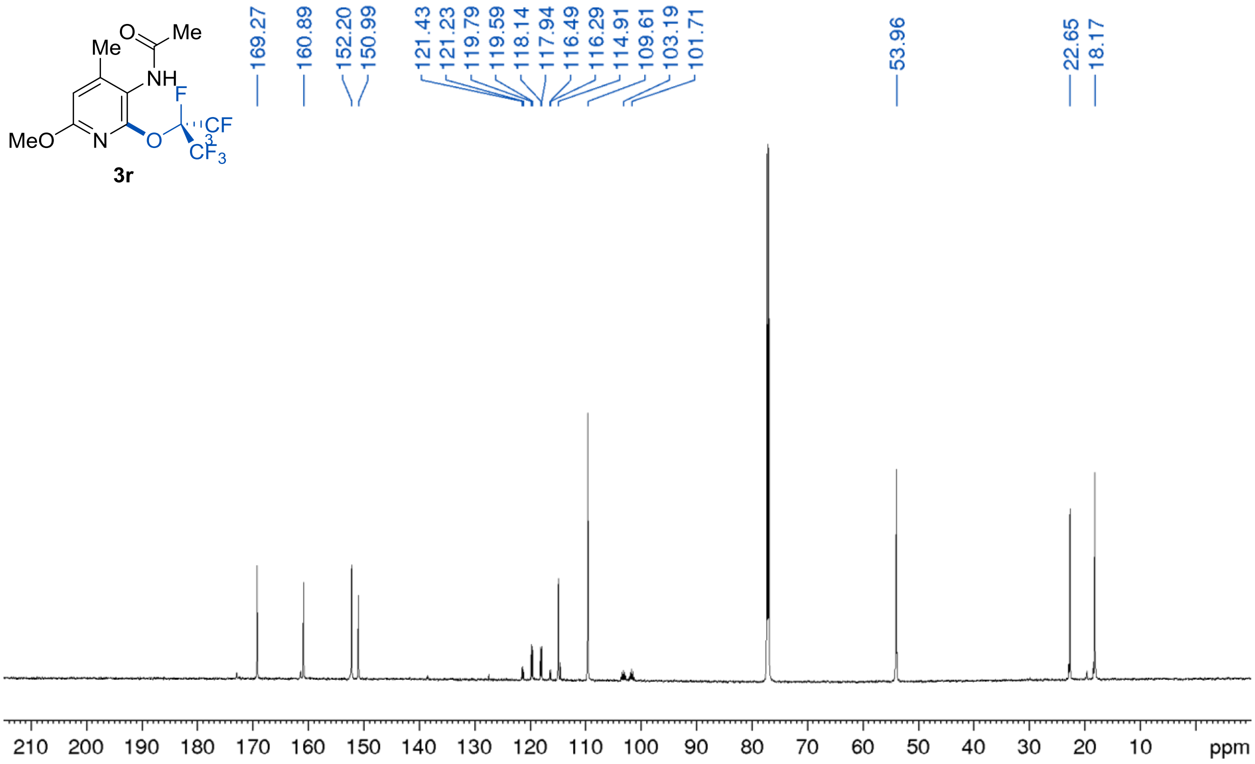

^{19}F NMR (376 MHz, $CDCl_3$, 60 ºC) of (3r)

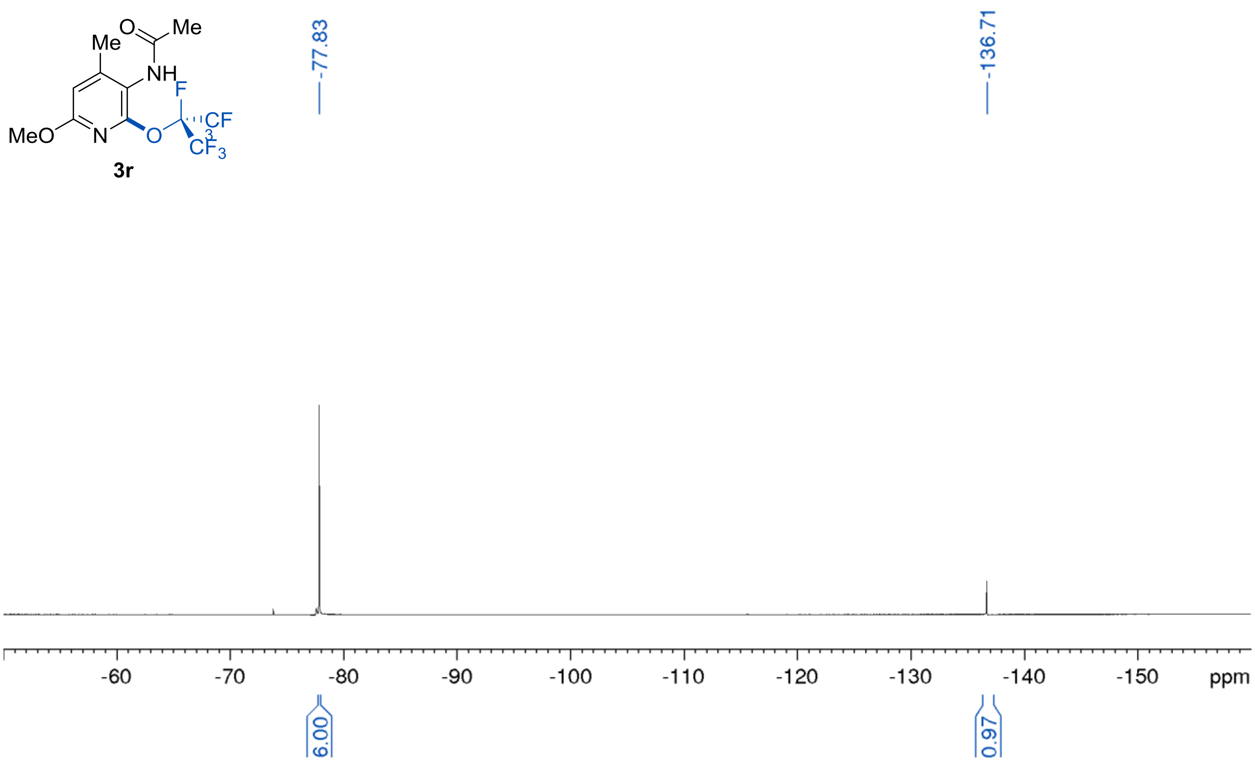

^{1}H NMR (700 MHz, $CDCl_3$, 25 ºC) of (3s)

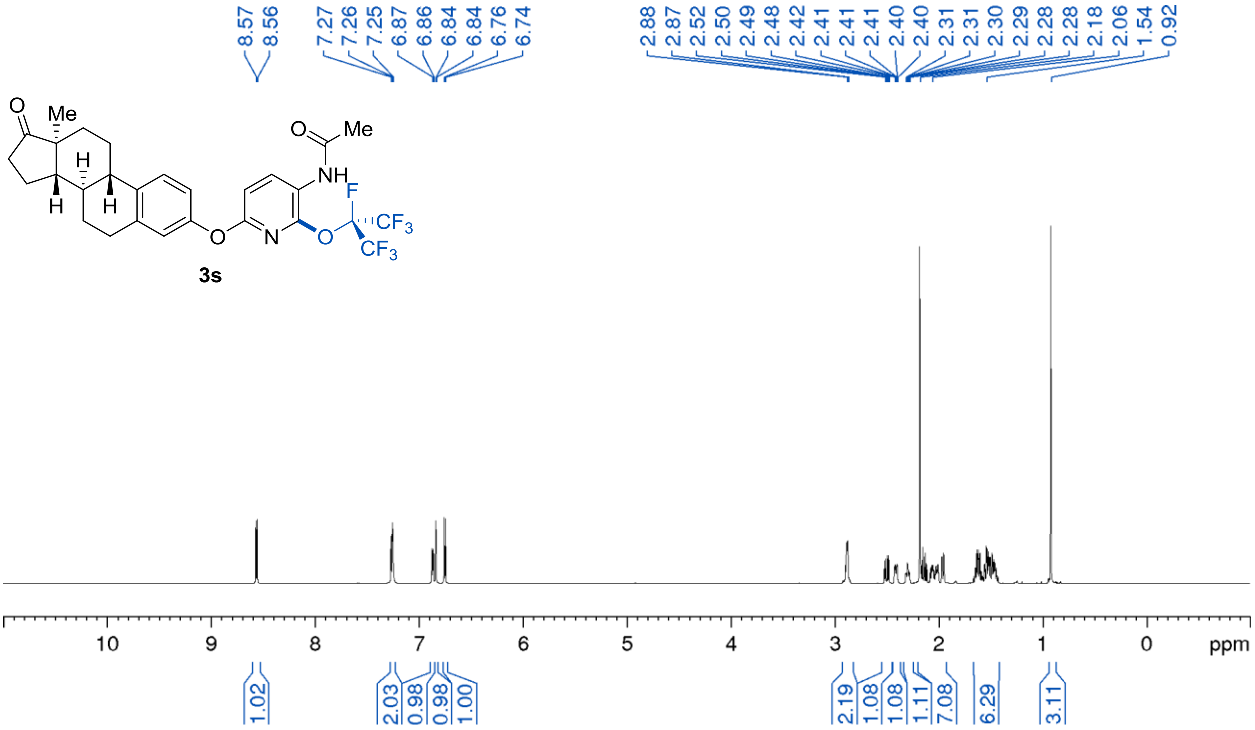

^{13}C NMR (175 MHz, $CDCl_3$, 25 ºC) of (3s)

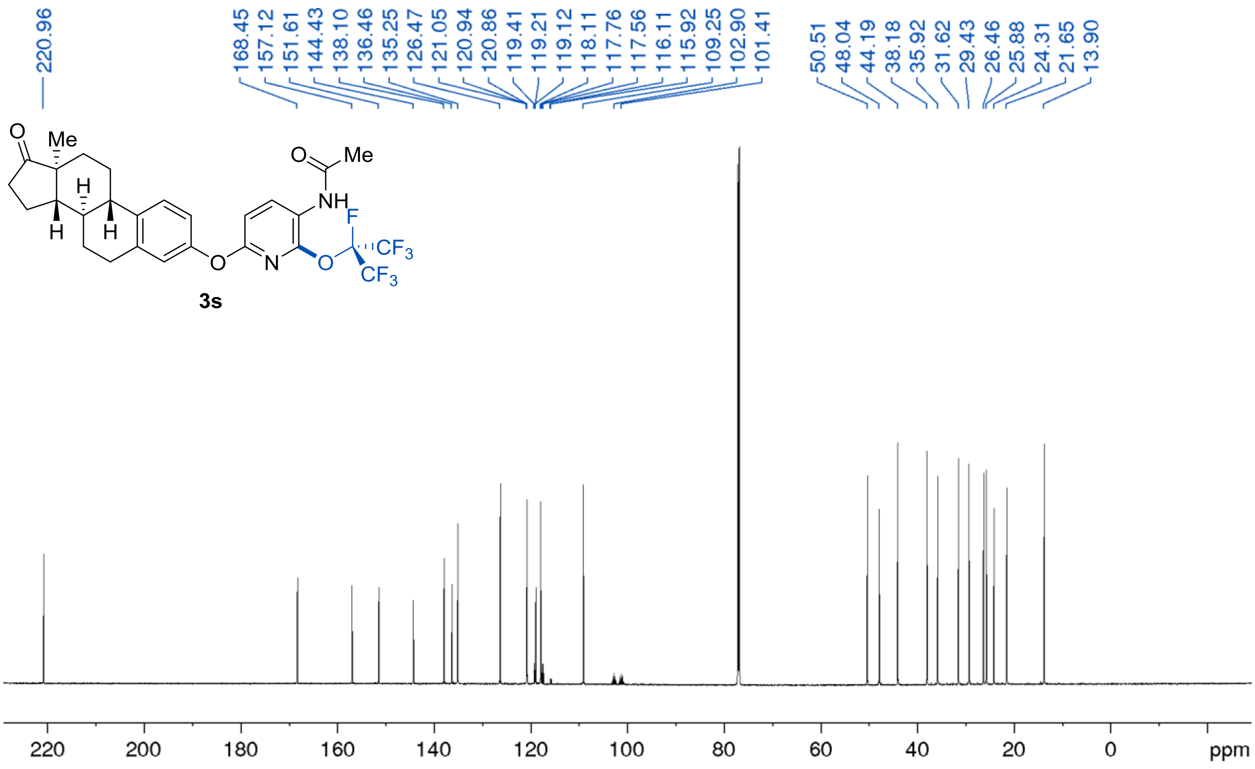

^{19}F NMR (376 MHz, $CDCl_3$, 25 ºC) of (3s)

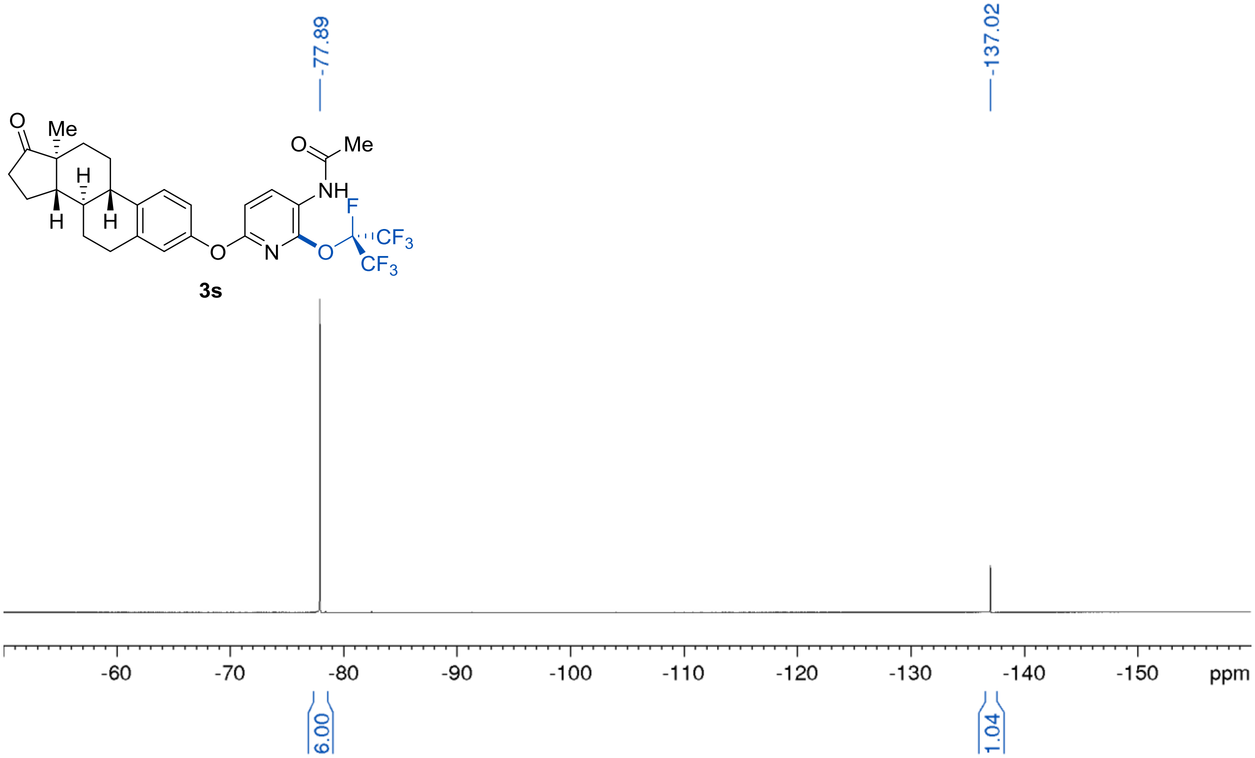

^{1}H NMR (700 MHz, $CDCl_3$, 25 ºC) of (3t)

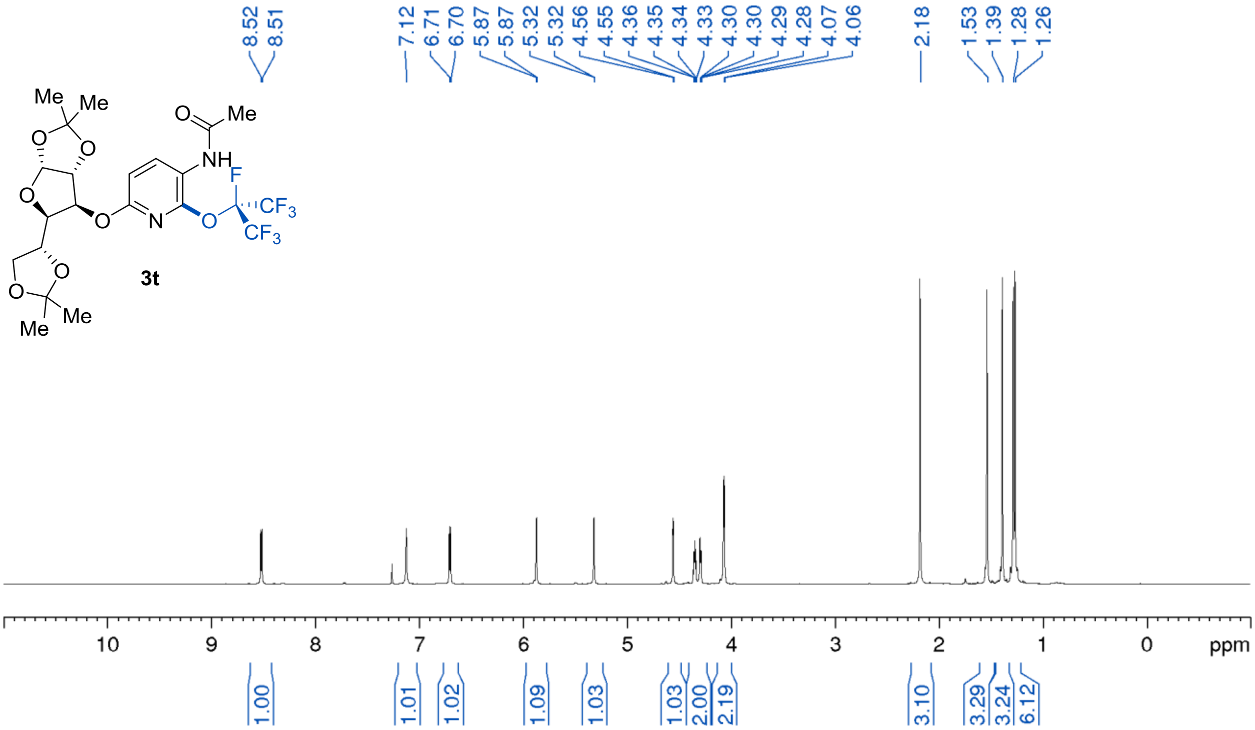

^{13}C NMR (175 MHz, $CDCl_3$, 25 ºC) of (3t)

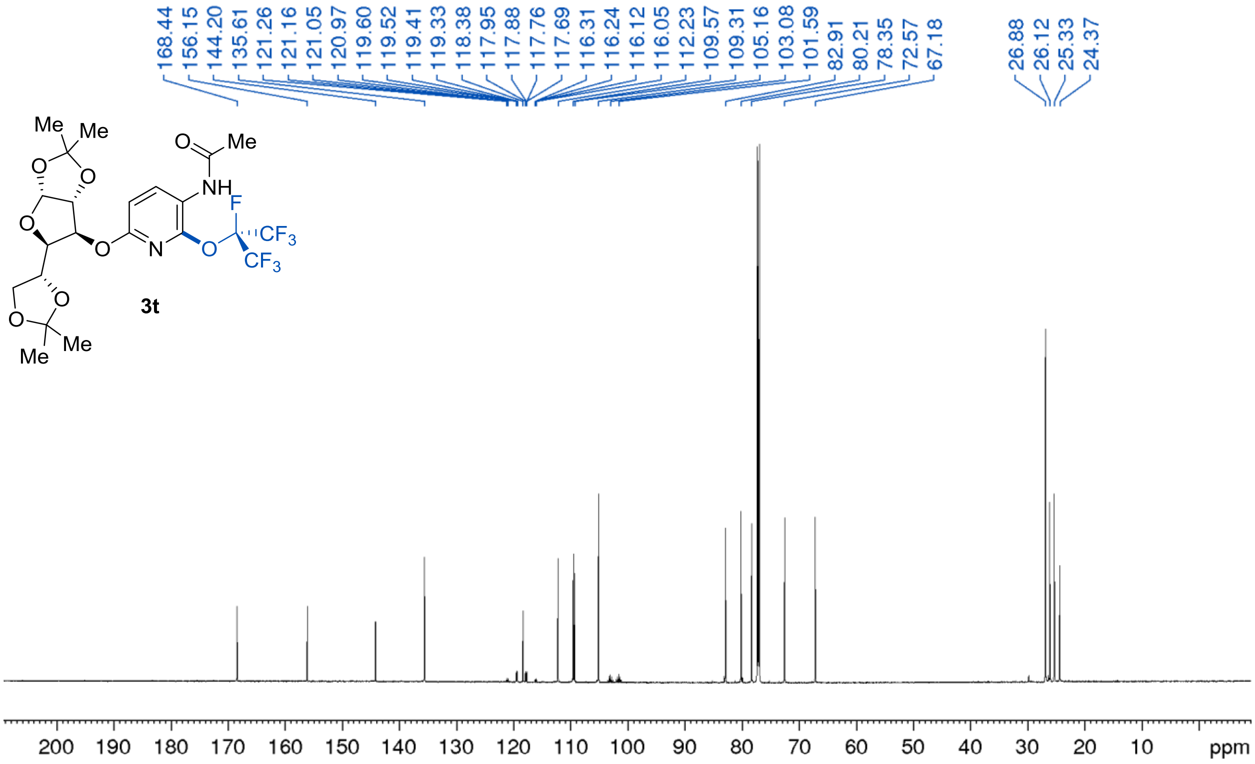

^{19}F NMR (376 MHz, $CDCl_3$, 25 ºC) of (3t)

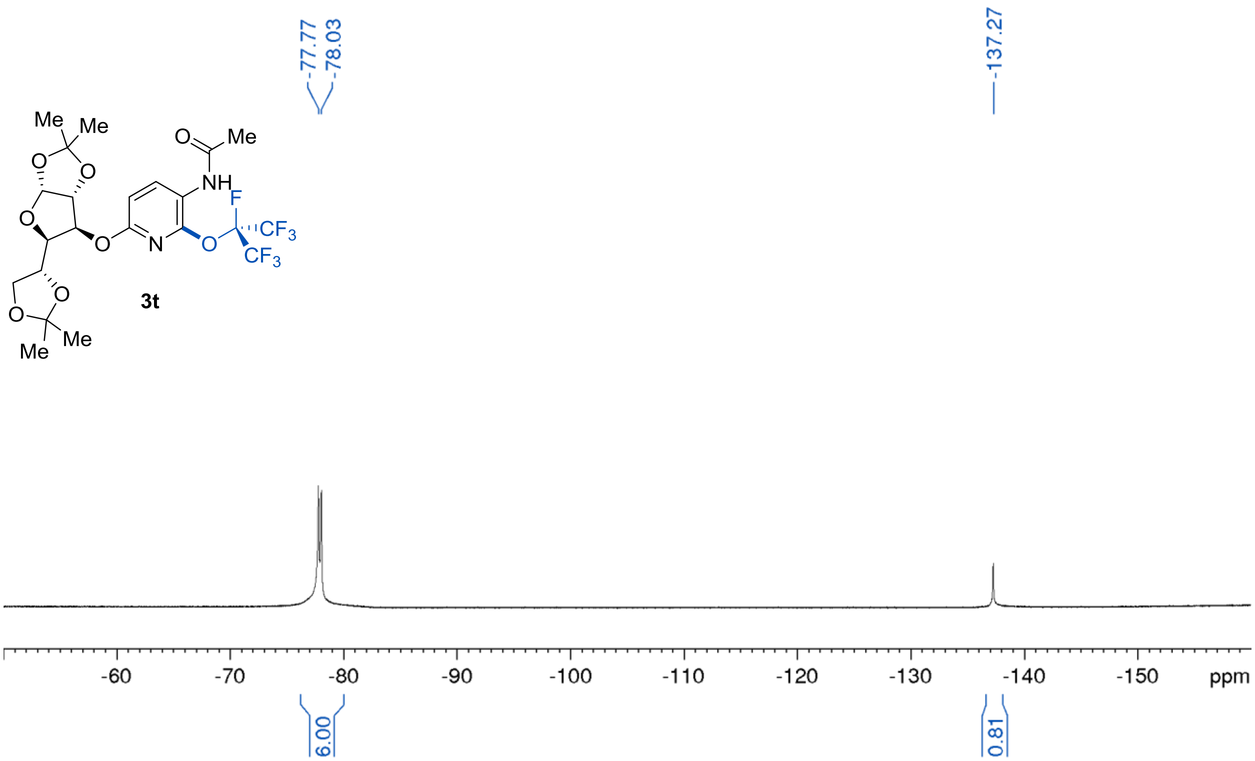

^{1}H NMR (700 MHz, $CDCl_3$, 25 ºC) of (4a)

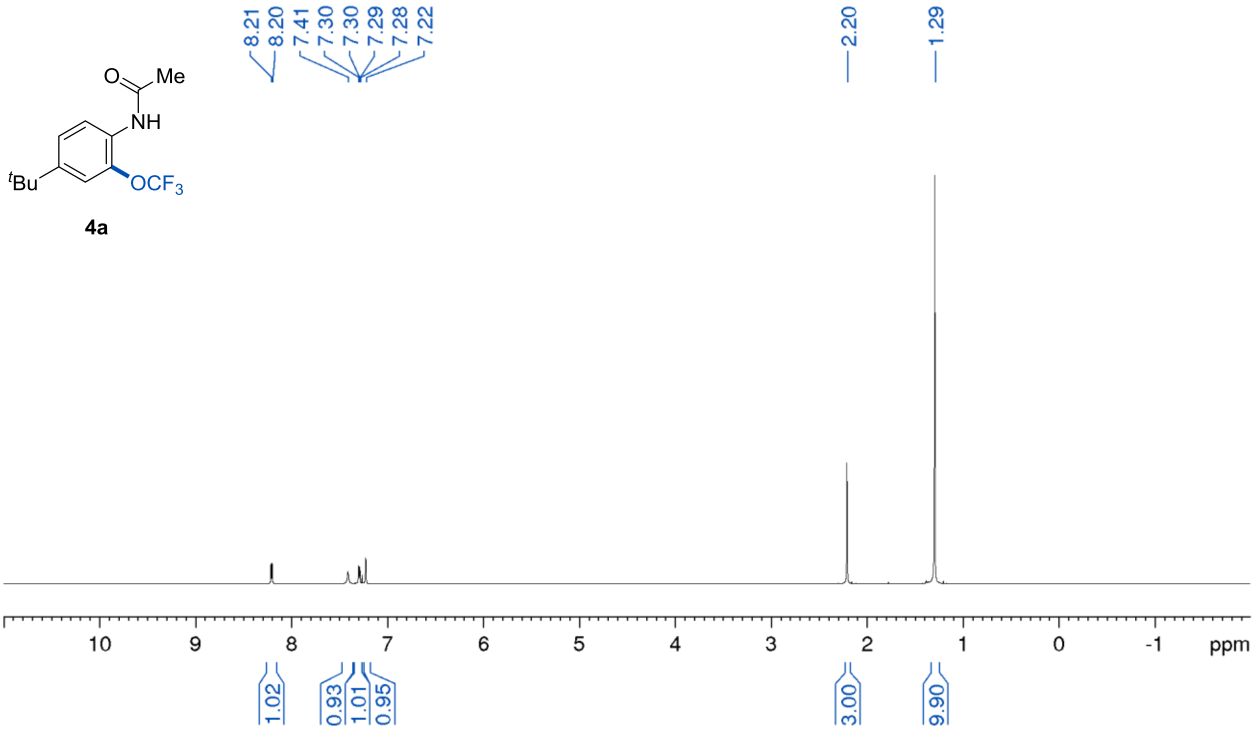

^{13}C NMR (175 MHz, $CDCl_3$, 25 ºC) of (4a)

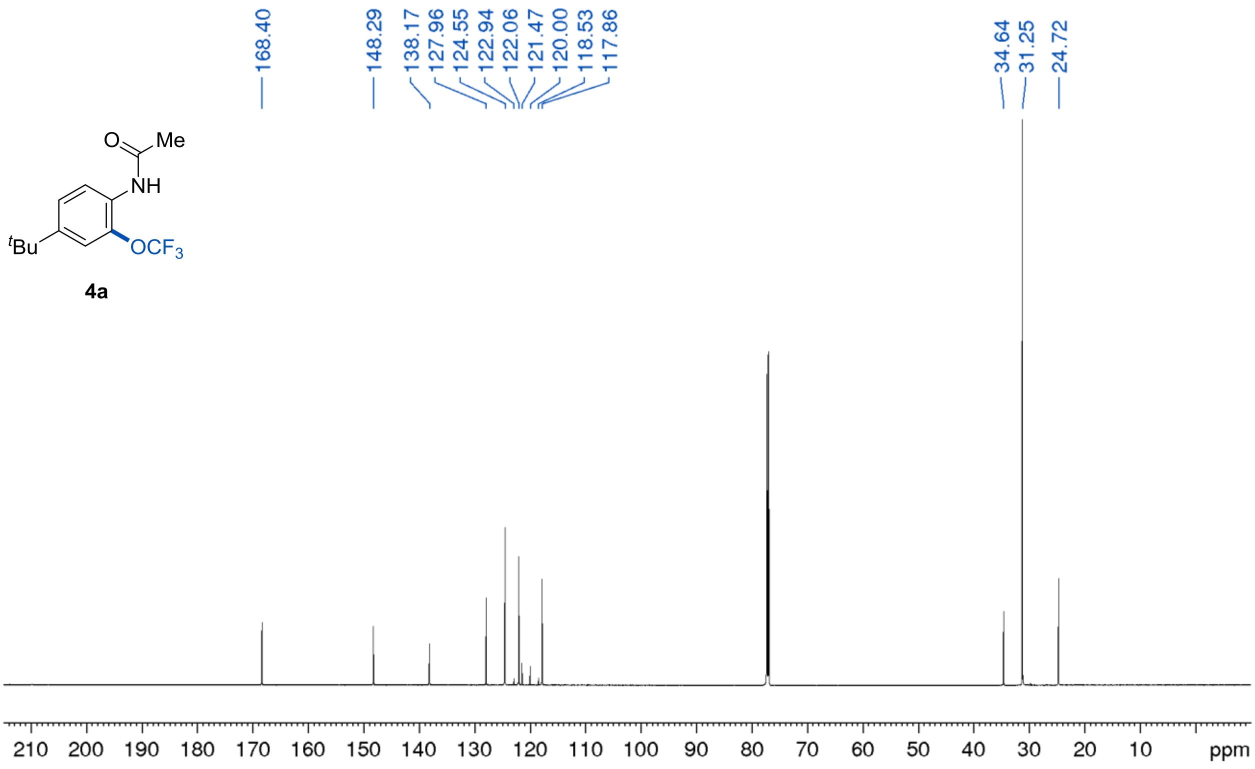

^{19}F NMR (376 MHz, $CDCl_3$, 25 ºC) of (4a)

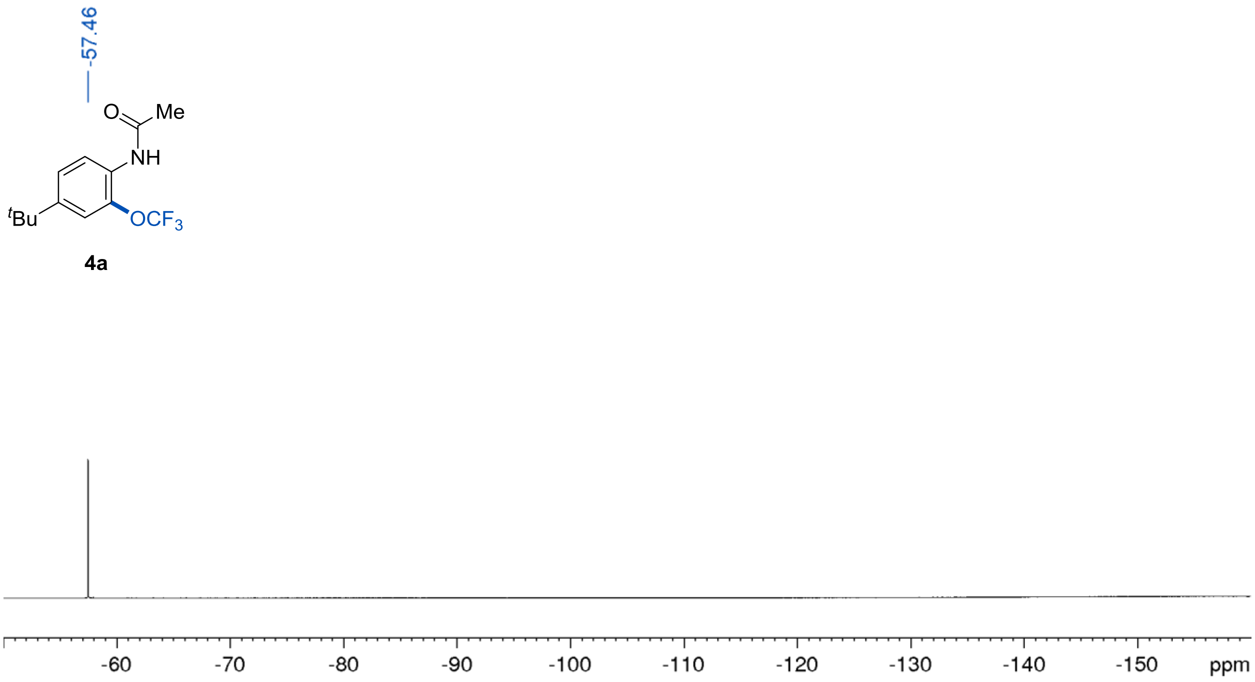

^{1}H NMR (700 MHz, $CDCl_3$, 25 ºC) of (4b)

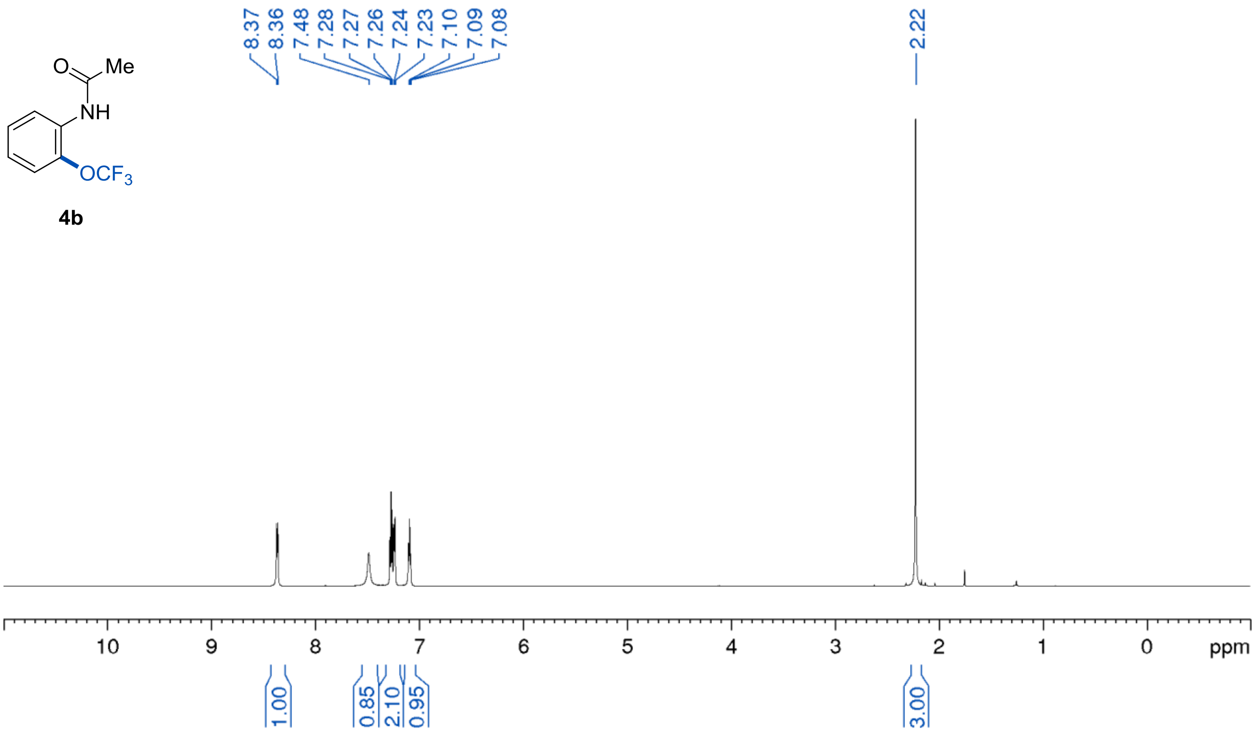

^{13}C NMR (175 MHz, $CDCl_3$, 25 ºC) of (4b)

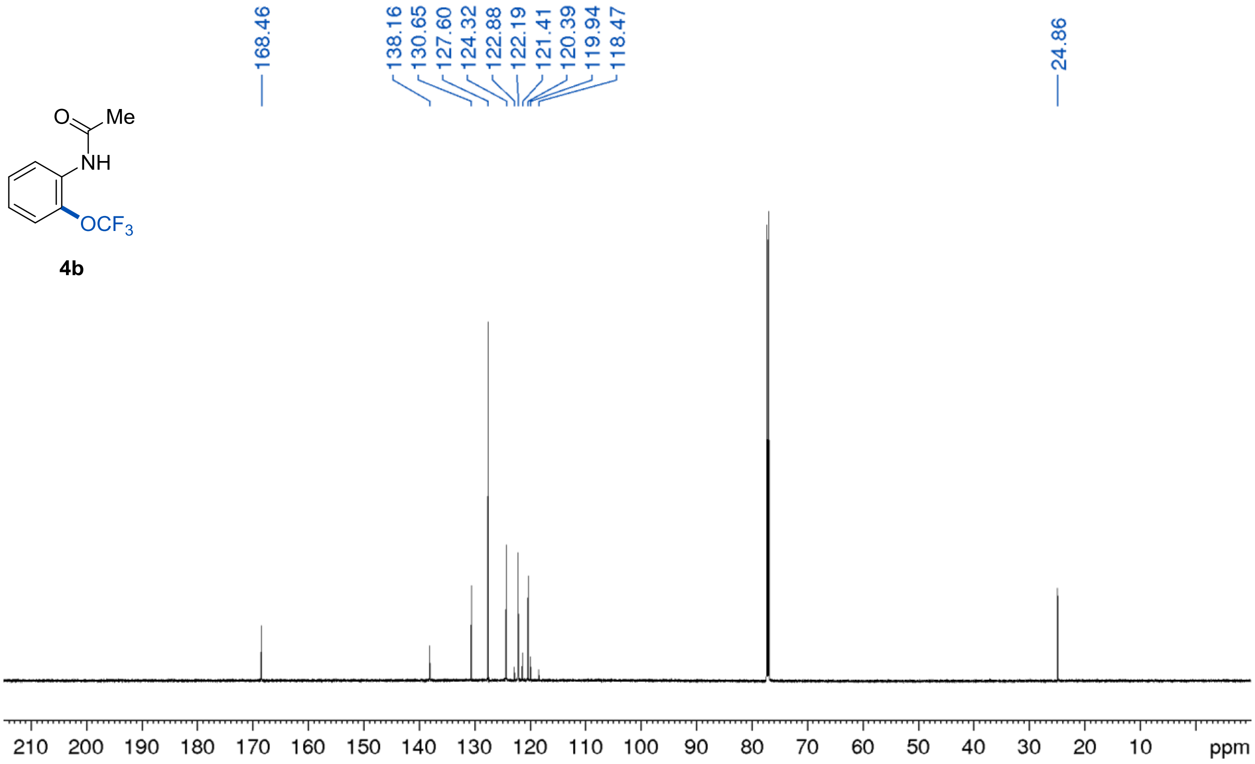

^{19}F NMR (376 MHz, $CDCl_3$, 25 ºC) of (4b)

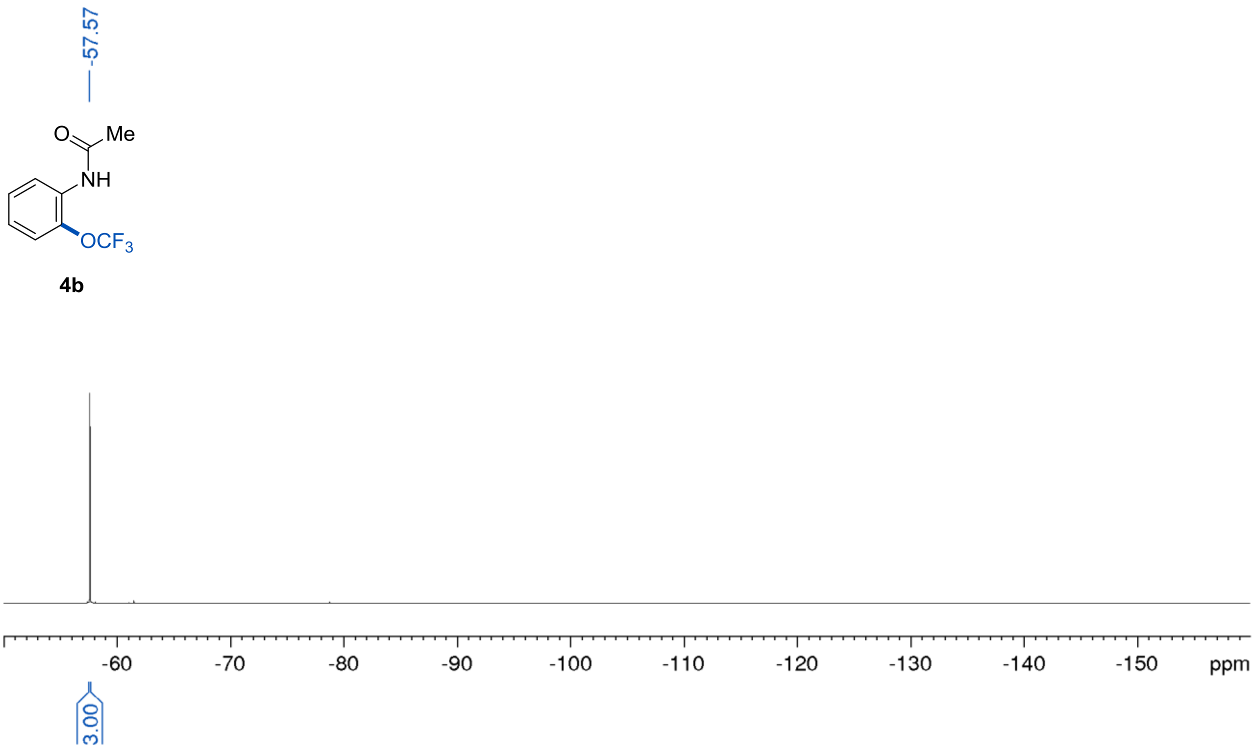

^{1}H NMR (700 MHz, CDCl$_3$, 25 °C) of (4c)

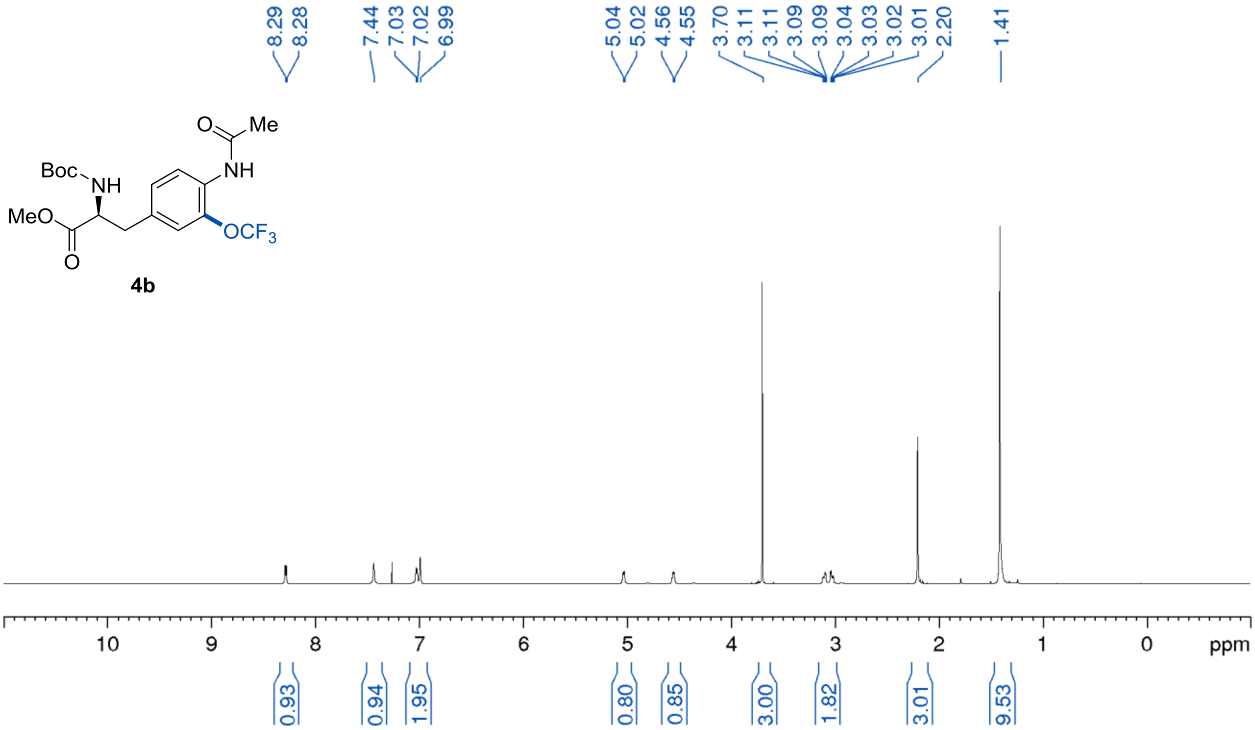

^{13}C NMR (175 MHz, CDCl$_3$, 25 °C) of (4c)

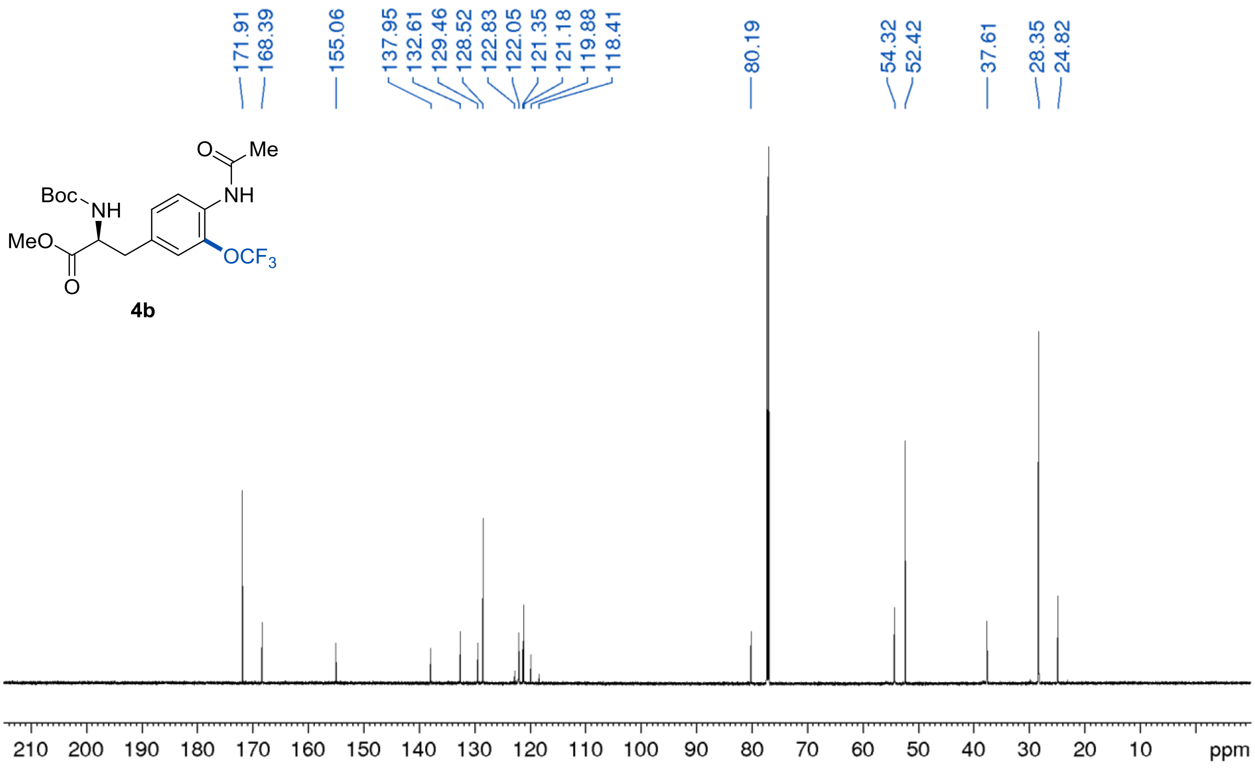

^{19}F NMR (376 MHz, $CDCl_3$, 25 ºC) of (4c)

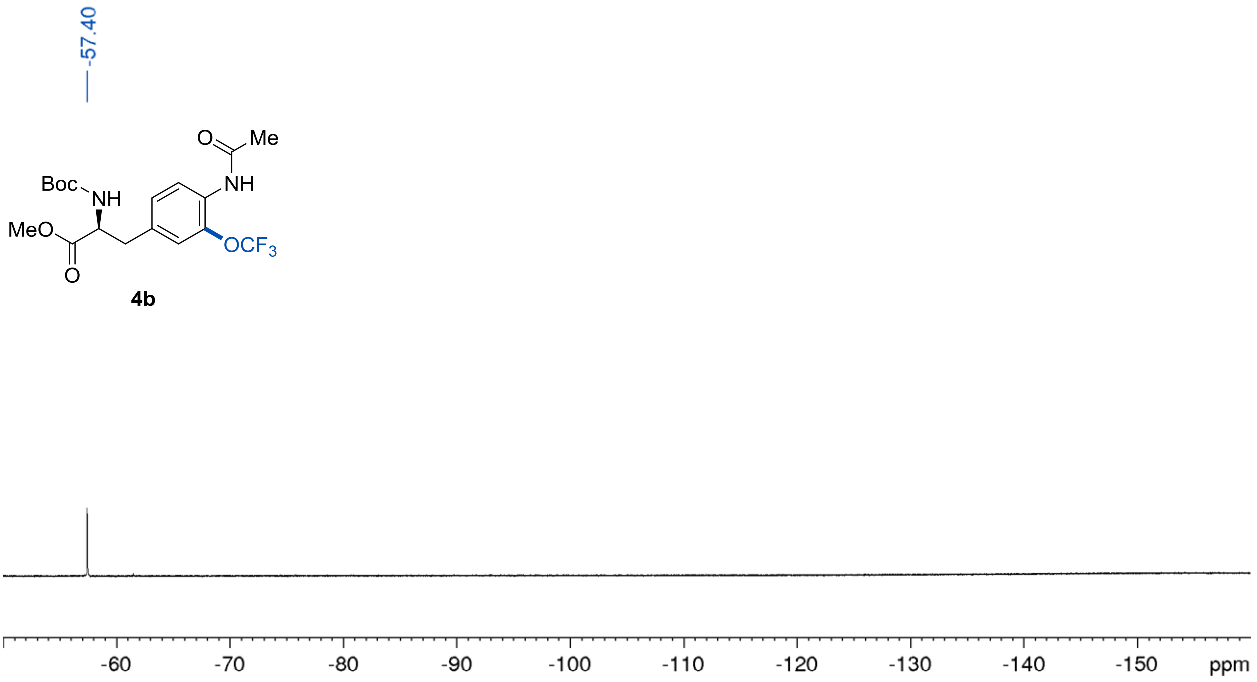

^{1}H NMR (700 MHz, $CDCl_3$, 25 ºC) of (4d)

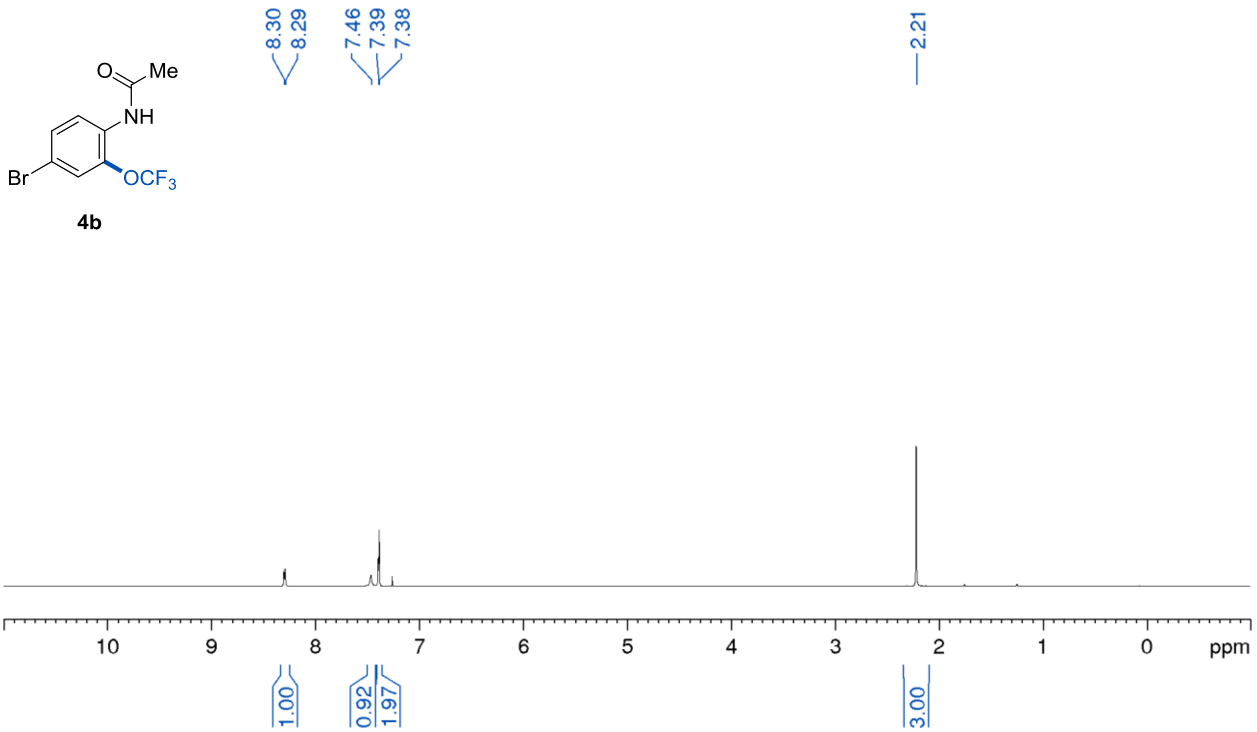

^{13}C NMR (175 MHz, $CDCl_3$, 25 ºC) of (4d)

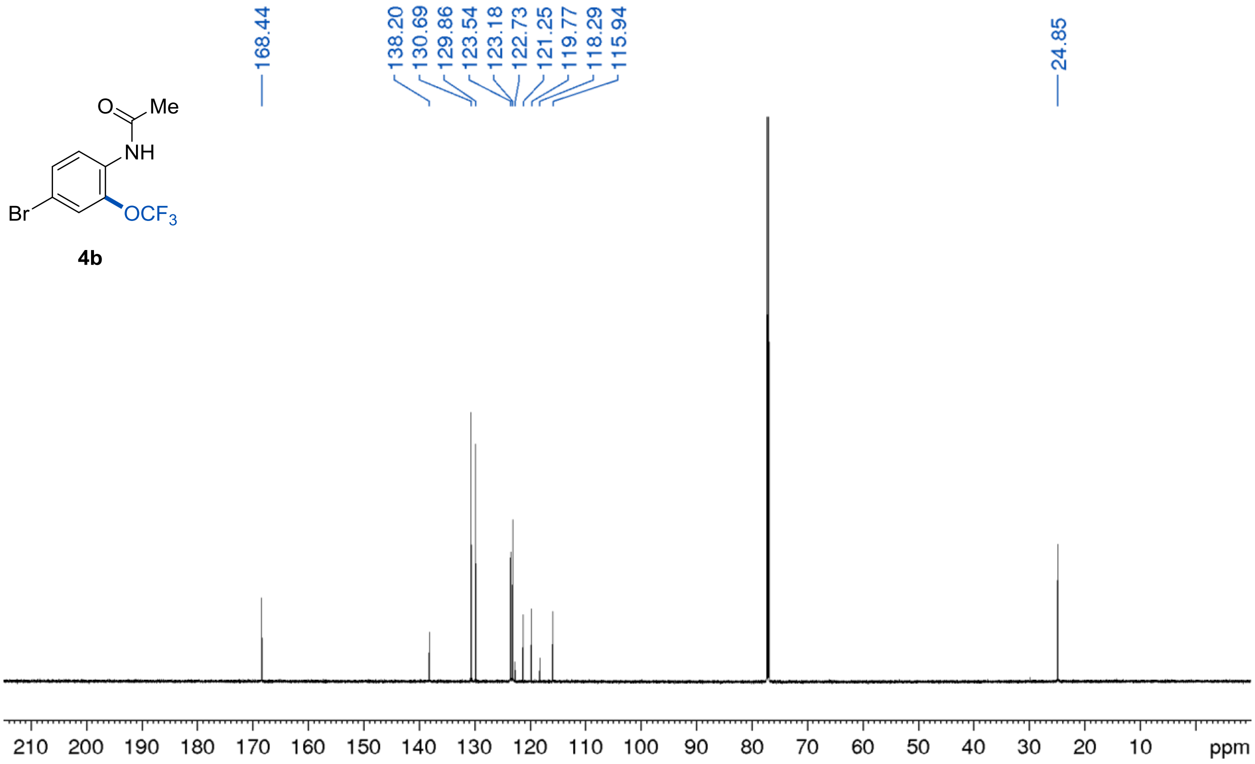

^{19}F NMR (376 MHz, $CDCl_3$, 25 ºC) of (4d)

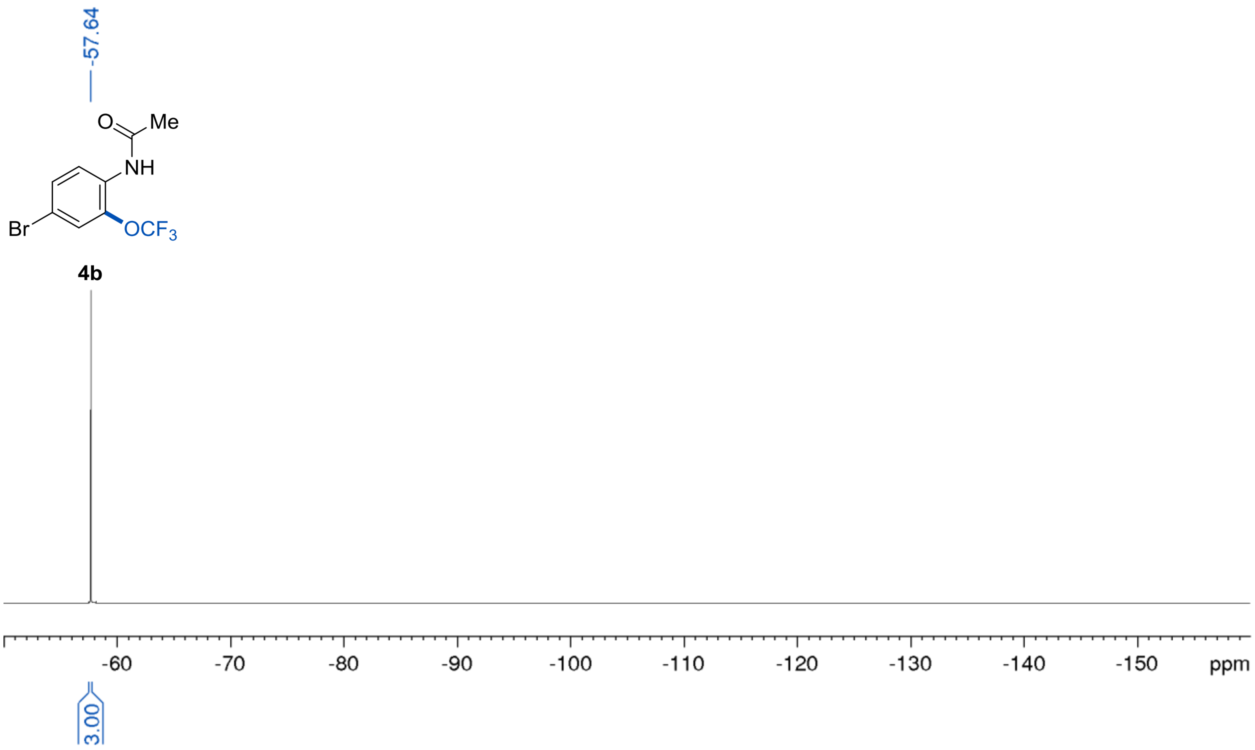

^{1}H NMR (700 MHz, CDCl$_3$, 25 ºC) of (4e)

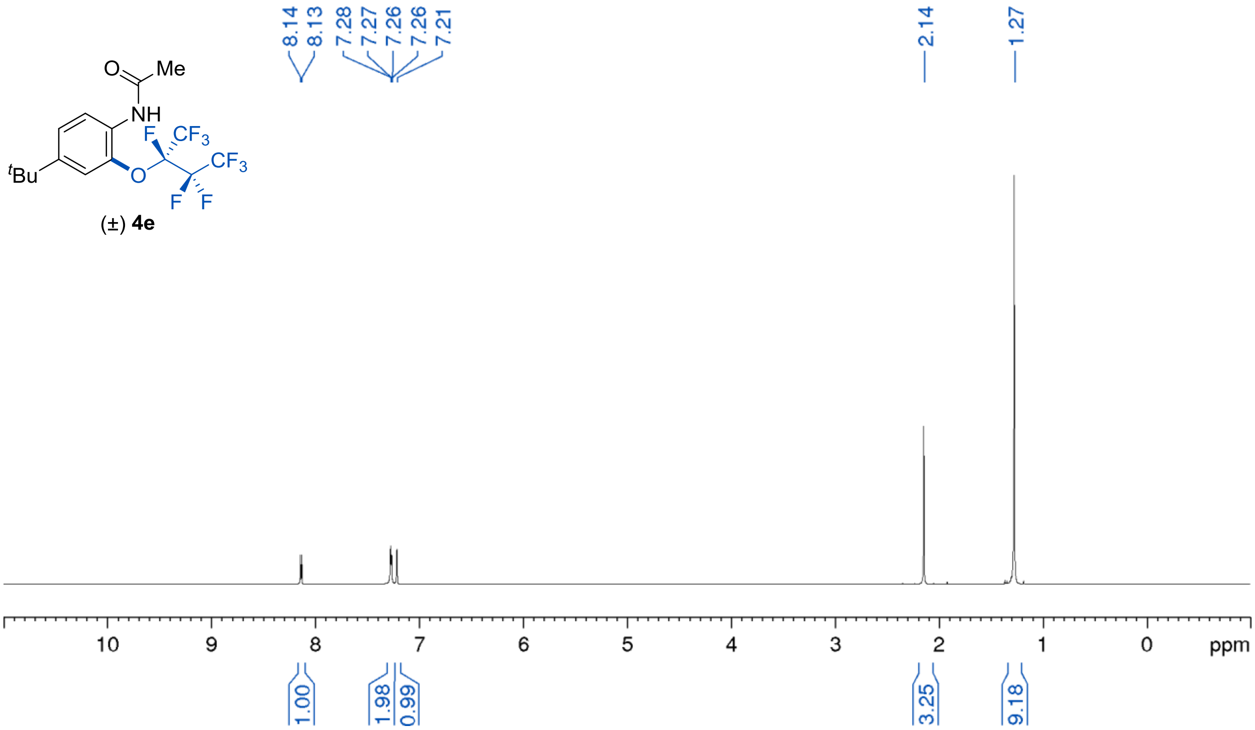

^{13}C NMR (175 MHz, CDCl$_3$, 25 ºC) of (4e)

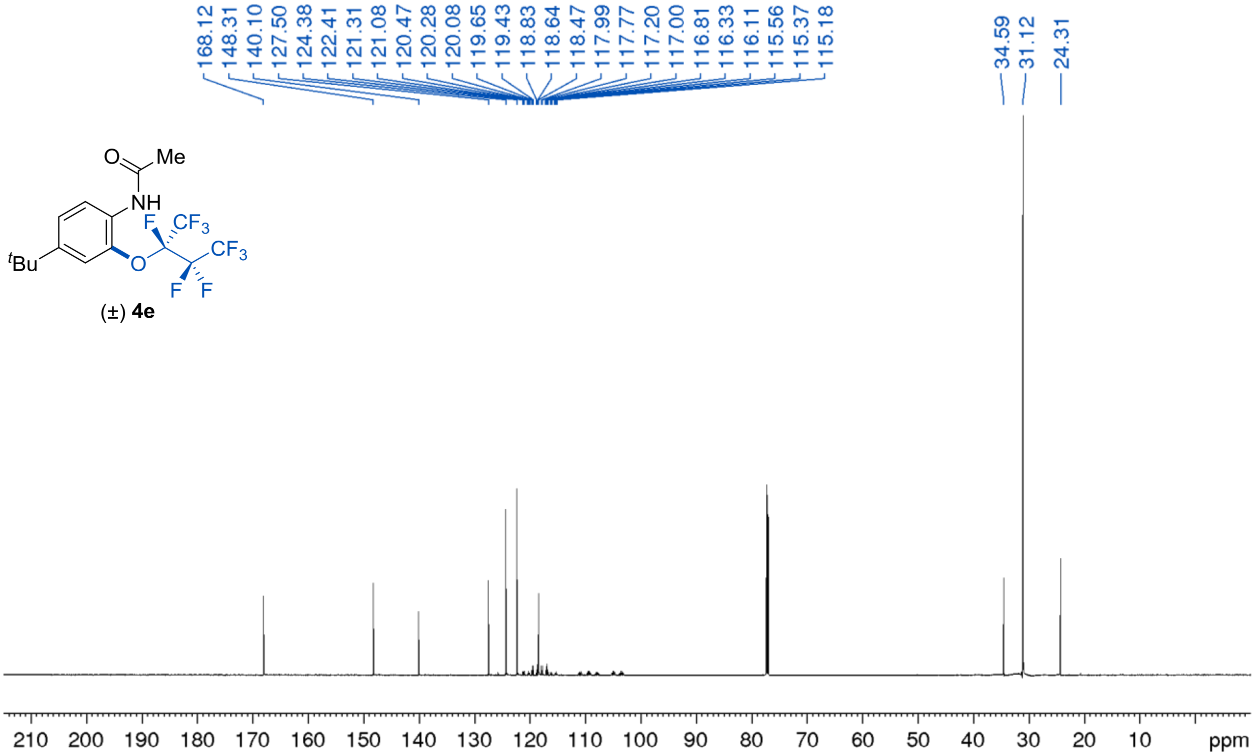

^{19}F NMR (376 MHz, $CDCl_3$, 25 ºC) of (4e)

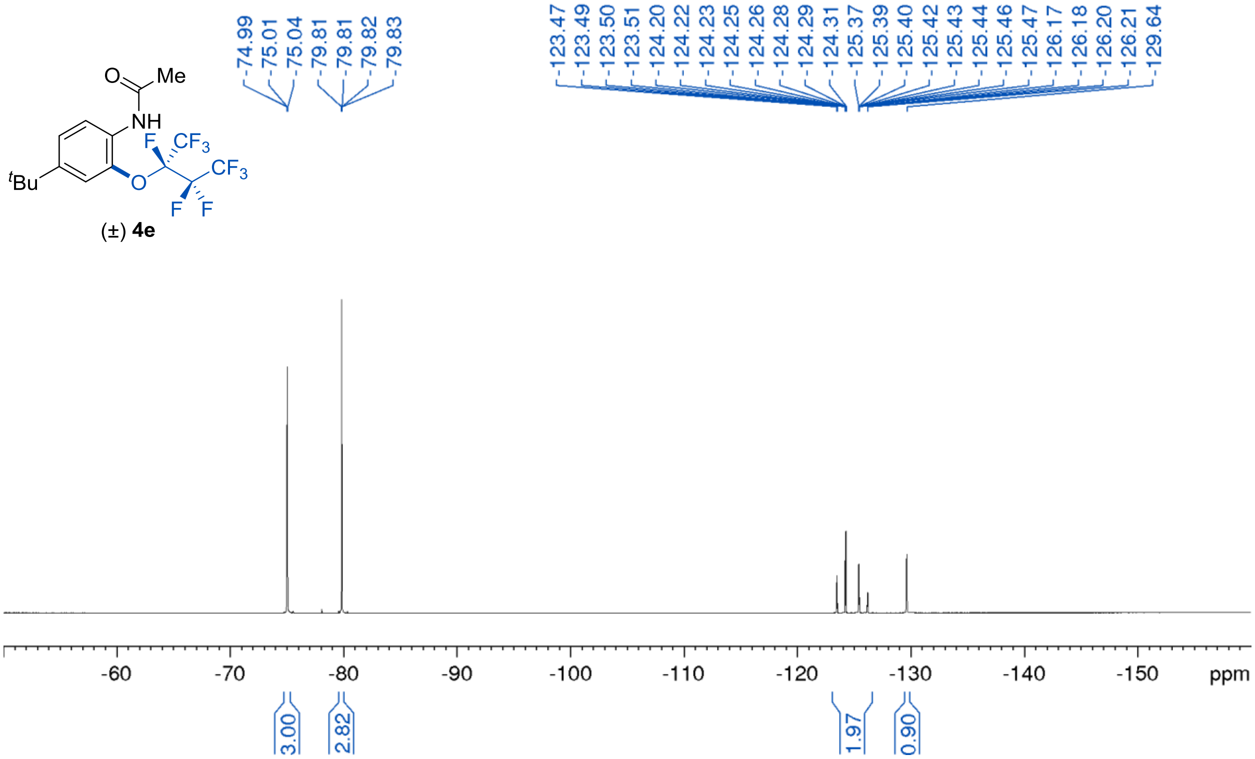

^{1}H NMR (700 MHz, $CDCl_3$, 25 ºC) of (4f)

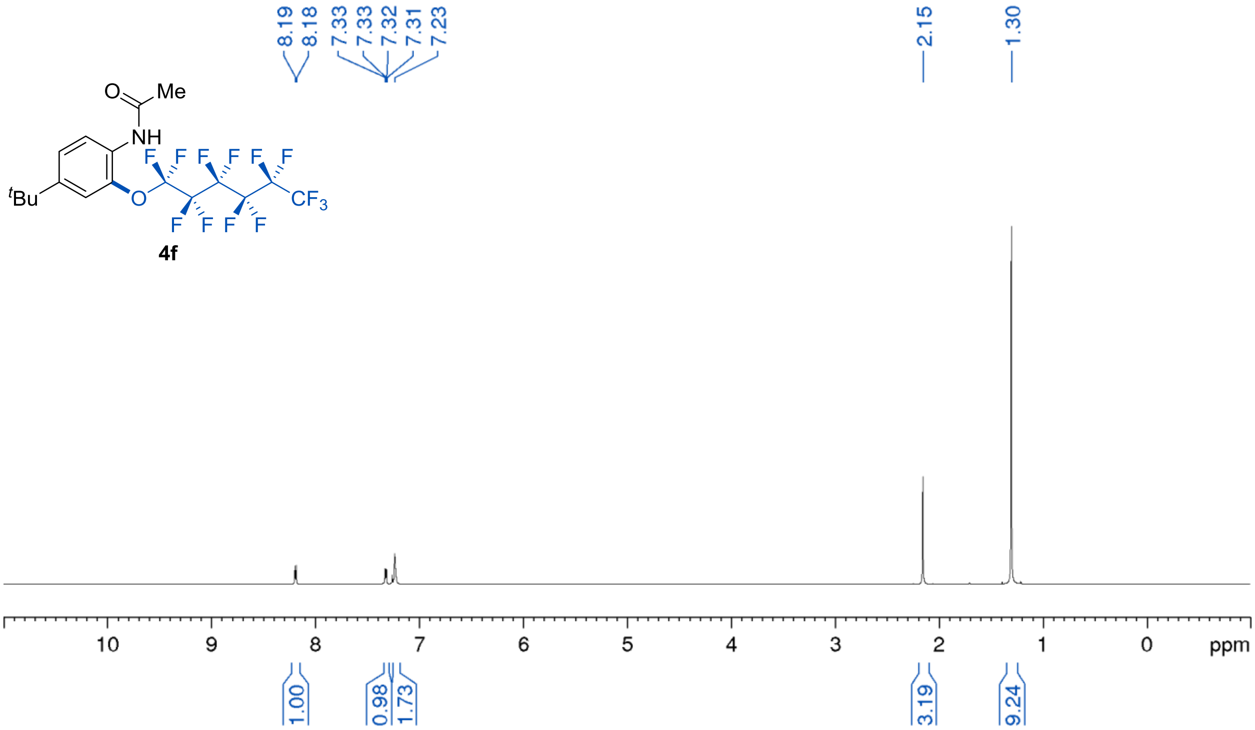

^{13}C NMR (175 MHz, CDCl$_3$, 25 ºC) of (4f)

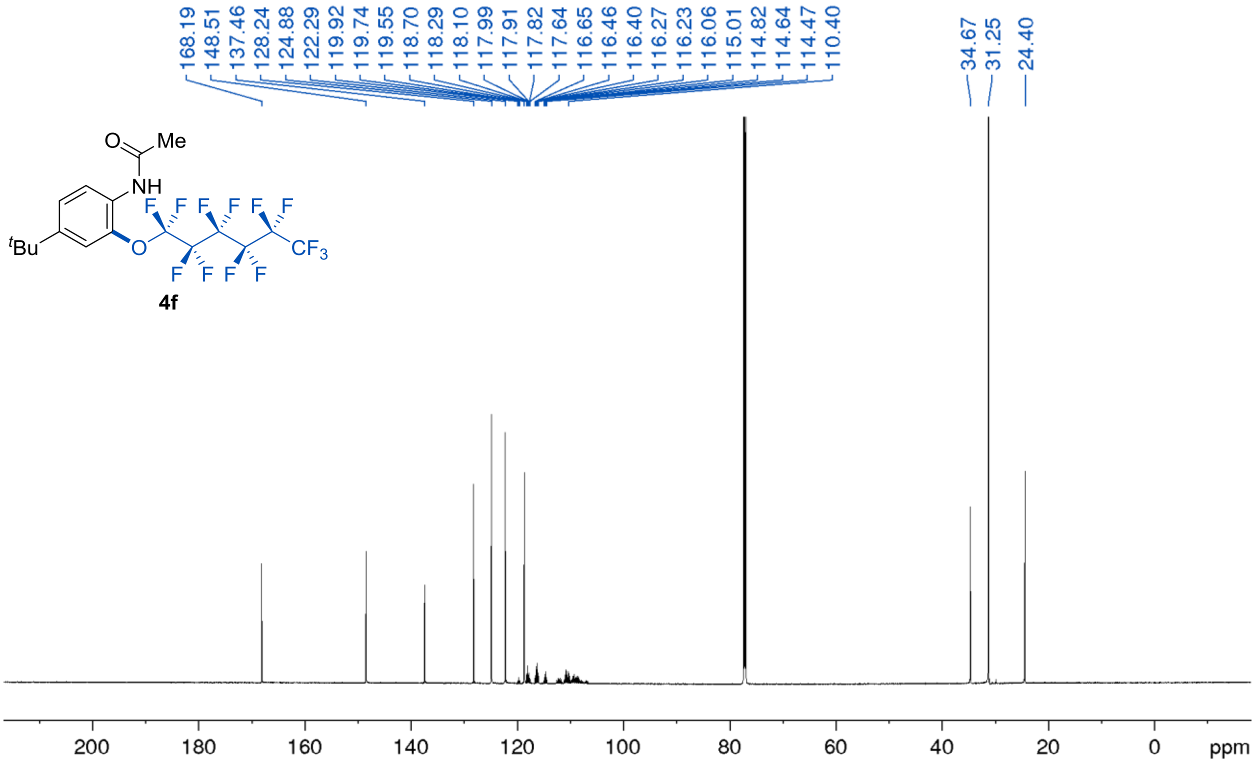

^{19}F NMR (376 MHz, CDCl$_3$, 25 ºC) of (4f)

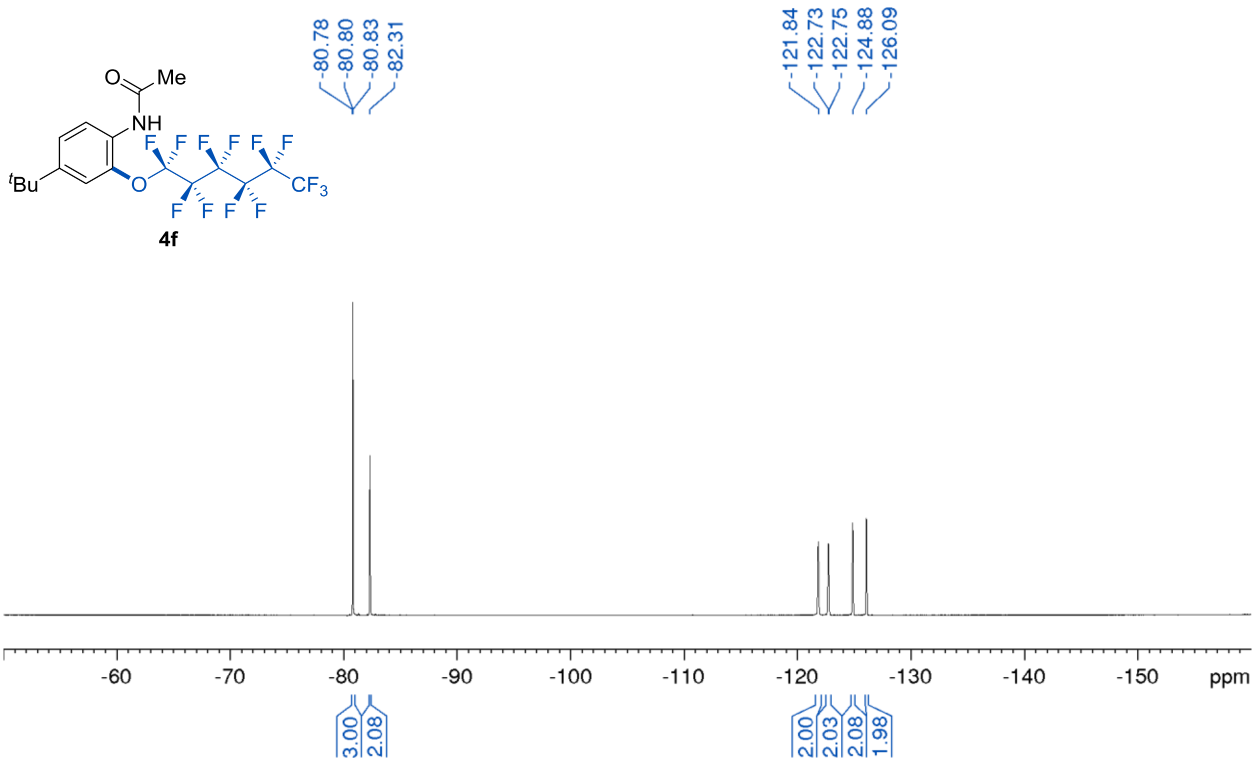

^{1}H NMR (700 MHz, $CDCl_3$, 25 ºC) of (4g)

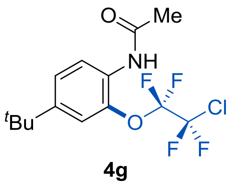

4g

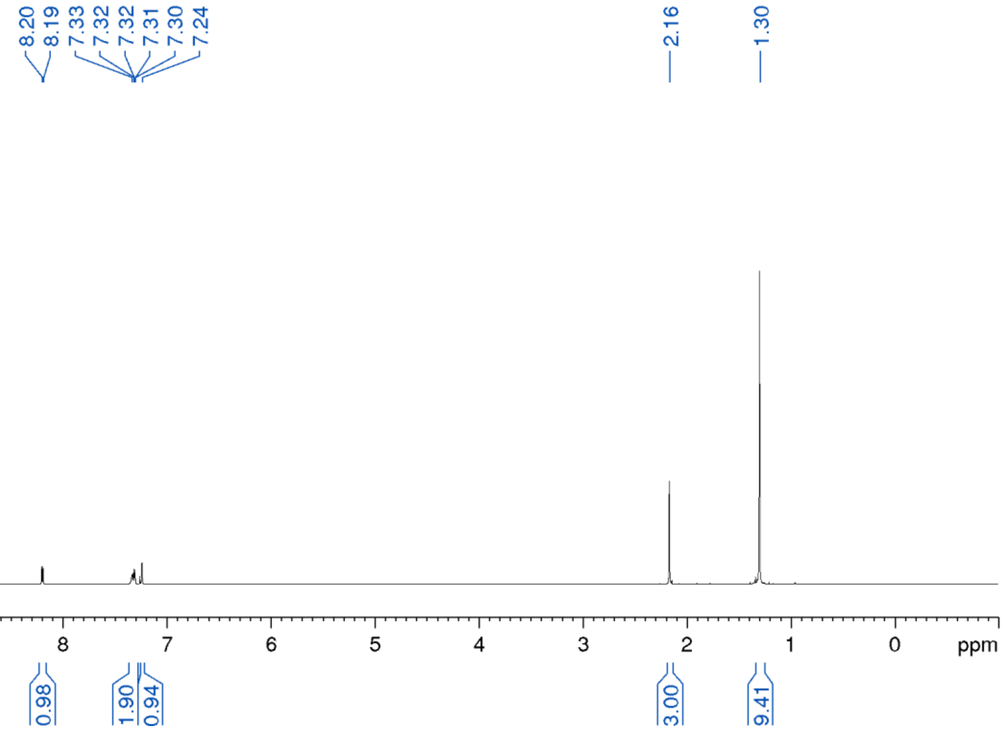

^{13}C NMR (175 MHz, $CDCl_3$, 25 ºC) of (4g)

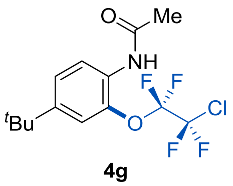

4g

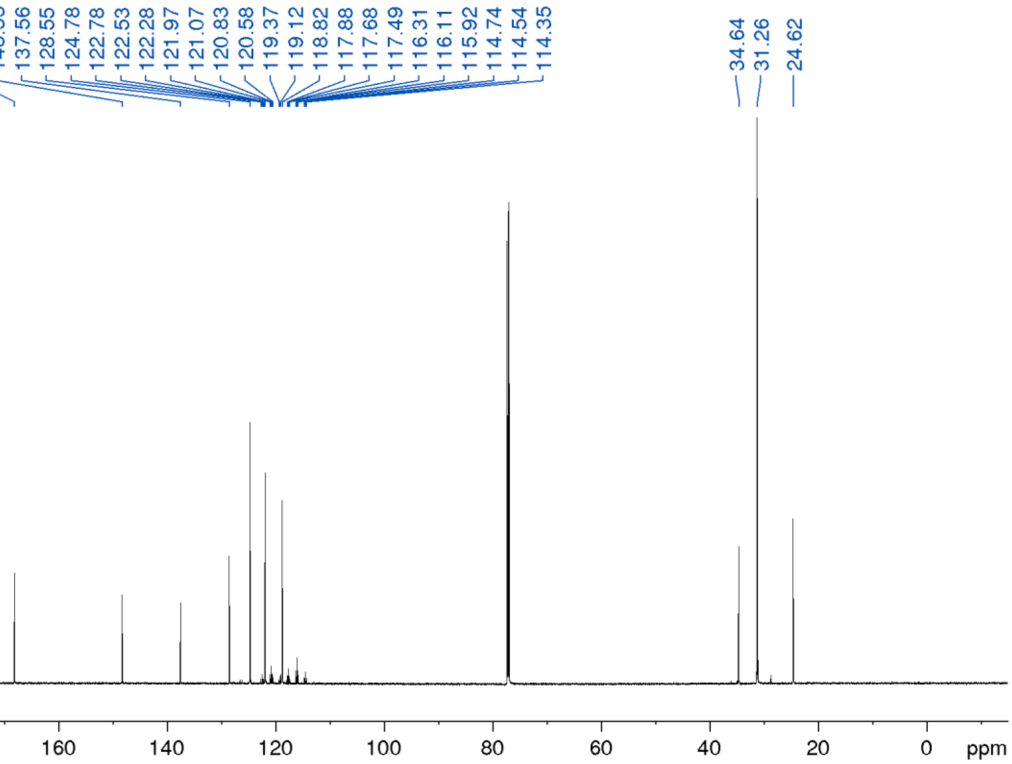

^{19}F NMR (376 MHz, CDCl$_3$, 25 ºC) of (4g)

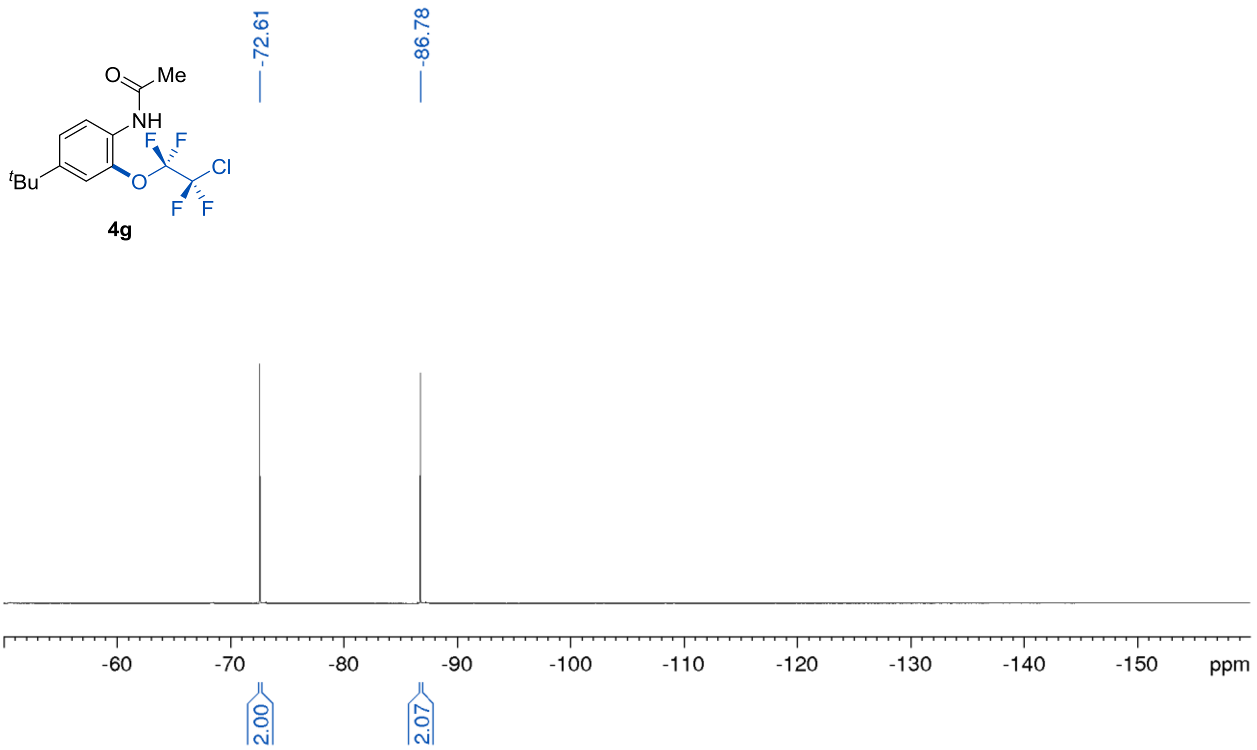

^{1}H NMR (700 MHz, CDCl$_3$, 25 ºC) of (4h)

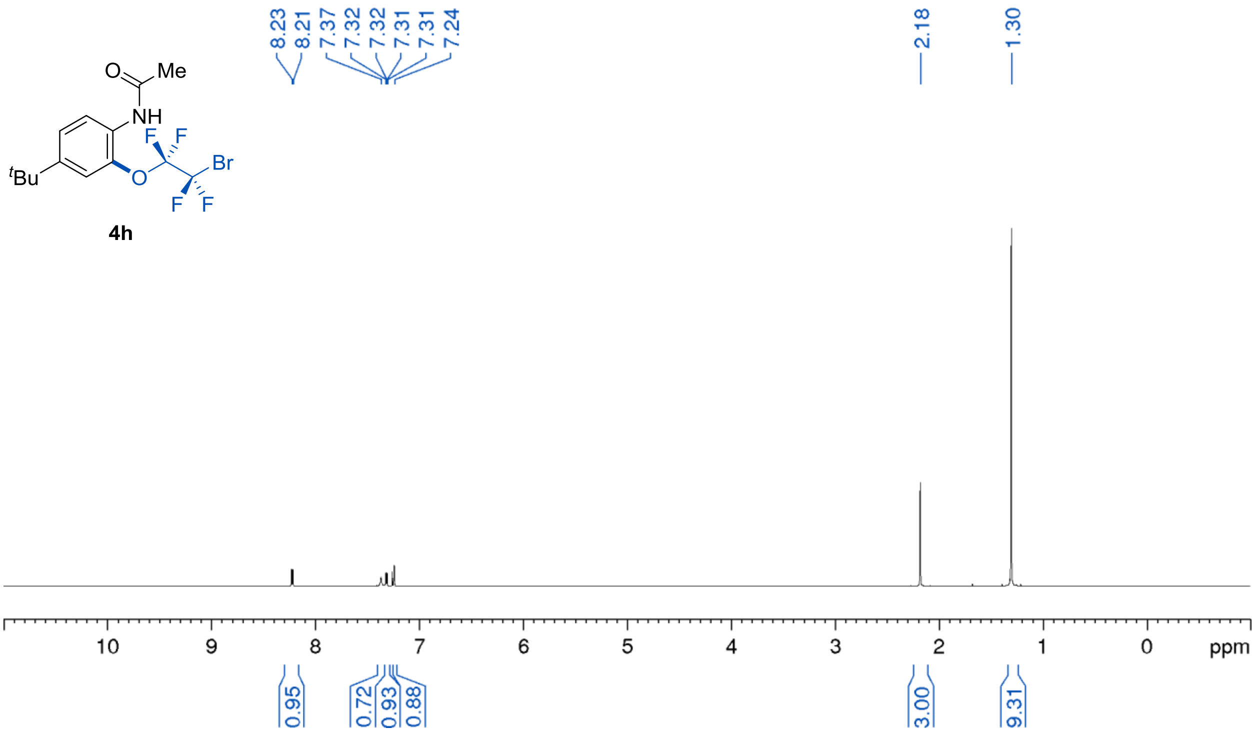

^{13}C NMR (175 MHz, $CDCl_3$, 25 ºC) of (4h)

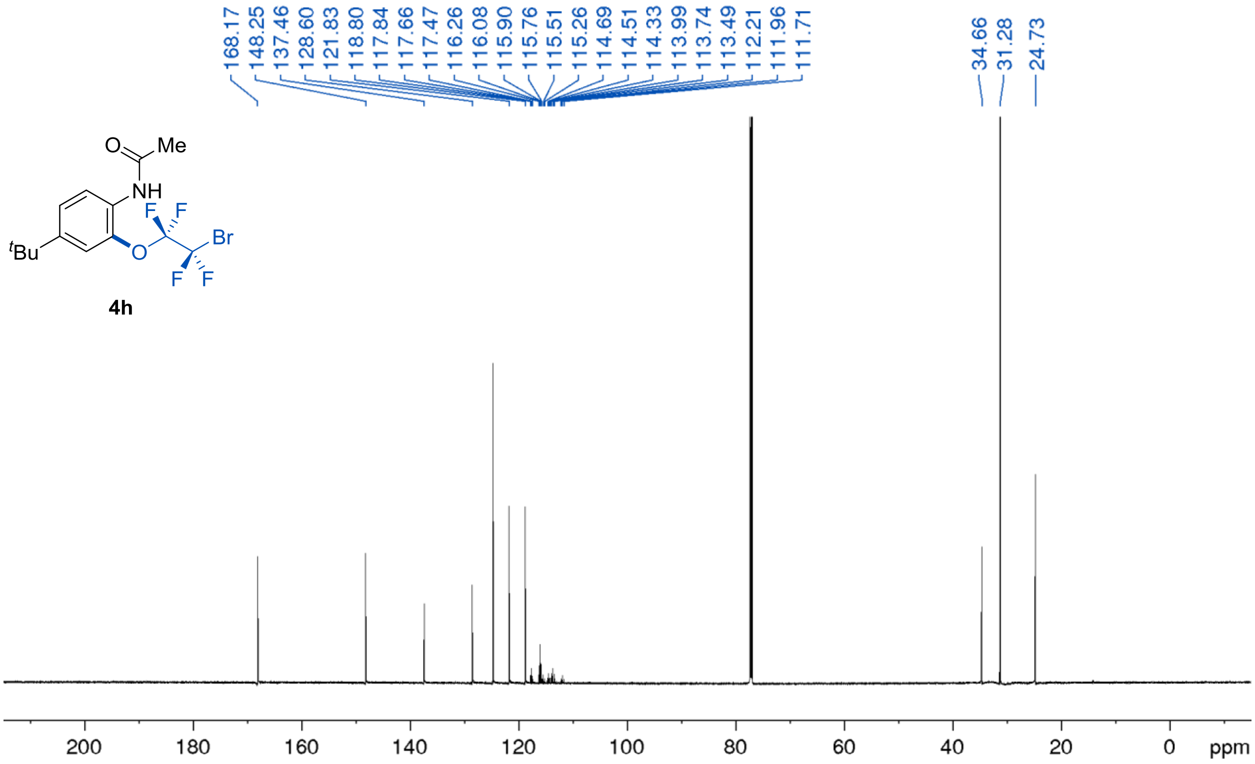

^{19}F NMR (376 MHz, $CDCl_3$, 25 ºC) of (4h)

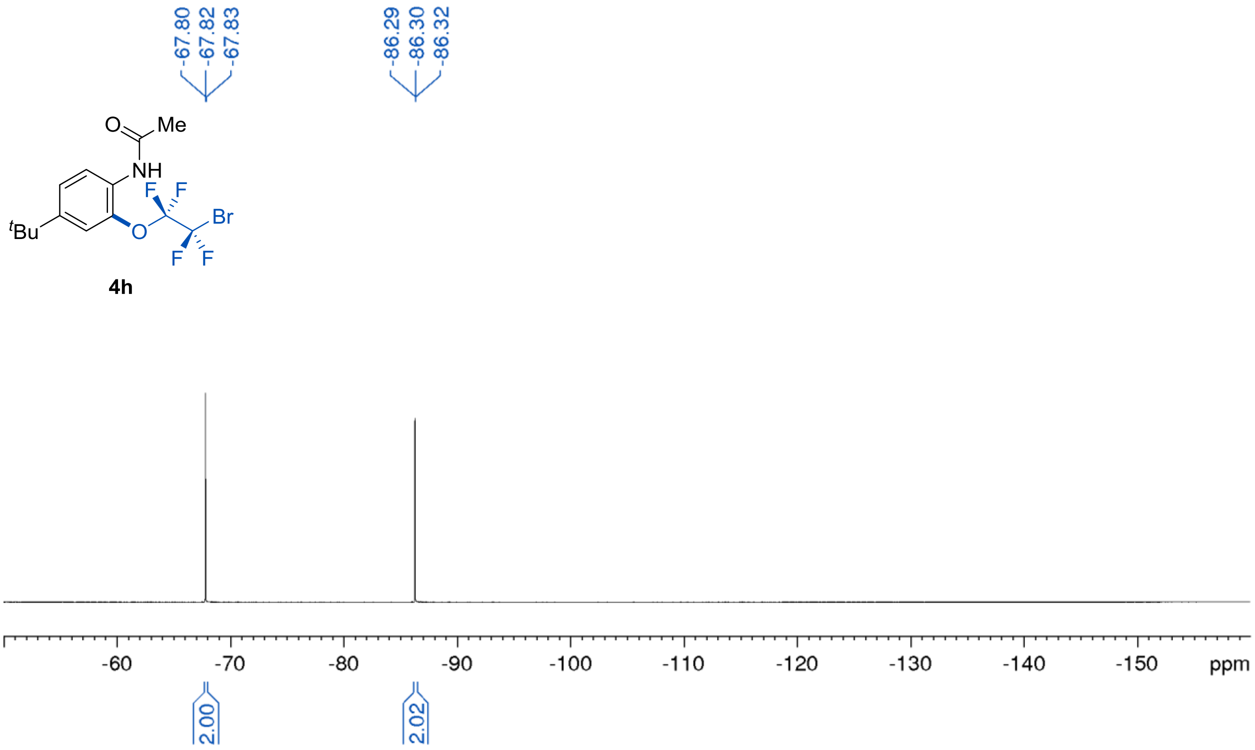

Supplement: Supplementary file 1 [file SC-009-C7SC01684K-s001.pdf]
